# Adverse health effects associated with household air pollution: a systematic review, meta-analysis, and burden estimation study

**DOI:** 10.1016/S2214-109X(20)30343-0

**Published:** 2020-10-15

**Authors:** Kuan Ken Lee, Rong Bing, Joanne Kiang, Sophia Bashir, Nicholas Spath, Dominik Stelzle, Kevin Mortimer, Anda Bularga, Dimitrios Doudesis, Shruti S Joshi, Fiona Strachan, Sophie Gumy, Heather Adair-Rohani, Engi F Attia, Michael H Chung, Mark R Miller, David E Newby, Nicholas L Mills, David A McAllister, Anoop S V Shah

**Affiliations:** a BHF Centre for Cardiovascular Science, University of Edinburgh, Edinburgh, UK; b Usher Institute of Population Health Sciences and Informatics, University of Edinburgh, Edinburgh, UK; c Center for Global Health, Department of Neurology and Department of Sport and Health Sciences, Technical University, Munich, Germany; d Liverpool School of Tropical Medicine, Liverpool, UK; e Department of Public Health and Environment, WHO, Geneva, Switzerland; f Department of Medicine, University of Washington, Seattle, WA, USA; g Department of Medicine, Aga Khan University, Nairobi, Kenya; h Institute of Health and Wellbeing, University of Glasgow, Glasgow, UK; i Department of Non-communicable Disease, London School of Hygiene & Tropical Medicine, London, UK

## Abstract

**Background:**

3 billion people worldwide rely on polluting fuels and technologies for domestic cooking and heating. We estimate the global, regional, and national health burden associated with exposure to household air pollution.

**Methods:**

For the systematic review and meta-analysis, we systematically searched four databases for studies published from database inception to April 2, 2020, that evaluated the risk of adverse cardiorespiratory, paediatric, and maternal outcomes from exposure to household air pollution, compared with no exposure. We used a random-effects model to calculate disease-specific relative risk (RR) meta-estimates. Household air pollution exposure was defined as use of polluting fuels (coal, wood, charcoal, agricultural wastes, animal dung, or kerosene) for household cooking or heating. Temporal trends in mortality and disease burden associated with household air pollution, as measured by disability-adjusted life-years (DALYs), were estimated from 2000 to 2017 using exposure prevalence data from 183 of 193 UN member states. 95% CIs were estimated by propagating uncertainty from the RR meta-estimates, prevalence of household air pollution exposure, and disease-specific mortality and burden estimates using a simulation-based approach. This study is registered with PROSPERO, CRD42019125060.

**Findings:**

476 studies (15·5 million participants) from 123 nations (99 [80%] of which were classified as low-income and middle-income) met the inclusion criteria. Household air pollution was positively associated with asthma (RR 1·23, 95% CI 1·11–1·36), acute respiratory infection in both adults (1·53, 1·22–1·93) and children (1·39, 1·29–1·49), chronic obstructive pulmonary disease (1·70, 1·47–1·97), lung cancer (1·69, 1·44–1·98), and tuberculosis (1·26, 1·08–1·48); cerebrovascular disease (1·09, 1·04–1·14) and ischaemic heart disease (1·10, 1·09–1·11); and low birthweight (1·36, 1·19–1·55) and stillbirth (1·22, 1·06–1·41); as well as with under-5 (1·25, 1·18–1·33), respiratory (1·19, 1·18–1·20), and cardiovascular (1·07, 1·04–1·11) mortality. Household air pollution was associated with 1·8 million (95% CI 1·1–2·7) deaths and 60·9 million (34·6–93·3) DALYs in 2017, with the burden overwhelmingly experienced in low-income and middle-income countries (LMICs; 60·8 million [34·6–92·9] DALYs) compared with high-income countries (0·09 million [0·01–0·40] DALYs). From 2000, mortality associated with household air pollution had reduced by 36% (95% CI 29–43) and disease burden by 30% (25–36), with the greatest reductions observed in higher-income nations.

**Interpretation:**

The burden of cardiorespiratory, paediatric, and maternal diseases associated with household air pollution has declined worldwide but remains high in the world's poorest regions. Urgent integrated health and energy strategies are needed to reduce the adverse health impact of household air pollution, especially in LMICs.

**Funding:**

British Heart Foundation, Wellcome Trust.

## Introduction

WHO estimates that almost 3 billion people worldwide rely on polluting fuels such as wood, coal, crop waste, animal dung, or charcoal paired with inefficient stoves for cooking and heating.^1^ These fuels burn inefficiently, emitting high concentrations of both gaseous and particulate pollutants within households.^2^ There is now considerable evidence linking household air pollution to a broad range of cardiorespiratory,^3^, ^4^, ^5^ paediatric,^6^ and maternal^6^ conditions, with these disease categories specifically highlighted by WHO.^7^ Exposure to household air pollution is among the top ten risk factors for disease, with the highest prevalence observed in the poorest communities in low-income and middle-income countries (LMICs).^8^

Timely and accurate information is urgently needed to facilitate the development of effective global health strategies to curb the adverse health effects associated with household air pollution. Both WHO^1^ and the Global Burden of Disease Study (GBD)^8^ investigators have estimated mortality and morbidity attributable to household air pollution from cardiorespiratory diseases. These estimates were primarily derived using comprehensive integrated exposure–response (IER) curves^9^, ^10^ for particulate matter with a diameter of less than 2·5 μm (PM_2·5_) for each of the risk–outcome pairs. These IERs have been predominantly based on exposure studies evaluating ambient air pollution and smoking,^11^ with only one study evaluating PM_2·5_ concentration from household air pollution.^12^

**Research in context**

**Evidence before this study**

Previous studies have estimated mortality and morbidity attributable to household air pollution. These estimates were derived using comprehensive integrated exposure–response functions that have been predominantly based on studies evaluating the risk of exposure to PM_2·5_ from ambient air pollution and smoking.

**Added value of this study**

We did a comprehensive systematic review and meta-analysis to evaluate the most up-to-date evidence on the adverse health outcomes associated with household air pollution, and calculated the pooled meta-estimates for each cardiovascular, respiratory, paediatric, and maternal outcome. Using a counterfactual of no exposure, we then estimated the trend in global, regional, and national mortality and burden associated with household air pollution.

**Implications of all the available evidence**

Our study highlights the urgent need for evidence-based policy making and decision making to reduce the substantial burden of disease associated with household air pollution, particularly in low-income and middle-income countries. Recent evidence suggests that cleaner-burning biomass-fuelled cookstoves do not deliver the expected health benefits; therefore, future clinical trials should evaluate the impact of cleaner fuel interventions on health outcomes.

In this impact assessment, we did a comprehensive systematic review and meta-analysis to evaluate the most up-to-date evidence on the adverse health outcomes associated with household air pollution. Using the counterfactual of no exposure to household polluting fuels and technologies, we estimated the global, regional, and national mortality and burden.

## Methods

### Search strategy and selection criteria

For this systematic review and meta-analysis, we did a systematic search of Ovid Embase, MEDLINE, and the Global Health and Web of Science for studies evaluating the association between exposure to household air pollution and adverse cardiorespiratory, paediatric health outcomes, and maternal health outcomes. We included all studies of any design published from database inception to April 2, 2020. The full search strategy is included in the appendix (pp 4–8). We identified further studies through searches of bibliographies and references.

We included studies reporting risk of cardiovascular disease, ischaemic heart disease, cerebrovascular disease, asthma, chronic obstructive pulmonary disease (COPD), acute respiratory infection, lung cancer, active pulmonary tuberculosis, low birthweight, stillbirth, or all-cause mortality in people exposed to household air pollution. Household air pollution exposure was defined as use of polluting fuels (coal, wood, charcoal, agricultural wastes, animal dung, or kerosene) for household cooking or heating. All users of polluting fuels were assumed to be exposed to household air pollution because there are currently no scalable efficient stoves.^7^ Studies reporting relative risks (RRs) for outcomes per unit increment of indoor pollutant concentrations, risk in those exposed to gas cooking or heating compared with those unexposed, and evaluating the impact of improved cookstoves were also included. The study methodology, reporting, and presentation were done in accordance with current guidelines (appendix pp 4–8, 54–56).^13^

All studies identified were screened by two investigators and conflicts adjudicated by a third (KKL, RB, JK, SB, NS, DS, AB, SSJ, FS, ASVS). There were no age or language restrictions and only original peer-reviewed articles were included. Studies with the largest participant size were chosen where there were multiple articles from the same cohort. Studies that evaluated a composite of acute cardiovascular or respiratory events that included our outcomes of interest but were not exclusive to these conditions were also included. Where required, authors were contacted for additional data or clarification.

### Prevalence of polluting fuel use and household air pollution associated burden

Annual prevalence estimates of polluting fuel use (based on national survey modelling) from 2000 to 2017 were provided for 183 of the 193 UN member states by WHO.^14^ Annual disability-adjusted life-years (DALYs) and deaths due to cardiorespiratory, paediatric, and maternal outcomes for 2000–17 were available from the Institute of Health Metrics and Evaluation (IHME) for 195 countries and territories.^15^ For 2017, age-standardised national estimates of the number of deaths and DALYs were obtained for each of these outcomes per 100 000 population. We classified each country according to the six WHO regions and World Bank income groups in 2018 (appendix pp 56–60).

### Data analysis

Data extraction with an electronic database was carried out independently by two investigators and conflicts were adjudicated by a third (KKL, RB, JK, SB, NS, DS, AB, SSJ, FS, ASVS). We extracted RRs, 2 × 2 contingency tables, baseline characteristics of the study population, type of fuel or cookstove used in the exposed and comparator group, and detailed characteristics of the study design.

A step-by-step description of the analysis is detailed in the appendix (pp 10–13). In brief, RR meta-estimates for risk–outcome pairs were computed using a random-effects model. Heterogeneity was assessed using the *I*^2^ statistic. Significant heterogeneity was anticipated across the studies and normal probability plots were used to evaluate the distribution of the RRs (appendix pp 14–15). Age and sex interactions were evaluated for the risk of cardiorespiratory disease in adults associated with household air pollution. As interactions were absent or weak, we did not compute age-stratified or sex-stratified cause-specific RRs (appendix pp 10, 16–21). Risk of bias for each study was assessed on the method used for case ascertainment and the degree of confounder adjustment (appendix p 9). Sensitivity analyses of RRs were done for studies at low or moderate risk of bias, longitudinal studies, and studies where the comparator group was only clean fuel use (gas or electricity).

Using a simulation-based approach, we obtained 10 000 samples from a log-normal distribution of the RR meta-estimates and a beta distribution of the prevalence of household air pollution exposure to calculate the cause-specific population attributable fraction and 95% CIs (appendix pp 10–13).^16^ Using cause-specific and year-specific DALY and mortality estimates from IHME and the population attributable fraction, we derived national estimates of disease burden and mortality associated with household air pollution, and combined these to derive global and regional estimates. Sensitivity analyses for disease burden were calculated from RRs restricted to studies where the comparator was specifically clean fuels and those at low or moderate risk of bias. All analyses were done in R (version 3.6.1). This study is registered with PROSPERO, CRD42019125060.

### Role of the funding source

The funder of the study had no role in study design, data collection, data analysis, data interpretation, or writing of the report. The corresponding author had full access to all the data and the final responsibility to submit for publication.

## Results

Our search identified 60 629 studies, with 41 identified through other sources (appendix p 22). After duplicates were removed, titles and abstracts of 46 584 articles were screened, 1646 full-text articles were reviewed, and 476 studies were included in the qualitative synthesis (appendix pp 22, 61–82). Of these, seven were randomised controlled trials, 75 were prospective cohort studies, 139 were case-control studies, 16 were retrospective cohort studies, and 239 were cross-sectional studies. These studies included 15·5 million participants across 123 countries, of which the majority (99 [80%] countries) were LMICs. 14 studies were excluded from the quantitative analysis owing to insufficient data.

**Figure 1:**
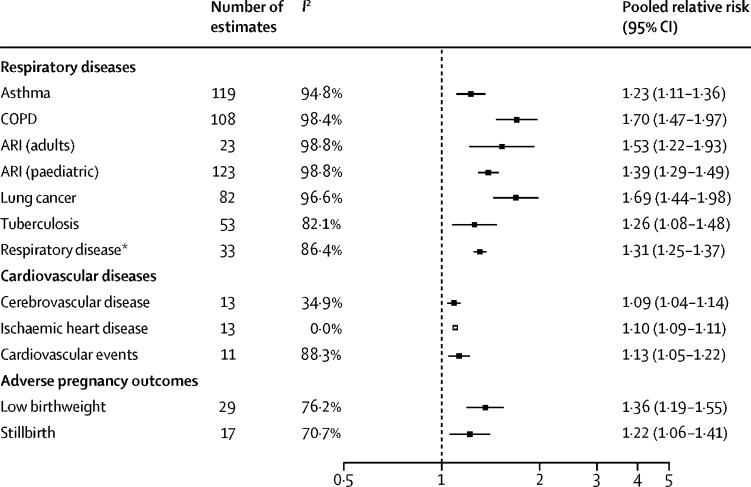
Pooled relative risks for cardiovascular, respiratory, and adverse pregnancy outcomes associated with use of polluting fuels and technologies ARI=acute respiratory infection. COPD=chronic obstructive pulmonary disease. *Composite estimate from studies that did not break down their findings by constituent respiratory disease.

267 articles provided 541 estimates to derive pooled RRs for respiratory diseases in people exposed to polluting fuels and technologies compared with those unexposed (figure 1). The pooled RRs showed increased risk of asthma, COPD, acute respiratory infection in adults and children, lung cancer, and pulmonary tuberculosis, with the highest RR for COPD (figure 1). Positive associations were also observed for ischaemic heart disease and cerebrovascular disease (figure 1).

19 articles provided 58 estimates for asthma or respiratory symptoms per unit increment in nitrogen dioxide (NO_2_) and PM_2·5_. Both NO_2_ (per 10 parts per billion) and PM_2·5_ (per 10 μg/m^3^) were associated with cough (1·03 [95% CI 1·00–1·05] and 1·01 [1·00–1·02], respectively) and dyspnoea (1·23 [1·06–1·38] and 1·01 [1·00–1·01], respectively; appendix pp 41–42). Across 31 studies, gas cooking or heating was associated with asthma (1·17 [1·07–1·29]; appendix p 35). 23 articles, including seven randomised controlled trials, reported RRs of respiratory disease (0·59 [0·45–0·77] for acute respiratory infection and 0·69 [0·45–1·04] for asthma) or symptoms (0·67 [0·54–0·84]) in people using improved cookstoves compared with traditional stoves (appendix p 36). Of these, the largest randomised controlled trial in 10 750 children did not show any reduction in acute respiratory infections.^17^

26 studies provided 46 estimates for adverse maternal outcomes (appendix p 30). The pooled RR was 1·36 (95% CI 1·19–1·55) for low birthweight and 1·22 (1·06–1·41) for stillbirth. Infants born to mothers exposed to polluting fuels and technologies were an average of 149 g (95% CI 101–196) lighter at birth (appendix p 40).

**Figure 2:**
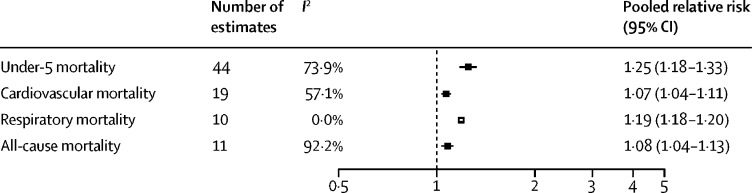
Pooled relative risks for under-5, cardiovascular, respiratory, and all-cause mortality associated with use of polluting fuels and technologies

35 studies provided 84 estimates for mortality in people exposed to polluting fuels and technologies. The pooled RR showed increased risk of under-5 mortality (figure 2). Exposure to polluting fuels and technologies also increased the risk of cardiovascular, respiratory, and all-cause mortality (figure 2).

Forest plots for each of the individual endpoints are presented in the appendix (pp 23–42). Sensitivity analyses restricted to studies with low or moderate risk of bias, with a longitudinal design, and in which the comparator was specifically clean fuels attenuated the overall effect of some estimates, with the effect direction mostly remaining unchanged (appendix pp 43–46).

**Figure 3:**
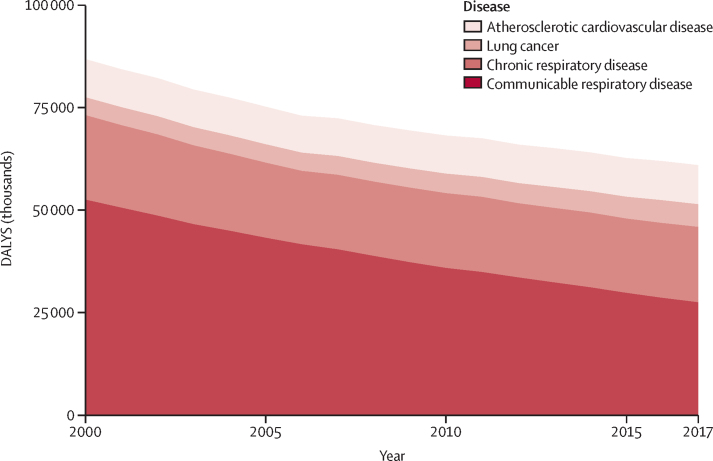
Trends in cause-specific burden of disease attributable to household air pollution, 2000–17 DALYs=disability-adjusted life-years.

In 2017, household air pollution contributed to 1·8 million (95% CI 1·1–2·7) deaths and 60·9 million (34·6–93·3) DALYs globally. Among these, respiratory disease was the leading cause of death and DALYs attributable to household air pollution accounted for 38% of all deaths (0·7 million [0·4–1·0]) and 75% of all DALYs (45·7 million [26·8–68·8]; figure 3; appendix p 84). Communicable respiratory disease (acute respiratory infection and pulmonary tuberculosis) accounted for most of the respiratory burden (27·4 million [16·4–41·0] DALYs), followed by chronic respiratory disease (18·4 million [10·4–27·8] DALYs for asthma and COPD) and lung cancer (5·5 million [2·8–9·0] DALYs; appendix p 84). Cardiovascular disease (ischaemic heart disease and cerebrovascular disease) accounted for 0·3 million (0·1–0·6) deaths and 9·5 million (5·0–15·6) DALYs.

**Figure 4:**
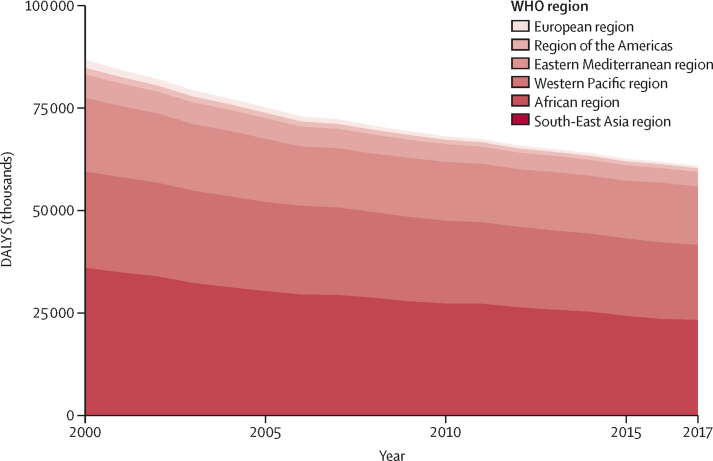
Trends in burden of disease attributable to household air pollution stratified by WHO region, 2000–17 DALYs=disability-adjusted life-years.

**Figure 5:**
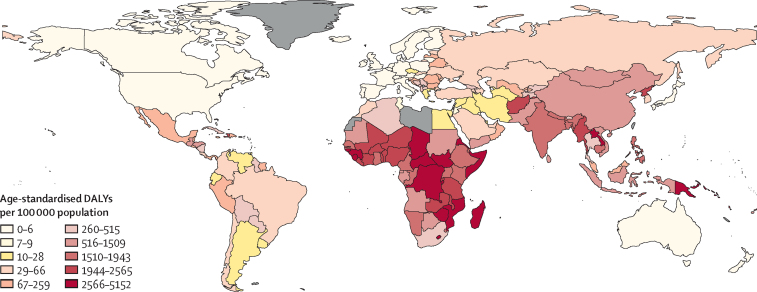
Age-standardised DALY rates attributable to household air pollution by country, 2017 Countries for which data were not available are shown in grey. DALY=disability-adjusted life-year.

In 2017, disease burden associated with household air pollution was almost exclusively concentrated in LMICs (60·8 million [95% CI 34·6–92·9] DALYs and 1·8 million [1·1–2·7] deaths *vs* 0·09 million [0·01–0·40] DALYs and 0·002 million [0·000–0·009] deaths in high-income countries; appendix pp 47–49, 85). The South-East Asia region had the highest burden due to household air pollution with 23·3 million (13·7–34·4) DALYs and 0·57 million (0·34–0·84) deaths, followed by the African region (18·3 million [11·8–26·2] DALYs and 0·72 million [0·51–0·97] deaths) and the Western Pacific region (14·2 million [6·4–23·6] DALYs and 0·33 million [0·14–0·57] deaths; figure 4; appendix pp 50, 86). The country with the highest burden was India (17·3 million [10·0–25·5] DALYs and 0·4 million [0·3–0·6] deaths) followed by China (11·7 million [5·0–19·6] DALYs and 0·3 million [0·1–0·5] deaths) and Nigeria (5·0 million [3·4–7·1] DALYs and 0·2 million [0·1–0·3] deaths; appendix pp 48, 87–93). Countries in the African region had the highest age-standardised disease burden per 100 000 population (figure 5; appendix pp 87–93).

Between 2000 and 2017, deaths and disease burden associated with household air pollution steadily reduced, by 36% (95% CI 29–43) and 30% (25–36), respectively (figure 3; appendix pp 49–50). The greatest relative reduction in DALYs occurred in the European region (71%) with the lowest reductions in the Western Pacific (21%) and African (22%) regions. India and China had the greatest absolute reduction in DALYs from 2000 to 2017 (8·0 million and 3·1 million, respectively; appendix p 86). DALYs attributable to household air pollution due to communicable respiratory disease reduced by 48% between 2000 and 2017 but increased by 27% for lung cancer and by 2% for ischaemic heart disease (appendix p 8).

Between 2000 and 2017, under-5 mortality attributable to household air pollution decreased by 50% from 1·6 million (95% CI 1·1–2·0) to 0·78 million (0·52–1·09) deaths (appendix p 51). Despite this reduction, under-5 mortality accounted for more than a third of all deaths associated with household air pollution in 2017, with most occurring in LMICs within the African (0·50 million [0·35–0·68]) and South-East Asia (0·16 million [0·10–0·23]) regions.

In our sensitivity analysis, using pooled RRs from studies where the comparator was clean fuel use, the estimated global burden associated with household air pollution was reduced to 49·1 million (95% CI 13·7–92·0) DALYs (appendix p 52). Using the pooled RRs from studies assessed to be at low or moderate risk of bias, the global burden estimate was 58·7 million (95% CI 21·2–101·9) DALYs (appendix p 53).

## Discussion

In this impact assessment evaluating the adverse health effects of household air pollution, we report several important observations. First, household air pollution is associated with an increased risk of adverse health effects, with the strongest association observed for respiratory diseases such as COPD and lung cancer. Second, in 2017, household air pollution was associated with 1·8 million deaths and 60·9 million DALYs globally. Third, under-5 mortality attributable to household air pollution halved between 2000 and 2017 but still accounted for more than a third of all deaths associated with household air pollution, the majority of which occurred in the African region. Fourth, burden associated with household air pollution has reduced by a third since 2000. Burden of communicable diseases associated with household air pollution, including acute respiratory infection and tuberculosis, has nearly halved but has increased for lung cancer and ischaemic heart disease. Finally, there was clear geographical variation, with the overwhelming majority of burden associated with household air pollution seen in LMICs.

Our global burden estimates lie between those previously reported by WHO (3·8 million deaths in 2016)^1^ and GBD (1·6 million deaths in 2017),^18^ which probably reflects the different methodological approaches taken. The WHO and GBD approaches used two components to estimate the burden: disease-specific IER functions and sex-specific and children-specific estimates of PM_2·5_ exposure from household air pollution.^9^, ^11^ Comprehensive modelling approaches were employed to derive the IER functions for PM_2·5_ concentration.^9^ RRs for household air pollution exposure were then derived from the outcome-specific IER functions comparing the risk from estimated household PM_2·5_ exposures against a “counterfactual low PM_2·5_ concentration”, based on expert consensus.^9^, ^11^, ^19^ The IER functions were derived from studies primarily originating from high-income nations that evaluated exposure to ambient air pollution and smoking.^9^ PM_2·5_ exposure estimates due to household air pollution were initially derived from households in India and subsequently from studies in the WHO Global Household Air Pollution database.^20^, ^21^ By contrast, our methodology derived RRs for each risk–outcome pair using studies that have defined polluting fuel use as a binary indicator for exposure. Our review identified more than 400 studies, with only a fraction (n=24) measuring personal exposure concentrations. Consistent with the body of published literature, our study evaluated the risk for each health outcome exposed to polluting fuels and technologies against the counterfactual of no exposure. Furthermore, GBD estimated burden from exposure to solid fuels.^9^ Contrary to this, both we and WHO defined kerosene exposure as a polluting fuel.^7^ Using this definition, we identified 76 studies evaluating exposure to kerosene use, further explaining our higher burden estimates compared with GBD.^18^

Our rationale for a counterfactual of no exposure was multifactorial. First, only 5% of the studies identified had directly measured pollutant concentrations, with the majority of these evaluating symptoms rather than clinical outcomes and originating from high-income countries (appendix). Second, previous burden estimates^1^, ^8^ for household air pollution were based on IER functions predominantly derived from studies in high-income countries and primarily evaluating exposure to ambient air pollution and smoking rather than household air pollution.^9^ This assumes that the adverse health effects of PM_2·5_ from household air pollution are comparable to those from other sources.^22^ Our choice of counterfactual attempts to estimate the specific risk associated with exposure to all household polluting fuels and technology. Third, WHO recommends a direct transition from polluting fuels and technologies to clean fuels.^7^ Although several trials have shown some potentially beneficial impact from the implementation of cleaner burning biomass-fuelled cookstoves, a randomised controlled trial showed no improvement in outcomes.^17^ Our choice of the counterfactual reflects this policy^7^ and attempts to estimate the burden of disease avoidable if such a transition were achieved. Fourth, alternative counterfactuals using theoretical minimum risk exposure thresholds are appropriate when estimating burden due to ambient air pollution, given the extensive ambient air quality monitoring systems in place and the literature evaluating health effects of directly measured ambient air pollutant concentrations.^23^, ^24^ These well developed ambient air quality monitoring systems are therefore able to monitor air quality to ensure compliance with national and international thresholds.^7^ However, monitoring indoor air quality at scale, especially across LMICs, to ensure compliance with recommended minimum thresholds is likely to be financially and logistically prohibitive.^20^ This is reflected in the paucity of personal exposure studies evaluating health outcomes associated with household air pollution. Conversely, determining indoor air pollution exposure using questionnaire-based systems is practically more feasible, and has been shown to reflect exposures accurately.^25^

Several limitations need to be taken into account when interpreting our results. First, a binary indicator as a proxy for exposure does not take into account heterogeneity of exposure,^20^ which is likely to vary by fuel type, frequency and duration of exposure, and ventilation. Additionally, although some studies clearly stated the comparator group as those exposed to clean fuels, other studies defined the comparator as no exposure to a specific polluting fuel. As such, our approach increases the risk of exposure misclassification and is unable to show a dose–response relationship. Using these meta-estimates in our burden estimates does not account for the indirect health impact of household air pollution, such as its contribution to ambient air pollution. Second, the majority of studies included in this meta-analysis were observational, with the level of adjustment varying considerably. One of the key factors that has hindered adoption of cleaner fuels in LMICs is affordability, compounded by issues with reliable supply chain and local cultural practices.^19^, ^26^ Unmeasured confounding might have affected the comparability between those exposed versus unexposed in our study. Sensitivity analysis restricted to studies that used the comparator of clean fuel use or studies at low risk of bias did attenuate the overall burden. Third, many studies in our review were cross-sectional and therefore did not establish a temporal relationship between exposure to household air pollution and adverse health outcome. The pooled RR estimates were attenuated in a sensitivity analysis restricted to only longitudinal studies. Fourth, we did not have access to individual participant-level data and therefore had to assume homogeneity of risk across all study participants for our health outcomes of interest. Finally, we observed significant heterogeneity in the risk estimates, and for several outcomes, such as atherosclerotic cardiovascular disease, the underlying distribution of the risk estimates did not conform to a normal distribution. Caution should therefore be exercised when interpreting the pooled risk estimates and highlights the need for more high-quality data to increase the certainty of these risk estimates.

Overall, the burden of disease attributable to household air pollution has declined over the past two decades, with clear geographical variation. The burden of disease from household air pollution is almost exclusively borne by LMICs where there is little access to electricity or gas cooking. Paradoxically, the greatest relative reduction in burden attributable to household air pollution was observed in Europe where the burden was the lowest. The African and Western Pacific regions, where the burden of disease due to household air pollution is much higher, have experienced a modest reduction in burden over the past two decades. The patterns of disease due to household air pollution have also evolved substantially, with communicable respiratory disease associated with household air pollution nearly halved but non-communicable diseases such as lung cancer and cardiovascular disease increasing. This is of particular concern given that air pollution is now the second most important risk factor for non-communicable disease globally, and in many countries, particularly in the South-East Asia region, it has overtaken tobacco smoking as the largest risk factor.^1^, ^15^ Furthermore, incidence and deaths due to non-communicable diseases are projected to rise substantially in LMICs over the next few decades.^27^

Overall, deaths due to household air pollution have declined by nearly a third since 2000, although the total number remains substantial at 1·8 million in 2017 alone. Respiratory mortality due to household air pollution has declined more modestly but appears to have plateaued in recent years. Conversely, cardiovascular mortality has marginally increased. Most encouraging was a marked decline in under-5 mortality, which halved during the past two decades but still remains unacceptably high, with the majority of burden borne by LMICs. There are several reasons why under-5 mortality attributable to household air pollution remains so pervasive. First, younger children, particularly those who are still breastfeeding, are more likely to remain indoors to be in close proximity to their mothers, resulting in greater exposure.^21^ Second, infants born to mothers exposed to polluting fuels were more likely to have low birthweight and this in itself increases the susceptibility of infants to complications such as hypothermia,^28^ cardiorespiratory abnormalities,^29^, ^30^ and infections.^31^ Third, exposure to household air pollution is likely to reflect a more deprived socioeconomic status with little access to both cleaner fuels and health care, further contributing to under-5 deaths.^32^ As such, poverty remains a dominant mediator not only in preventing access to clean household energy but also exacerbating poor access to health care, compounding the burden associated with household air pollution.^33^

Achieving universal access to clean fuels and technologies for cooking by 2030 is a key element of the UN Sustainable Development Goals.^34^ Government intervention through targeted policy making and investments can accelerate the adoption of clean cooking solutions. Clean fuel subsidies in China, India, and Indonesia have already resulted in substantial reductions in people without access to clean cooking.^35^, ^36^, ^37^ Although a direct transition to clean fuels remains the ideal solution, in many regions, progress is slow. Acknowledging these challenges, WHO guidelines recommend where access to clean fuels and technologies remains near impossible, more advanced combustion cookstoves offering some health benefits should be prioritised in the transition to clean cooking solutions.^7^ Recent evidence, however, suggests that cleaner burning biomass-fuelled cookstoves do not deliver the expected health benefits.^17^ Our analysis further highlights the urgent need for clinical trials evaluating cleaner fuel interventions on health outcomes to underpin evidence-based policy and decision making.

In conclusion, our analysis shows that household air pollution increases the risk of a wide range of adverse cardiorespiratory, paediatric, and maternal health outcomes. Although burden of disease due to household air pollution has declined by a third since 2000, the adverse health effects remain pervasive in LMICs in the South-East Asia and African regions. There is an urgent need for evidence-based policy and decision making to ensure children and adults living in LMICs have clean air to breathe in their homes.

For the **WHO regions** see https://www.who.int/about/who-we-are/regional-offices

For more on **World Bank income groups** see https://datahelpdesk.worldbank.org

For the **R code and data assets** see https://github.com/kk-lee/HAP

## Data sharing

The R code and data assets permissible for sharing are available in a public repository.
