# Supplementary material for: Adverse health effects associated with household air pollution: a systematic review, meta-analysis, and burden estimation study
Source: Lancet Glob Health. 2020 Oct 15;8(11):e1427–34. doi: 10.1016/S2214-109X(20)30343-0 (PMC7564377; doi:10.1016/S2214-109X(20)30343-0)
Supplement: Supplementary appendix [file mmc1.pdf]

THE LANCET
Global Health

Supplementary appendix

This appendix formed part of the original submission and has been peer reviewed. We post it as supplied by the authors.

Supplement to: Lee KK, Bing R, Kiang J, et al. Adverse health effects associated with household air pollution: a systematic review, meta-analysis, and burden estimation study. *Lancet Glob Health* 2020; **8:** e1427–34.

DATA SUPPLEMENT

Adverse health effects associated with household air pollution: a systematic review, meta-analysis, and burden estimation study

Kuan Ken Lee MD[1], Rong Bing MD[1], Joanne Kiang BSc[1], Sophia Bashir BSc[1], Nicholas Spath MD[1], Dominik Stelzle MD[2,3], Kevin Mortimer PhD[4], Anda Bularga MD[1], Dimitrios Doudesis MSc[1,5], Shruti S Joshi MD[1], Fiona Strachan PhD[1], Sophie Gumy PhD[6], Heather Adair-Rohani MPH[6], Engi F Attia MD[7], Michael H Chung MD[8], Mark R Miller PhD[1], David E Newby MD[1], Nicholas L Mills MD[1,5], David A McAllister MD*[9] and Anoop SV Shah MD*[10]

[1]BHF Centre for Cardiovascular Science, University of Edinburgh, United Kingdom
[2]Center for Global Health, Department of Neurology, Technical University, Munich, Germany
[3]Chair of Epidemiology, Department of Sport and Health Sciences, Technical University of Munich, Germany
[4]Liverpool School of Tropical Medicine, Liverpool
[5]Usher Institute of Population Health Sciences and Informatics, University of Edinburgh, United Kingdom
[6]Department of Public Health and Environment, World Health Organization, Geneva, Switzerland
[7]Department of Medicine, University of Washington, Seattle, USA
[8]Department of Medicine, Aga Khan University, Nairobi, Kenya
[9]Institute of Health and Wellbeing, University of Glasgow, United Kingdom
[10]Department of non-communicable disease, London School of Hygiene and Tropical Medicine, United Kingdom
**Contributed equally*

Correspondence and requests for reprints:
Dr Anoop SV Shah
Department of non communicable disease
Keppel St,
Bloomsbury,
London
WC1E 7HT

Mobile: +44 7766 544156
E-mail: anoopsshah@gmail.com

Table of contents

Supplementary text 1. Literature search strategy 4

Supplementary text 2. Risk of bias assessment criteria 9

Supplementary text 3. Method for calculating burden and mortality attributable to household air pollution 10

Figure S1: Normal probability plots of the risk ratios for cardiorespiratory and adverse pregnancy outcomes associated with use of polluting fuels and technologies 14

Figure S2: Normal probability plots of the risk ratios for mortality associated with use of polluting fuels and technologies 15

Figure S3: Meta-regression for COPD by mean age 16

Figure S4: Meta-regression for Asthma by mean age 17

Figure S5: Meta-regression for Tuberculosis by mean age 18

Figure S6: Meta-regression for lung cancer by mean age 19

Figure S7: Meta-regression for cardiovascular disease by mean age 20

Figure S8: Pooled risk ratios for cardiorespiratory conditions associated with use of polluting fuels and technologies stratified by gender 21

Figure S9: Flow diagram of study selection 22

Figure S10: Pooled risk ratio for asthma associated with use of polluting fuels and technologies 23

Figure S11: Pooled risk ratio for chronic obstructive pulmonary disease associated with use of polluting fuels and technologies 24

Figure S12: Pooled risk ratio for acute respiratory infection in children associated with use of polluting fuels and technologies 25

Figure S13: Pooled risk ratio for acute respiratory infection in adults associated with use of polluting fuels and technologies 26

Figure S14: Pooled risk ratio for lung cancer associated with use of polluting fuels and technologies27

Figure S15: Pooled risk ratio for tuberculosis associated with use of polluting fuels and technologies 28

Figure S16: Pooled risk ratio for cardiovascular disease associated with use of polluting fuels and technologies 29

Figure S17: Pooled risk ratio for low birthweight associated with use of polluting fuels and technologies 30

Figure S18: Pooled risk ratio for under-five mortality associated with use of polluting fuels and technologies 31

Figure S19: Pooled risk ratio for cardiovascular mortality associated with use of polluting fuels and technologies 32

Figure S21: Pooled risk ratio for all-cause mortality associated with use of polluting fuels and technologies 34

Figure S22: Pooled risk ratio for respiratory disease associated with use of gas for cooking or heating 35

Figure S23: Pooled risk ratios for respiratory conditions and symptoms in studies comparing use of improved cook stoves and traditional cook stoves 36

Figure S24: Pooled risk ratio for symptoms of wheeze associated with use of polluting fuels and technologies 37

Figure S25: Pooled risk ratio for symptoms of cough associated with use of polluting fuels and technologies 38

Figure S26: Pooled risk ratio for symptoms of shortness of breath associated with use of polluting fuels and technologies 39

Figure S27: Pooled mean difference in birth weight associated with use of polluting fuels and technologies. 40
Figure S28: Pooled risk ratio for asthma and respiratory symptoms per 10 ppb increase in indoor nitrogen dioxide. 41
Figure S29: Pooled risk ratio for asthma and respiratory symptoms per 10 µg/m3 increase in indoor PM2.5 42
Figure S30: Pooled risk ratios for cardiorespiratory and adverse pregnancy outcomes associated with use of polluting fuels and technologies across studies assessed to be at low or moderate risk of bias. 43
Figure S31: Pooled risk ratios for mortality associated with use of polluting fuels and technologies across studies assessed to be at low or moderate risk of bias. 44
Figure S32: Sensitivity analysis of risk ratios for cardiorespiratory and adverse pregnancy outcomes associated with use of polluting fuels and technologies across longitudinal studies. 45
Figure S33: Pooled risk ratios for cardiorespiratory and paediatric conditions in studies comparing use of polluting fuels or technologies and clean fuels 46
Figure S34: Trends in burden of disease (disability adjusted life years) attributable to household air pollution stratified by 2018 World Bank income groups from 2000-2017. 47
Figure S35: Cartogram of global burden of disease attributable to household air pollution in 2017... 48
Figure S36: Trends in deaths attributable to household air pollution stratified by income 49
Figure S37: Trends in deaths attributable to household air pollution stratified by WHO regions 50
Figure S38: Trends in cardiovascular, respiratory and under five mortality attributable to household air pollution 51
Figure S39: Trends in burden attributable to household air pollution using pooled risk ratios from studies where the comparator was clean fuel use stratified by WHO regions 52
Figure S40: Trends in burden attributable to household air pollution using pooled risk ratios from studies that were assessed to be at low or moderate risk of bias stratified by WHO regions 53
Table S1: PRISMA Checklist 54
Table S2: List of countries and World Bank income group stratified by WHO regions 56
Table S3: Baseline details of included studies 61
Table S4: Trends in cause-specific burden of disease attributable to household air pollution from 2000-2017 83
Table S5: Trends in burden of disease attributable to household air pollution stratified by 2018 World Bank income groups from 2000-2017 84
Table S6: Trends in burden of disease attributable to household air pollution stratified by WHO regions from 2000-2017 85
Table S7: Country-level estimates of burden of disease and deaths attributable to household air pollution in 2017 86
References 93

Supplementary text 1. Literature search strategy

MEDLINE (up to 2nd Apr 2020: 18,807 search results)

1. Air Pollution, Indoor/
2. ((household or indoor*) adj2 air adj2 (pollut* or quality)).tw.
3. ((indoor or household or domestic or home) adj3 (fire or fires or smok* or woodsmok*)).tw.
4. (time* adj5 (stove or cook*)).tw.
5. ((combust* or burn*) adj3 (stove* or wood or kerosene or biomass or coal or charcoal or dung or crop waste* or agricultur* residu*)).tw.
6. (stove* or cookstove* or kitchen ventilat* or combustion or house construction or solar).tw.
7. (vent* adj5 (home* or living space* or household* or kitchen* or cook* or stove* or window* or hood* or grate*)).tw.
8. ((improv* or replac* or chang* or reduc*) adj5 (fuel* or biogas* or burn* or ethanol* or gas or electric* or energy or lamp* or kerosene or fire* or smok* or dung or cook* or chula or chulla or coal or paraffin or charcoal or crop waste* or agricultural residue* or combustion)).tw.
9. or/1-8
10. exp Myocardial Infarction/
11. exp Coronary Thrombosis/
12. Cardiovascular Disease.mp.
13. acute coronary.mp.
14. Myocardial infarct$.mp.
15. heart infarct.mp.
16. acs.mp.
17. ami.mp.
18. (coronary adj3 syndrome$).mp.
19. cerebrovascular disorders/ or basal ganglia cerebrovascular disease/ or brain ischemia/ or exp brain infarction/ or hypoxia-ischemia, brain/ or carotid artery diseases/ or carotid artery thrombosis/ or carotid artery, internal, dissection/ or intracranial arterial diseases/ or cerebral arterial diseases/ or infarction, anterior cerebral artery/ or infarction, middle cerebral artery/ or infarction, posterior cerebral artery/ or exp "intracranial embolism and thrombosis"/ or exp stroke/ or vertebral artery dissection/
20. (isch?emi$ adj5 (stroke$ or apoplex$ or cerebral vasc$ or cerebrovasc$ or cva)).tw.
21. ((brain or cerebr$ or cerebell$ or vertebrobasil$ or hemispher$ or intracran$ or intracerebral or infratentorial or supratentorial or middle cerebr$ or mca$ or anterior circulation or basilar artery or vertebral artery) adj5 (isch?emi$ or infarct$ or thrombo$ or emboli$ or occlus$ or hypoxi$)).tw.
22. exp Pulmonary Disease, Chronic Obstructive/
23. (obstruct$ adj3 (lung$ or respirat$ or pulmonar$) adj3 disease$).mp.
24. Bronchiti$.mp.
25. emphysema$.mp.
26. (chronic adj5 obstruct$).mp.
27. (pulmonar$ or lung$ or airway$ or airflow$ or bronch$ or respirat$).mp.
28. 26 and 27
29. (COPD or COAD).mp.
30. AECB.mp.
31. exp asthma/
32. asthma?.ti,ab,kw.
33. exp tuberculosis/
34. (tuberculosis or TB).mp.
35. exp pneumonia/
36. exp respiratory tract infection$/
37. (pneumonia$ or lung inflammation$ or respiratory tract infection$ or respiratory infection$).mp.
38. (acute respiratory infection* or acute respiratory tract infection* or lower respiratory infection* or lower respiratory tract infection*).tw.
39. (ari or lrti or alri).tw.
40. bronchitis/ or exp bronchiolitis/
41. (bronchiolit* or acute bronchit* or wheez*).tw.
42. Respiratory Syncytial Virus Infections/
43. respiratory syncytial viruses/ or respiratory syncytial virus, human/
44. (respiratory syncytial virus* or rsv).tw.
45. exp Haemophilus influenzae/
46. Streptococcus pneumoniae/
47. Staphylococcus aureus/
48. (streptococc* or staphylococc* or haemophilus influenzae).tw.
49. ((lung or pulmonary) adj2 (inflamm* or infect*)).tw.

50. (low adj3 (birthweight$ or birth weight$)).ti,ab.
51. (lbw or vlbw or elbw).ti,ab.
52. Stillbirth/
53. Fetal death/
54. Foetal death/
55. fetal death$.tw.
56. foetal death$.tw.
57. perinatal death$.tw.
58. intrauterine death$.tw.
59. intrauterine fetal death$.tw.
60. intrauterine foetal death$.tw.
61. perinatal fetal death$.tw.
62. perinatal foetal death$.tw.
63. fetal mortality.tw.
64. foetal mortality.tw.
65. intrauterine fetal mortality.tw.
66. intrauterine foetal mortality.tw.
67. perinatal fetal mortality.tw.
68. perinatal foetal mortality.tw.
69. pregnancy loss.tw.
70. exp Mortality/
71. mortality.tw,ot.
72. mortaliti$.tw,ot.
73. exp lung cancer/
74. (pulmonar$ or lung$ or airway$ or airflow$ or bronch$ or respirat$).mp.
75. (cancer$ or neoplasm$ or tumour$ or tumour$).mp.
76. 74 and 75
77. 10 or 11 or 12 or 13 or 14 or 15 or 16 or 17 or 18 or 19 or 20 or 21 or 22 or 23 or 24 or 27 or 28 or 29 or 30 or 31 or 32 or 33 or 34 or 35 or 36 or 37 or 38 or 39 or 40 or 41 or 42 or 43 or 44 or 45 or 46 or 47 or 48 or 49 or 50 or 51 or 52 or 53 or 54 or 55 or 56 or 57 or 58 or 59 or 60 or 61 or 62 or 63 or 64 or 65 or 66 or 67 or 68 or 69 or 72 or 73 or 76
78. 9 and 77
79. limit 78 to humans

EMBASE (up to 2nd Apr 2020: 23,519 search results)

1. Air Pollution, Indoor/
2. ((household or indoor*) adj2 air adj2 (pollut* or quality)).tw.
3. ((indoor or household or domestic or home) adj3 (fire or fires or smok* or woodsmok*)).tw.
4. (time* adj5 (stove or cook*)).tw.
5. ((combust* or burn*) adj3 (stove* or wood or kerosene or biomass or coal or charcoal or dung or crop waste* or agricultur* residu*)).tw.
6. (stove* or cookstove* or kitchen ventilat* or combustion or house construction or solar).tw.
7. (vent* adj5 (home* or living space* or household* or kitchen* or cook* or stove* or window* or hood* or grate*)).tw.
8. ((improv* or replac* or chang* or reduc*) adj5 (fuel* or biogas* or burn* or ethanol* or gas or electric* or energy or lamp* or kerosene or fire* or smok* or dung or cook* or chula or chulla or coal or paraffin or charcoal or crop waste* or agricultural residue* or combustion)).tw.
9. or/1-8
10. exp Heart Infarction/
11. coronary thrombosis.mp. or exp Coronary Artery Thrombosis/
12. cardiovascular disease.mp.
13. acute coronary.mp.
14. Myocardial infarct$.mp.
15. heart infarct:.mp.
16. acs.mp.
17. ami.mp.
18. (coronary adj3 syndrome$).mp.
19. brain infarction/ or brain stem infarction/ or cerebellum infarction/ or exp brain ischemia/ or carotid artery disease/ or exp carotid artery obstruction/ or cerebral artery disease/ or exp cerebrovascular accident/ or exp occlusive cerebrovascular disease/ or stroke patient/
20. (isch?emi$ adj5 (stroke$ or apoplex$ or cerebral vasc$ or cerebrovasc$ or cva)).tw.
21. ((brain or cerebr$ or cerebell$ or vertebrobasil$ or hemispher$ or intracran$ or intracerebral or infratentorial or supratentorial or middle cerebr$ or mca$ or anterior circulation or basilar artery or vertebral artery) adj5 (isch?emi$ or infarct$ or thrombo$ or emboli$ or occlus$ or hypoxi$)).tw.

22. Chronic Obstructive Lung Disease/
23. Emphysema/
24. exp Lung Emphysema/
25. Chronic Bronchitis/
26. (obstruct$ adj3 (lung$ or respirat$ or pulmonar$) adj3 disease$).mp.
27. Bronchiti$.mp.
28. emphysema$.mp.
29. (chronic adj5 obstruct$).mp.
30. (pulmonar$ or lung$ or airway$ or airflow$ or bronch$ or respirat$).mp.
31. 29 and 30
32. (COPD or COAD).mp.
33. AECB.mp.
34. exp *asthma/
35. asthma?.ti,ab,kw.
36. exp lung tuberculosis/
37. (tuberculosis or TB).mp.
38. exp Pneumonia/
39. (pneumon* or bronchopneumon* or pleuropneumon*).tw.
40. bronchitis/ or exp bronchiolitis/
41. (bronchiolit* or acute bronchit* or wheez*).tw.
42. Respiratory Syncytial Virus Infections/
43. respiratory syncytial viruses/ or respiratory syncytial virus, human/
44. (respiratory syncytial virus* or rsv).tw.
45. exp Haemophilus influenzae/
46. Streptococcus pneumoniae/
47. Staphylococcus aureus/
48. (streptococc* or staphylococc* or haemophilus influenzae).tw.
49. ((lung or pulmonary) adj2 (inflamm* or infect*)).tw.
50. (low adj3 (birthweight$ or birth weight$)).ti,ab.
51. (lbw or vlbw or elbw).ti,ab.
52. Stillbirth/
53. Fetal death/
54. Foetal death/
55. fetal death$.tw.
56. foetal death$.tw.
57. perinatal death$.tw.
58. intrauterine death$.tw.
59. intrauterine fetal death$.tw.
60. intrauterine foetal death$.tw.
61. perinatal fetal death$.tw.
62. perinatal foetal death$.tw.
63. pregnancy loss.tw.
64. exp mortality/
65. (mortality or mortaliti*).tw,ot.
66. exp lung cancer/
67. (pulmonar$ or lung$ or airway$ or airflow$ or bronch$ or respirat$).mp.
68. (cancer$ or neoplasm$ or tumour$ or tumour$).mp.
69. 67 and 68
70. 10 or 11 or 12 or 13 or 14 or 15 or 16 or 17 or 18 or 19 or 20 or 21 or 22 or 23 or 24 or 25 or 26 or 27 or 28 or 31 or 32 or 33 or 34 or 35 or 36 or 37 or 38 or 39 or 40 or 41 or 42 or 43 or 44 or 45 or 46 or 47 or 48 or 49 or 50 or 51 or 52 or 53 or 54 or 55 or 56 or 57 or 58 or 59 or 60 or 61 or 62 or 63 or 64 or 65 or 66 or 69
71. 9 and 70
72. limit 71 to human

Web of Science (up to 2nd Apr 2020: 13,670 search results)

1. TS=(air pollution)
2. TS=(stove* or cookstove* or biomass or fuel* or biogas* or burn* or ethanol* or gas or electric* or energy or lamp* or kerosene or fire* or smok* or dung or cook* or chula or chulla or coal or paraffin or charcoal or crop waste* or agricultural residue* or combustion or wood or fire* or solar)
3. 1 OR 2
4. TS=(indoor or household or domestic or home)
5. 3 AND 4

6. TS=(Myocardial Infarction or Coronary Thrombosis or acute coronary syndrome or ACS or cerebrovascular disease or stroke or brain infarction or brain ischaemia or brain ischemia or chronic obstructive pulmonary disease or bronchitis or emphysema or COPD or COAD or asthma or tuberculosis or TB or pneumonia or respiratory tract infection or ari or lrti or alri or bronchitis or bronchiolitis or respiratory syncytial virus* or rsv or haemophilus influenzae or streptococcus pneumoniae or staphylococcus aureus or lung cancer* or lung neoplasm* or lung tumor* or lung tumour* or low birth weight or still birth or fetal death or foetal death or perinatal death or intrauterine death or pregnancy loss or mortality or mortalit*)
7. 5 AND 6

Global Health (up to 2nd Apr 2020: 4,599 search results)

1. exp indoor air pollution/
2. ((household or indoor*) adj2 air adj2 (pollut* or quality)).tw.
3. ((indoor or household or domestic or home) adj3 (fire or fires or smok* or woodsmok*)).tw.
4. (time* adj5 (stove or cook*)).tw.
5. ((combust* or burn*) adj3 (stove* or wood or kerosene or biomass or coal or charcoal or dung or crop waste* or agricultur* residu*)).tw.
6. (stove* or cookstove* or kitchen ventilat* or combustion or house construction or solar).tw.
7. (vent* adj5 (home* or living space* or household* or kitchen* or cook* or stove* or window* or hood* or grate*)).tw.
8. ((improv* or replac* or chang* or reduc*) adj5 (fuel* or biogas* or burn* or ethanol* or gas or electric* or energy or lamp* or kerosene or fire* or smok* or dung or cook* or chula or chulla or coal or paraffin or charcoal or crop waste* or agricultural residue* or combustion)).tw.
9. or/1-8
10. exp Myocardial Infarction/
11. Cardiovascular Disease.mp.
12. acute coronary.mp.
13. Myocardial infarct$.mp.
14. heart infarct.mp.
15. acs.mp.
16. ami.mp.
17. (coronary adj3 syndrome$).mp.
18. cerebrovascular disorders/ or basal ganglia cerebrovascular disease/ or brain ischemia/ or exp brain infarction/ or hypoxia-ischemia, brain/ or carotid artery diseases/ or carotid artery thrombosis/ or carotid artery, internal, dissection/ or intracranial arterial diseases/ or cerebral arterial diseases/ or infarction, anterior cerebral artery/ or infarction, middle cerebral artery/ or infarction, posterior cerebral artery/ or exp "intracranial embolism and thrombosis"/ or exp stroke/ or vertebral artery dissection/
19. (isch?emi$ adj5 (stroke$ or apoplex$ or cerebral vasc$ or cerebrovasc$ or cva)).tw.
20. ((brain or cerebr$ or cerebell$ or vertebrobasil$ or hemispher$ or intracran$ or intracerebral or infratentorial or supratentorial or middle cerebr$ or mca$ or anterior circulation or basilar artery or vertebral artery) adj5 (isch?emi$ or infarct$ or thrombo$ or emboli$ or occlus$ or hypoxi$)).tw.
21. exp Chronic Obstructive Pulmonary Disease/
22. (obstruct$ adj3 (lung$ or respirat$ or pulmonar$) adj3 disease$).mp.
23. Bronchiti$.mp.
24. emphysema$.mp.
25. (chronic adj5 obstruct$).mp.
26. (pulmonar$ or lung$ or airway$ or airflow$ or bronch$ or respirat$).mp.
27. 25 and 26
28. (COPD or COAD).mp.
29. AECB.mp.
30. exp asthma/
31. asthma?.mp.
32. exp tuberculosis/
33. (tuberculosis or TB).mp.
34. exp pneumonia/
35. (pneumonia$ or lung inflammation$ or respiratory tract infection$ or respiratory infection$).mp.
36. (acute respiratory infection* or acute respiratory tract infection* or lower respiratory infection* or lower respiratory tract infection*).tw.
37. (ari or lrti or alri).tw.
38. bronchitis/ or exp bronchiolitis/
39. (bronchiolit* or acute bronchit* or wheez*).tw.
40. (respiratory syncytial virus* or rsv).tw.
41. exp Haemophilus influenzae/
42. Streptococcus pneumoniae/

43. Staphylococcus aureus/
44. (streptococc* or staphylococc* or haemophilus influenzae).tw.
45. ((lung or pulmonary) adj2 (inflamm* or infect*)).tw.
46. (low adj3 (birthweight$ or birth weight$)).ti,ab.
47. (lbw or vlbw or elbw).ti,ab.
48. Stillbirth/
49. Fetal death/
50. Foetal death/
51. fetal death$.tw.
52. foetal death$.tw.
53. perinatal death$.tw.
54. intrauterine death$.tw.
55. intrauterine fetal death$.tw.
56. intrauterine foetal death$.tw.
57. perinatal fetal death$.tw.
58. perinatal foetal death$.tw.
59. fetal mortality.tw.
60. foetal mortality.tw.
61. intrauterine fetal mortality.tw.
62. intrauterine foetal mortality.tw.
63. perinatal fetal mortality.tw.
64. perinatal foetal mortality.tw.
65. pregnancy loss.tw.
66. exp Mortality/
67. mortality.tw,ot.
68. mortaliti$.tw,ot.
69. exp lung cancer/
70. (pulmonar$ or lung$ or airway$ or airflow$ or bronch$ or respirat$).mp.
71. (cancer$ or neoplasm$ or tumour$ or tumour$).mp.
72. 70 and 71
73. 10 or 11 or 12 or 13 or 14 or 15 or 16 or 17 or 18 or 19 or 20 or 21 or 22 or 23 or 24 or 27 or 28 or 29 or 30 or 31 or 32 or 33 or 34 or 35 or 36 or 37 or 38 or 39 or 40 or 41 or 42 or 43 or 44 or 45 or 46 or 47 or 48 or 49 or 50 or 51 or 52 or 53 or 54 or 55 or 56 or 57 or 58 or 59 or 60 or 61 or 62 or 63 or 64 or 65 or 66 or 67 or 68 or 69 or 72
74. 9 and 73

Supplementary text 2. Risk of bias assessment criteria

Each individual study was assessed for risk of bias in the assessment of endpoints and adjustment for confounders. Studies that used International Classification of Disease (ICD) codes, physician diagnosis or that used all clinical information to define endpoints were deemed at low risk of bias. Studies that used less robust definitions such as those reliant on participant questionnaires alone were considered as high risk of bias. For confounder adjustment, studies that have adjusted for age, sex and two other relevant covariates were deemed to be at low risk of bias. Studies that have only adjusted for age or sex were considered at moderate risk of bias. Studies that have not adjusted for age, sex or smoking were classified as high risk of bias.

Supplementary text 3. Method for calculating burden and mortality attributable to household air pollution

Step 1 We performed a systematic literature review and meta-analysis to calculate the pooled risk ratio for each individual cardiorespiratory, pediatric and maternal outcome associated with household air pollution (HAP). Where the risk estimates and confidence intervals were reported within the articles, we have not calculated confidence intervals but have instead used those reported within the article. However, where risk ratios were not reported but a 2X2 table was reported, the risk ratios and confidence intervals were calculated as follows:

	**Event**	**No event**
**Exposed**	A	B
**Not exposed**	C	D

Risk ratio (RR) = $\frac{AD}{BC}$

The meta-analyses were performed using log units.

lnRR=ln(RR)

with the approximate variance (V) and standard error (SE) calculated as follows

$$V_{lnRR} = \frac{1}{A} + \frac{1}{B} + \frac{1}{C} + \frac{1}{D}$$

and

$$SE_{lnRR} = \sqrt{V_{lnRR}}$$

We anticipated heterogeneity between studies when estimating the risk ratio due to different study designs, methods of analysis and varying adjustment, and geographical and population differences. We therefore used a random effects model to account for both within and between study heterogeneity.

Step 2 At a study level, we explored interaction between age and cause-specific risk of household air pollution in adults ***(Supplementary figures 1-5)***.

We further evaluated sex interaction in studies that provided risk ratios stratified by sex (11 studies for cardiovascular disease and 27 studies for respiratory disease). For cardiovascular disease, no significant interaction was observed (1.00, 95%CI 0.92-1.09). For respiratory disease, we observed a weak interaction (1.16, 95% CI 1.01-1.33).

Given the absence / weak interaction of age/sex and risk of adult cardiorespiratory disease associated with indoor air pollution we did not compute age- and sex- stratified cause specific risk ratios.

Step 3 We obtained annual prevalence estimates of polluting fuel use in 183 countries from 2000 to 2017 from the World Health Organization (WHO). This was derived by WHO through multilevel modelling based on national survey data on primary cooking fuel. For each country, we obtained WHO derived unpublished prevalence estimates with uncertainty intervals stratified by year.

Similarly, we obtained annual cause and year specific burden (DALYs) and mortality estimates from the Institute of Health Metrics and Evaluation (IHME) from 2000 to 2017 across these countries.[1]

Step 4 Assumptions

- We assume that the pooled risk ratios obtained from a range of case-control/cohort/cross-sectional studies were applicable across both developed and developing countries.
- The prevalence data derived from WHO at national levels were not stratified by age but applicable to the entire population. As such when calculating the population attributable fraction (see below) we applied these prevalence estimates to the entire population.
- We were limited to those confounders included in the primary studies, and while most studies adjusted for patient demographics and some risk factors / mediators including social deprivation there is the potential for residual confounding.

Step 5 We used a simulation based approach to estimate the cause specific population attributable fraction for household air pollution.

The cause-specific risk estimates from random effects meta-analysis were represented as log normal distributions, from which 10,000 samples were obtained using R. Likewise, we represented the prevalence of HAP as a beta distribution and obtained 10,000 samples for each country. We subsequently performed the following calculation for each of the 10,000 samples to derive the cause specific population attributable fraction:

$$Population\ Attributable\ Fraction\ (PAF) = \frac{Prevalence * \ (RR - 1)}{1 + Prevalence * (RR - 1)}$$

We report these uncertainty estimates as uncertainty range (UR). The uncertainty ranges can be viewed as approximate Bayesian posterior probability limits with very diffuse priors for the prevalence estimates and risk-ratios.

Step 6 In a similar manner as Step 5, we represented the cause specific disability-adjusted life years (DALYs) and mortality for each country as log normal distributions and obtained 10,000 samples. For each of the 10,000 samples, we applied the cause specific population attributable fraction calculated in Step 5 to the cause specific DALYs and mortality for each country to estimate the burden and mortality attributable to HAP using the following formula:

$$DALYs\ or\ mortality\ attributable\ to\ HAP = \text{PAF} \times\ \text{DALYs or mortality}$$

Step 7 Steps 5-6 were repeated for each exposure-outcome pair to calculate the burden of disease and mortality for each country. Burden estimates were calculated using outcome-specific risk ratios whilst mortality estimates were calculated using cause-specific mortality risk ratios. We classified each country into WHO regions and 2018 World Bank income groups. Burden and mortality estimate for each WHO region and income group were calculated by summing the country level estimates within each group.

Illustration for deriving country-specific PAF:

Deriving the population attributable fraction of COPD attributable to household air pollution in India in 2017

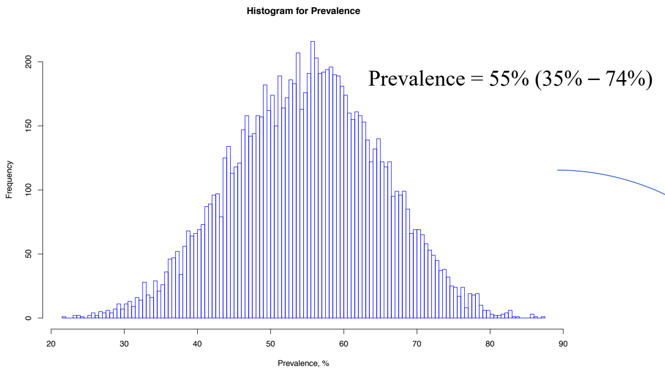

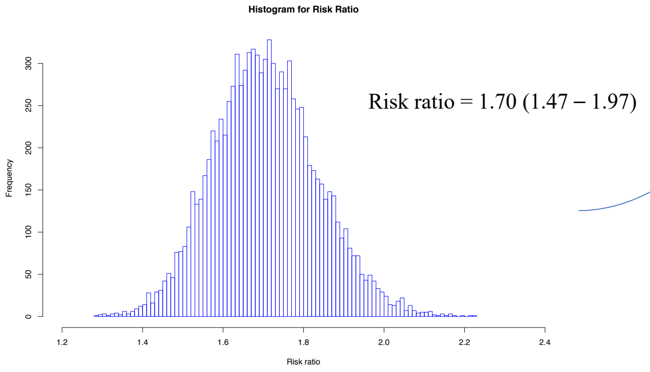

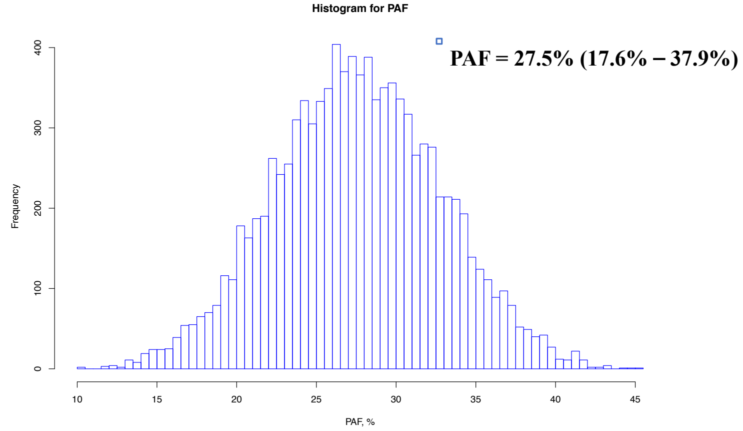

$$Population\ Attributable\ Fraction\ (PAF) = \frac{Prevalence * \ (RR - 1)}{1 + Prevalence * (RR - 1)}$$

Illustration for deriving country-specific burden:

Deriving the burden of COPD attributable to household air pollution in India in 2017

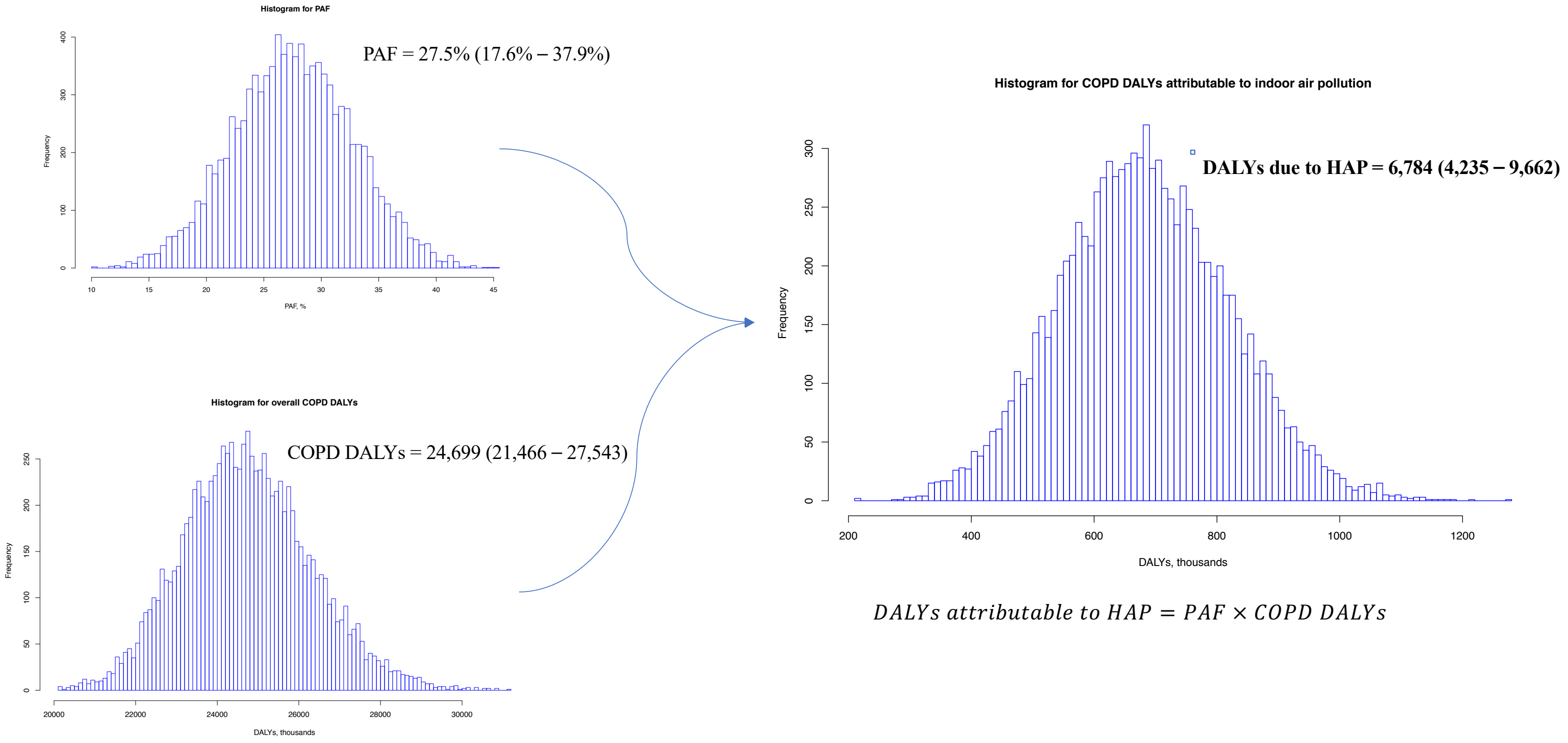

$$DALYs\ attributable\ to\ HAP = PAF \times COPD\ DALYs$$

Figure S1: Normal probability plots of the risk ratios for cardiorespiratory and adverse pregnancy outcomes associated with use of polluting fuels and technologies

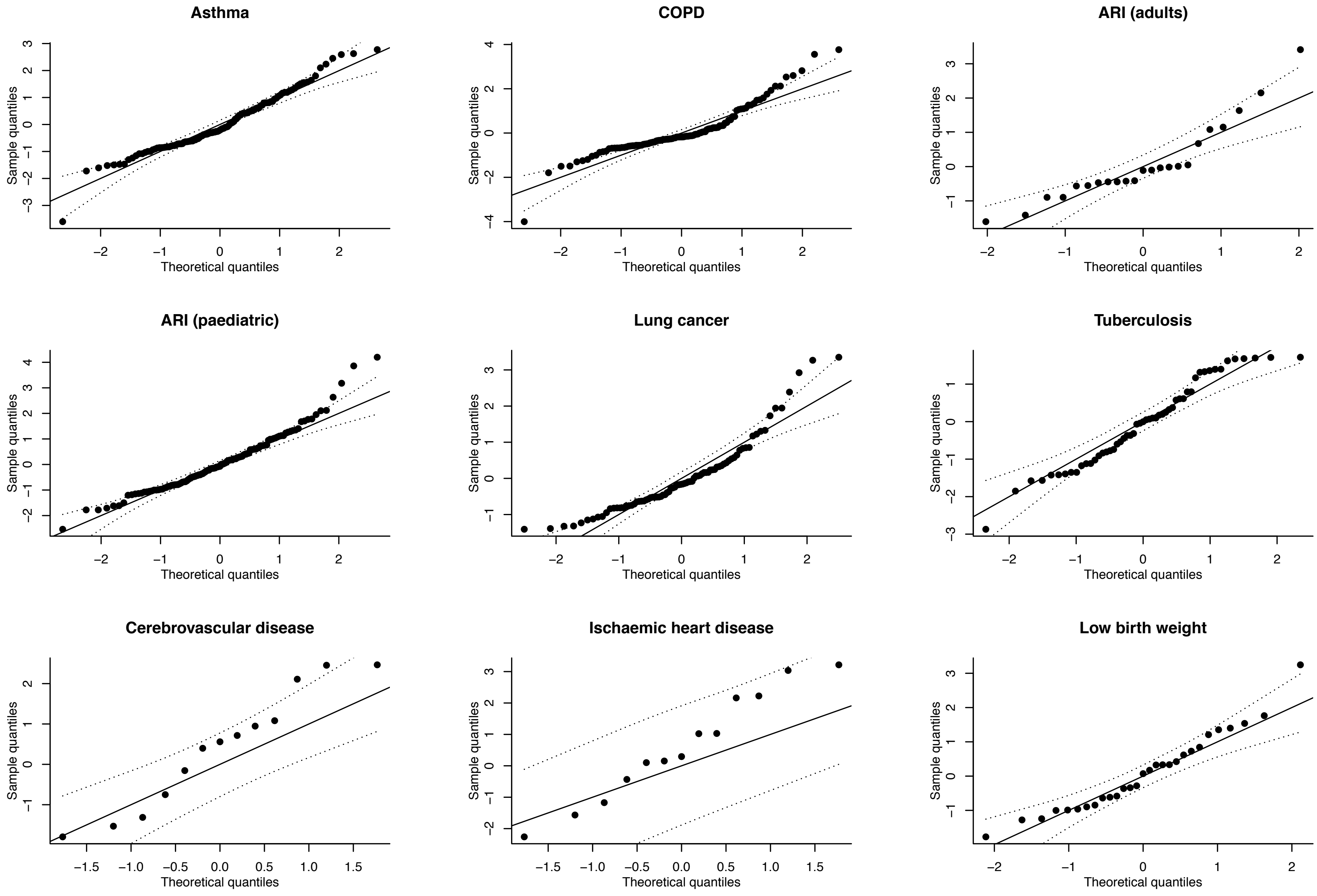

Figure S2: Normal probability plots of the risk ratios for mortality associated with use of polluting fuels and technologies

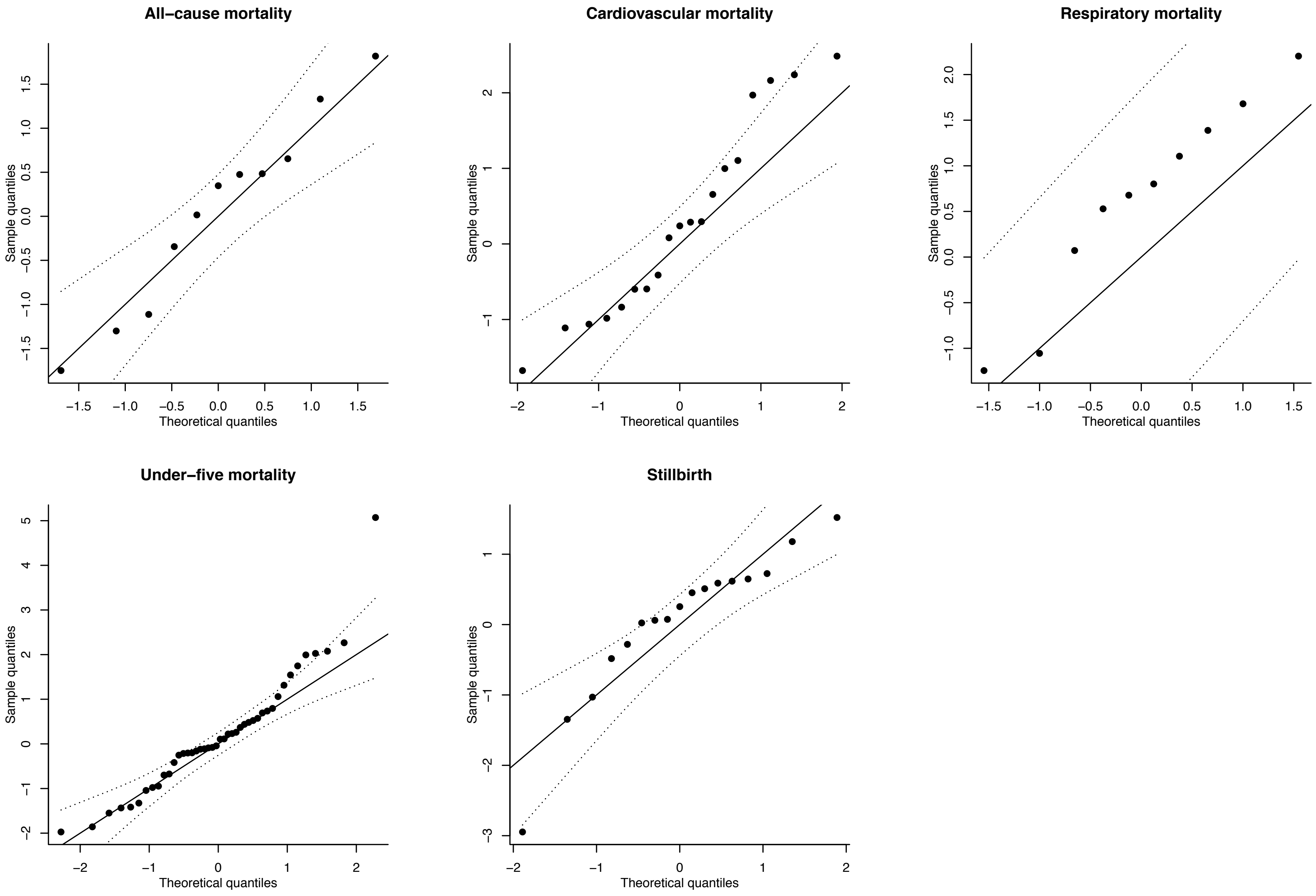

Figure S3: Meta-regression for COPD by mean age
(P value for interaction = 0.910)
*size of each dot represents the weight of each risk estimate using the inverse-variance method.

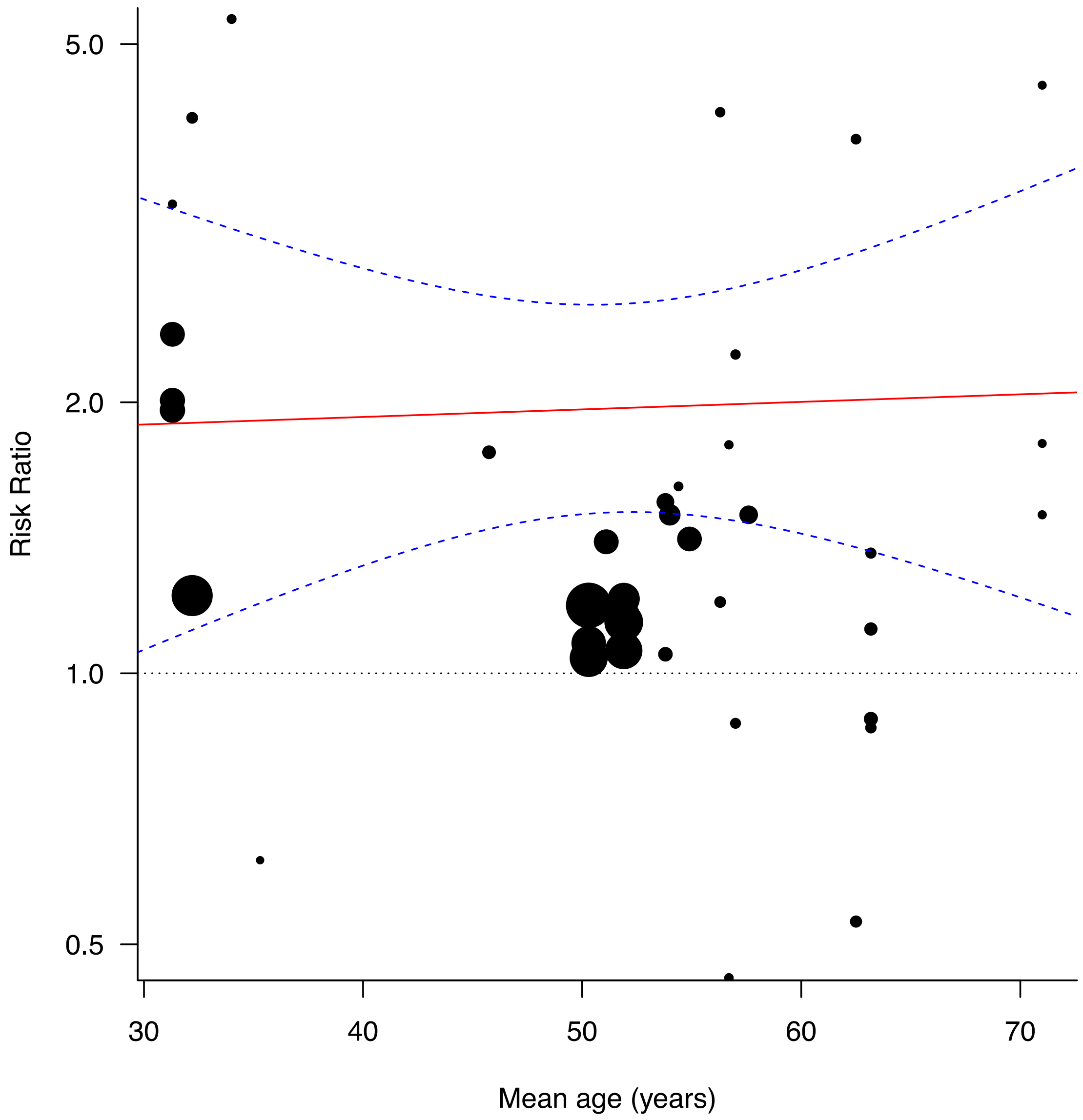

Figure S4: Meta-regression for Asthma by mean age
(P value for interaction = 0.537)
*size of each dot represents the weight of each risk estimate using the inverse-variance method.

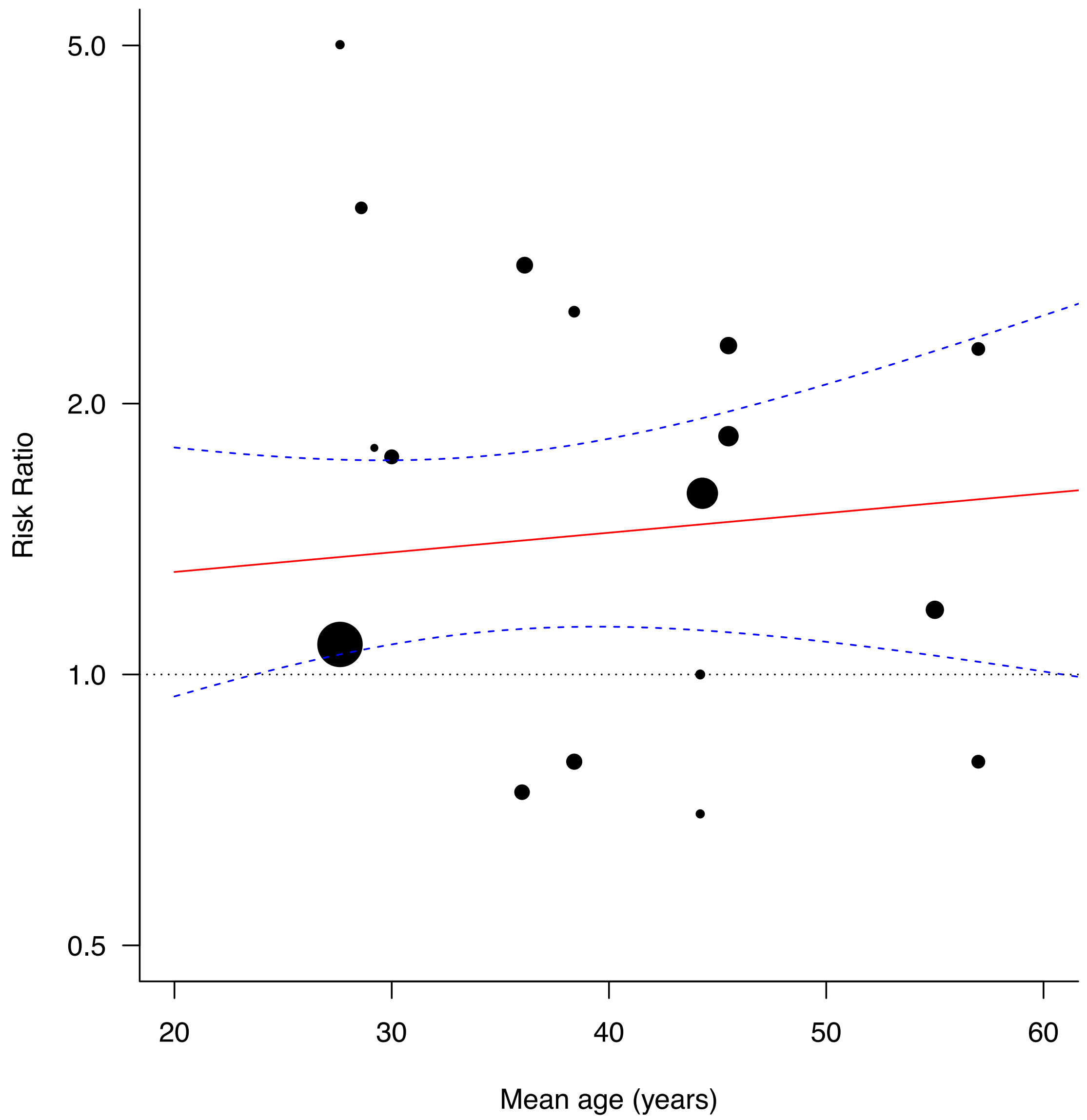

Figure S5: Meta-regression for Tuberculosis by mean age
(P value for interaction = 0.289)
*size of each dot represents the weight of each risk estimate using the inverse-variance method.

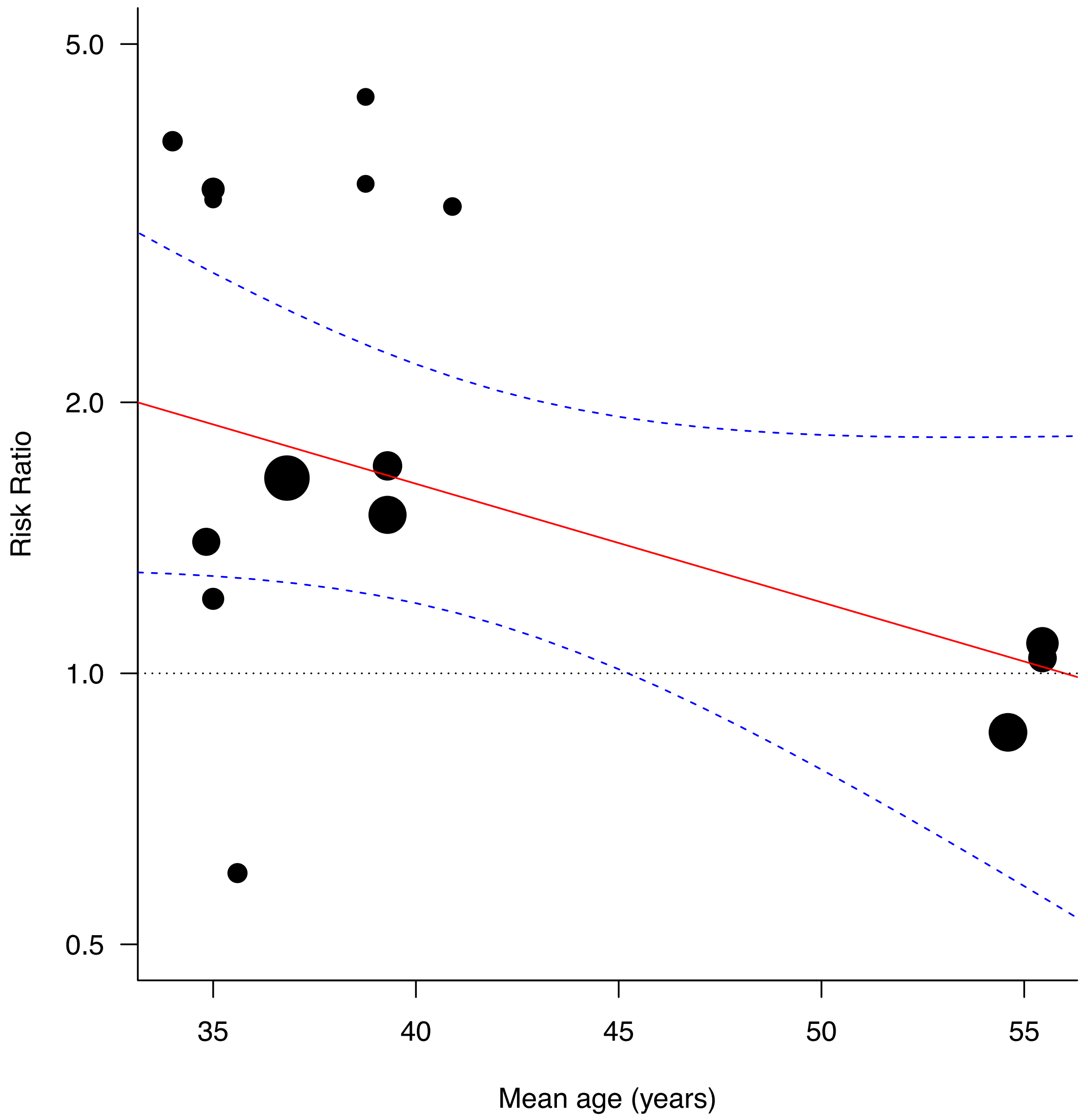

Figure S6: Meta-regression for lung cancer by mean age
(P value for interaction = 0.666)
*size of each dot represents the weight of each risk estimate using the inverse-variance method.

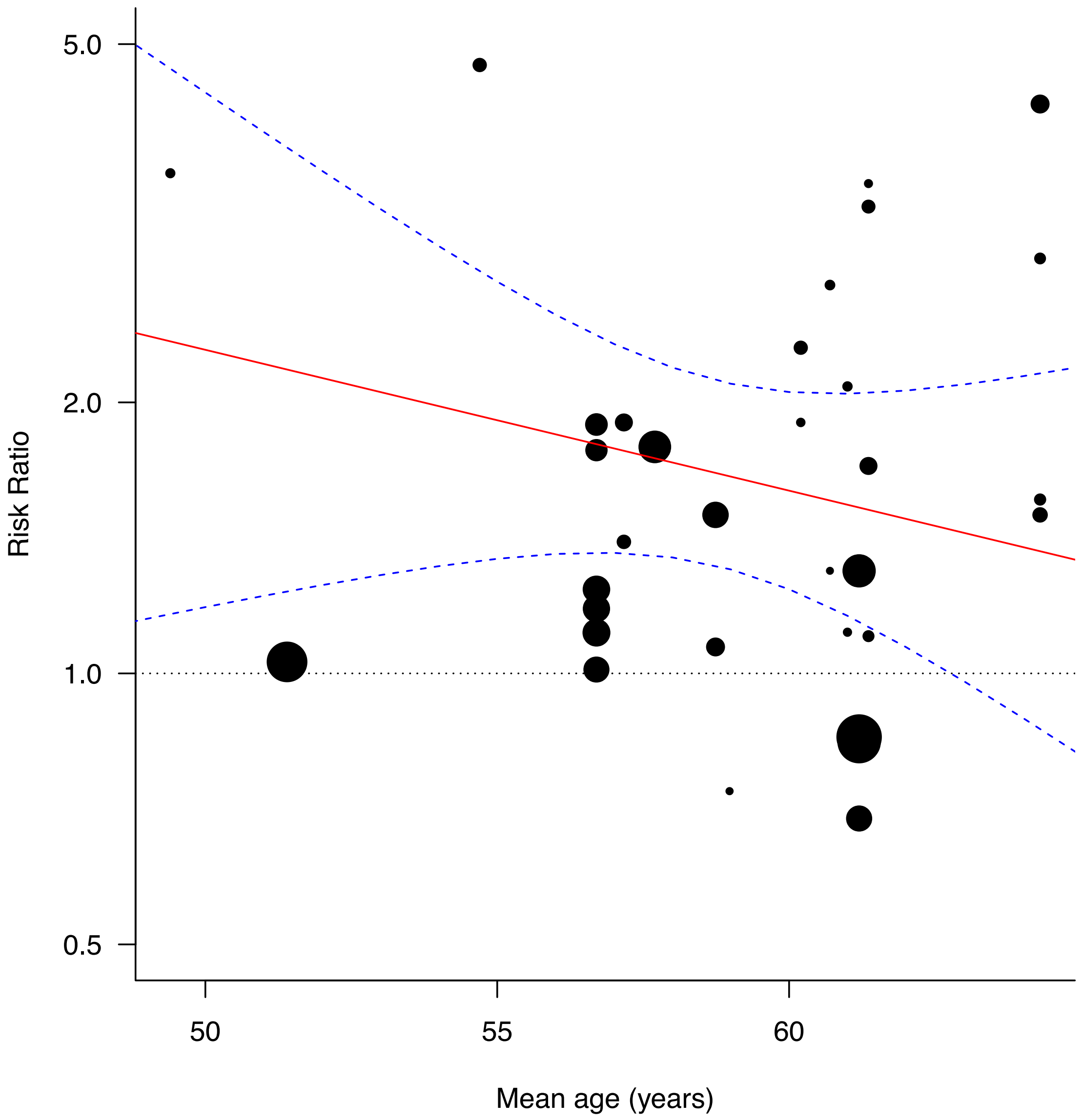

Figure S7: Meta-regression for cardiovascular disease by mean age
(P value for interaction = 0.844)
*size of each dot represents the weight of each risk estimate using the inverse-variance method.

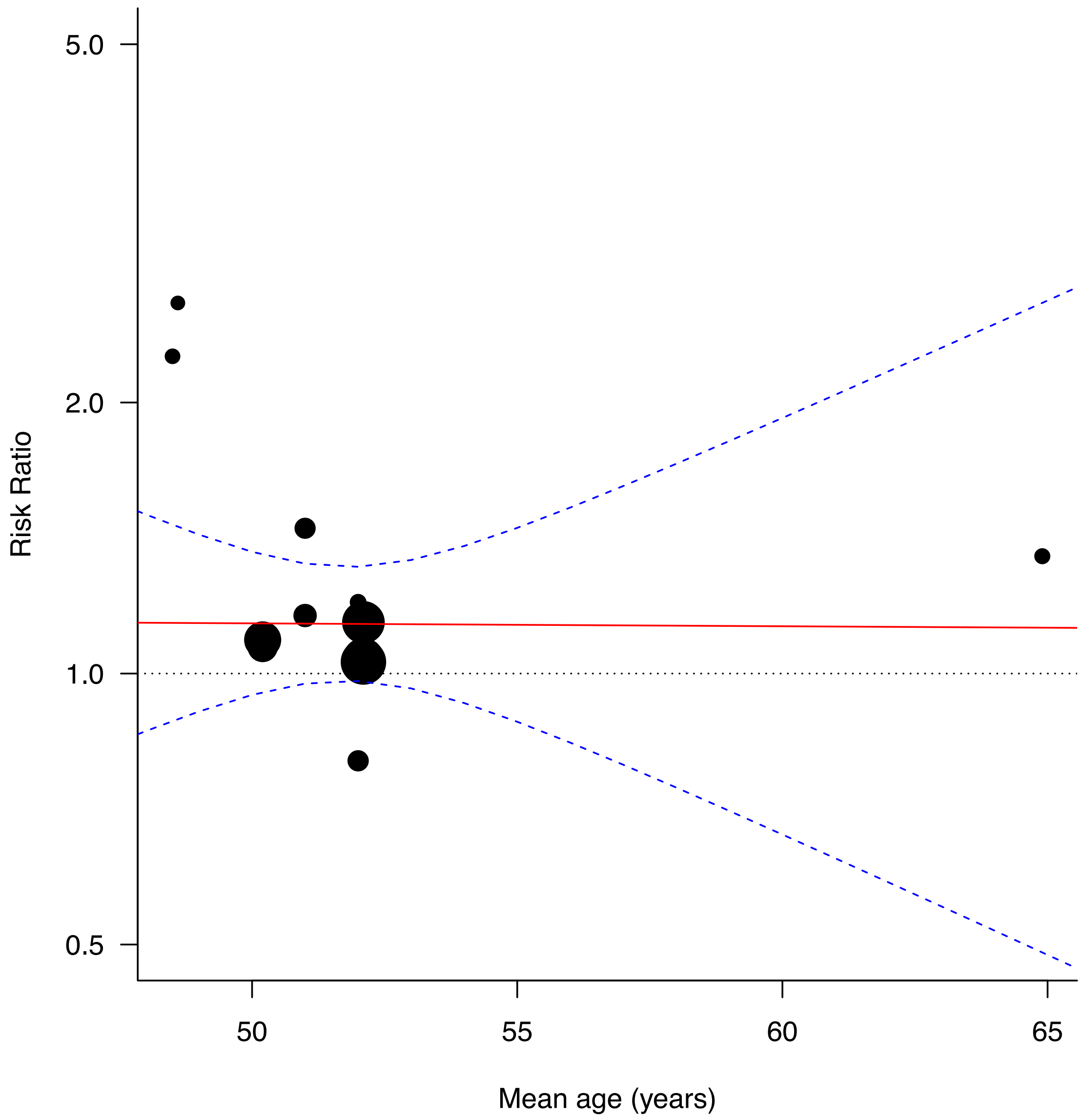

Figure S8: Pooled risk ratios for cardiorespiratory conditions associated with use of polluting fuels and technologies stratified by gender

Disease	No. of estimates	I^2	Pooled Risk Ratios [95% CI]
Asthma	4	56	1.22 (1.02-1.45)
	13	47	1.39 (1.13-1.7)
COPD	11	0	1.19 (1-1.42)
	43	91	2.48 (1.75-3.5)
Ischemic heart disease	5	0.9	1.06 (1.01-1.11)
	7	94	1.36 (1.01-1.82)
Lung cancer	12	88.6	1.45 (1.02-2.06)
	47	81.5	1.41 (1.23-1.63)
Cerebrovascular disease	6	0	1.04 (0.98-1.12)
	6	0	1.03 (0.97-1.1)

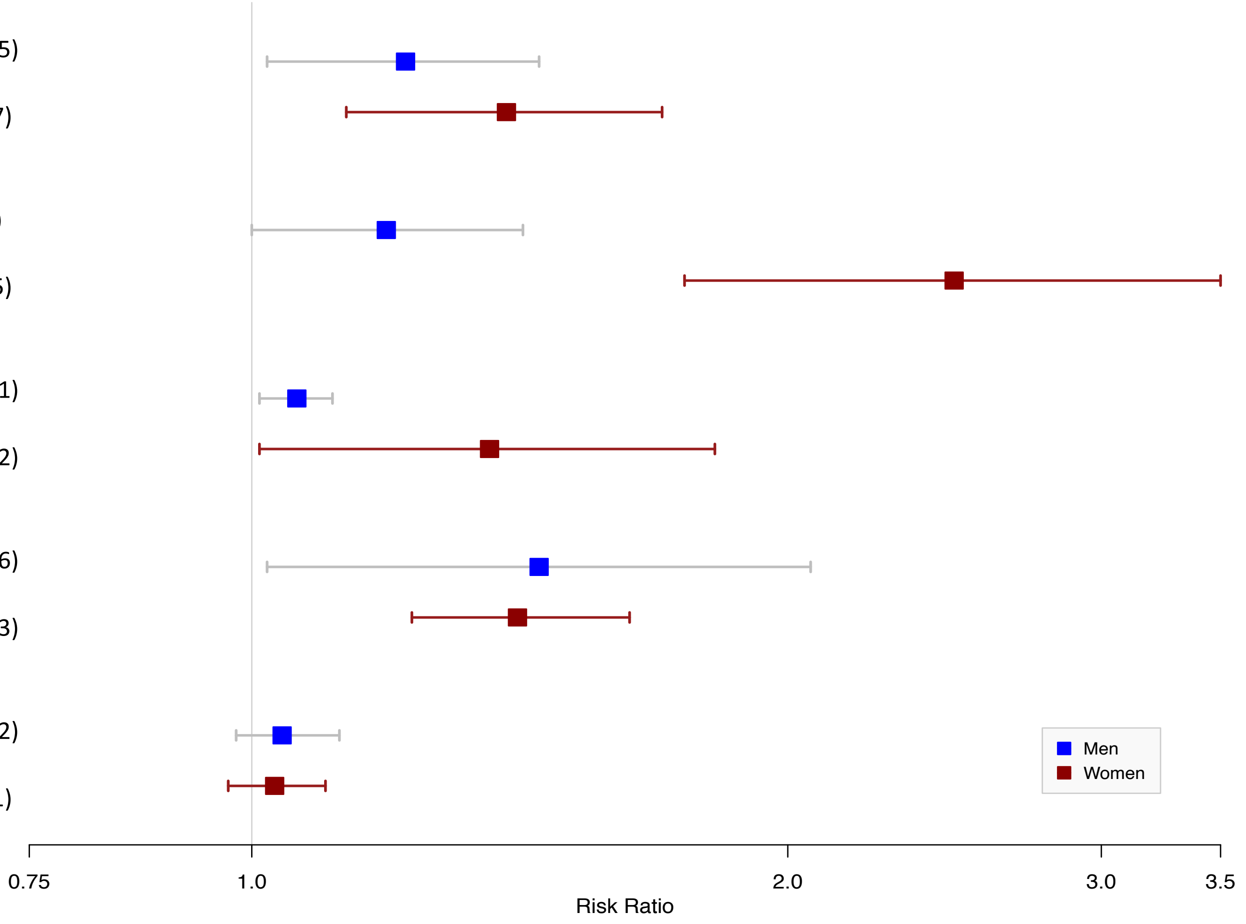

Figure S9: Flow diagram of study selection

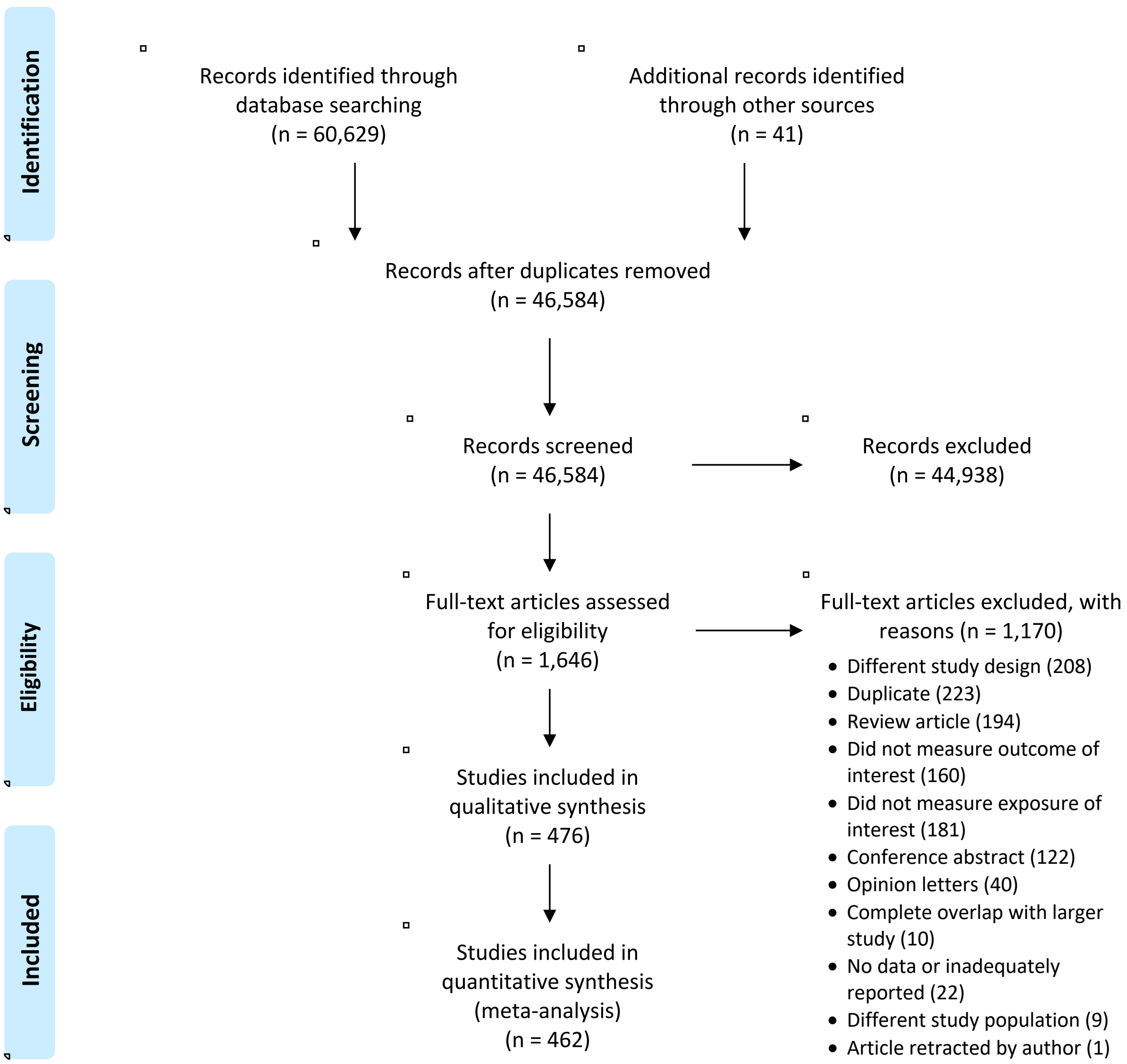

Figure S10: Pooled risk ratio for asthma associated with use of polluting fuels and technologies

Author(s)	Year	Number of events in exposed vs unexposed	Risk Ratio [95% CI]
Azizi et al.1	1991	9/87 vs 19/988	0.50 [0.20-1.00]
Azizi et al.2	1991	18/426 vs 19/988	1.30 [0.90-1.70]
Kuhr et al	1991	not reported	4.79 [1.95-11.80]
Infante-Rivard et al.1	1993	9/22 vs 448/892	0.67 [0.27-1.64]
Infante-Rivard et al.2	1993	98/209 vs 359/705	0.82 [0.58-1.17]
Infante-Rivard et al.3	1993	76/157 vs 381/757	0.91 [0.62-1.32]
Xu et al.1	1993	not reported	0.80 [0.30-2.10]
Fagbule et al	1994	99/212 vs 41/68	0.15 [0.07-0.30]
Ostro et al	1994	not reported	1.59 [1.28-1.97]
Azizi et al.3	1995	18/44 vs 140/315	0.90 [0.50-1.60]
Azizi et al.4	1995	10/19 vs 148/340	1.40 [0.60-3.60]
Volkmer et al	1995	not reported	0.84 [0.76-0.94]
Goren et al	1995	364/3371 vs 231/2242	1.05 [0.89-1.25]
Noorhassim et al	1995	9/208 vs 54/799	0.62 [0.30-1.29]
von Mutius et al	1996	30/622 vs 47/610	0.79 [0.47-1.32]
Xu et al.2	1996	not reported	1.51 [1.05-2.17]
Maier et al	1997	not reported	0.90 [0.60-1.40]
Qian et al	2000	not reported	0.78 [0.36-1.70]
Addo-Yobo et al	2001	34/59 vs 7/22	2.94 [1.03-8.33]
Kilpelainen et al	2001	28/700 vs 356/7810	0.99 [0.65-1.53]
Melsom et al	2001	44/75 vs 65/145	2.20 [1.00-4.50]
Pokharel et al	2001	39/113 vs 1/7	3.16 [0.47-24.70]
von Maffei et al.1	2001	not reported	0.81 [0.58-1.12]
von Maffei et al.2	2001	not reported	1.06 [0.81-1.40]
von Maffei et al.3	2001	not reported	0.78 [0.46-1.33]
von Maffei et al.4	2001	not reported	5.07 [3.24-7.94]
Eisner et al.1	2002	not reported	1.00 [0.30-3.00]
Eisner et al.2	2002	not reported	0.70 [0.20-2.50]
Golshan et al.1	2002	not reported	5.01 [1.45-17.32]
Golshan et al.2	2002	not reported	1.08 [1.01-1.27]
McConnell et al.1	2002	not reported	0.90 [0.60-1.50]
McConnell et al.2	2002	not reported	0.90 [0.60-1.30]
Pan et al	2002	not reported	2.85 [1.61-5.03]
Zheng et al	2002	72/162 vs 23/67	1.60 [0.80-2.80]
Bothwell et al	2003	not reported	1.81 [1.40-2.40]
Mishra et al	2003	not reported	1.59 [1.30-1.94]
Zejda et al	2003	not reported	3.33 [1.26-8.16]
Phoa et al	2004	78/242 vs 138/409	0.91 [0.72-1.14]
Qian et al.3	2004	not reported	1.52 [1.06-2.15]
Qian et al.4	2004	not reported	1.10 [0.84-1.43]
Behrens et al.1	2005	not reported	1.11 [0.16-7.57]
Behrens et al.2	2005	not reported	1.28 [0.85-1.93]
Behrens et al.3	2005	not reported	0.89 [0.40-2.00]
Behrens et al.4	2005	not reported	2.40 [0.35-16.42]
Behrens et al.5	2005	not reported	0.80 [0.43-1.45]
Behrens et al.6	2005	not reported	1.57 [0.69-3.58]
Peabody et al	2005	not reported	1.79 [0.40-7.69]
Siddiqui et al	2005	23/57 vs 11/65	3.30 [1.50-8.10]
Aggarwal et al.1	2006	not reported	0.87 [0.62-1.21]
Aggarwal et al.2	2006	not reported	1.03 [0.84-1.28]
Kurt et al.2	2007	not reported	1.49 [1.27-1.79]
Qian et al.1	2007	not reported	0.80 [0.41-1.37]
Qian et al.2	2007	not reported	2.53 [1.02-6.61]
Dong et al.1	2008	not reported	0.54 [0.21-1.39]
Dong et al.3	2008	not reported	0.70 [0.43-1.15]
Padhi et al	2008	not reported	4.27 [3.00-4.95]
Kumar et al	2008	80/1079 vs 187/2377	0.94 [0.71-1.23]
Ding et al	2009	not reported	1.67 [1.17-5.54]
Kurt et al.1	2009	not reported	1.12 [1.01-1.25]
Barry et al.1	2010	35/142 vs 53/280	2.30 [1.10-5.00]
Barry et al.2	2010	49/239 vs 30/198	0.80 [0.40-1.80]
Kasznia-Kocot et al	2010	not reported	1.91 [1.06-3.45]
Trang et al	2010	156/608 vs 866/2702	0.60 [0.50-0.80]
Vasudeva et al	2010	839/50829 vs 551/34870	1.41 [1.05-1.90]
Firdaus et al	2011	not reported	3.80 [3.41-4.25]
Mustapha et al.1	2011	not reported	0.26 [0.02-4.15]
Mustapha et al.2	2011	not reported	0.13 [0.01-1.78]
Stankovic et al	2011	not reported	0.90 [0.70-1.16]
Agrawal et al.1	2012	1398/69900 vs 501/29670	1.26 [1.06-1.49]
Agrawal et al.2	2012	774/38698 vs 253/18044	0.98 [0.77-1.24]
Ding et al	2012	328/1071 vs 67/251	2.16 [1.70-2.75]
Fedortsiv et al	2012	2/163 vs 81/4709	0.91 [0.22-3.45]
Jindal et al	2012	not reported	1.25 [1.06-1.58]
Pant et al	2012	not reported	1.01 [1.00-1.03]
van Miert et al	2012	31/230 vs 44/514	1.90 [1.10-3.40]
Wolff et al	2012	not reported	2.40 [0.32-17.86]
Alvis-Guzman et al	2013	not reported	0.42 [0.14-1.23]
Dong et al.2	2013	23/2021 vs 195/28759	1.14 [0.64-2.05]
Wang et al	2013	not reported	0.65 [0.29-1.47]
Wong et al.1	2013	not reported	1.45 [1.03-2.03]
Wong et al.2	2013	not reported	1.70 [1.43-2.03]
Zhou et al	2013	not reported	0.97 [0.37-2.50]
Sukhsohale et al.1	2013	10/252 vs 2/192	3.93 [0.85-18.13]
Sukhsohale et al.2	2013	3/73 vs 2/192	4.07 [0.67-24.88]
Sukhsohale et al.3	2013	3/243 vs 2/192	1.19 [0.20-7.18]
Alim et al	2014	13/224 vs 3/196	3.42 [0.50-23.59]
Liu et al.1	2014	152/1226 vs 218/2000	0.96 [0.75-1.23]
Liu et al.2	2014	8/42 vs 218/2000	2.84 [1.24-6.54]
Liu et al.3	2014	not reported	0.79 [0.49-1.30]
Mukherjee et al	2014	26/681 vs 9/438	1.89 [0.88-4.08]
Choi et al	2015	12/161 vs 19/386	0.37 [0.04-3.14]
Gonzalez-Garcia et al	2015	324/3364 vs 177/2175	1.07 [0.87-1.32]
Gaviola et al	2016	30/790 vs 180/2163	1.18 [0.70-1.91]
Patra et al.1	2016	not reported	1.54 [0.62-3.85]
Patra et al.2	2016	not reported	0.84 [0.74-1.25]
Jie et al.1	2016	35/247 vs 12/241	3.15 [1.59-6.23]
Jie et al.2	2016	9/49 vs 12/371	6.73 [2.67-16.96]
Jie et al.3	2016	12/122 vs 12/241	2.08 [0.91-4.78]
Jie et al.4	2016	25/190 vs 12/371	4.53 [2.22-9.24]
Oluwole et al	2017	20/865 vs 14/825	1.13 [0.59-2.16]
Phillips et al	2018	not reported	1.09 [1.03-1.16]
Piekarska et al.1	2018	107/289 vs 669/2217	1.36 [1.05-1.75]
Piekarska et al.2	2018	2075/15122 vs 16/195	0.56 [0.34-0.94]
Piekarska et al.3	2018	207/1347 vs 16/195	0.49 [0.29-0.84]
Piekarska et al.4	2018	713/15121 vs 5/195	0.53 [0.22-1.30]
Piekarska et al.5	2018	48/1779 vs 5/195	2.50 [1.75-3.70]
Svendsen et al.1	2018	6/29 vs 86/644	1.69 [0.67-4.28]
Svendsen et al.2	2018	127/931 vs 86/644	1.02 [0.76-1.38]
Svendsen et al.3	2018	17/170 vs 86/644	0.72 [0.42-1.25]
Panigrahi et al	2018	34/573 vs 12/344	1.75 [0.89-3.42]
Faizan et al.1	2019	not reported	1.17 [1.01-1.35]
Faizan et al.2	2019	not reported	1.13 [0.84-1.52]
Faizan et al.3	2019	not reported	1.05 [0.64-1.71]
Faizan et al.4	2019	not reported	0.77 [0.44-1.34]
Hystad et al	2019	not reported	0.88 [0.69-1.11]
Li et al.1	2019	not reported	1.84 [1.20-2.81]
Li et al.2	2019	not reported	2.32 [1.35-3.98]
Norback et al	2019	not reported	1.09 [0.88-1.35]
Sana et al	2019	28/1015 vs 28/690	0.74 [0.39-1.39]

RE Model (df = 118; I^2 = 94.8%), Pooled risk ratio = 1.23 [95% CI 1.11 - 1.36]

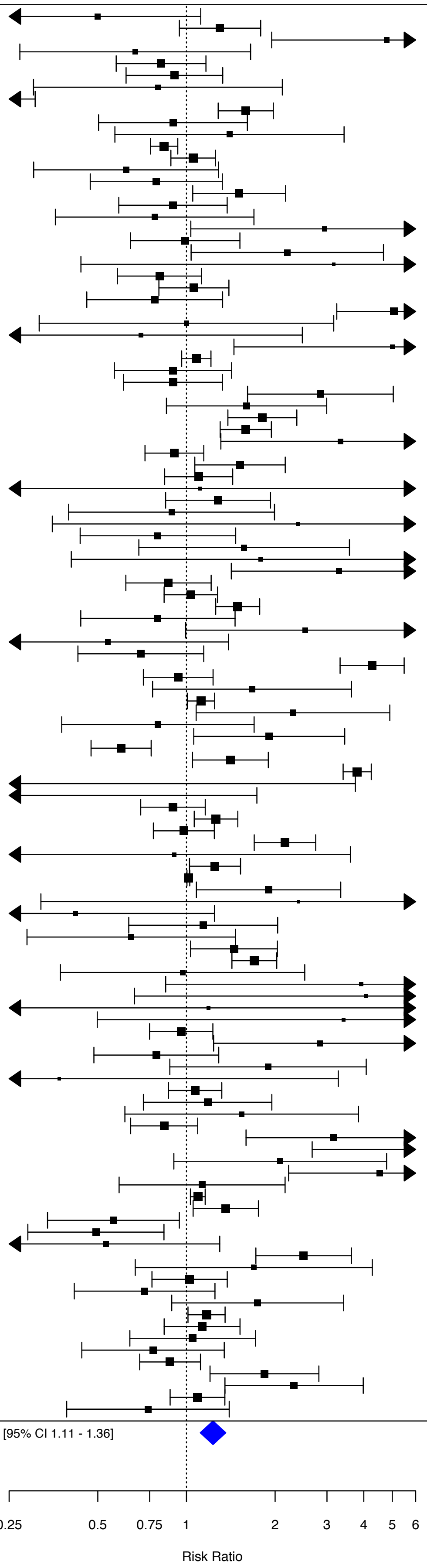

Figure S11: Pooled risk ratio for chronic obstructive pulmonary disease associated with use of polluting fuels and technologies

Author(s)	Year	Number of events in exposed vs unexposed	Risk Ratio [95% CI]
Menezes et al.1	1994	257/1606 vs 411/4419	1.49 [0.92-2.41]
Menezes et al.2	1994	385/2750 vs 411/4419	1.30 [0.75-2.27]
Dossing et al.1	1994	26/37 vs 1/28	63.82 [7.68-529.98]
Dossing et al.2	1994	18/31 vs 5/25	5.54 [1.65-18.61]
Dennis et al.1	1996	88/152 vs 16/56	3.92 [1.70-9.10]
Dennis et al.2	1996	54/124 vs 50/84	0.53 [0.27-1.03]
Perez-Padilla et al.1	1996	48/217 vs 15/221	1.20 [0.60-2.50]
Perez-Padilla et al.2	1996	not reported	9.30 [4.30-20.00]
Perez-Padilla et al.3	1996	not reported	15.00 [5.60-40.00]
Perez-Padilla et al.4	1996	35/107 vs 6/126	4.20 [1.70-10.00]
Perez-Padilla et al.5	1996	not reported	10.30 [3.10-35.00]
Perez-Padilla et al.6	1996	not reported	75.00 [18.00-306.00]
Dutt et al	1996	6/197 vs 1/98	3.05 [0.36-25.67]
Gupta et al	1997	not reported	7.86 [2.84-21.83]
Cetinkaya et al	2000	not reported	1.58 [1.07-2.32]
Golshan et al.1	2002	not reported	1.27 [1.02-1.66]
Golshan et al.2	2002	not reported	2.91 [2.08-4.40]
Kiraz et al	2003	30/242 vs 4/102	1.90 [1.30-3.00]
Ehrlich et al.1	2004	51/1652 vs 81/4018	1.50 [0.90-2.40]
Ehrlich et al.2	2004	109/2752 vs 116/5402	1.50 [1.00-2.10]
Ekici et al	2005	113/397 vs 27/199	1.40 [1.20-1.70]
Peabody et al	2005	not reported	2.33 [0.30-20.00]
Jindal et al.2	2006	44/800 vs 1132/22640	1.25 [0.89-1.76]
Jindal et al.3	2006	117/3773 vs 1132/22640	0.10 [0.79-1.26]
Orozco-Levi et al.1	2006	11/24 vs 9/26	1.80 [0.60-6.00]
Orozco-Levi et al.2	2006	15/35 vs 9/26	1.50 [0.50-4.60]
Orozco-Levi et al.3	2006	25/35 vs 9/26	4.50 [1.40-14.20]
Regalado et al	2006	105/778 vs 6/67	1.50 [0.50-4.30]
Sezer et al.1	2006	9/41 vs 18/40	0.46 [0.16-1.31]
Sezer et al.2	2006	15/27 vs 18/40	1.79 [0.61-5.26]
Sezer et al.3	2006	32/40 vs 18/40	6.61 [2.17-20.18]
Akhtar et al.1	2007	not reported	2.38 [2.12-3.01]
Akhtar et al.2	2007	not reported	2.01 [1.72-2.42]
Akhtar et al.3	2007	not reported	3.32 [1.11-9.88]
Akhtar et al.4	2007	not reported	1.96 [1.75-2.45]
Fei et al.1	2007	47/82 vs 1291/2577	1.32 [0.79-2.20]
Fei et al.2	2007	405/827 vs 1291/2577	0.71 [0.46-1.10]
Fei et al.3	2007	81/144 vs 900/1783	1.18 [0.80-1.76]
Liu et al.1	2007	21/172 vs 132/1808	1.55 [0.74-3.22]
Liu et al.2	2007	157/1306 vs 132/1808	1.72 [1.27-2.35]
Zhong et al	2007	892/9584 vs 776/10661	1.35 [1.20-1.52]
Zhao et al	2009	not reported	36.21 [13.55-96.77]
Zhou et al.2	2009	not reported	0.84 [0.69-1.02]
Zhou et al.3	2009	not reported	1.05 [0.74-1.49]
Zhou et al.4	2009	not reported	1.39 [1.11-1.74]
Zhou et al.5	2009	not reported	2.26 [0.84-6.10]
Zhou et al.6	2009	not reported	1.37 [0.34-5.47]
Zhou et al.7	2009	not reported	2.83 [0.53-3.44]
Zhou et al.8	2009	not reported	1.05 [0.86-1.29]
Zhou et al.9	2009	not reported	1.08 [0.75-1.56]
Zhou et al.10	2009	not reported	1.15 [0.91-1.45]
Zhou et al.11	2009	not reported	1.46 [1.09-1.97]
Zhou et al.12	2009	not reported	1.40 [0.70-2.80]
Zhou et al.13	2009	not reported	1.56 [1.09-2.24]
Sood et al.1	2010	164/515 vs 323/1346	1.96 [1.52-2.52]
Sood et al.2	2010	199/515 vs 373/1346	1.64 [1.31-2.06]
Firdaus et al	2011	not reported	1.12 [1.01-1.25]
Johnson et al	2011	not reported	1.43 [0.36-5.73]
Laniado-Laborin et al	2011	not reported	1.50 [1.20-2.20]
Jindal et al.1	2012	not reported	1.65 [1.35-2.00]
Alvis-Guzman et al	2013	not reported	3.93 [0.33-46.70]
Dong et al	2013	42/2021 vs 327/28759	1.52 [0.98-2.36]
Sukhsohale et al.1	2013	42/252 vs 24/192	1.40 [0.82-2.40]
Sukhsohale et al.2	2013	9/73 vs 24/192	0.98 [0.43-2.23]
Sukhsohale et al.3	2013	20/243 vs 24/192	0.63 [0.34-1.17]
Alim et al	2014	15/224 vs 3/196	5.94 [1.02-34.45]
Dutta et al.1	2014	8/244 vs 3/236	5.33 [1.38-9.60]
Dutta et al.2	2014	6/244 vs 1/236	12.00 [5.42-16.19]
Kurmi et al.1	2014	9/382 vs 13/380	0.62 [0.17-2.31]
Kurmi et al.2	2014	13/463 vs 9/423	0.30 [0.08-1.11]
Yang et al	2014	207/317 vs 1304/2871	2.04 [1.59-2.63]
Zhou et al.1	2014	18/147 vs 20/164	1.61 [0.61-4.35]
Mukherjee et al	2014	31/681 vs 4/438	5.17 [1.81-14.76]
Alam et al	2015	321/1854 vs 164/1659	1.50 [1.20-1.90]
Ding et al	2015	not reported	8.39 [4.91-14.66]
Jaganath et al.1	2015	not reported	1.08 [0.55-2.11]
Jaganath et al.2	2015	not reported	2.22 [1.02-4.81]
Tan et al.1	2015	not reported	0.88 [0.39-1.96]
Tan et al.2	2015	not reported	2.26 [0.93-5.52]
Ngahane et al	2015	11/145 vs 1/155	12.64 [1.61-99.21]
van Gemert et al	2016	not reported	1.76 [1.03-3.00]
de Oca et al.1	2017	not reported	1.27 [0.84-1.93]
de Oca et al.2	2017	not reported	1.34 [0.92-1.96]
Kwas et al	2017	13/81 vs 1/59	11.09 [1.41-87.34]
Chen et al.1	2018	52/126 vs 125/319	0.89 [0.54-1.49]
Chen et al.2	2018	34/77 vs 125/319	1.12 [0.64-1.98]
Chen et al.3	2018	21/95 vs 33/227	0.87 [0.41-1.85]
Chen et al.4	2018	17/60 vs 33/227	1.36 [0.63-2.97]
Siddharthan et al	2018	not reported	1.41 [1.18-1.68]
Musafiri et al.1	2018	not reported	4.14 [2.71-11.18]
Musafiri et al.2	2018	not reported	1.22 [1.13-1.29]
Panigrahi et al	2018	50/573 vs 15/344	1.96 [1.06-3.64]
Phillips et al	2018	not reported	1.14 [1.12-1.16]
Chan et al.1	2019	not reported	1.08 [0.98-1.18]
Chan et al.2	2019	not reported	1.04 [0.96-1.12]
Chan et al.3	2019	not reported	1.19 [1.13-1.26]
Faizan et al.1	2019	not reported	1.37 [1.04-1.79]
Faizan et al.2	2019	not reported	1.37 [0.82-2.28]
Faizan et al.3	2019	not reported	0.49 [0.12-1.97]
Faizan et al.4	2019	not reported	1.70 [0.79-3.67]
Hystad et al	2019	not reported	1.15 [0.91-1.44]
Li et al.1	2019	not reported	1.06 [0.98-1.15]
Li et al.2	2019	not reported	1.14 [1.06-1.23]
Li et al.3	2019	not reported	1.16 [1.04-1.29]
Li et al.4	2019	not reported	1.21 [1.09-1.35]
Woldeamanuel et al	2019	118/602 vs 13/132	2.05 [1.06-3.95]
Zha et al.1	2019	110/961 vs 161/1808	1.05 [0.65-1.69]
Zha et al.2	2019	58/518 vs 213/2251	1.55 [1.11-2.15]

RE Model (df = 107; I^2 = 98.4%), Pooled risk ratio = 1.70 [95% CI 1.47 - 1.97]

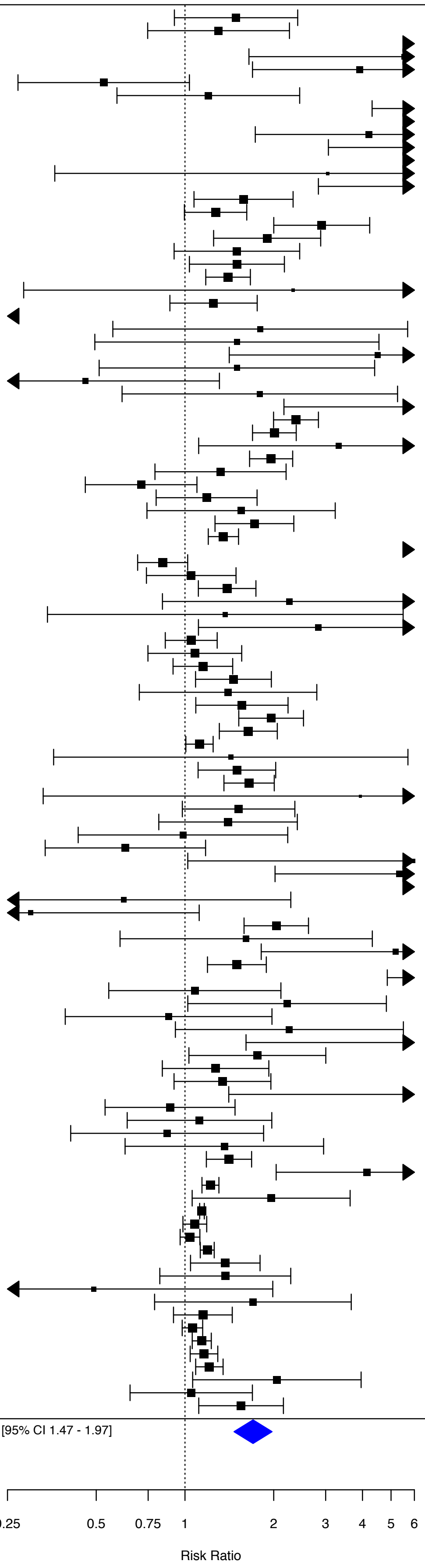

Figure S12: Pooled risk ratio for acute respiratory infection in children associated with use of polluting fuels and technologies

Author(s)	Year	Number of events in exposed vs unexposed	Risk Ratio [95% CI]
Cerqueiro et al	1990	not reported	7.50 [1.80-31.40]
Collings et al	1990	209/576 vs 35/168	2.16 [1.44-3.26]
Shah et al	1994	not reported	0.82 [0.46-1.43]
Victora et al	1994	31/60 vs 479/960	1.10 [0.61-1.98]
Zhou et al	1994	not reported	0.21 [0.06-0.77]
Tumwesigire et al	1995	not reported	0.99 [0.97-1.02]
Goren et al	1995	570/3436 vs 388/2283	0.97 [0.84-1.12]
Dharmage et al	1996	not reported	2.14 [0.87-5.26]
Robin et al.1	1996	13/28 vs 6/9	0.80 [0.30-2.20]
Robin et al.2	1996	28/51 vs 6/9	1.30 [0.50-3.80]
Robin et al.3	1996	14/19 vs 3/7	5.00 [0.60-42.80]
Qian et al	2000	not reported	0.73 [0.56-0.94]
Broor et al	2001	not reported	2.51 [1.51-4.16]
Etiler et al	2002	not reported	1.77 [1.27-2.46]
Mahalanabis et al	2002	97/136 vs 30/126	7.96 [4.39-14.50]
Mishra et al.3	2003	422/2352 vs 84/830	2.20 [1.16-4.19]
Mishra et al.4	2003	57/377 vs 84/830	1.33 [0.64-2.77]
Kumar et al	2004	not reported	3.87 [1.42-10.59]
Wayse et al	2004	32/80 vs 40/70	1.39 [0.61-3.23]
Mishra et al.1	2005	4054/19113 vs 476/3661	1.58 [1.28-1.95]
Mishra et al.2	2005	1287/6995 vs 476/3661	1.41 [1.17-1.70]
Kristensen et al	2006	not reported	1.13 [0.84-1.52]
Wichmann et al	2006	not reported	1.27 [1.05-1.55]
Kilabuko et al	2007	477/4432 vs 73/792	1.01 [0.78-1.42]
Savitha et al	2007	102/159 vs 2/49	42.05 [9.85-179.59]
Johnson et al	2008	not reported	2.92 [1.18-6.87]
Rehfuess et al	2009	141/17491 vs 12/3996	2.48 [1.29-4.77]
Tielsch et al	2009	not reported	1.34 [1.09-1.64]
Kashima et al.1	2010	not reported	0.74 [0.59-0.92]
Kashima et al.2	2010	not reported	0.93 [0.67-1.30]
Kashima et al.3	2010	not reported	1.08 [0.94-1.24]
Kashima et al.4	2010	not reported	1.09 [0.90-1.34]
Bhat et al	2012	95/133 vs 6/69	4.73 [1.67-13.45]
Bulkow et al	2012	60/125 vs 69/186	2.21 [1.20-4.10]
Janjua et al.1	2012	not reported	1.50 [1.20-2.00]
Janjua et al.2	2012	not reported	2.90 [1.70-5.00]
Mir et al	2012	150/492 vs 202/1152	2.06 [1.62-2.63]
Murray et al	2012	not reported	1.05 [0.87-1.27]
van Miert et al	2012	28/230 vs 80/514	0.70 [0.40-1.10]
Aung et al	2013	73/130 vs 45/228	5.20 [3.20-8.40]
Cardoso et al.1	2013	56/150 vs 62/167	0.79 [0.42-1.50]
Gurley et al	2013	not reported	1.10 [0.74-1.62]
Patel et al.1	2013	not reported	1.39 [1.01-1.92]
Patel et al.2	2013	not reported	1.48 [1.08-2.03]
Patel et al.3	2013	not reported	1.47 [1.22-1.76]
Patel et al.4	2013	not reported	1.54 [1.33-1.77]
Patel et al.5	2013	not reported	1.31 [0.92-1.88]
Patel et al.6	2013	not reported	1.53 [1.21-1.93]
Sharma et al	2013	91/251 vs 44/249	2.84 [1.27-6.42]
Wang et al	2013	not reported	0.97 [0.76-1.25]
Zheng et al	2013	not reported	1.75 [1.16-2.64]
Islam et al	2013	87/326 vs 10/44	1.24 [0.59-2.61]
Alemayehu et al.1	2014	128/179 vs 3/10	3.89 [1.54-28.25]
Alemayehu et al.2	2014	57/106 vs 3/10	1.14 [0.15-8.85]
Budge et al	2014	not reported	1.11 [0.52-2.34]
Fakunle et al	2014	39/44 vs 181/396	2.21 [1.70-7.00]
Ram et al	2014	281/884 vs 50/110	1.75 [1.16-2.63]
Sanbata et al.1	2014	76/254 vs 9/92	2.96 [1.38-3.87]
Sanbata et al.2	2014	16/76 vs 9/92	1.96 [0.78-4.89]
Sanbata et al.3	2014	37/95 vs 10/95	5.42 [2.50-11.76]
Sanbata et al.4	2014	6/34 vs 10/95	1.82 [0.61-5.46]
Acharya et al	2015	199/4061 vs 25/742	1.79 [1.02-3.14]
Buchner et al.1	2015	2746/18991 vs 135/1366	1.23 [0.77-1.95]
Buchner et al.2	2015	30/300 vs 135/1366	1.35 [1.06-1.72]
Buchner et al.3	2015	511/4472 vs 135/1366	1.32 [1.04-1.66]
Buchner et al.4	2015	33/244 vs 135/1366	1.07 [0.69-1.66]
Buchner et al.5	2015	2300/24968 vs 109/1490	1.64 [0.99-2.71]
Buchner et al.6	2015	23/184 vs 109/1490	1.54 [1.21-1.97]
Buchner et al.7	2015	423/4098 vs 109/1490	1.20 [0.95-1.51]
Buchner et al.8	2015	28/324 vs 109/1490	1.49 [0.93-2.35]
Cardoso et al.2	2015	127/4602 vs 34/1475	1.17 [0.78-1.76]
Cardoso et al.3	2015	7/275 vs 109/4644	0.84 [0.33-2.16]
Cardoso et al.4	2015	44/1157 vs 109/4634	1.47 [0.81-2.64]
Harerimana et al	2016	33/743 vs 279/7761	1.12 [0.65-1.96]
Praygod et al.1	2016	5/12 vs 1/4	2.10 [0.20-27.00]
Praygod et al.2	2016	38/99 vs 1/4	1.90 [0.20-18.00]
Ramani et al	2016	not reported	3.29 [2.08-5.19]
Rey-Ares et al.1	2016	not reported	1.12 [0.61-2.05]
Rey-Ares et al.2	2016	not reported	1.87 [0.98-3.55]
Tin et al	2016	not reported	0.79 [0.66-0.93]
Hasan et al.1	2017	59/729 vs 359/7031	1.62 [1.22-2.16]
Hasan et al.2	2017	153/2950 vs 83/2190	1.31 [0.96-1.79]
Hasan et al.3	2017	1083/6602 vs 670/4437	1.01 [0.88-1.16]
Khan et al	2017	2667/18020 vs 530/3681	1.07 [0.95-1.20]
Lamichhane et al	2017	not reported	0.95 [0.94-0.96]
Admasie et al	2018	265/1001 vs 21/143	2.09 [1.29-3.39]
Akinyemi et al.1	2018	not reported	0.99 [0.98-1.00]
Akinyemi et al.2	2018	not reported	1.00 [0.99-1.01]
Akinyemi et al.3	2018	not reported	1.44 [0.66-3.13]
Bates et al.1	2018	9/24 vs 317/679	0.74 [0.31-1.72]
Bates et al.2	2018	67/121 vs 317/679	1.47 [0.99-2.18]
Bates et al.3	2018	104/187 vs 70/181	2.13 [1.39-3.27]
Bates et al.4	2018	109/218 vs 70/181	1.65 [1.10-2.49]
Chen et al	2018	not reported	1.29 [0.72-2.33]
Kafando et al.1	2018	not reported	0.18 [0.01-2.38]
Kafando et al.2	2018	not reported	0.63 [0.10-3.81]
Kafando et al.3	2018	not reported	14.70 [1.16-186.89]
Khan et al	2018	not reported	1.51 [1.03-2.21]
Tazinya et al	2018	198/309 vs 82/203	1.85 [1.22-2.78]
Indahsari et al	2018	18/19 vs 104/137	5.71 [0.73-44.43]
Alemayehu et al.3	2019	45/155 vs 47/133	2.21 [1.12-9.37]
Arlington et al.1	2019	26/143 vs 66/499	1.20 [0.70-2.20]
Arlington et al.2	2019	79/395 vs 66/499	1.40 [1.00-1.90]
Arlington et al.3	2019	68/317 vs 66/499	1.50 [1.10-2.00]
Arlington et al.4	2019	63/232 vs 66/499	2.10 [1.40-3.30]
Ngocho et al	2019	79/268 vs 32/186	1.80 [1.00-3.30]
Patel et al.7	2019	not reported	0.78 [0.75-0.80]
Patel et al.8	2019	not reported	0.94 [0.90-0.98]
Patel et al.9	2019	not reported	1.21 [1.17-1.25]
Patel et al.10	2019	not reported	1.06 [0.96-1.16]
Suryadhi et al.1	2019	not reported	1.00 [0.93-1.09]
Suryadhi et al.2	2019	not reported	1.22 [1.08-1.37]
Anteneh et al.1	2020	not reported	0.80 [0.24-2.64]
Anteneh et al.2	2020	not reported	1.01 [0.70-1.46]
Anteneh et al.3	2020	not reported	1.27 [0.87-1.85]
Anteneh et al.4	2020	not reported	1.90 [1.15-3.15]
Budhathoki et al.1	2020	198/4041 vs 25/721	1.19 [0.72-1.98]
Budhathoki et al.2	2020	89/3375 vs 13/1140	1.98 [1.01-3.92]
Rana et al.1	2020	not reported	1.10 [0.98-1.23]
Rana et al.2	2020	not reported	1.17 [1.03-1.32]
Wu et al.1	2020	not reported	0.56 [0.12-2.56]
Wu et al.2	2020	not reported	0.88 [0.29-2.66]
Wu et al.3	2020	not reported	0.94 [0.73-1.21]

RE Model (df = 122; I^2 = 98.8%), Pooled risk ratio = 1.39 [95% CI 1.29 - 1.49]

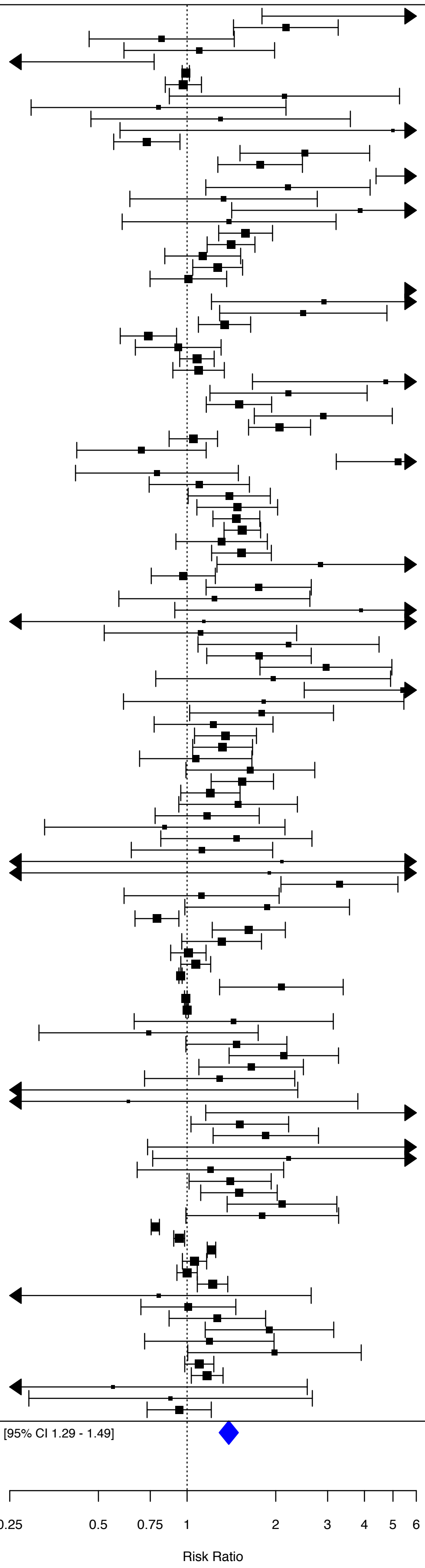

Figure S13: Pooled risk ratio for acute respiratory infection in adults associated with use of polluting fuels and technologies

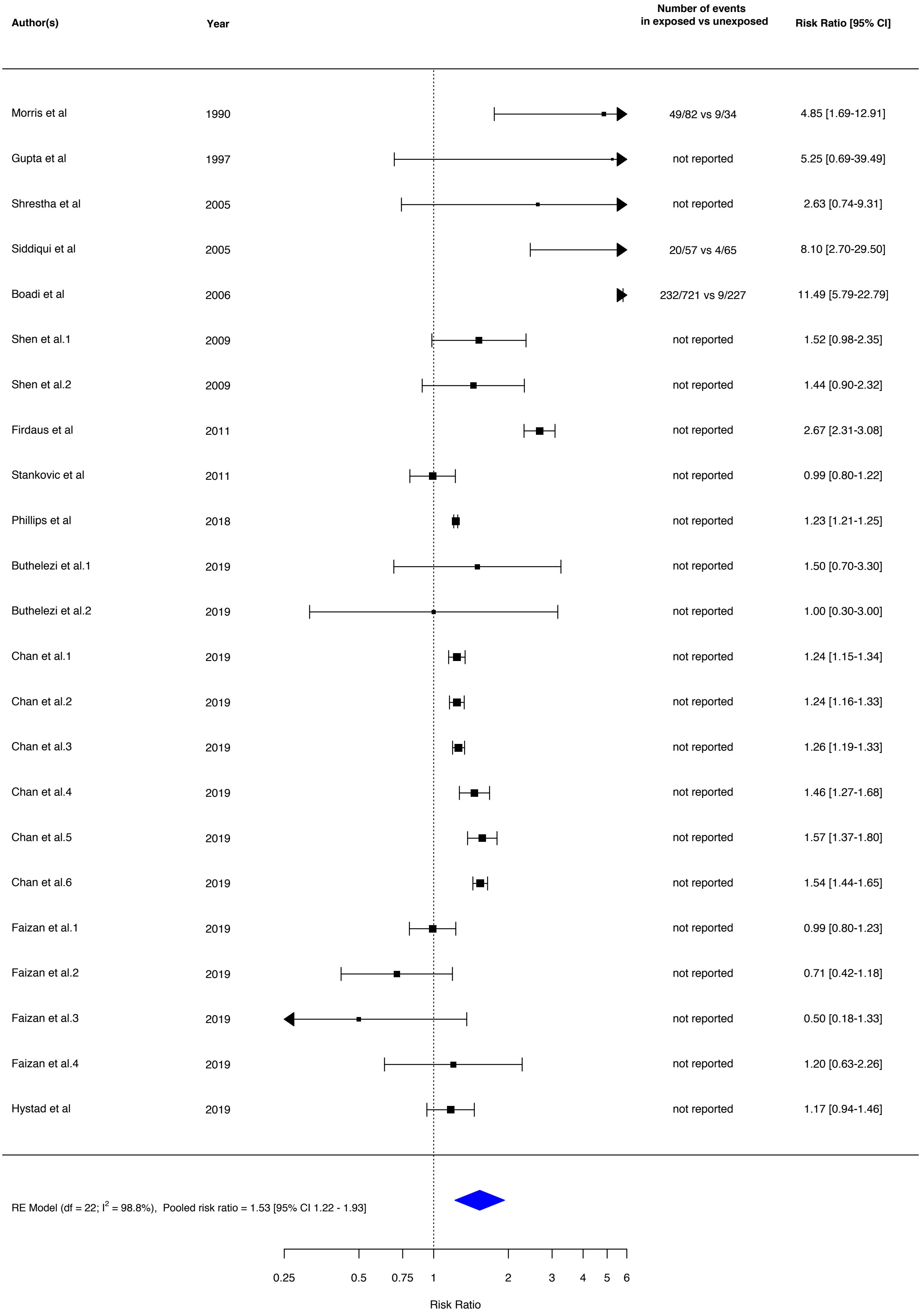

Figure S14: Pooled risk ratio for lung cancer associated with use of polluting fuels and technologies

Author(s)	Year	Number of events in exposed vs unexposed	Risk Ratio [95% CI]
Koo et al.1	1983	not reported	0.69 [0.57-1.27]
Koo et al.2	1983	not reported	0.85 [0.78-1.11]
Koo et al.3	1983	not reported	0.84 [0.77-1.12]
Koo et al.4	1983	not reported	1.30 [0.84-1.49]
Wu et al.1	1985	not reported	2.30 [1.00-5.50]
Wu et al.2	1985	not reported	1.90 [0.50-6.50]
Gao et al.1	1987	not reported	0.90 [0.70-1.30]
Gao et al.2	1987	not reported	1.00 [0.60-1.80]
Sobue et al.1	1990	85/455 vs 59/420	1.24 [0.86-1.81]
Sobue et al.2	1990	32/103 vs 112/772	1.89 [1.16-3.06]
Sobue et al.3	1990	104/634 vs 40/241	1.01 [0.68-1.52]
Sobue et al.4	1990	93/530 vs 51/345	1.18 [0.81-1.72]
Sobue et al.5	1990	67/394 vs 77/481	1.11 [0.77-1.60]
Sobue et al.6	1990	not reported	1.77 [1.08-2.91]
Wu-Williams et al.1	1990	173/295 vs 677/1417	1.50 [1.10-2.00]
Wu-Williams et al.2	1990	173/341 vs 525/1108	1.10 [0.80-1.40]
Wu-Williams et al.3	1990	106/197 vs 677/1417	1.20 [0.90-1.70]
Wu-Williams et al.4	1990	258/460 vs 525/1108	1.20 [1.00-1.60]
Huang et al	1992	not reported	1.59 [1.01-2.50]
Sun et al.4	1992	not reported	2.26 [1.53-3.33]
Wang et al.3	1992	not reported	1.31 [0.73-1.79]
Lan et al	1993	74/96 vs 65/182	7.53 [3.31-17.17]
Liu et al.1	1993	14/36 vs 200/393	2.08 [0.62-6.67]
Liu et al.2	1993	8/17 vs 81/160	1.11 [0.30-4.17]
Dai et al.1	1996	not reported	18.75 [3.94-29.32]
Dai et al.2	1996	not reported	4.46 [1.61-12.33]
Du et al.1	1996	not reported	1.50 [0.69-3.27]
Du et al.2	1996	not reported	4.29 [2.33-7.88]
Du et al.3	1996	not reported	1.56 [0.57-4.25]
Du et al.4	1996	not reported	2.89 [1.09-8.65]
Luo et al.1	1996	not reported	14.10 [11.80-16.10]
Luo et al.2	1996	not reported	6.00 [4.90-7.10]
Wang et al.1	1996	88/135 vs 89/135	0.97 [0.64-1.48]
Wang et al.2	1996	100/135 vs 107/135	2.37 [1.44-3.91]
Ko et al.1	1997	not reported	1.30 [0.30-5.80]
Ko et al.2	1997	not reported	2.70 [0.90-8.90]
Mzileni et al.1	1999	260/418 vs 28/53	1.90 [0.90-3.30]
Mzileni et al.2	1999	51/207 vs 9/50	1.40 [0.60-3.20]
Zhong et al	1999	255/567 vs 153/319	1.11 [0.81-1.52]
Gupta et al.1	2001	58/184 vs 39/102	0.75 [0.43-1.32]
Gupta et al.2	2001	135/377 vs 39/102	0.72 [0.44-1.19]
Gupta et al.3	2001	7/39 vs 4/21	0.73 [0.15-3.45]
Gupta et al.4	2001	19/60 vs 4/21	1.43 [0.33-6.30]
Lee et al.1	2001	14/40 vs 48/141	1.10 [0.40-3.10]
Lee et al.2	2001	49/121 vs 73/239	1.70 [0.90-3.30]
Lee et al.3	2001	22/37 vs 48/141	3.50 [0.90-12.90]
Lee et al.4	2001	40/75 vs 73/239	3.30 [1.40-8.00]
Qin et al	2002	not reported	1.78 [1.33-2.38]
Sasco et al	2002	8/20 vs 110/333	0.74 [0.17-3.14]
Sun et al.1	2002	not reported	2.13 [1.05-4.32]
Sun et al.2	2002	not reported	2.10 [0.58-8.21]
Sun et al.3	2002	not reported	2.12 [1.27-3.56]
Hernandez-Garduno et al.1	2004	15/79 vs 36/128	0.60 [0.30-1.20]
Hernandez-Garduno et al.2	2004	15/70 vs 36/128	0.60 [0.30-1.30]
Hernandez-Garduno et al.3	2004	47/109 vs 36/128	1.90 [1.10-3.50]
Behera et al	2005	not reported	3.59 [1.08-11.97]
Lissowska et al	2005	2379/4909 vs 482/1070	1.22 [1.04-1.44]
Ramanakumar et al.1	2007	330/773 vs 108/254	0.70 [0.50-1.00]
Ramanakumar et al.2	2007	253/475 vs 62/221	2.50 [1.50-3.60]
Galeone et al	2008	202/586 vs 14/65	2.19 [1.08-4.46]
Sapkota et al	2008	not reported	0.98 [0.74-1.31]
Stein et al.1	2008	103/403 vs 230/1309	1.60 [1.20-2.20]
Stein et al.2	2008	30/733 vs 60/2030	1.40 [0.80-2.20]
Hosgood et al	2010	3557/7360 vs 1548/4280	1.56 [1.44-1.69]
Firdaus et al	2011	not reported	1.15 [1.04-1.28]
Tse et al.1	2011	85/148 vs 706/1310	1.15 [0.82-1.63]
Tse et al.2	2011	30/55 vs 706/1310	1.03 [0.60-1.76]
Sloan et al	2012	not reported	1.00 [0.98-1.01]
Mu et al.1	2013	91/146 vs 28/92	4.08 [2.17-7.67]
Mu et al.2	2013	70/141 vs 56/180	2.00 [1.24-3.23]
Mu et al.3	2013	20/32 vs 56/180	3.20 [1.40-7.32]
Kim et al	2014	249/458 vs 11/62	6.31 [2.85-13.94]
Phukan et al.1	2014	25/86 vs 205/604	1.07 [0.58-1.97]
Phukan et al.2	2014	111/254 vs 119/436	1.50 [1.01-2.22]
Yang et al	2014	171/285 vs 1388/2953	1.54 [1.19-2.00]
Kim et al.2	2015	270/44858 vs 159/26462	1.03 [0.83-1.26]
Kim et al.1	2016	not reported	1.20 [0.95-1.52]
Raspanti et al	2016	556/1104 vs 30/68	1.77 [1.00-3.14]
Hystad et al	2019	not reported	0.79 [0.53-1.18]
Wong et al.1	2019	603/1031 vs 89/493	19.64 [9.95-38.77]
Wong et al.2	2019	63/1031 vs 15/493	10.83 [4.61-25.46]
Wong et al.3	2019	33/1031 vs 22/493	4.74 [2.03-11.04]

RE Model (df = 81; I^2 = 96.6%), Pooled risk ratio = 1.69 [95% CI 1.44 - 1.98]

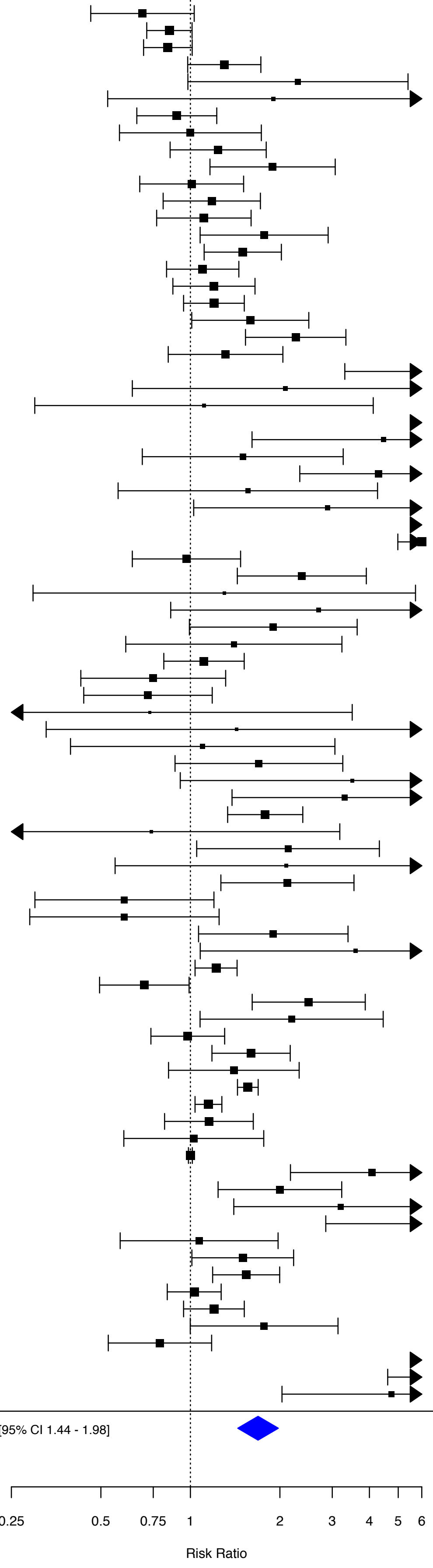

Figure S15: Pooled risk ratio for tuberculosis associated with use of polluting fuels and technologies

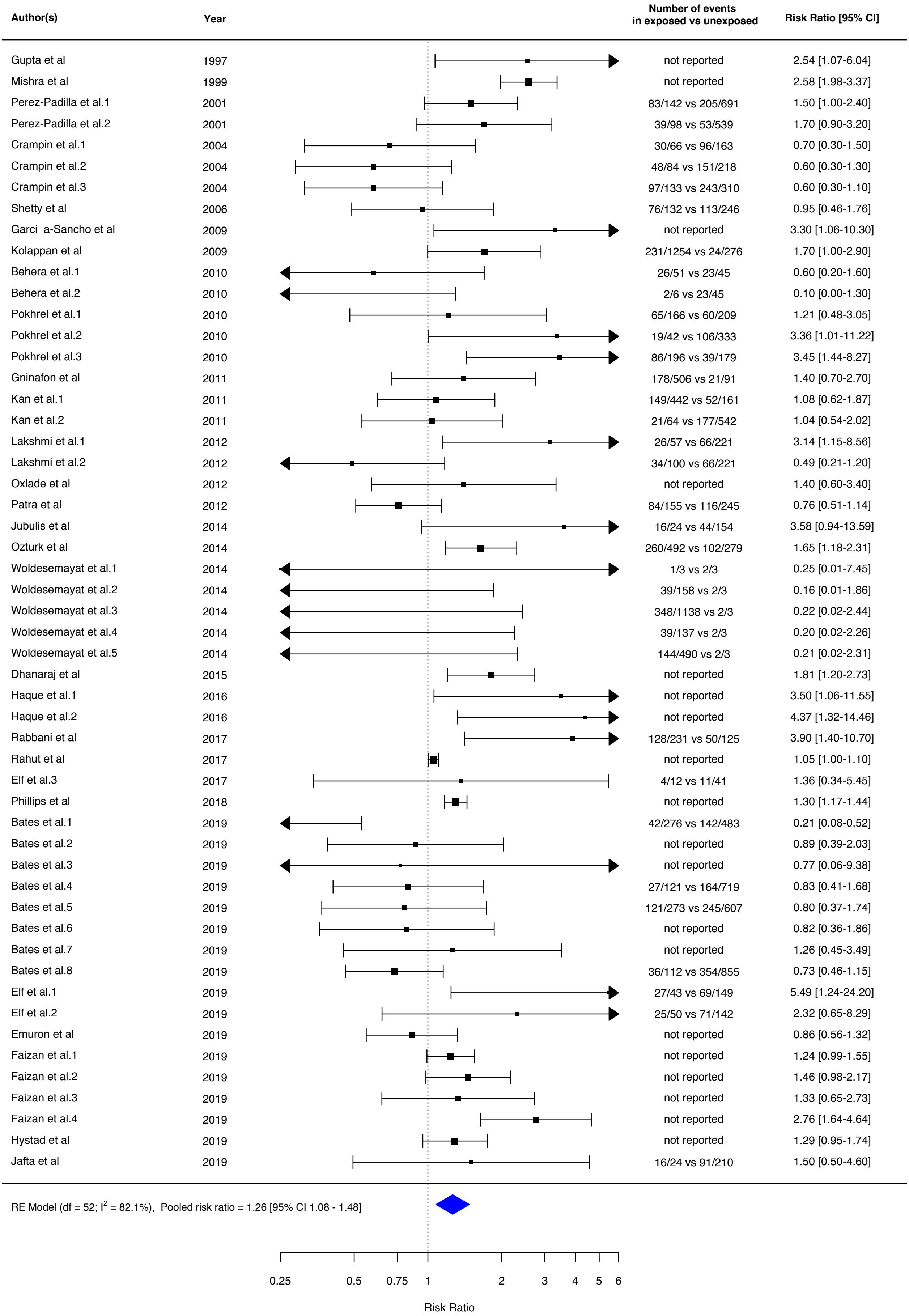

Figure S16: Pooled risk ratio for cardiovascular disease associated with use of polluting fuels and technologies

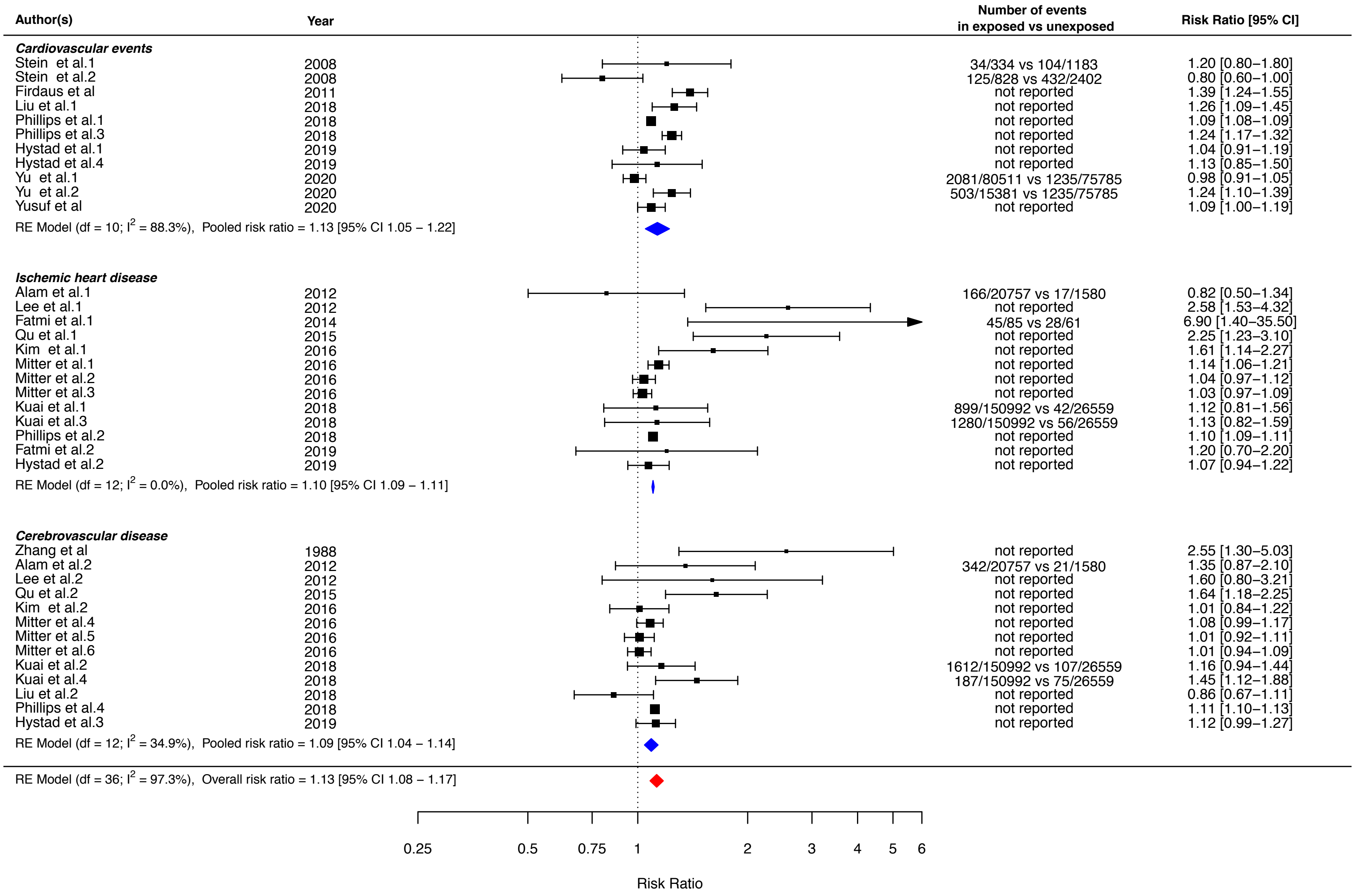

Figure S17: Pooled risk ratio for low birthweight associated with use of polluting fuels and technologies

Author(s)	Year	Number of events in exposed vs unexposed	Risk Ratio [95% CI]
Mavalankar et al.1	1992	234/676 vs 439/1462	1.23 [1.01-1.50]
Mavalankar et al.2	1992	252/694 vs 392/1415	1.49 [1.22-1.82]
Siddiqui et al	2008	83/366 vs 40/268	1.86 [1.11-3.14]
Tielsch et al	2009	3052/12010 vs 120/766	1.49 [1.25-1.77]
Sreeramareddy et al	2011	7634/34365 vs 2016/12774	1.07 [0.94-1.22]
Yucra et al	2011	49/77 vs 24/62	3.73 [1.14-12.10]
Abusalah et al	2012	55/79 vs 168/367	2.30 [1.20-4.70]
Amegah et al	2012	64/282 vs 15/161	1.41 [0.62-3.23]
Epstein et al.1	2013	not reported	1.51 [1.08-2.12]
Epstein et al.2	2013	not reported	1.24 [1.04-1.48]
Epstein et al.3	2013	not reported	1.57 [1.03-2.41]
Jiang et al.1	2015	70/340 vs 371/7336	1.09 [0.67-1.78]
Jiang et al.2	2015	42/115 vs 371/7336	2.51 [1.26-5.01]
Mukherjee et al.1	2015	not reported	0.70 [0.20-2.45]
Mukherjee et al.2	2015	not reported	1.90 [1.18-5.21]
Mukherjee et al.3	2015	not reported	5.10 [2.06-7.04]
Wylie et al	2015	286/1485 vs 48/301	0.95 [0.58-1.57]
Adeyemi et al	2016	not reported	1.07 [0.85-1.34]
Manyeh et al.1	2016	not reported	0.82 [0.65-1.04]
Manyeh et al.2	2016	not reported	0.95 [0.74-1.22]
Khan et al	2017	2140/11889 vs 328/1569	0.96 [0.81-1.13]
Pathirathna et al.1	2017	10/64 vs 1/3	0.37 [0.03-4.48]
Pathirathna et al.2	2017	2/9 vs 1/3	0.57 [0.03-10.07]
Balakrishnan et al.1	2018	34/171 vs 106/720	1.44 [0.99-2.21]
Balakrishnan et al.2	2018	17/98 vs 106/720	1.22 [0.69-2.13]
Admasie et al.1	2018	49/244 vs 17/165	3.83 [1.95-15.35]
Admasie et al.2	2018	104/633 vs 17/165	3.47 [0.99-12.24]
Suryadhi et al	2019	not reported	1.61 [1.37-1.89]
Weber et al	2020	30/279 vs 56/540	1.05 [0.57-1.93]

RE Model (df = 28; I^2 = 76.2%), Pooled risk ratio = 1.36 [95% CI 1.19 - 1.55]

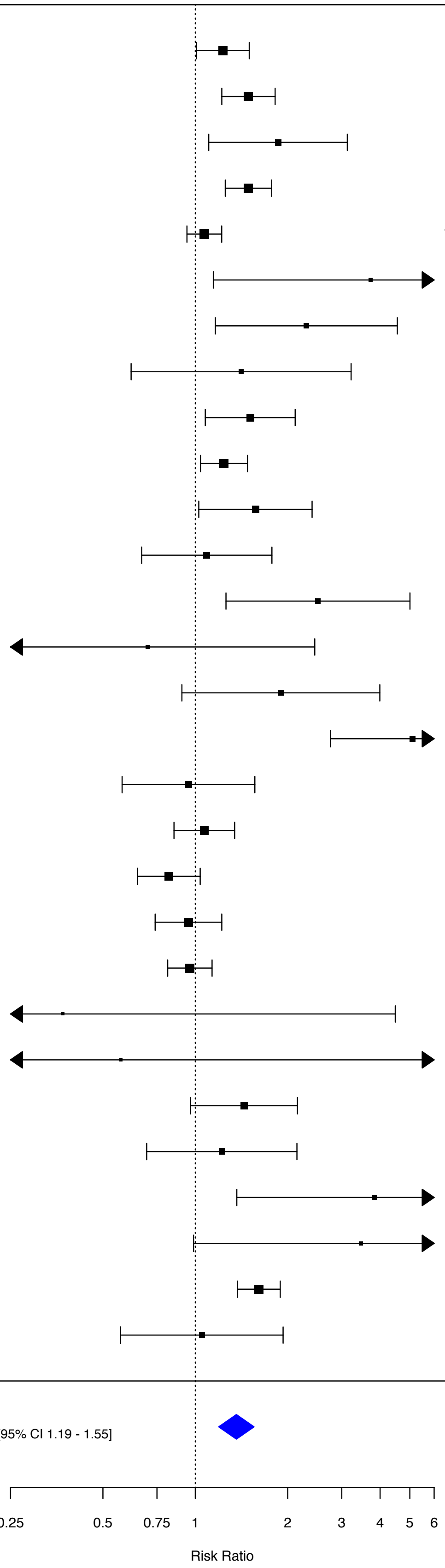

Figure S18: Pooled risk ratio for under-five mortality associated with use of polluting fuels and technologies

Author(s)	Year	Number of events in exposed vs unexposed	Risk Ratio [95% CI]
Wichmann et al	2006	not reported	1.99 [1.04-3.68]
Rinne et al	2007	not reported	2.20 [1.10-4.10]
Tielsch et al.1	2009	369/12305 vs 21/1021	1.17 [0.70-1.96]
Tielsch et al.2	2009	586/12522 vs 29/1029	1.21 [0.79-1.84]
Bassani et al.1	2010	1197/55835 vs 241/14161	0.95 [0.79-1.15]
Bassani et al.2	2010	1444/50984 vs 346/12702	0.91 [0.77-1.07]
Bassani et al.3	2010	1366/193865 vs 204/58693	1.30 [1.08-1.56]
Bassani et al.4	2010	1766/178144 vs 230/52271	1.33 [1.12-1.58]
Kashima et al.1	2010	not reported	1.29 [0.75-2.23]
Kashima et al.2	2010	not reported	1.47 [0.92-2.33]
Kashima et al.3	2010	not reported	1.05 [0.72-1.53]
Kashima et al.4	2010	not reported	1.30 [1.00-1.70]
Pandey et al	2012	not reported	1.23 [1.06-1.43]
Epstein et al.1	2013	not reported	2.88 [1.18-7.02]
Epstein et al.2	2013	not reported	0.84 [0.39-1.81]
Epstein et al.3	2013	not reported	24.15 [7.98-73.12]
Ezeh et al.1	2014	not reported	1.01 [0.73-1.26]
Ezeh et al.2	2014	not reported	1.92 [1.42-2.58]
Ezeh et al.3	2014	not reported	1.63 [1.09-2.42]
Kleimola et al.1	2015	not reported	1.34 [1.18-1.52]
Kleimola et al.2	2015	not reported	1.24 [1.14-1.34]
Kleimola et al.3	2015	not reported	1.12 [0.99-1.27]
Kleimola et al.4	2015	not reported	1.21 [1.12-1.30]
Naz et al.3	2015	850/16465 vs 73/1843	1.14 [0.83-1.55]
Patel et al.1	2015	not reported	1.82 [1.47-2.22]
Patel et al.2	2015	not reported	1.28 [0.91-1.76]
Akinyemi et al.1	2016	not reported	1.44 [1.18-1.76]
Akinyemi et al.2	2016	not reported	1.21 [0.94-1.56]
Naz et al.4	2016	10461/128573 vs 850/26498	1.30 [1.18-1.43]
Khan et al.1	2017	875/19022 vs 213/3873	0.96 [0.78-1.19]
Khan et al.2	2017	600/18750 vs 139/3861	1.03 [0.80-1.33]
Khan et al.3	2017	791/11633 vs 198/3882	0.88 [0.73-1.07]
Naz et al.2	2017	571/7462 vs 197/4045	1.22 [0.92-1.64]
Owili et al.1	2017	11383/111885 vs 1822/32037	1.21 [1.10-1.34]
Owili et al.2	2017	82756/612569 vs 1822/32037	1.20 [1.08-1.32]
Owili et al.3	2017	2200/20974 vs 1822/32037	1.01 [0.90-1.14]
Tripathy et al	2017	not reported	1.00 [0.90-1.10]
Naz et al.1	2018	989/17052 vs 25/1471	2.19 [1.37-3.51]
Nisha et al	2018	not reported	1.40 [1.01-2.10]
Samuel et al	2018	786/8248 vs 151/2735	1.23 [0.98-1.54]
Ghimere et al	2019	not reported	1.44 [1.09-1.91]
Suryadhi et al.1	2019	not reported	1.37 [1.13-1.66]
Suryadhi et al.2	2019	not reported	1.41 [1.22-1.63]
Weber et al	2020	7/279 vs 4/540	7.60 [1.67-36.00]

RE Model (df = 43; I^2 = 73.9%), Pooled risk ratio = 1.25 [95% CI 1.18 - 1.33]

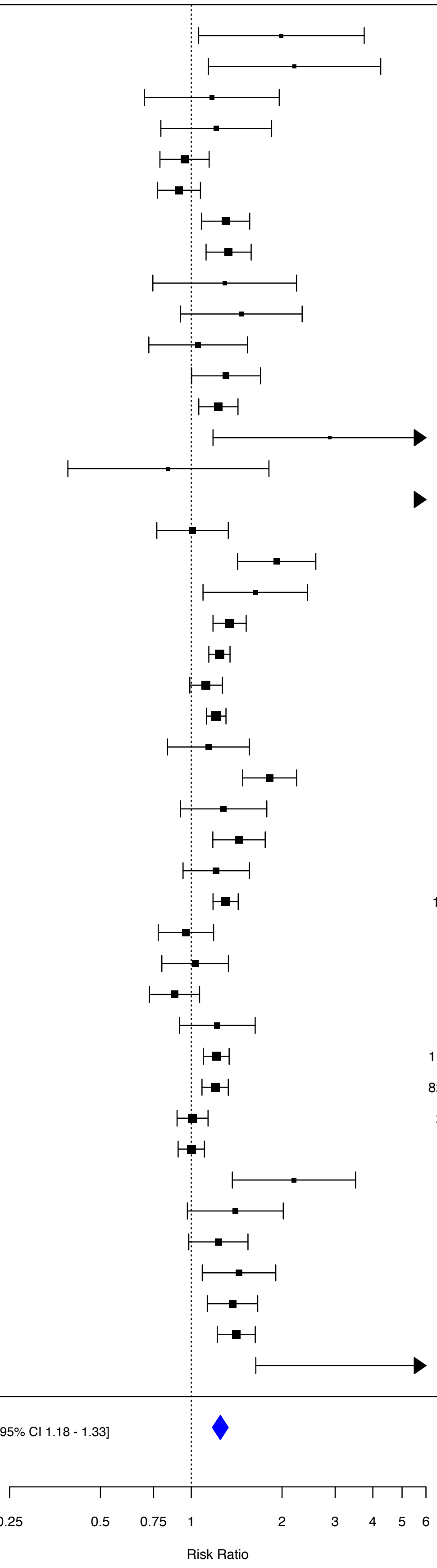

Figure S19: Pooled risk ratio for cardiovascular mortality associated with use of polluting fuels and technologies

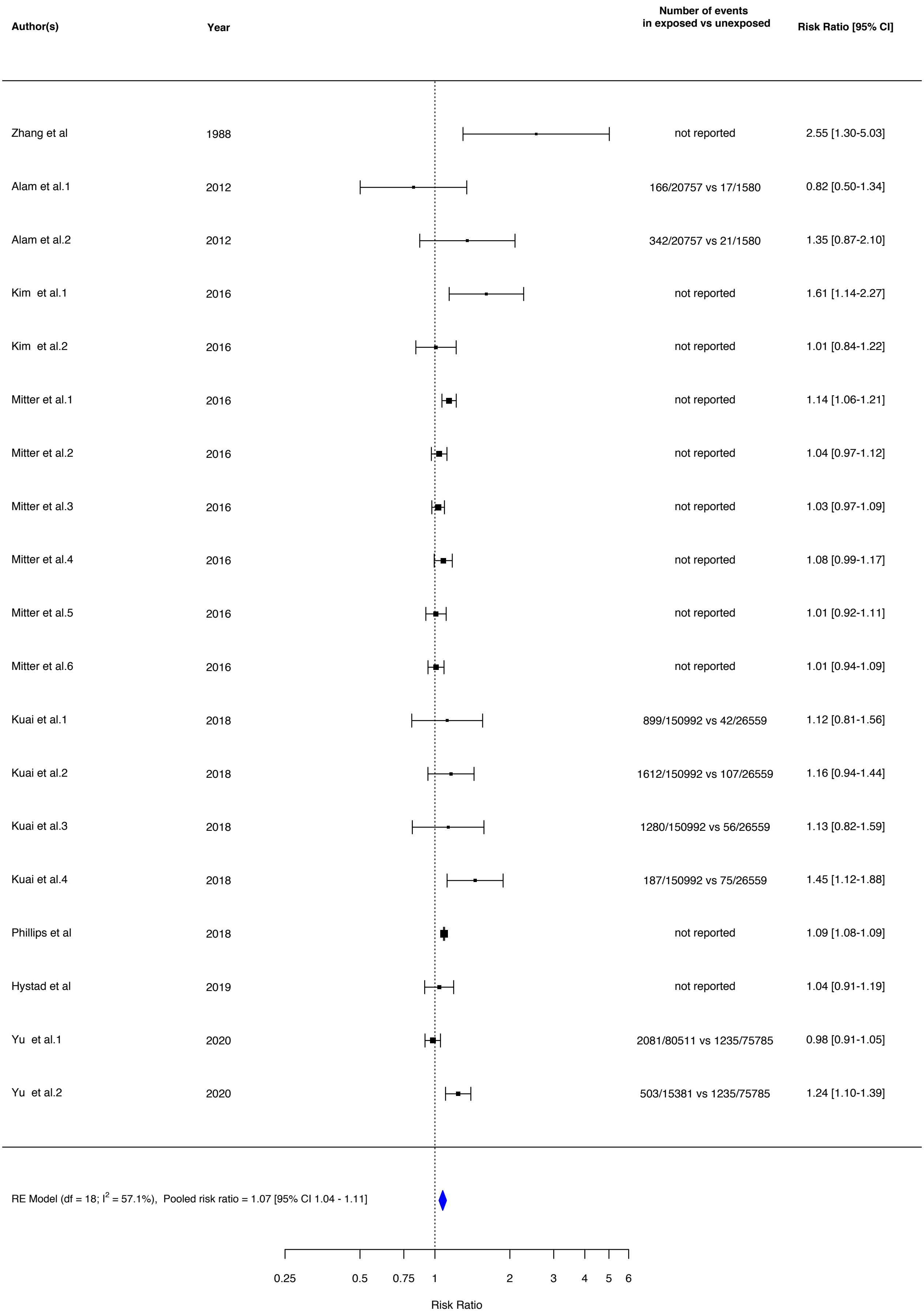

Figure S20: Pooled risk ratio for respiratory mortality associated with use of polluting fuels and technologies

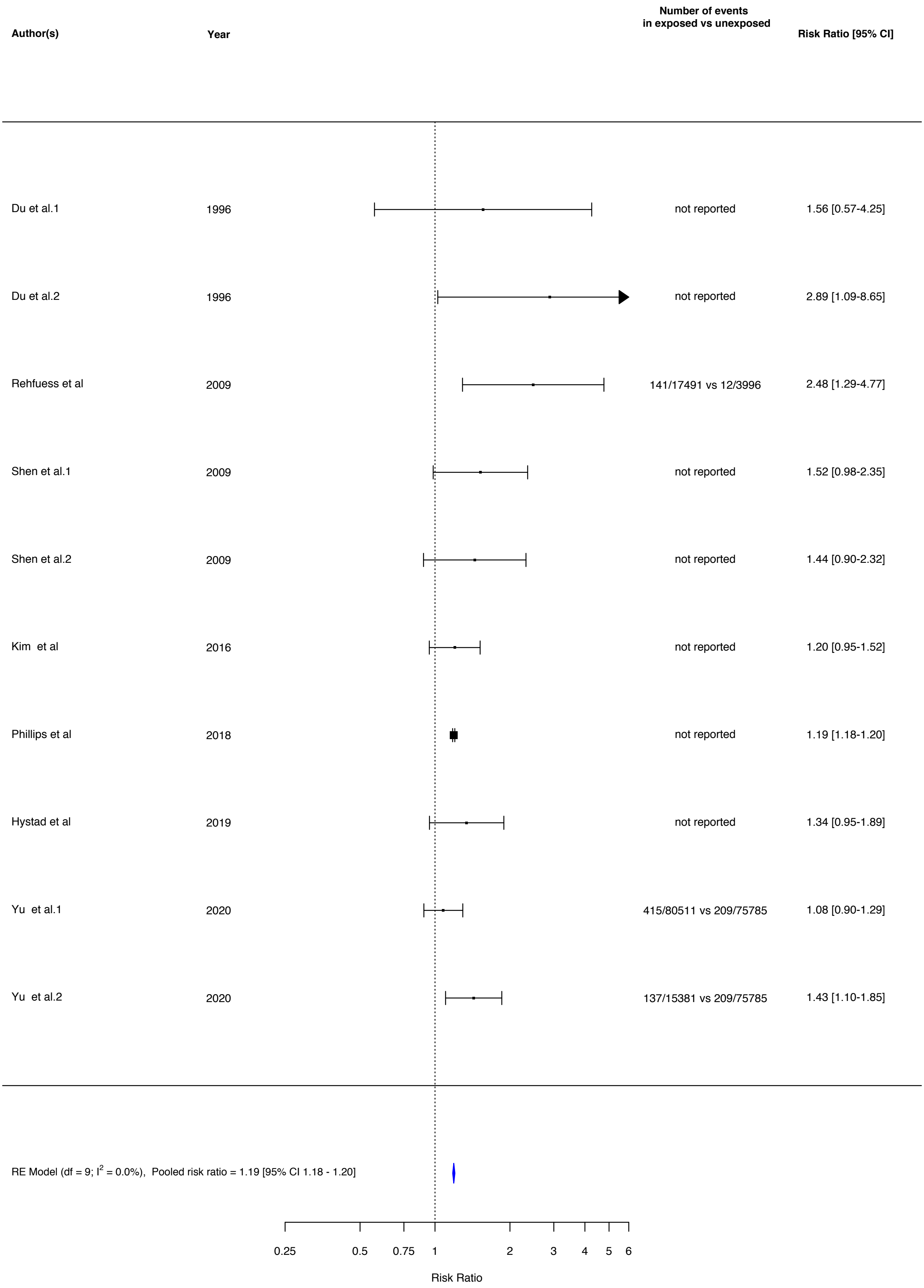

Figure S21: Pooled risk ratio for all-cause mortality associated with use of polluting fuels and technologies

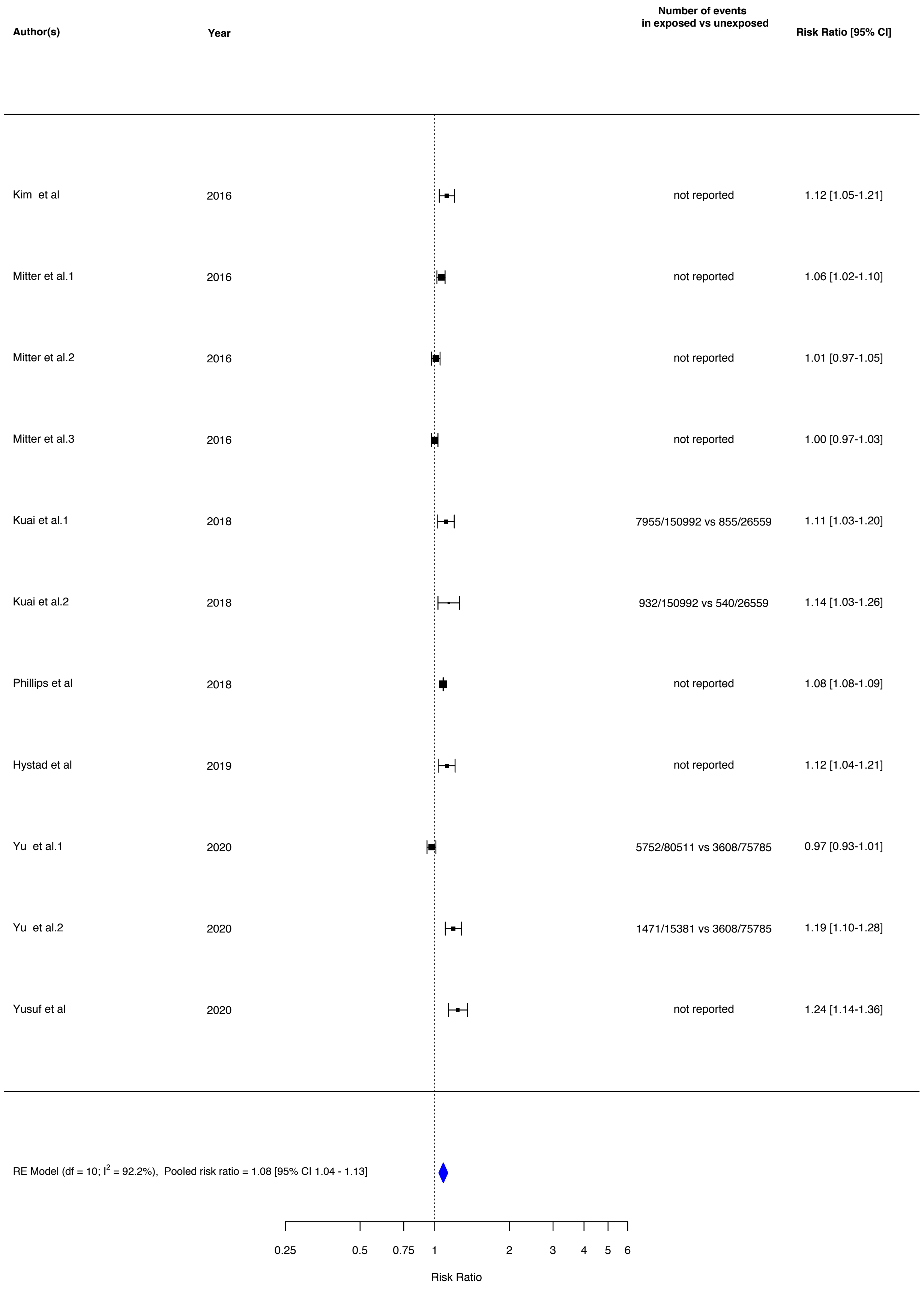

Figure S22: Pooled risk ratio for respiratory disease associated with use of gas for cooking or heating

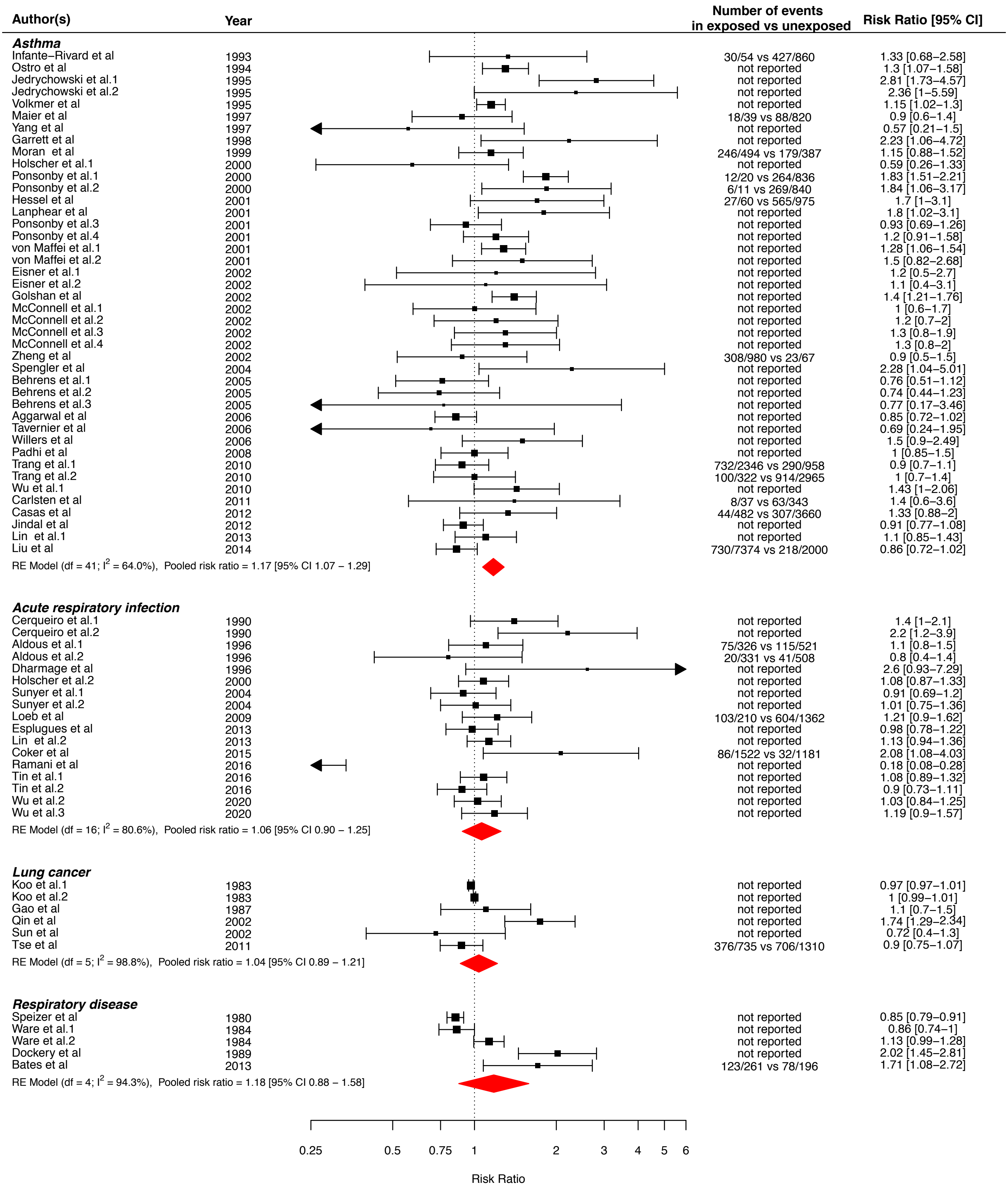

Figure S23: Pooled risk ratios for respiratory conditions and symptoms in studies comparing use of improved cook stoves and traditional cook stoves

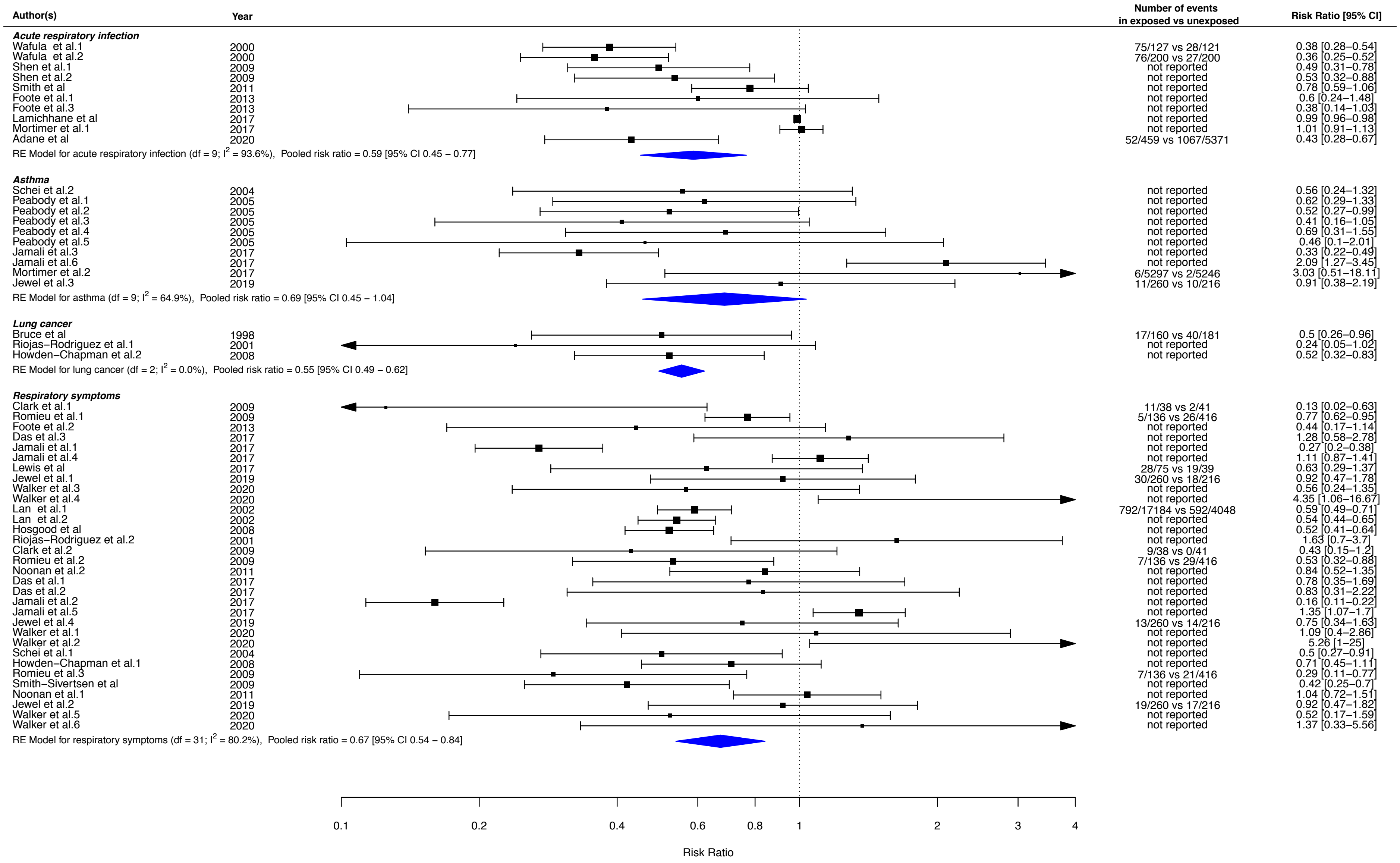

Figure S24: Pooled risk ratio for symptoms of wheeze associated with use of polluting fuels and technologies

Author(s)	Year	Number of events in exposed vs unexposed	Risk Ratio [95% CI]
Honicky et al	1985	17/31 vs 0/31	76.03 [4.27-1353.37]
Pope et al.1	1993	16/101 vs 16/182	1.83 [0.84-3.97]
Pope et al.2	1993	23/203 vs 36/409	1.2 [0.6-2.41]
Pope et al.3	1993	3/36 vs 3/42	1.07 [0.29-4]
Goren et al.1	1995	508/3657 vs 388/2469	0.87 [0.75-1]
Goren et al.2	1995	253/3463 vs 190/2319	0.88 [0.73-1.07]
Fischer et al.1	1998	not reported	0.94 [0.8-1.11]
Fischer et al.2	1998	not reported	0.96 [0.77-1.19]
Fischer et al.3	1998	not reported	0.93 [0.79-1.11]
Fischer et al.4	1998	not reported	1.06 [0.89-1.27]
Fischer et al.5	1998	not reported	0.84 [0.7-1]
Zacharasiewicz et al.1	1999	not reported	0.32 [0.04-2.32]
Zacharasiewicz et al.2	1999	not reported	0.7 [0.54-0.91]
Zacharasiewicz et al.3	1999	not reported	0.73 [0.39-1.35]
Zacharasiewicz et al.4	1999	not reported	0.95 [0.61-1.49]
Zacharasiewicz et al.5	1999	not reported	1.41 [0.4-4.97]
Zacharasiewicz et al.6	1999	not reported	0.89 [0.52-1.54]
Venn et al	2001	not reported	0.64 [0.44-0.93]
Zhang et al.1	2002	not reported	0.98 [0.7-1.4]
Zhang et al.2	2002	not reported	1.3 [0.9-1.9]
Zhang et al.3	2002	not reported	0.85 [0.6-1.2]
Belanger et al	2003	21/49 vs 351/784	0.76 [0.37-1.55]
Bothwell et al	2003	not reported	2.47 [1.83-3.3]
Guggisberg et al	2003	not reported	0.9 [0.2-4]
Uzun et al	2003	62/90 vs 15/87	10.63 [5.21-21.69]
Dagoye et al.1	2004	12/111 vs 122/3162	3.36 [1.77-6.36]
Dagoye et al.2	2004	54/1012 vs 122/3162	1.44 [1.02-2.05]
Dagoye et al.3	2004	150/3411 vs 6/231	2.72 [0.86-8.64]
Dagoye et al.4	2004	32/643 vs 6/231	2.73 [0.82-9.16]
Dagoye et al.5	2004	3/132 vs 55/2705	0.97 [0.23-4.08]
Dagoye et al.6	2004	62/1180 vs 2/54	1.1 [0.26-4.28]
Dagoye et al.7	2004	124/3051 vs 2/54	0.93 [0.22-3.88]
Dagoye et al.8	2004	56/2749 vs 2/109	0.76 [0.18-3.21]
Salo et al	2004	481/2075 vs 397/2455	1.57 [1.07-2.29]
Mommers et al	2005	not reported	0.93 [0.34-2.37]
Prietsch et al.1	2006	not reported	2.68 [1.44-4.98]
Prietsch et al.2	2006	not reported	2.57 [1.36-4.83]
Khalequzzaman et al	2007	26/65 vs 6/51	4 [1.1-16.2]
Noonan et al.1	2007	10/141 vs 9/116	0.91 [0.36-2.31]
Noonan et al.2	2007	4/62 vs 9/116	0.82 [0.24-2.78]
Dong et al	2008	not reported	0.76 [0.34-1.7]
Rudnai et al	2008	not reported	1.15 [0.92-1.42]
Winchmann et al	2009	206/1207 vs 67/413	0.93 [0.54-1.62]
Bueso et al.1	2010	not reported	1.2 [0.46-3.15]
Bueso et al.2	2010	not reported	1.11 [0.53-2.34]
Bueso et al.3	2010	not reported	0.68 [0.21-2.18]
Bueso et al.4	2010	not reported	2.27 [0.78-6.59]
Bueso et al.5	2010	not reported	0.95 [0.33-2.75]
Desalu et al	2010	14/161 vs 3/108	3.82 [0.83-17.54]
Hersoug et al.1	2010	34/159 vs 531/2347	1.17 [0.77-1.79]
Hersoug et al.2	2010	30/144 vs 531/2347	1.23 [0.79-1.92]
Hersoug et al.3	2010	69/296 vs 531/2347	1.31 [0.95-1.81]
Hersoug et al.4	2010	63/339 vs 531/2347	0.88 [0.63-1.22]
da Silva et al.1	2012	17/60 vs 68/466	2.33 [1.25-4.38]
da Silva et al.2	2012	52/218 vs 68/466	1.78 [1.18-2.69]
da Silva et al.3	2012	20/69 vs 51/360	2.46 [1.34-4.47]
da Silva et al.4	2012	52/229 vs 51/360	1.77 [1.15-2.72]
Kraai et al	2013	not reported	2.12 [1.18-3.84]
Solis-Soto et al.1	2013	not reported	0.86 [0.62-1.19]
Solis-Soto et al.2	2013	not reported	1.94 [1.16-3.26]
Thacher et al	2013	not reported	1.2 [0.45-3.18]
Zhou et al	2013	not reported	0.53 [0.27-1.03]
Alim et al	2014	32/224 vs 11/196	2.58 [0.79-8.44]
Kurmi et al.1	2014	91/382 vs 32/380	2.48 [1.28-4.82]
Kurmi et al.2	2014	152/463 vs 41/423	4.62 [2.71-7.87]
Sanya et al	2014	32/65 vs 11/21	0.88 [0.33-2.36]
Shirinde et al.1	2014	not reported	0.06 [0.83-1.37]
Shirinde et al.2	2014	not reported	1.16 [0.83-1.62]
Mukherjee et al	2014	62/681 vs 14/438	3.03 [1.68-5.49]
Choi et al.1	2015	27/161 vs 40/386	0.79 [0.24-2.62]
Choi et al.2	2015	9/274 vs 18/571	1.21 [0.31-4.66]
Kurti et al	2016	14/31 vs 3/36	9.06 [2.28-35.92]
Obaseki et al.1	2017	3.44/215 vs 3.76/376	1.2 [0.2-5.6]
Obaseki et al.2	2017	15.568/556 vs 3.76/376	2 [0.7-5.8]
Rumchev et al	2017	not reported	1.82 [1.18-2.82]
Devien et al.1	2018	not reported	0.44 [0.13-1.1]
Devien et al.2	2018	not reported	0.52 [0.08-1.9]
Misra et al	2018	not reported	1.41 [0.72-2.77]
Panigrahi et al	2018	73/573 vs 22/344	2.14 [1.3-3.51]
Juntarawijit et al.1	2019	not reported	1.35 [0.16-11.78]
Juntarawijit et al.2	2019	not reported	1.68 [0.8-3.55]
Norback et al	2019	not reported	1.07 [0.93-1.3]
Sana et al	2019	184/1015 vs 90/690	1.37 [1.01-1.87]

RE Model (df = 82; I^2 = 90.0%), Pooled risk ratio = 1.27 [95% CI 1.09 - 1.49]

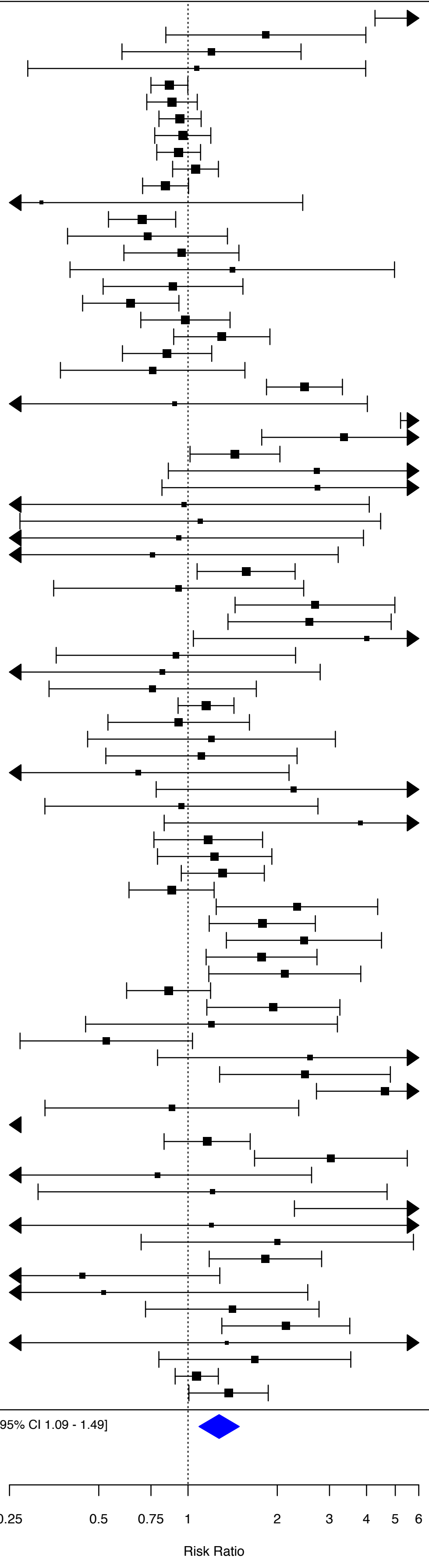

Figure S25: Pooled risk ratio for symptoms of cough associated with use of polluting fuels and technologies

Author(s)	Year	Number of events in exposed vs unexposed	Risk Ratio [95% CI]
Honicky et al	1985	14/31 vs 1/32	25.53 [3.09-211.26]
Viegi et al.1	1991	not reported	1.66 [2.65-1.04]
Viegi et al.2	1992	not reported	2.52 [1.07-5.93]
Pope et al.1	1993	10/101 vs 22/182	0.8 [0.35-1.81]
Pope et al.2	1993	25/203 vs 51/409	1.03 [0.97-1.1]
Pope et al.3	1993	9/36 vs 7/42	3.07 [1.23-7.65]
Xu et al	1993	not reported	1.4 [0.7-2.7]
Ostro et al	1994	not reported	1.44 [1.05-1.99]
Dutt et al	1996	34/197 vs 8/98	2.35 [1.04-5.29]
Fischer et al.1	1998	not reported	1.22 [0.99-1.51]
Fischer et al.2	1998	not reported	0.81 [0.66-0.98]
Fischer et al.3	1998	not reported	1.02 [0.85-1.23]
Qian et al	2000	not reported	1.28 [1.04-1.57]
Zhang et al.1	2002	not reported	0.98 [0.6-1.5]
Zhang et al.2	2002	not reported	1.4 [0.9-2.3]
Zhang et al.3	2002	not reported	1.2 [0.8-1.9]
Singh et al	2002	17/327 vs 7/197	1.49 [0.61-3.66]
Belanger et al.1	2003	34/49 vs 394/784	1.68 [0.89-3.2]
Belanger et al.2	2003	not reported	1.04 [0.27-3.97]
Bothwell et al	2003	not reported	2.2 [1.65-2.8]
Guggisberg et al	2003	not reported	1.1 [0.2-4.8]
Uzun et al	2003	55/90 vs 24/87	4.13 [2.19-7.77]
Salo et al	2004	495/2075 vs 622/2455	0.92 [0.76-1.11]
Mommers et al	2005	not reported	1.52 [0.72-3.23]
Shrestha et al	2005	47/125 vs 6/43	3.75 [1.45-9.69]
Siddiqui et al	2005	44/57 vs 29/65	3.8 [1.7-8.6]
Khalequzzaman et al	2007	45/65 vs 26/51	1.9 [0.6-6.3]
Rinne et al	2007	not reported	1.2 [0.9-1.8]
Dong et al.1	2008	not reported	1.3 [0.72-2.36]
Dong et al.2	2008	not reported	1.17 [0.81-1.7]
Padhi et al	2008	not reported	4.23 [3.86-4.75]
Desalu et al	2010	22/161 vs 4/108	4.82 [1.44-16.1]
Hersoug et al.1	2010	29/158 vs 387/2347	1.37 [0.86-2.16]
Hersoug et al.2	2010	15/143 vs 387/2347	0.65 [0.35-1.18]
Hersoug et al.3	2010	40/295 vs 387/2347	0.99 [0.66-1.47]
Hersoug et al.4	2010	44/340 vs 387/2347	0.93 [0.64-1.36]
Mustapha et al.1	2011	not reported	1.53 [0.63-3.7]
Mustapha et al.2	2011	not reported	1.76 [0.75-4.13]
Stankovic et al	2011	not reported	1.36 [1.07-1.74]
da Silva et al.1	2012	32/60 vs 132/466	2.93 [1.68-5.1]
da Silva et al.2	2012	24/69 vs 114/360	1.64 [1.16-2.31]
Dohoo et al	2012	8/31 vs 6/30	1.39 [0.42-4.63]
Ingale et al.1	2013	not reported	0.99 [0.73-1.49]
Ingale et al.2	2013	not reported	0.83 [0.63-1.3]
Ingale et al.3	2013	not reported	1.84 [0.95-2.1]
Ingale et al.4	2013	not reported	1.02 [0.75-1.48]
Ingale et al.5	2013	not reported	1.79 [0.91-1.98]
Ingale et al.6	2013	not reported	1.01 [0.66-1.09]
Thacher et al	2013	not reported	1.57 [0.74-3.36]
Sukhsohale et al.1	2013	25/302 vs 12/192	1.35 [0.66-2.76]
Sukhsohale et al.2	2013	4/73 vs 12/192	0.87 [0.27-2.79]
Sukhsohale et al.3	2013	15/243 vs 12/192	0.99 [0.45-2.16]
Kurmi et al.1	2014	19/382 vs 19/380	0.87 [0.28-2.69]
Kurmi et al.2	2014	22/463 vs 16/423	0.41 [0.15-1.18]
Liu et al.1	2014	271/1243 vs 353/2040	1.24 [1.01-1.51]
Liu et al.2	2014	10/47 vs 353/2040	0.85 [0.35-2.06]
Liu et al.3	2014	not reported	1 [0.8-1.26]
Choi et al.1	2015	118/161 vs 212/386	1.95 [0.9-4.24]
Choi et al.2	2015	134/274 vs 215/571	1.34 [0.63-2.85]
Obaseki et al.1	2017	23.865/215 vs 25.568/376	1.5 [0.8-2.9]
Obaseki et al.2	2017	63.384/556 vs 25.568/376	1.7 [1-2.8]
Oluwole et al	2017	200/865 vs 143/825	1.44 [1.14-1.84]
Tripathy et al	2017	not reported	1.5 [1.3-1.7]
Devien et al.1	2018	not reported	0.96 [0.39-2.03]
Devien et al.2	2018	not reported	1.97 [0.45-6.14]
Juntarawijit et al.1	2019	not reported	1.59 [0.67-3.77]
Juntarawijit et al.2	2019	not reported	0.77 [0.55-1.09]
Sana et al	2019	287/1015 vs 166/690	1.15 [0.9-0.15]

RE Model (df = 67; I^2 = 82.6%), Pooled risk ratio = 1.37 [95% CI 1.23 - 1.52]

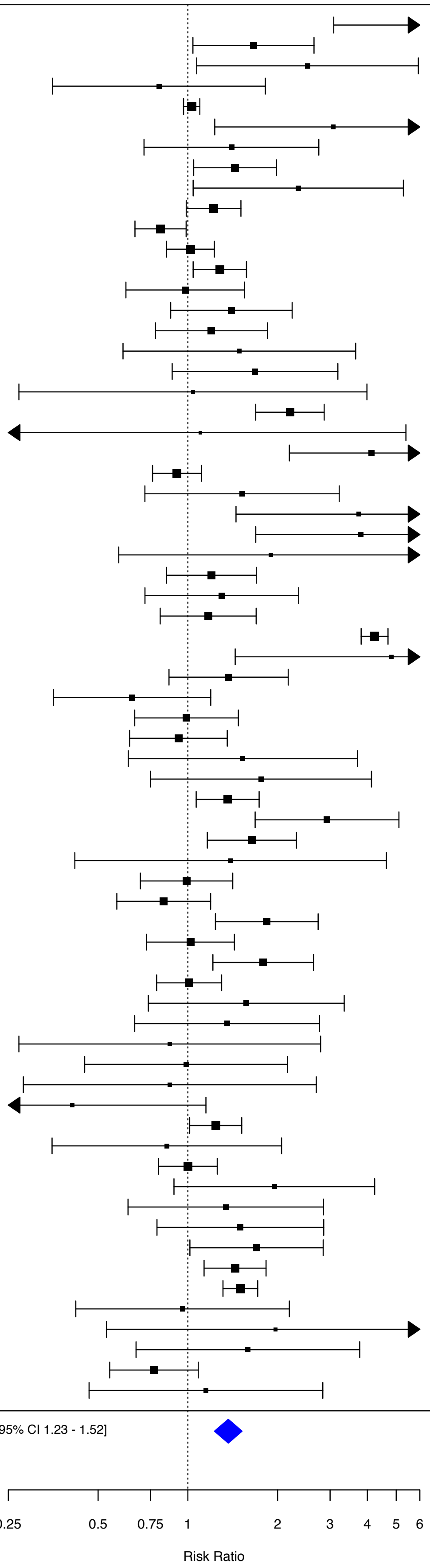

Figure S26: Pooled risk ratio for symptoms of shortness of breath associated with use of polluting fuels and technologies

Author(s)	Year	Number of events in exposed vs unexposed	Risk Ratio [95% CI]
Viegi et al	1991	not reported	1.73 [1.12-2.67]
Pope et al.1	1993	5/101 vs 14/182	0.74 [0.25-2.16]
Pope et al.2	1993	26/203 vs 36/409	1.88 [0.93-3.81]
Pope et al.3	1993	6/36 vs 3/42	3.55 [1.2-10.5]
Xu et al	1993	not reported	1.3 [0.6-2.6]
Ostro et al	1994	not reported	1.7 [1.24-2.31]
Dutt et al	1996	17/197 vs 3/98	2.99 [0.85-10.46]
Fischer et al.1	1998	not reported	0.91 [0.73-1.14]
Fischer et al.2	1998	not reported	0.84 [0.64-1.1]
Fischer et al.3	1998	not reported	1.02 [0.81-1.28]
Fischer et al.4	1998	not reported	1 [0.79-1.25]
Fischer et al.5	1998	not reported	0.86 [0.62-1.19]
Guggisberg et al	2003	not reported	1.1 [0.3-4.5]
Uzun et al	2003	46/90 vs 21/87	3.29 [1.73-6.24]
Shrestha et al	2005	41/125 vs 5/43	3.66 [1.34-9.99]
Siddiqui et al	2005	44/57 vs 30/65	3.9 [1.7-8.8]
Khalequzzaman et al	2007	24/65 vs 6/51	6.3 [1.6-29.1]
Desalu et al	2010	19/161 vs 7/108	1.54 [0.26-1.62]
Stankovic et al	2011	not reported	1.4 [1.12-1.75]
da Silva et al.1	2012	14/60 vs 49/466	2.59 [1.32-5.09]
da Silva et al.2	2012	17/69 vs 44/360	2.29 [1.21-4.32]
Dohoo et al	2012	16/31 vs 9/30	2.49 [0.87-7.12]
Sukhsohale et al.1	2013	5/252 vs 2/192	1.92 [0.37-10.02]
Sukhsohale et al.2	2013	3/73 vs 2/192	4.07 [0.67-24.88]
Sukhsohale et al.3	2013	3/243 vs 2/192	1.19 [0.2-7.18]
Kurmi et al.1	2014	48/382 vs 9/380	7.88 [2.84-21.88]
Kurmi et al.2	2014	86/463 vs 30/423	3.9 [2-7.79]
Mukherjee et al	2014	398/681 vs 87/681	9.6 [7.32-12.6]
Choi et al	2015	50/161 vs 83/386	1.3 [0.51-3.34]
Ngahane et al	2015	32/145 vs 8/155	5.2 [2.31-11.73]
Rumchev et al	2017	not reported	2.59 [1.08-6.19]
Misra et al.1	2018	not reported	1.06 [0.6-1.87]
Misra et al.2	2018	not reported	1.29 [0.65-2.56]
Juntarawijit et al.1	2019	not reported	1.95 [0.52-7.25]
Juntarawijit et al.2	2019	not reported	1.27 [0.7-2.3]
Sana et al	2019	209/1015 vs 114/690	1.11 [0.83-1.48]

RE Model (df = 35; I^2 = 86.0%), Pooled risk ratio = 1.88 [95% CI 1.49 - 2.37]

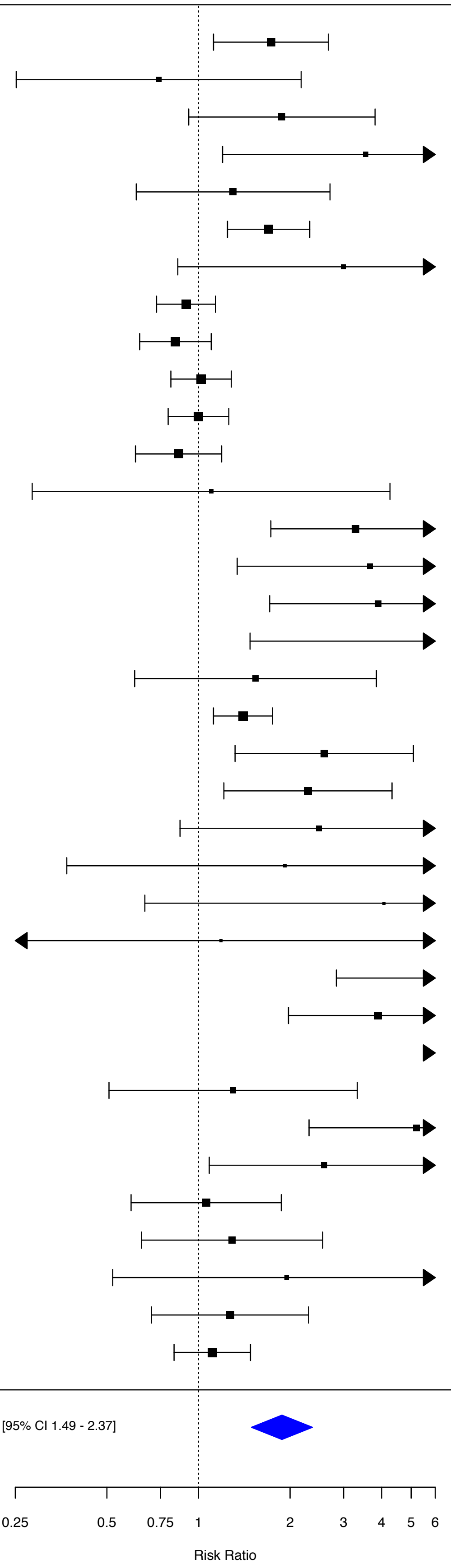

Figure S27: Pooled mean difference in birth weight associated with use of polluting fuels and technologies.

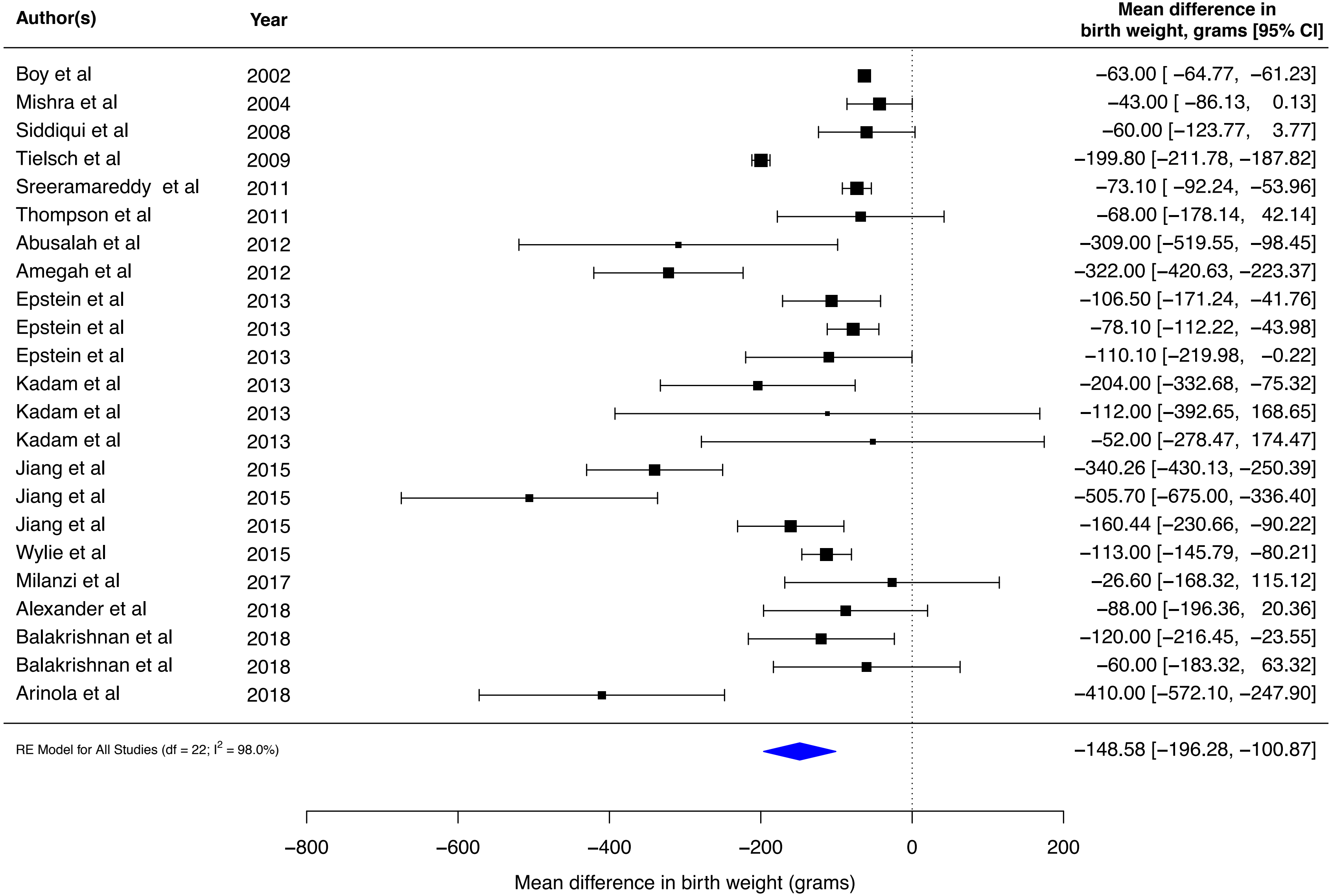

Figure S28: Pooled risk ratio for asthma and respiratory symptoms per 10 ppb increase in indoor nitrogen dioxide.

Author(s)	Year	Risk Ratio [95% CI] (per 10 ppb increment)
Asthma		
Neas et al.4	1991	0.94 [0.71–1.23]
Garrett et al.1	1998	1 [0.58–1.66]
Shima et al.2	2000	0.87 [0.51–1.43]
Wu et al	2010	1.19 [1–1.42]
Belanger et al.1	2013	1.88 [1.02–3.57]
RE Model for asthma (df = 4; I^2 = 0.0%), Pooled risk ratio = 1.11 [95% CI 0.97 – 1.27]		
Wheeze		
Neas et al.2	1991	1.1 [0.93–1.32]
Garrett et al.2	1998	1.3 [0.74–2.25]
Shima et al.1	2000	0.73 [0.45–1.14]
Smith et al.1	2000	0.99 [0.97–1.01]
Belanger et al.3	2003	1.1 [0.87–1.4]
Belanger et al.5	2003	1.1 [0.96–1.25]
Emenius et al	2003	1.12 [0.57–2.2]
vanStrien et al.1	2004	1.02 [0.74–1.41]
Belanger et al.7	2006	1.23 [1.02–1.49]
Belanger et al.10	2006	0.99 [0.84–1.17]
Nitschke et al.1	2006	0.98 [0.92–1.04]
Nitschke et al.2	2006	1 [0.9–1.11]
Esplugues et al.1	2011	1.14 [0.75–1.71]
Belanger et al.2	2013	2.22 [1.19–4.12]
Hansel et al.2	2013	0.91 [0.77–1.07]
RE Model for wheeze (df = 14; I^2 = 0.0%), Pooled risk ratio = 0.99 [95% CI 0.97 – 1.01]		
Cough		
Neas et al.3	1991	1.12 [0.91–1.37]
Smith et al.3	2000	1.01 [0.99–1.03]
Belanger et al.4	2003	1.01 [0.81–1.26]
Belanger et al.6	2003	1.21 [1.05–1.4]
vanStrien et al.2	2004	1.26 [0.95–1.67]
Belanger et al.8	2006	1.03 [0.87–1.22]
Belanger et al.11	2006	1.03 [0.88–1.21]
Nitschke et al.5	2006	1.01 [0.97–1.05]
Nitschke et al.6	2006	0.99 [0.96–1.02]
Hansel et al.4	2008	1.02 [0.98–1.06]
Hansel et al.5	2008	1.07 [1.03–1.11]
Esplugues et al.2	2011	1.73 [0.93–3.21]
Hansel et al.3	2013	0.99 [0.75–1.28]
RE Model for cough (df = 12; I^2 = 43.7%), Pooled risk ratio = 1.03 [95% CI 1.00 – 1.05]		
Shortness of breath		
Neas et al.1	1991	1.15 [0.95–1.37]
Smith et al.2	2000	1 [0.98–1.15]
vanStrien et al.3	2004	1.72 [1.14–2.62]
Belanger et al.9	2006	1.13 [0.92–1.38]
Belanger et al.12	2006	0.91 [0.72–1.14]
Nitschke et al.3	2006	1 [0.98–1.03]
Nitschke et al.4	2006	1.03 [1.01–1.05]
Hansel et al.1	2013	1.23 [1.1–1.38]
RE Model for shortness of breath (df = 7; I^2 = 84.0%), Pooled risk ratio = 1.06 [95% CI 1.00 – 1.13]		

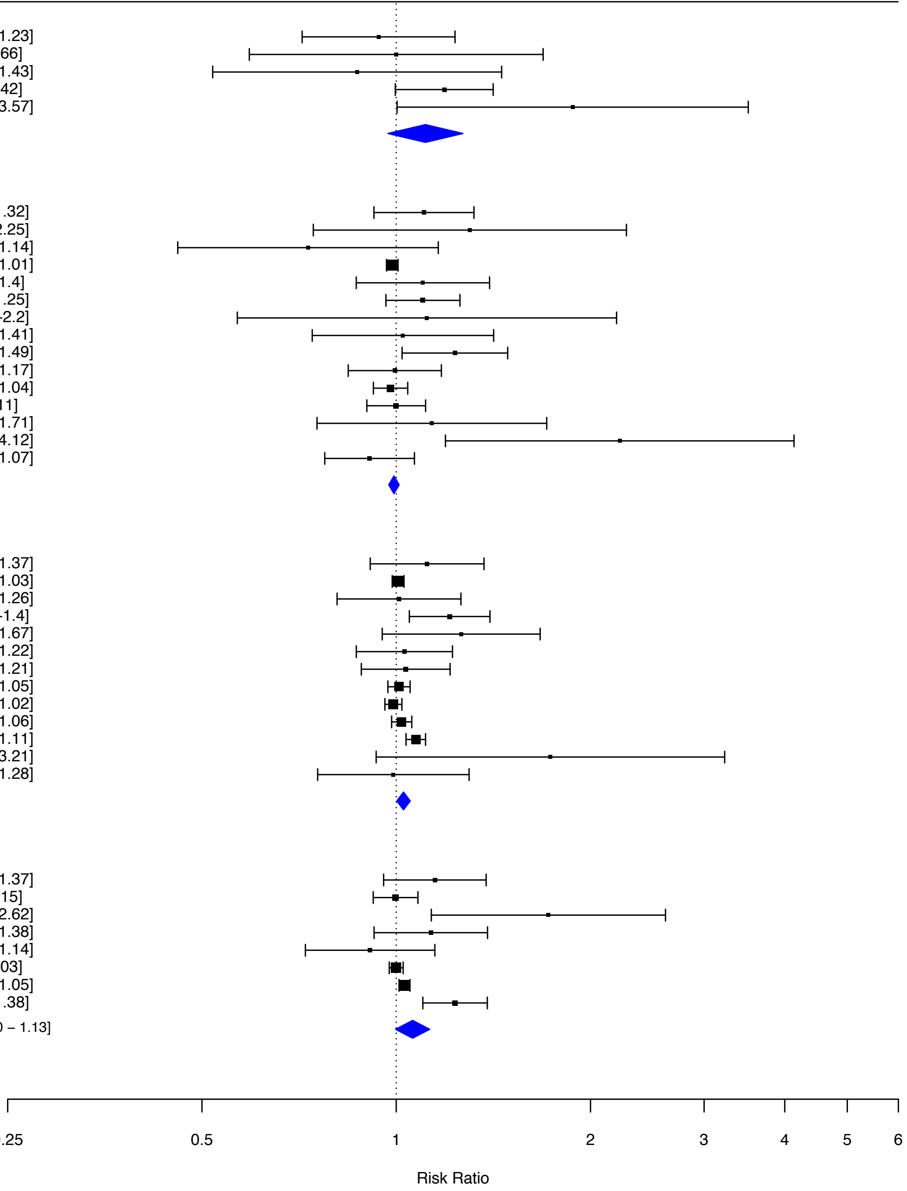

Figure S29: Pooled risk ratio for asthma and respiratory symptoms per 10 µg/m3 increase in indoor PM2.5

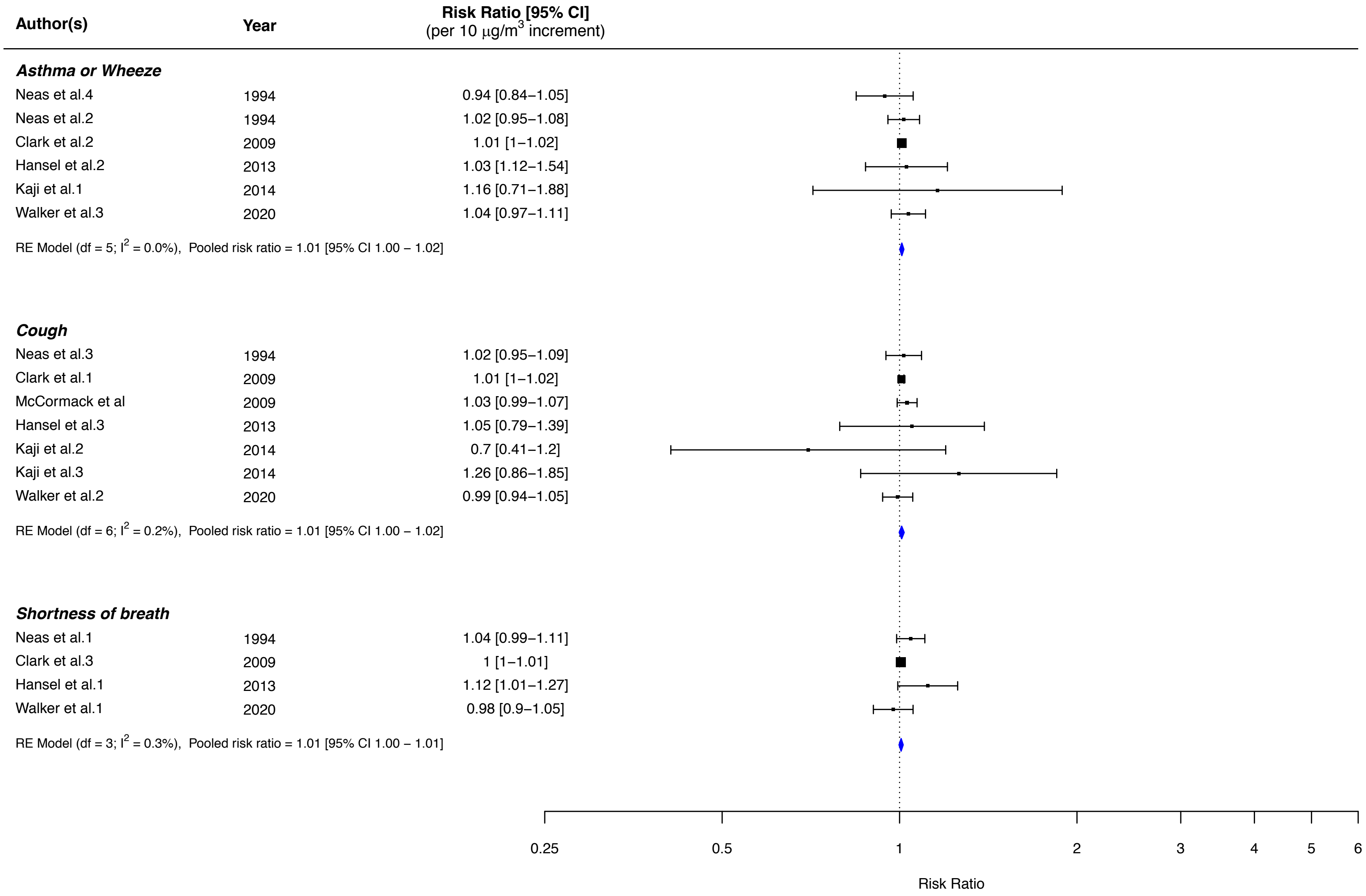

Figure S30: Pooled risk ratios for cardiorespiratory and adverse pregnancy outcomes associated with use of polluting fuels and technologies across studies assessed to be at low or moderate risk of bias.

Disease	No. of Estimates	I^2	Pooled risk ratios [95% CI]
Respiratory diseases			
Asthma	7	0%	0.89 (0.76–1.04)
COPD	57	97.6%	1.74 (1.43–2.11)
ARI	27	96.8%	1.54 (1.26–1.89)
Lung cancer	66	97%	1.74 (1.45–2.1)
Tuberculosis	28	63.7%	1.17 (0.92–1.49)
Cardiovascular diseases			
Cerebrovascular disease	7	0%	1.05 (1.01–1.1)
Ischemic heart disease	7	30.6%	1.08 (1.03–1.14)
Adverse pregnancy outcomes			
Low birth weight	13	30.8%	1.39 (1.18–1.64)
Stillbirth	8	0%	1.31 (1.21–1.42)

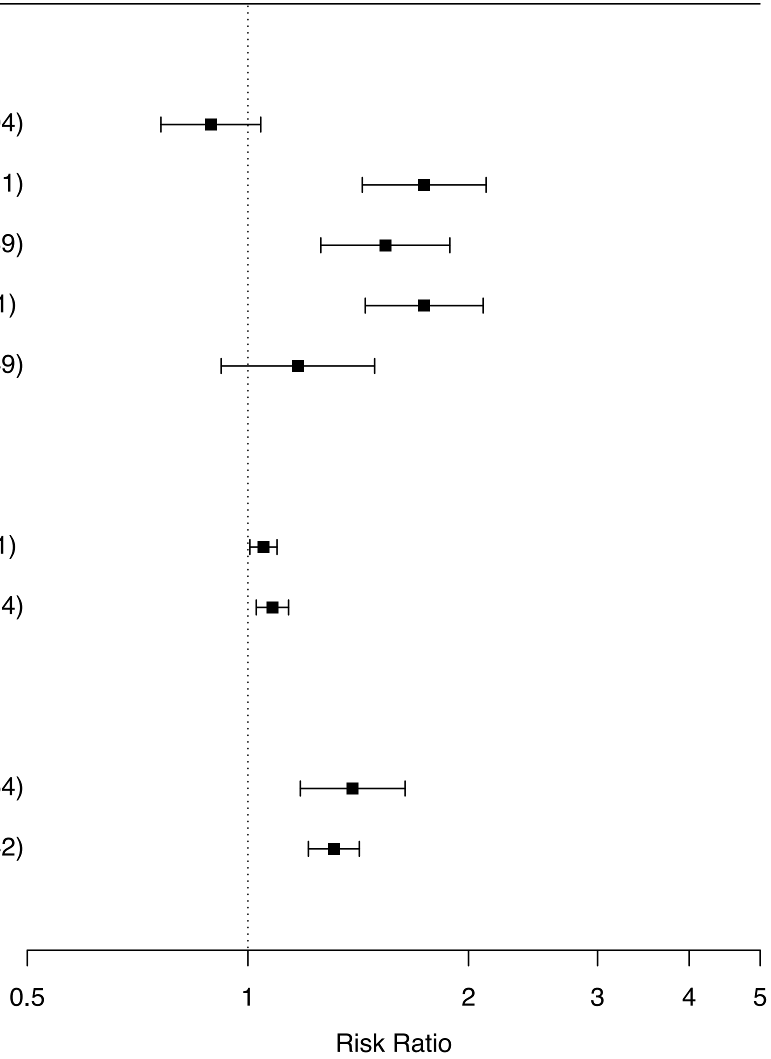

Figure S31: Pooled risk ratios for mortality associated with use of polluting fuels and technologies across studies assessed to be at low or moderate risk of bias.

Mortality	No. of Estimates	I^2	Pooled risk ratios [95% CI]
Under–five mortality	9	73.5%	1.24 (1.05–1.47)
Cardiovascular mortality	13	26.3%	1.07 (1.03–1.11)
Respiratory mortality	3	0%	1.27 (1.01–1.58)
All–cause mortality	7	84%	1.08 (1.03–1.14)

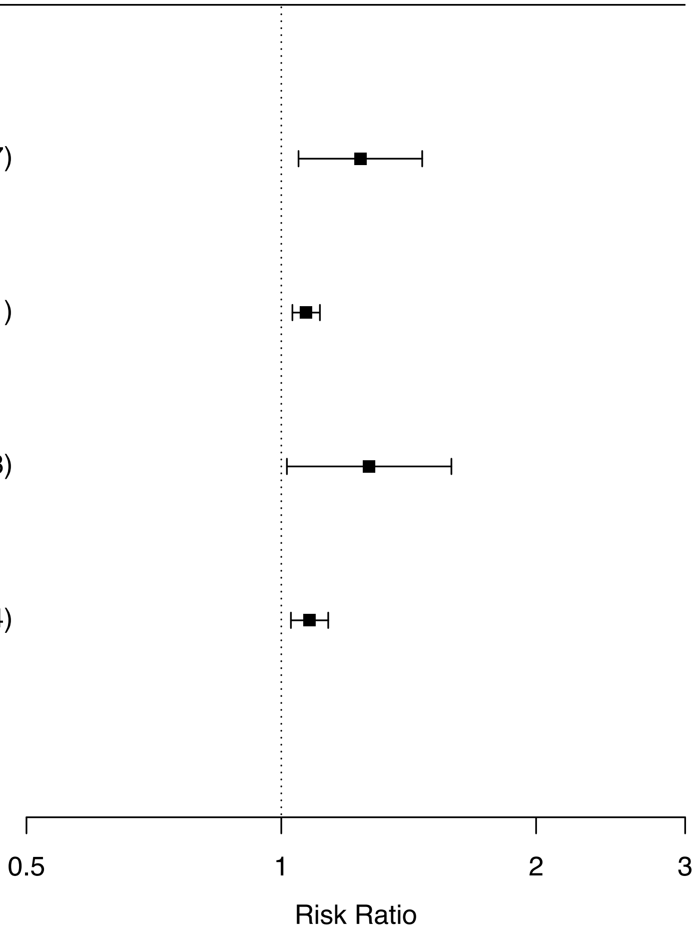

Figure S32: Sensitivity analysis of risk ratios for cardiorespiratory and adverse pregnancy outcomes associated with use of polluting fuels and technologies across longitudinal studies.

Disease	No. of Estimates	I^2	Pooled risk ratios [95% CI]
Respiratory diseases			
Asthma	17	91.8%	1.68 (1.22–2.33)
COPD	15	85%	1.21 (1.12–1.31)
ARI (adults)	10	94.9%	1.47 (1.26–1.7)
ARI (paediatric)	15	76.7%	1.39 (1.16–1.68)
Lung cancer	8	88.2%	1.39 (0.99–1.96)
Respiratory disease	20	83.5%	1.31 (1.25–1.37)
Cardiovascular diseases			
Cerebrovascular disease	10	1.8%	1.06 (1.01–1.1)
Ischemic heart disease	8	22.6%	1.08 (1.03–1.13)
Cardiovascular events	7	77.5%	1.15 (1.05–1.26)
Adverse pregnancy outcomes			
Low birth weight	9	0%	1.45 (1.27–1.66)
Stillbirth	3	0%	1.5 (1.25–1.79)

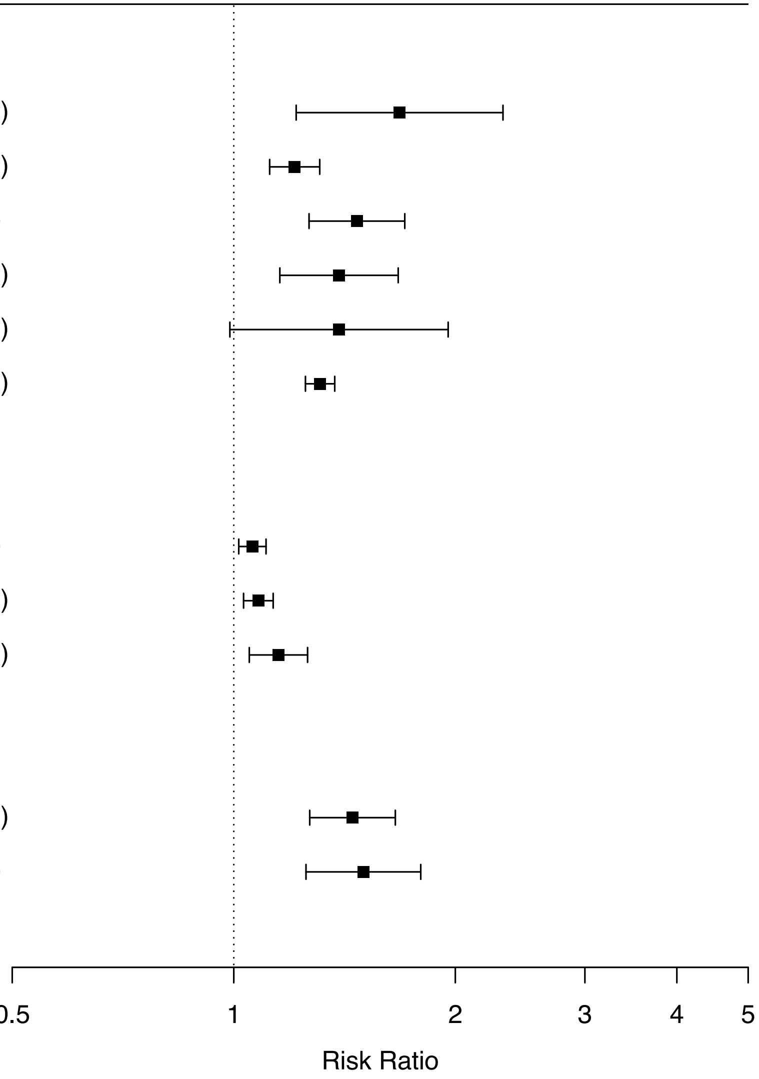

Figure S33: Pooled risk ratios for cardiorespiratory and paediatric conditions in studies comparing use of polluting fuels or technologies and clean fuels

Disease	No. of Estimates	I^2	Pooled risk ratios [95% CI]
Respiratory diseases			
Asthma	53	94.6%	1.18 (1–1.4)
COPD	56	98.1%	1.44 (1.18–1.75)
ARI (adults)	16	98.5%	1.4 (1.05–1.86)
ARI (paediatric)	84	98.3%	1.31 (1.22–1.4)
Lung cancer	26	54.3%	1.46 (1.28–1.66)
Tuberculosis	26	83.5%	1.09 (0.84–1.43)
Respiratory disease	26	81.2%	1.31 (1.25–1.38)
Cardiovascular diseases			
Cerebrovascular disease	4	0%	1.18 (1.07–1.3)
Ischemic heart disease	5	0%	1.08 (0.96–1.2)
Adverse pregnancy and pediatric outcomes			
Low birth weight	21	80.5%	1.36 (1.15–1.62)
Stillbirth	12	0%	1.28 (1.19–1.38)
Under–five death	27	77.1%	1.27 (1.18–1.37)

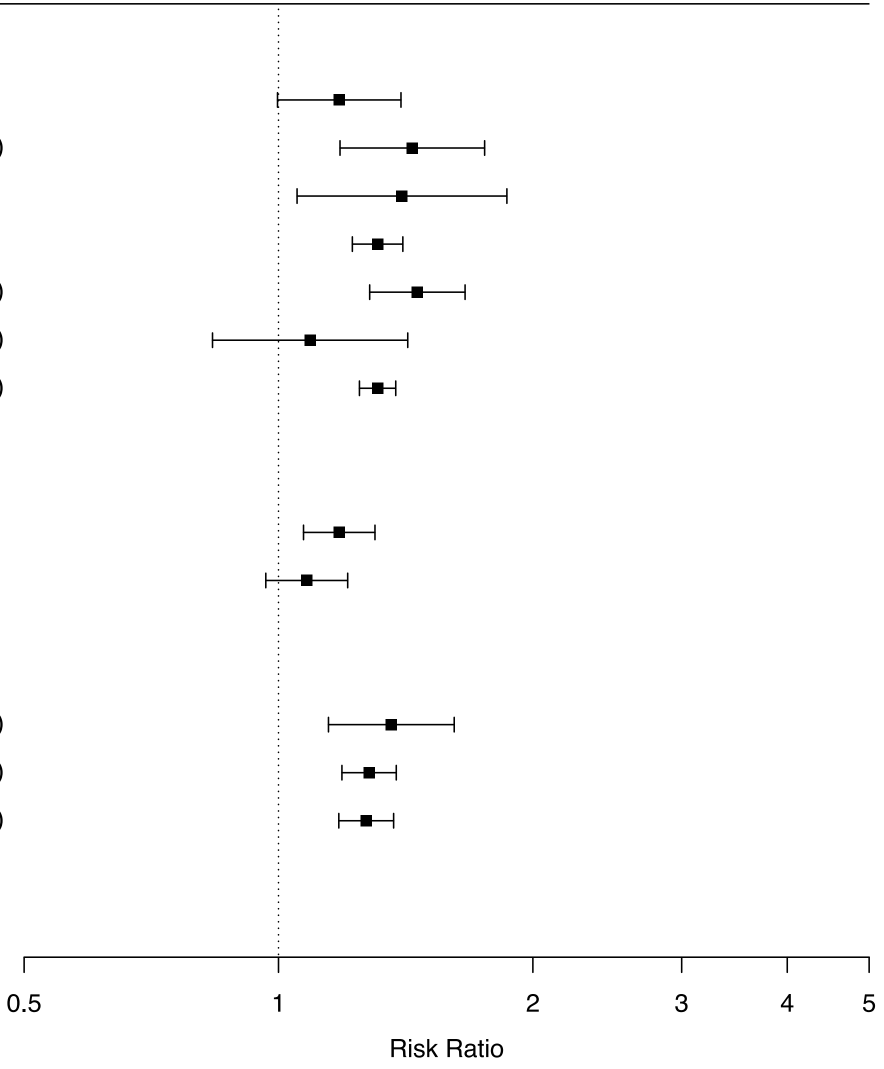

Figure S34: Trends in burden of disease (disability adjusted life years) attributable to household air pollution stratified by 2018 World Bank income groups from 2000-2017.

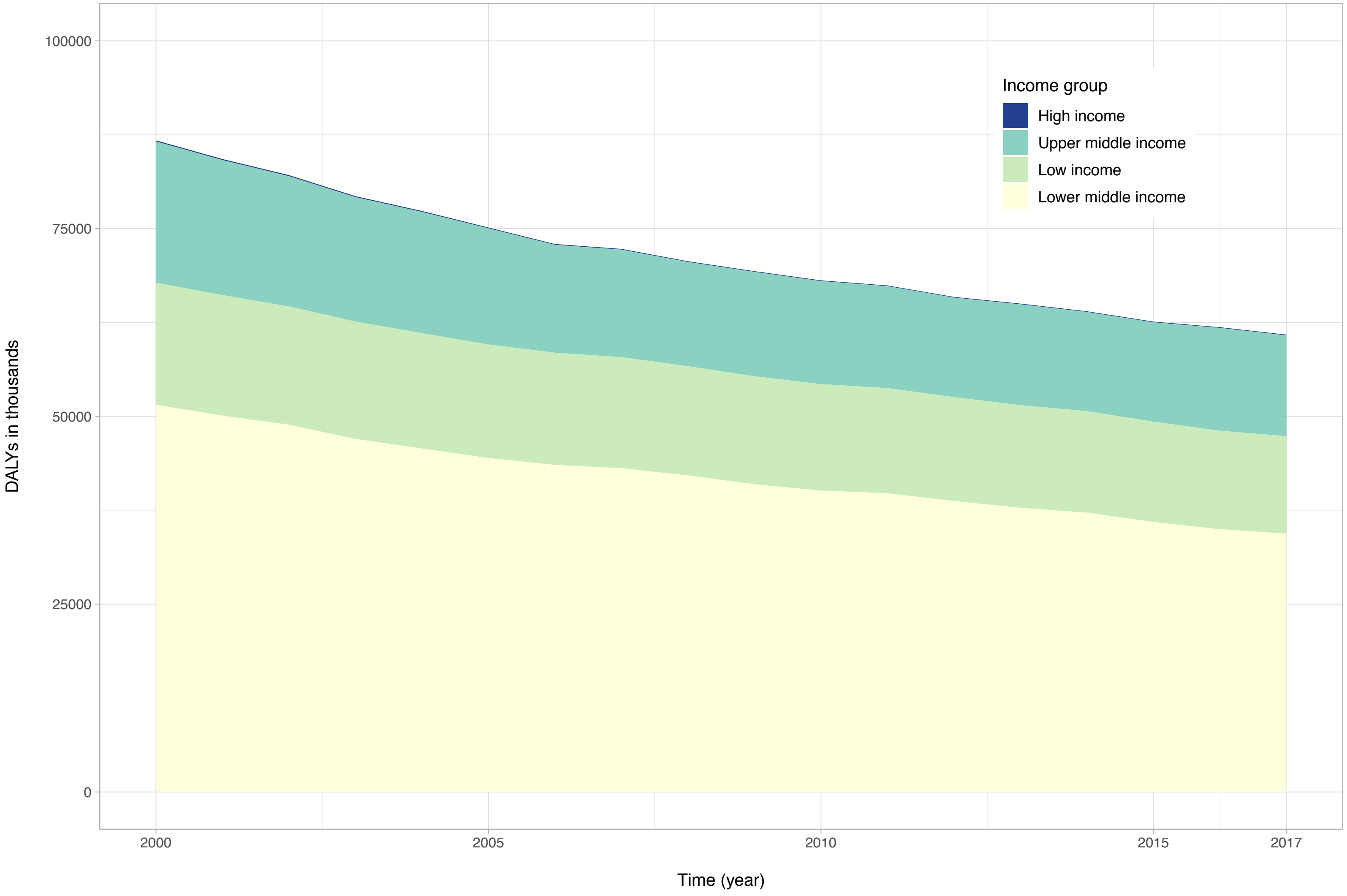

Figure S35: Cartogram of global burden of disease attributable to household air pollution in 2017.

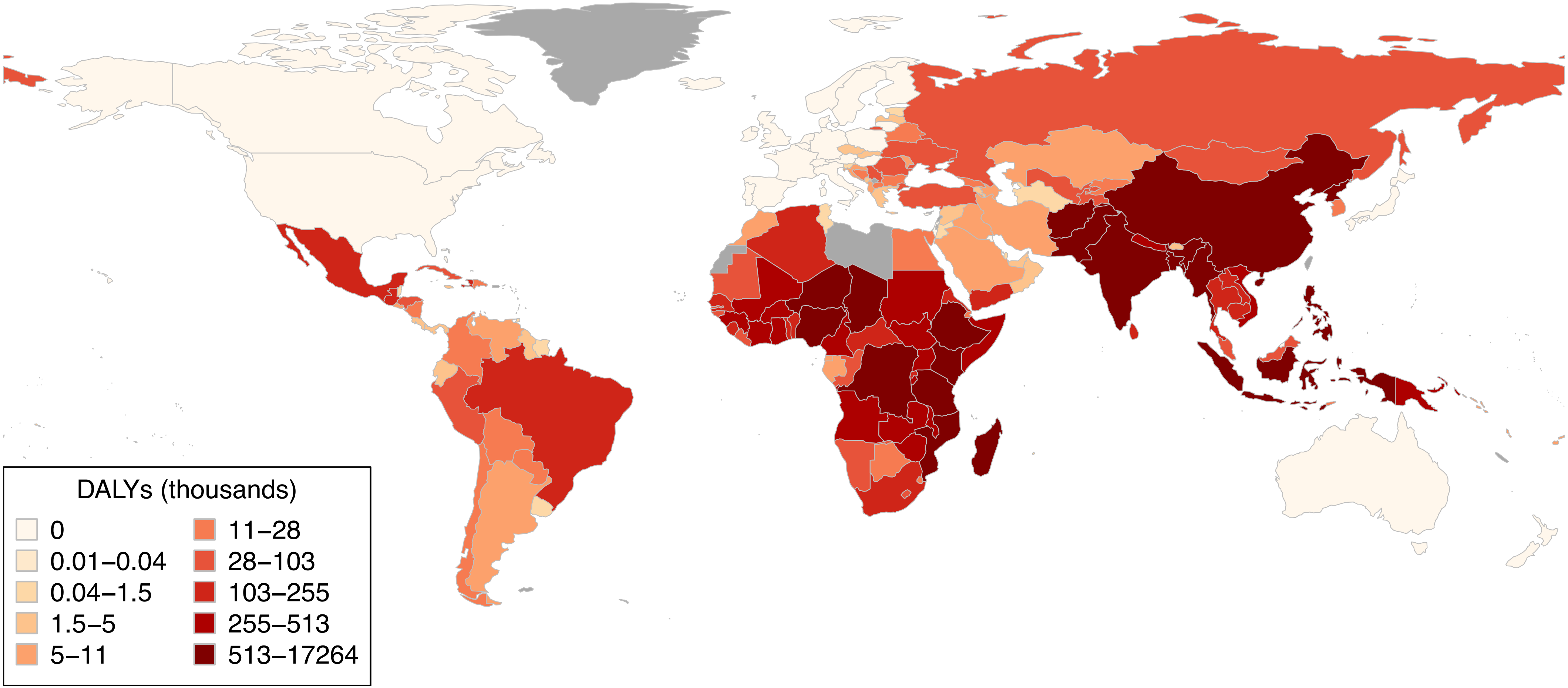

Figure S36: Trends in deaths attributable to household air pollution stratified by income

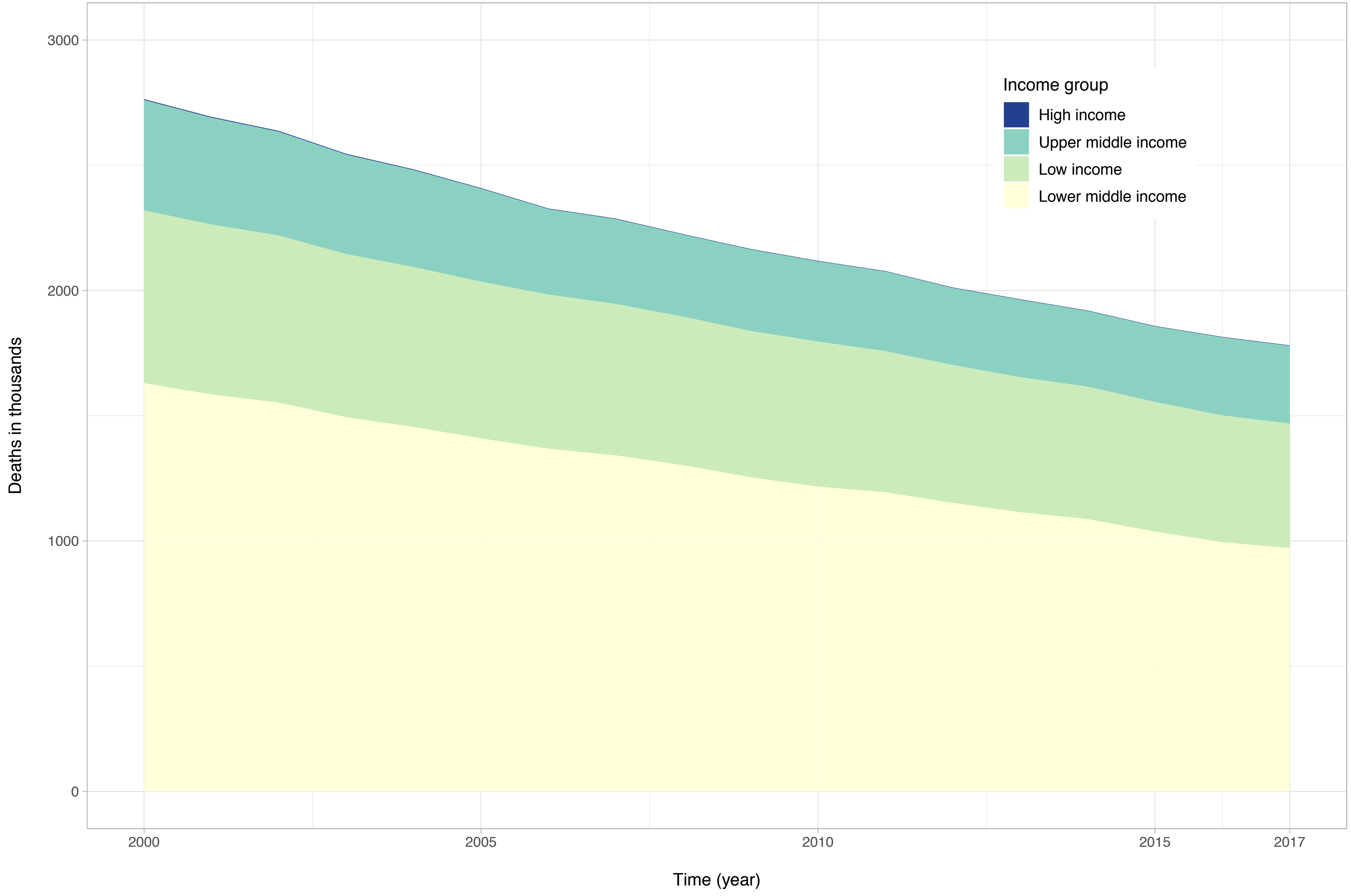

Figure S37: Trends in deaths attributable to household air pollution stratified by WHO regions

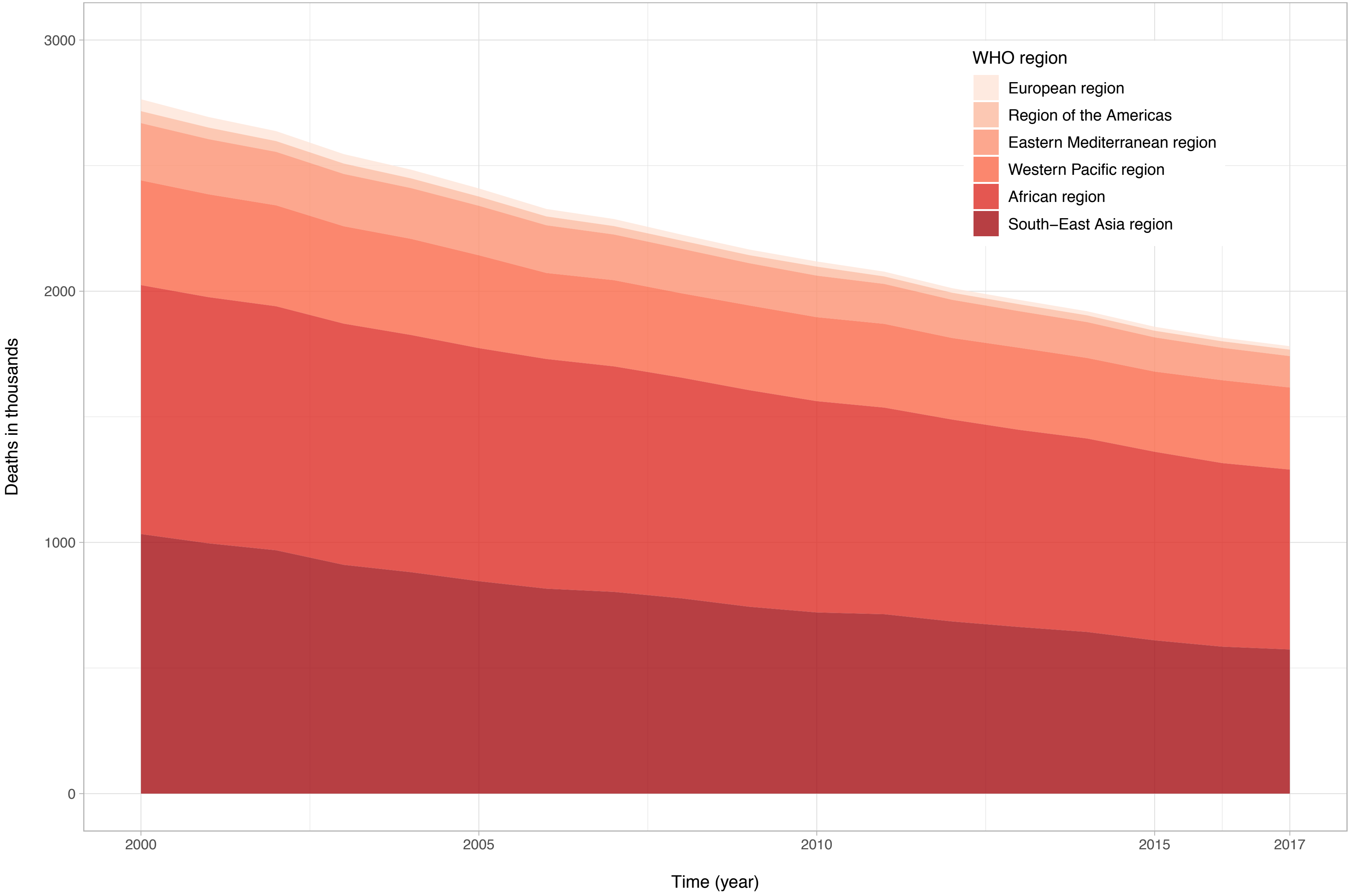

Figure S38: Trends in cardiovascular, respiratory and under five mortality attributable to household air pollution

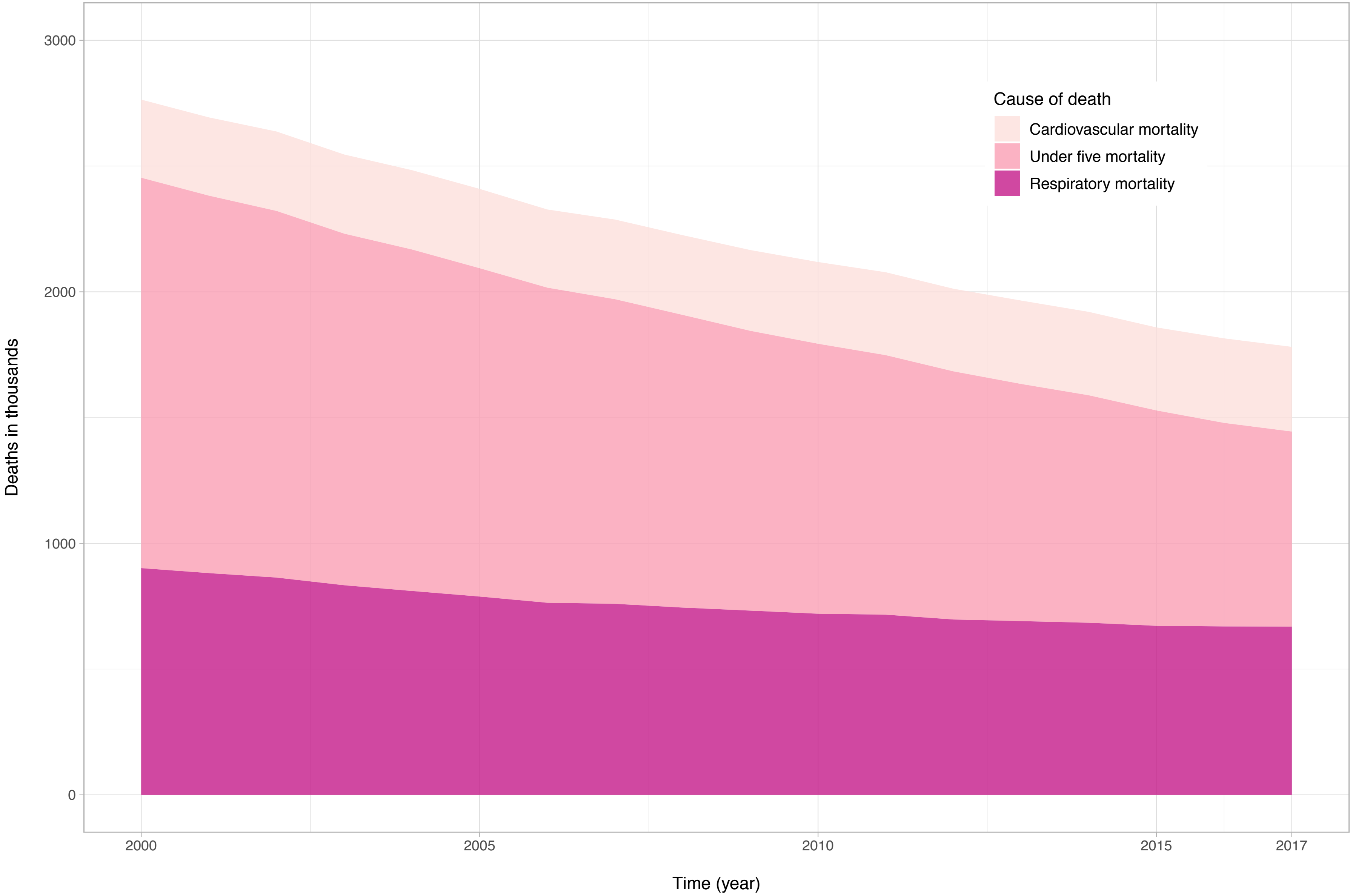

Figure S39: Trends in burden attributable to household air pollution using pooled risk ratios from studies where the comparator was clean fuel use stratified by WHO regions

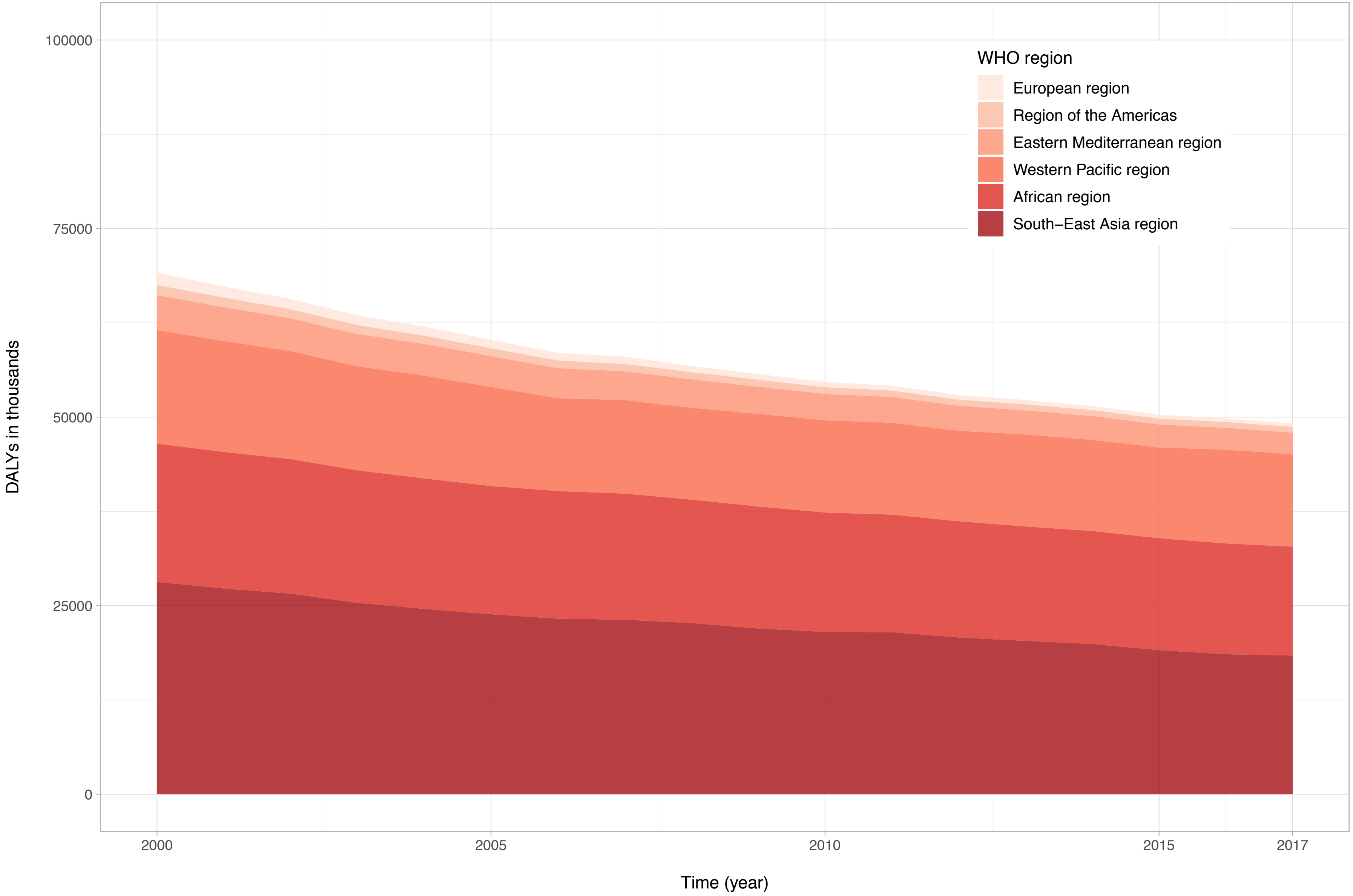

Figure S40: Trends in burden attributable to household air pollution using pooled risk ratios from studies that were assessed to be at low or moderate risk of bias stratified by WHO regions

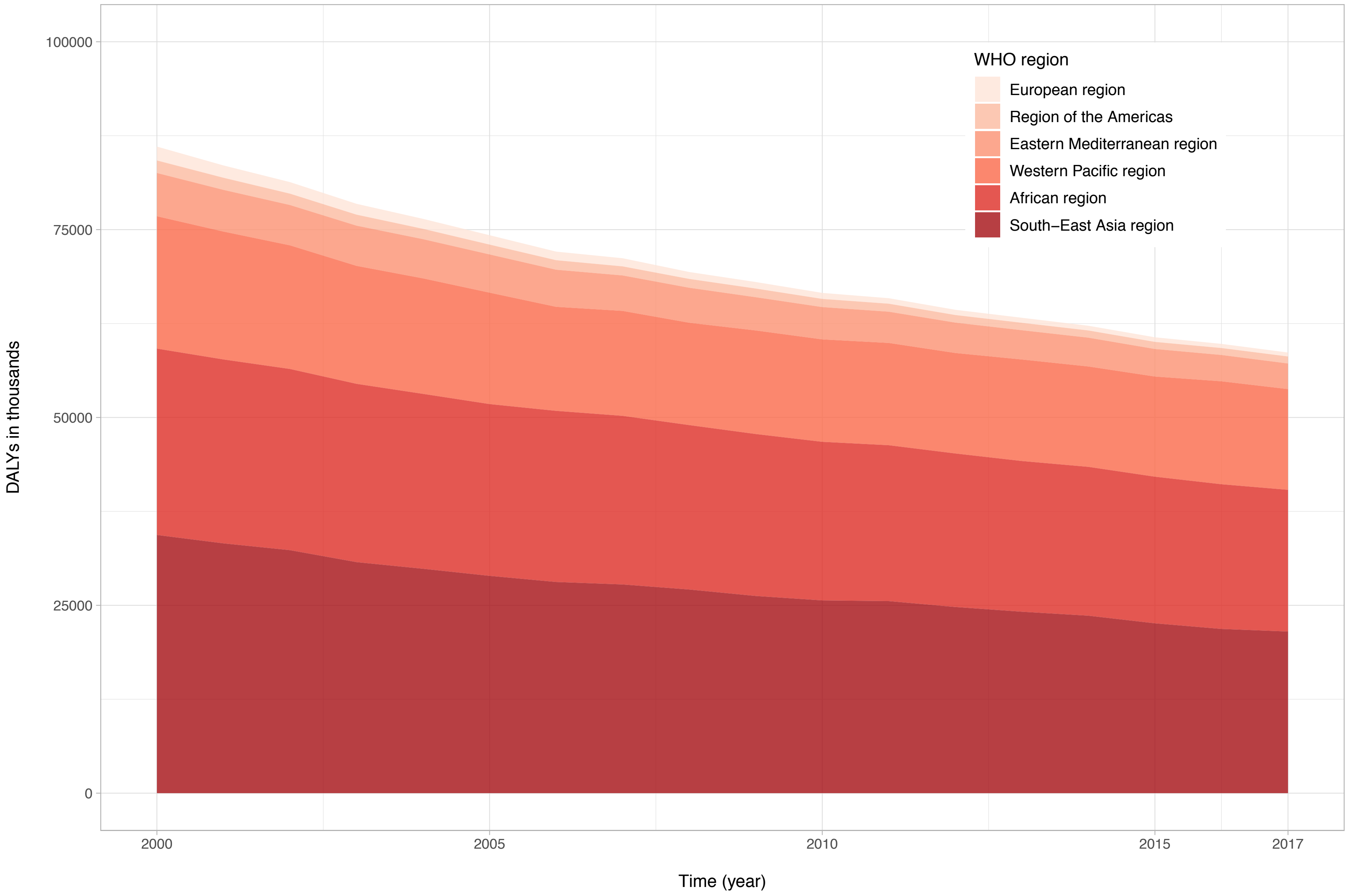

Table S1: PRISMA Checklist

Section/topic	#	Checklist item	Reported on page #
**TITLE**			
Title	1	Identify the report as a systematic review, meta-analysis, or both.	
**ABSTRACT**			
Structured summary	2	Provide a structured summary including, as applicable: background; objectives; data sources; study eligibility criteria, participants, and interventions; study appraisal and synthesis methods; results; limitations; conclusions and implications of key findings; systematic review registration number.	2
**INTRODUCTION**			
Rationale	3	Describe the rationale for the review in the context of what is already known.	3
Objectives	4	Provide an explicit statement of questions being addressed with reference to participants, interventions, comparisons, outcomes, and study design (PICOS).	3
**METHODS**			
Protocol and registration	5	Indicate if a review protocol exists, if and where it can be accessed (e.g., Web address), and, if available, provide registration information including registration number.	1
Eligibility criteria	6	Specify study characteristics (e.g., PICOS, length of follow-up) and report characteristics (e.g., years considered, language, publication status) used as criteria for eligibility, giving rationale.	Supplementary text 1
Information sources	7	Describe all information sources (e.g., databases with dates of coverage, contact with study authors to identify additional studies) in the search and date last searched.	Supplementary text 1
Search	8	Present full electronic search strategy for at least one database, including any limits used, such that it could be repeated.	Supplementary text 1
Study selection	9	State the process for selecting studies (i.e., screening, eligibility, included in systematic review, and, if applicable, included in the meta-analysis).	Supplementary text 1
Data collection process	10	Describe method of data extraction from reports (e.g., piloted forms, independently, in duplicate) and any processes for obtaining and confirming data from investigators.	Supplementary text 1
Data items	11	List and define all variables for which data were sought (e.g., PICOS, funding sources) and any assumptions and simplifications made.	4
Risk of bias in individual studies	12	Describe methods used for assessing risk of bias of individual studies (including specification of whether this was done at the study or outcome level), and how this information is to be used in any data synthesis.	Supplementary text 1

Summary measures	13	State the principal summary measures (e.g., risk ratio, difference in means).	4
Synthesis of results	14	Describe the methods of handling data and combining results of studies, if done, including measures of consistency (e.g., I^2) for each meta-analysis.	5
Risk of bias across studies	15	Specify any assessment of risk of bias that may affect the cumulative evidence (e.g., publication bias, selective reporting within studies).	5
Additional analyses	16	Describe methods of additional analyses (e.g., sensitivity or subgroup analyses, meta-regression), if done, indicating which were pre-specified.	5
**RESULTS**			
Study selection	17	Give numbers of studies screened, assessed for eligibility, and included in the review, with reasons for exclusions at each stage, ideally with a flow diagram.	6
Study characteristics	18	For each study, present characteristics for which data were extracted (e.g., study size, PICOS, follow-up period) and provide the citations.	6, Supplementary table S1
Risk of bias within studies	19	Present data on risk of bias of each study and, if available, any outcome level assessment (see item 12).	Supplementary table S1
Results of individual studies	20	For all outcomes considered (benefits or harms), present, for each study: (a) simple summary data for each intervention group (b) effect estimates and confidence intervals, ideally with a forest plot.	Data supplement
Synthesis of results	21	Present results of each meta-analysis done, including confidence intervals and measures of consistency.	Figure 1
Risk of bias across studies	22	Present results of any assessment of risk of bias across studies (see Item 15).	Data supplement
Additional analysis	23	Give results of additional analyses, if done (e.g., sensitivity or subgroup analyses, meta-regression [see Item 16]).	5-7, Data supplement
**DISCUSSION**			
Summary of evidence	24	Summarize the main findings including the strength of evidence for each main outcome; consider their relevance to key groups (e.g., healthcare providers, users, and policy makers).	10
Limitations	25	Discuss limitations at study and outcome level (e.g., risk of bias), and at review-level (e.g., incomplete retrieval of identified research, reporting bias).	10-11
Conclusions	26	Provide a general interpretation of the results in the context of other evidence, and implications for future research.	11-13
**FUNDING**			
Funding	27	Describe sources of funding for the systematic review and other support (e.g., supply of data); role of funders for the systematic review.	14

Table S2: List of countries and World Bank income group stratified by WHO regions

1) African region

Country	ISO3 code	Income group
**Benin**	**BEN**	Low income
**Burkina Faso**	BFA	Low income
**Burundi**	BDI	Low income
**Central African Republic**	CAF	Low income
**Chad**	TCD	Low income
**Comoros**	COM	Low income
**Democratic Republic of the Congo**	COD	Low income
**Eritrea**	ERI	Low income
**Ethiopia**	ETH	Low income
**Gambia**	GMB	Low income
**Guinea**	GIN	Low income
**Guinea-Bissau**	GNB	Low income
**Liberia**	LBR	Low income
**Madagascar**	MDG	Low income
**Malawi**	MWI	Low income
**Mali**	MLI	Low income
**Mozambique**	MOZ	Low income
**Niger**	NER	Low income
**Rwanda**	RWA	Low income
**Senegal**	SEN	Low income
**Sierra Leone**	SLE	Low income
**South Sudan**	SSD	Low income
**Togo**	TGO	Low income
**Uganda**	UGA	Low income
**United Republic of Tanzania**	TZA	Low income
**Zimbabwe**	ZWE	Low income
**Angola**	AGO	Lower middle income
**Cabo Verde**	CPV	Lower middle income
**Cameroon**	CMR	Lower middle income
**Congo**	COG	Lower middle income
**Cote d'Ivoire**	CIV	Lower middle income
**Ghana**	GHA	Lower middle income
**Kenya**	KEN	Lower middle income
**Lesotho**	LSO	Lower middle income
**Mauritania**	MRT	Lower middle income
**Nigeria**	NGA	Lower middle income
**Swaziland**	SWZ	Lower middle income
**Zambia**	ZMB	Lower middle income
**Algeria**	DZA	Upper middle income
**Botswana**	BWA	Upper middle income

**Equatorial Guinea**	GNQ	Upper middle income
**Gabon**	GAB	Upper middle income
**Mauritius**	MUS	Upper middle income
**Namibia**	NAM	Upper middle income
**South Africa**	ZAF	Upper middle income
**Seychelles**	SYC	High income

2) Region of the Americas

Country	ISO3 code	Income group
**Haiti**	HTI	Low income
**Bolivia (Plurinational State of)**	BOL	Lower middle income
**El Salvador**	SLV	Lower middle income
**Honduras**	HND	Lower middle income
**Nicaragua**	NIC	Lower middle income
**Belize**	BLZ	Upper middle income
**Brazil**	BRA	Upper middle income
**Colombia**	COL	Upper middle income
**Costa Rica**	CRI	Upper middle income
**Cuba**	CUB	Upper middle income
**Dominica**	DMA	Upper middle income
**Dominican Republic**	DOM	Upper middle income
**Ecuador**	ECU	Upper middle income
**Grenada**	GRD	Upper middle income
**Guatemala**	GTM	Upper middle income
**Guyana**	GUY	Upper middle income
**Jamaica**	JAM	Upper middle income
**Mexico**	MEX	Upper middle income
**Paraguay**	PRY	Upper middle income
**Peru**	PER	Upper middle income
**Saint Lucia**	LCA	Upper middle income
**Saint Vincent and the Grenadines**	VCT	Upper middle income
**Suriname**	SUR	Upper middle income
**Venezuela (Bolivarian Republic of)**	VEN	Upper middle income
**Antigua and Barbuda**	ATG	High income
**Argentina**	ARG	High income
**Barbados**	BRB	High income
**Chile**	CHL	High income
**Panama**	PAN	High income
**Trinidad and Tobago**	TTO	High income
**Uruguay**	URY	High income
**Bahamas**	BHS	High income
**Canada**	CAN	High income
**United States of America**	USA	High income

3) South-East Asia Region

Country	ISO3 code	Income group
**Democratic People's Republic of Korea**	PRK	Low income
**Nepal**	NPL	Low income
**Bangladesh**	BGD	Lower middle income
**Bhutan**	BTN	Lower middle income
**India**	IND	Lower middle income
**Indonesia**	IDN	Lower middle income
**Myanmar**	MMR	Lower middle income
**Sri Lanka**	LKA	Lower middle income
**Timor-Leste**	TLS	Lower middle income
**Maldives**	MDV	Upper middle income
**Thailand**	THA	Upper middle income

4) European Region

Country	ISO3 code	Income group
**Tajikistan**	TJK	Low income
**Georgia**	GEO	Lower middle income
**Kyrgyzstan**	KGZ	Lower middle income
**Republic of Moldova**	MDA	Lower middle income
**Ukraine**	UKR	Lower middle income
**Uzbekistan**	UZB	Lower middle income
**Albania**	ALB	Upper middle income
**Armenia**	ARM	Upper middle income
**Azerbaijan**	AZE	Upper middle income
**Belarus**	BLR	Upper middle income
**Bosnia and Herzegovina**	BIH	Upper middle income
**Bulgaria**	BGR	Upper middle income
**Kazakhstan**	KAZ	Upper middle income
**Montenegro**	MNE	Upper middle income
**Romania**	ROU	Upper middle income
**Russian Federation**	RUS	Upper middle income
**Serbia**	SRB	Upper middle income
**The former Yugoslav Republic of Macedonia**	MKD	Upper middle income
**Turkey**	TUR	Upper middle income
**Turkmenistan**	TKM	Upper middle income
**Croatia**	HRV	High income
**Czechia**	CZE	High income
**Estonia**	EST	High income
**Greece**	GRC	High income
**Latvia**	LVA	High income
**Slovakia**	SVK	High income

**Slovenia**	SVN	High income
**Andorra**	AND	High income
**Austria**	AUT	High income
**Belgium**	BEL	High income
**Cyprus**	CYP	High income
**Denmark**	DNK	High income
**Finland**	FIN	High income
**France**	FRA	High income
**Germany**	DEU	High income
**Hungary**	HUN	High income
**Iceland**	ISL	High income
**Ireland**	IRL	High income
**Israel**	ISR	High income
**Italy**	ITA	High income
**Lithuania**	LTU	High income
**Luxembourg**	LUX	High income
**Malta**	MLT	High income
**Netherlands**	NLD	High income
**Norway**	NOR	High income
**Poland**	POL	High income
**Portugal**	PRT	High income
**Spain**	ESP	High income
**Sweden**	SWE	High income
**Switzerland**	CHE	High income
**United Kingdom**	GBR	High income

5) Eastern Mediterranean Region

Country	ISO3 code	Income group
**Afghanistan**	AFG	Low income
**Somalia**	SOM	Low income
**Syrian Arab Republic**	SYR	Low income
**Yemen**	YEM	Low income
**Djibouti**	DJI	Lower middle income
**Egypt**	EGY	Lower middle income
**Morocco**	MAR	Lower middle income
**Pakistan**	PAK	Lower middle income
**Sudan**	SDN	Lower middle income
**Tunisia**	TUN	Lower middle income
**Iran (Islamic Republic of)**	IRN	Upper middle income
**Iraq**	IRQ	Upper middle income
**Jordan**	JOR	Upper middle income
**Oman**	OMN	High income
**Qatar**	QAT	High income
**Saudi Arabia**	SAU	High income

**United Arab Emirates**	ARE	High income
**Bahrain**	BHR	High income
**Kuwait**	KWT	High income

6) Western Pacific Region

Country	ISO3 code	Income group
**Cambodia**	KHM	Lower middle income
**Kiribati**	KIR	Lower middle income
**Lao People's Democratic Republic**	LAO	Lower middle income
**Micronesia (Federated States of)**	FSM	Lower middle income
**Mongolia**	MNG	Lower middle income
**Papua New Guinea**	PNG	Lower middle income
**Philippines**	PHL	Lower middle income
**Solomon Islands**	SLB	Lower middle income
**Vanuatu**	VUT	Lower middle income
**Viet Nam**	VNM	Lower middle income
**China**	CHN	Upper middle income
**Fiji**	FJI	Upper middle income
**Malaysia**	MYS	Upper middle income
**Marshall Islands**	MHL	Upper middle income
**Samoa**	WSM	Upper middle income
**Tonga**	TON	Upper middle income
**Republic of Korea**	KOR	High income
**Australia**	AUS	High income
**Brunei Darussalam**	BRN	High income
**Japan**	JPN	High income
**New Zealand**	NZL	High income
**Singapore**	SGP	High income

Table S3: Baseline details of included studies

Abbreviations: LPG= liquid petroleum gas, COPD= chronic obstructive pulmonary disease, NA= not applicable.

* Mean age or range of ages are in years, unless otherwise stated

Author	Publication year	Country	Study design	Sample size	Men, %	Age*	Fuel type in exposed group	Fuel type in control group	Outcomes assessed	Risk of bias
**Abusalah et al[2]**	2012	Palestine	case-control	446	0	26.8	wood	not exposed to wood	low birth weight	low
**Acharya et al[3]**	2015	Nepal	case-control	4802	52.1	under 5	solid fuel (wood, animal dung, straw, shrubs, grass, crop residue, coal, lignite and charcoal)	cleaner fuel	acute respiratory infection	high
**Adane et al[4]**	2020	Ethiopia	cross-sectional	5830	51.7	under 5	biomass fuel	biomass fuel	acute lower respiratory infection	moderate
**Addo-Yobo et al[5]**	2001	Ghana	case-control	96	50	13.3	electricity	charcoal, firewood, liquefied petroleum gas, and kerosene	asthma	high
**Adesanya et al[6]**	2016	Nigeria	case-control	28596	50.1	under 5	biomass fuel	kerosene/charcoal	acute respiratory infection	high
**Adeyemi et al[7]**	2016	Nigeria	case-control	27983	0	27.3	firewood/dung	LPG/kerosene	low birth weight	moderate
**Admasie et al[8]**	2018	Ethiopia	case-control	1144	52.97	under 5	unclean fuel	clean fuel	acute respiratory infection	high
**Admasie et al[8]**	2018	Ethiopia	cross-sectional study	1042	54.4	2.0	biomass/charcoal	electricity	low birth weight	moderate
**Aggarwal et al[9]**	2006	India	case-control	73605	52	not reported	LPG, kerosene, solid fuel	no self cooking	asthma	high
**Agrawal et al[10]**	2012	India	retrospective cohort study	156316	36.3	20-49	biomass fuel, solid fuel	cleaner fuel	asthma	high
**Akhtar et al[11]**	2007	Pakistan	case-control	74918	50.8	31.3	wood, dung cake, rice straw, kai grass	LPG	chronic bronchitis	high
**Akinyemi et al[12]**	2016	15 sub-Saharan African countries (Burkino Faso, Burundi, Comoros, Congo (Brazzaville), Congo Democratic Republic, Cote d'Ivoire, Gabon, Lesotho, Madagascar, Mozambique, Namibia, Niger, Rwanda, Sierra Leone, Zambia)	case-control	143602	50.4	under 5	solid fuel (coal, lignite, charcoal, wood, straw/shrubs/grass, agricultural crop and animal dung)	non-solid fuel (electricity, LPG, natural gas, biogas, kerosene)	infant mortality and child mortality	high
**Akinyemi et al[13]**	2018	Nigeria	cross-sectional	59370	50.3	under 5	unclean	clean	Acute respiratory infection	high

**Alam et al[14]**	2012	Bangladesh	retrospective cohort study	946	56.2	64.9	solid fuel	LPG	cardiovascular mortality, ischaemic heart disease mortality, stroke mortality, respiratory mortality	high
**Alam et al[15]**	2015	Bangladesh	cross-sectional	3759	46	54.0	sold fuel	LPG	COPD	low
**Aldous et al[16]**	1996	USA	prospective cohort study	936	NA	under 1	LPG	electricity	acute respiratory infection	high
**Alemayehu et al[17]**	2014	Ethiopia	cross-sectional	715	51.3	2.4	High/medium pollution fuel	Low pollution fuel	acute respiratory infection	high
**Alemayehu et al[18]**	2019	Ethiopia	case-control	288	55.5	under 5	cow dung	no cow dung	Acute respiratory infection	high
**Alexander et al[19]**	2018	Nigeria	Randomised-controlled trial	324	0	28.0	ethanol cook stove	firewood or kerosene stove	stillbirth and birth weight	high
**Alim et al[20]**	2014	Bangladesh	cross-sectional	420	0	not reported	biomass fuel	gas	any respiratory involvement	high
**Alvis-Guzman et al[21]**	2013	Colombia	cross-sectional	203	25.4	not reported	biomass fuel	LPG	COPD	low
**Amegah et al[22]**	2012	Ghana	cross-sectional	592	NA	not reported	charcoal	LPG	low birth weight	low
**Anteneh et al[23]**	2020	Ethiopia	cross-sectional	10006	51	0-59 months	wood, straw, agricultural products, kerosene, charcoal, animal dung,	electricity, gas	acute respiratory infection	high
**Arinola et al[24]**	2018	Nigeria	cross-sectional study	68	0	30.2	kerosene	liquefied natural gas	low birth weight	high
**Arlington et al[25]**	2019	India	prospective cohort study	1586	50.76	0-6 months	wood, straw, shrubs, grass, agricultural crop, animal dung, coal or charcoal	iquid petroleum gas or electricity	acute lower respiratory infections	high
**Aung et al[26]**	2013	Myanmar	case-control	358	65.3	6.7	smoke producing fuel	no smoke producing fuel	acute respiratory infection	moderate
**Awasthi et al[27]**	1996	India	cohort study	664	56	under 4.5	coal, wood, kerosene, dung	LPG	Respiratory symptom complex	high
**Azad et al[28]**	2014	Bangladesh	cross-sectional	145	61.8	7.0	wood	no wood	acute respiratory infection	high
**Azizi et al[29]**	1991	Malaysia	cross-sectional	1501	57.3	9.0	wood/kerosene stove	electric/gas stove	Asthma, persistent wheeze	high
**Azizi et al[30]**	1995	Malaysia	case-control	359	57.4	3.0	kerosene, wood	no kerosene or wood	asthma	high
**Baker et al[31]**	2006	Czech Republic	cohort study	452	55.3	1.5	coal use for heating	Distant heat + other	Lower Respiratory Illness	low
**Balakrishnan et al[32]**	2018	India	cohort study	1125	0	0.0	solid fuel	LPG	birth weight	low
**Barone-Adesi et al[33]**	2012	China	retrospective cohort study	37272	52.7	56.7	smoky coal (bituminous)	smokeless coal (anthracite)	lung cancer	low
**Barria et al[34]**	2008	Chile	cohort study	316	50.6	0.0	wood	Not exclusively using wood	acute lower respiratory illness	high
**Barry et al[35]**	2010	USA	cohort study	508	59.4	57.0	wood/coal	Not using wood/coal for cooking/heating	asthma	high
**Bassani et al[36]**	2010	India	case-control	482763	52	under 4	solid fuel	Non-solid fuels	death	moderate

**Bassig et al[37]**	2019	China	prospective cohort study	16323	0	39.4	smokeless coal users	smoky coal users	mortality from ischaemic heart disease or stroke	moderate
**Bates et al[38]**	2013	Nepal	case-control	917	56.7	1.5	kerosene, biomass, gas, wood or coal	Electricity, gas or none	acute lower respiratory illness	high
**Bates et al[39]**	2018	Nepal	case-control	824	56	1.5	PM2.5 concentration	NA	acute lower respiratory illness	high
**Bates et al[39]**	2018	Nepal	case-control	824	55.9	18-70	gas/kerosene/biomass. Heating with wood/kerosene/coal/electric/gas	electricity, no heating	acute lower respiratory infection	high
**Bates et al[40]**	2019	Nepal	case-control	1807	53.5	0-36months	biogas, wood stove, kerosene, heating with solid fuels	lpg,no kerosene, no heating	active pulmonary tuberculosis	moderate
**Bautista et al[41]**	2009	Dominican Republic	cohort study	415	52.3	under 1.5	Charcoal	Propane	ALRI	high
**Behera et al[42]**	2005	India	case-control	113	0	49.4	biomass fuel	LPG	lung cancer	moderate
**Behera et al[43]**	2010	India	case-control	204	0	35.6	Biomass, kerosene	LPG	tuberculosis	moderate
**Behrens et al[44]**	2005	Germany	cross-sectional	6996	51.7	6-7	gas,coal, oil, wood	no gas, no coal, no oil, no wood	wheezing and atopic symptoms	high
**Belanger et al[45]**	2003	USA	prospective cohort study	849	48.3	under 1	wood stove, gas stove, NO2 concentration	no exposure to wood stove, gas stove	respiratory symptoms	high
**Belanger et al[46]**	2006	USA	case-control	728	62.4	under 12	gas stove and NO2 concentration	no gas stove	respiratory symptoms in children with asthma (wheeze, persistent cough, shortness of breath, chest tightness)	high
**Belanger et al[47]**	2013	USA	prospective cohort study	1342	59	5-10	NO2 concentration	NA	asthma	high
**Bhat et al[48]**	2012	india	case-control	202	52.5	under 5	fuel other than LPG	LPG	acute respiratory infection	high
**Bhat et al[49]**	2013	India	case-control	214	55.6	under 5	fuel other than LPG	LPG	acute respiratory infection	moderate
**Boadi et al[50]**	2006	Ghana	cross-sectional	948	0	32.4	charcoal, firewood, kerosene, LPG	LPG, electricity	respiratory infection	high
**Bothwell et al[51]**	2003	Northern Ireland	cross-sectional	2578	NA	under 12	glass-fronted solid fuel fire	no glass-fronted solid fuel heating	asthma and asthma symptoms (wheeze, cough and inhaler use)	high
**Boy et al[52]**	2002	Guatemala	prospective cohort study	1717	0	25.0	wood	electricity, LPG	birth weight	low
**Broor et al[53]**	2001	India	case-control	512	70	under 5	cooking fuel other than LPG	LPG	severe acute lower respiratory infection	high
**Bruce et al[54]**	1998	Guatemala	cross-sectional	321	0	28.0	plancha	open fire	respiratory symptoms	high
**Buchner et al[55]**	2015	Countries in Sub-Saharan Africa (Benin, Burkino Faso, Cameroon, Ethiopia, Ghana, Guinea, Kenya, Madagascar, Mali, Malawi, Mozambique, Namibia, Niger, Senegal, Tanzania, Uganda, Zambia, Zimbabwe)	cross-sectional	56437	51	under 5	kerosene, wood, coal, lowergrade biomass (straw, dung, grass, shrubs, crop residues)	clean fuel (electricity, LPG, gas, biogas, no food cooked in the house)	acute respiratory infection	high
**Budge et al[56]**	2014	Peru	prospective cohort study	892	NA	under 3	improved, vented stove	open indoor fire for cooking	acute respiratory infection	moderate

**Budhathoki et al[57]**	2020	Nepal	cross-sectional	15372	NA	0-59 months	kerosene, wood, straw, shrubs, grass, animal dung, coal,charcoal	electricity, LPG (liquefied petroleum gas), biogas, natural gas	pneumonia	high
**Bueso et al[58]**	2010	Honduras, El Salvador	cross-sectional	1827	52.215	1.1	gas, kerosene, coal, firewood, other, various	electicity	wheeze	high
**Bulkow et al[59]**	2012	USA	case-control	314	52	under 3	woodstove	does not use woodstove	acute respiratory infection	high
**Burr et al[60]**	1999	United Kingdom	cross-sectional	25393	NA	12-14	gas, solid fuel	electricity only	respiratory symptoms (wheeze, speech limiting wheeze, wheeze without colds, rhinitis, spring/summer rhinitis, cough without colds, phlegm with colds, phlegm without colds, >3 colds per year, dry night cough without colds)	high
**Buthelezi et al[61]**	2019	South Africa	cross-sectional	245	39	not reported	coal, wood, gas, paraffin	electric energy source	respiratory disease and symptoms	high
**Butland et al[62]**	1997	United Kingdom	case-control	749	NA	7.5-8.5	LPG	no LPG	asthma symptoms (wheeze)	high
**Cardoso et al[63]**	2013	Brazil	case-control	321	NA	under 5	wood	no domestic heating	acute respiratory infection	high
**Cardoso et al[64]**	2015	Brazil	cross-sectional	6081	51.4	under 5	wood, coal, other	none / LPG	acute respiratory infection	high
**Carlsten et al[65]**	2011	Canada	case-control	545	56	7.0	gas stove	not exposed to gas stove	asthma	high
**Casas et al[66]**	2012	Germany	prospective cohort study	5078	51	under 10	gas	no gas	asthma	high
**Cerqueiro et al[67]**	1990	Argentina	case-control	1338	57.4	under 5	bottled gas, any fuel, charcoal	no exposure to bottled gas, any fuel, charcoal	lower respiratory infection	low
**Cetinkaya et al[68]**	2000	Turkey	cross-sectional	1023	45.1	not reported	biomass fuel	no biomass fuel	chronic bronchitis	high
**Chan et al[69]**	2019	China	prospective cohort study	277838	9	50.3	coal, wood, charcoal	electricity, gas	respiratory diseases and death from any respiratory disease	low
**Chen et al[70]**	1990	Taiwan	case-control	931	79.1	not reported	wood and coal	charcoal, LPG, electricity	lung cancer	moderate
**Chen et al[71]**	2018	Taiwan	cross-sectional	644	0	63.2	biomass fuel, coal	gas/electric stove	chronic bronchitis and COPD	low
**Chen et al[71]**	2018	China	cross-sectional	1940	55	0-18	coal, wood, charcoal	electricity, kerosene, liquefied or natural gas	acute respiratory infection in the past four weeks	high
**Choi et al[72]**	2015	India	cross-sectional	1392	NA	not reported	kerosene	LPG	bronchitis	high
**Clark et al[73]**	2009	Honduras	cross-sectional	79	0	41.5	traditional stove and PM2.5 and CO concentration	improved Justa stoves	respiratory symptoms (cough, phlegm, shortness of breath)	high
**Coggon et al[74]**	1993	UK	retrospective cohort study	8812	NA	not reported	gas cooker	no gas cooker	mortality	moderate
**Coker et al[75]**	2015	USA	cross-sectional	3240	NA	under 5	gas stove	no gas stove for heating	acute respiratory infection	high

**Collings et al[76]**	1990	Zimbabwe	cross-sectional	744	53.9	10 months	open fire	kerosene, gas, electric stoves	lower respiratory tract infection	high
**Comstock et al[77]**	1981	USA	cross-sectional	1724	73.6	not reported	LPG	Electricity	respiratory symptoms (chronic cough, chronic phlegm, wheeze, shortness of breath, chest illness)	high
**Crampin et al[78]**	2004	Malawi	case-control	1590	46	not reported	wood	no wood	tuberculosis	moderate
**da Silva et al[79]**	2012	Brazil	cross-sectional	1402	41.2	27.8	biomass fuel	LPG	respiratory symptoms	high
**Dagoye et al[80]**	2004	Ethiopia	cross-sectional	7155	49	3.0	kerosene	no kerosene	wheeze	high
**Dai et al[81]**	1996	China	case-control	120	0	30-69	coal	no coal stove in bedroom	lung adenocarcinoma	moderate
**Daigler et al[82]**	1991	USA	case-control	508	55.3	3.4	woodburning stove	no woodburning stove	symptoms	high
**Das et al[83]**	2017	Malawi	cross-sectional	655	0	37.3	firewood or crop residue, traditional stove	charcoal/improved stove	cardiorespiratory symptoms (shortness of breath walking uphill, shortness of breath at rest, difficulty breathing/chest tightness, chest ains/palpitations, persistent phlegm, persistent cough)	high
**de Oca et al[84]**	2017	Argentina, Colombia, Venezuela, and Uruguay	cross-sectional	1743	49	not reported	Coal or wood	NA	COPD	high
**Dekker et al[85]**	1991	Canada	cross-sectional	14059	NA	5-8	gas stove, gas and oil heating system	no gas stove	asthma	high
**Dennis et al[86]**	1996	Columbia	case-control	208	0	62.5	woodsmoke, gasoline	not exposed to woodsmoke	COPD	low
**Desalu et al[87]**	2010	Nigeria	cross-sectional	269	0	55.0	biomass fuel	non-biomass fuel	respiratory symptoms (cough, chest pain, shortness of breath and wheeze) and chronic bonchitis	high
**Devien et al[88]**	2018	France	cross-sectional	3039	47	53.2	gas stove, open fireplace, coal	no gas stove, open fireplace, coal	respiratory symptoms (wheeze and chronic cough)	high
**Dhanaraj et al[89]**	2015	India	cross-sectional	59957	50.5	not reported	solid fuel	smokeless fuel	pulmonary tuberculosis	low
**Dharmage et al[90]**	1996	Sri Lanka	case-control	200	NA	under 5	gas, kerosene	not exposed to gas and kerosene oil	acute lower respiratory infection	moderate
**Diette et al[91]**	2007	USA	case-control	600	50	4.4	gas	electricity	asthma	high
**Ding et al[92]**	2009	China	case-control	140	69.29	6.8	coal	no exposure to coal	asthma	moderate
**Ding et al[93]**	2012	China	case-control	1737	NA	not reported	straw or wood	No exposure to straw or wood	asthma	high
**Ding et al[94]**	2015	China	case-control	584	48.1	63.3	biomass fuel	modern fuel	COPD	moderate
**Dockery et al[95]**	1989	USA	cross-sectional	5338	NA	7-11	gas, kerosene, wood	no gas, kerosene, wood	respiratory symotoms	high

**Dodge et al**[96]	1982	USA	cross-sectional	419	NA	not reported	gas	electricity	asthma, wheeze and cough	high
**Dohoo et al**[97]	2012	Kenya	cross-sectional	62	0	45.0	no biogas	bio gas	breathing problems	high
**Dong et al**[98]	2008	China	cross-sectional	3945	50.1	4.9	coal	no coal	asthma	high
**Dong et al**[99]	2008	China	cross-sectional	10784	50.4	9.9	coal	no exposure to coal	asthma, respiratory symptoms	high
**Dong et al**[100]	2013	China	cross-sectional	30780	0	23-49	coal	no coal	asthma	high
**Dossing et al**[101]	1994	Saudia Arabia	case-control	121	46	56.9	open indoor fire	no open indoor fire	COPD	high
**Dow et al**[102]	1999	UK	cross-sectional	4792	NA	over 65	gas appliances	no exposure to gas appliances	respiratory symptoms	high
**Du et al**[103]	1996	China	retrospective cohort study	5546	67.8	64.3	coal	no coal	lung cancer	moderate
**Dutt et al**[104]	1996	India	prospective cohort study	295	0	35.0	biofuel and kerosene	gas	bronchitis and cough	high
**Dutta et al**[105]	2013	India	cross-sectional	480	0	34.0	biomass fuel	LPG	COPD	moderate
**Ehrlich et al**[106]	2004	South Africa	cross-sectional	13826	41	over 15	wood, coal, dung	electricity, paraffin, gas	chronic bronchitis	high
**Eisner et al**[107]	2002	USA	prospective cohort study	349	28	44.2	gas stove, woodsmoke	not exposed to gas or woodsmoke	asthma	high
**Eisner et al**[108]	2003	USA	cross-sectional	445	34	42.3	gas stove	not exposed to gas stove	asthma	high
**Ekici et al**[109]	2005	Turkey	case-control	596	0	51.1	biomass fuel	LPG	COPD	high
**Elf et al**[110]	2017	South Africa	cross-sectional	53	NA	not reported	kerosene, wood	electricity	tuberculosis	high
**Elf et al**[111]	2019	India	case-control	192	NA	not reported	kerosene, wood	no kerosene, no wood	tuberculosis	high
**Emenius et al**[112]	2003	Sweden	case-control	540	53	under 2	NO2 concentration and gas stove	no gas stove	respiratory symptoms (recurrent wheeze)	high
**Emuron et al**[113]	2019	Peru, Chile, Argentina, Uruguay, Bangladesh, Uganda	cross-sectional	12592	51.4	54.6	solid fuel	clean fuel	pulmonary tuberculosis	high
**Epstein et al**[114]	2013	India	cross-sectional	36529	0	not reported	kerosene, coal/coal lignite, and biomass fuels (wood, agricultural crop waste, straw/shrubs/grass, dung cakes, and charcoal)	LPG, electricity and biogas	low birth weight, neonatal death	high
**Esplugues et al**[115]	2011	Spain	cohort study	352	54	under 1	NO2	NA	lower respiratory tract infection, wheezing, persistent cough	moderate
**Esplugues et al**[116]	2013	Spain	prospective cohort study	2003	NA	under 1	gas cooker	no gas cooker	LRTI, wheezing, persistent cough, chestiness	high
**Etiler et al**[117]	2002	Turkey	prospective cohort study	204	50.1	under 1	biomass fuel	central heating	acute respiratory infection	high
**Ezeh et al**[118]	2014	Nigeria	cross-sectional	30726	NA	under 5	solid fuel	non-solid fuel	neonatal mortality, post-neonatal mortality, child mortality	high
**Fagbule et al**[119]	1994	Nigeria	case-control	280	NA	5.5	biomass smoke	no biomass smoke	asthma	high
**Faizan et al**[120]	2019	India	cross-sectional	54985	51.47	not reported	solid fuel	clean fuel	tb, auri, copd, bronchial asthma	high

**Fakunle et al[121]**	2014	Nigeria	case-control	440	55.5	under 5	firewood	no firewood	acute respiratory infection	moderate
**Farrow et al[122]**	1997	United Kingdom	cross-sectional	921	NA	3 to 12 months	NO2 concentration	NA	respiratory symptoms (cough, breathlessness, wheeze)	high
**Fatmi et al[123]**	2014	Pakistan	case-control	146	0	56.3	solid fuel	LPG	acute coronary syndrome	low
**Fatmi et al[124]**	2019	Pakistan	cross-sectional	850	0	not reported	biomass fuel	no biomass fuel	Hypertension, angina, heart attack, definite or probable CHD on ECG	high
**Fedortsiv et al[125]**	2012	Ukraine	cross-sectional	4871	49.2	41791.0	coal or wood stove	central heating	asthma and respiratory symptoms (wheeze, shortness of breath)	high
**Fei et al[126]**	2007	China	case-control	3486	40	over 35	coal and firewood/straw; electricity or coal	electricity or natural gas, no heating	COPD	low
**Firdaus et al[127]**	2011	India	retrospective cohort study	5949	NA	not reported	traditional fuel	no exposure to traditional fuel	acute respiratory infection, COPD, asthma, lung cancer, cardiovascular disease	high
**Fischer et al[128]**	1998	UK, Czech Republic, Poland, Netherlands	retrospective cohort study	16023	50.947	8.1	cooking gas, heating gas, other indoor source	no exposure to cooking gas, heating gas	wheeze, asthma attack, cough usually	high
**Foote et al[129]**	2013	Kenya	prospective cohort study	168	53	5 months	upesi jiko	no upesi jiko	pneumonia	high
**Franklin et al[130]**	2012	Australia	case-control	71	42	68.8	unflued gas heaters	other heating	asthma	high
**Galeone et al[131]**	2008	China	case-control	654	NA	not reported	solid fuel	gas heating, no coal for cooking	Lung cancer	low
**Gao et al[132]**	1987	China	case-control	1407	0	36 - 69	coal, gas, wood	no exposure to coal, gas, wood	lung cancer	low
**Garcia-Sancho et al[133]**	2009	Mexico	case-control	126	0	40.9	biomass fuel	less than 20 years of exposure to smoke from biomass fuels	tuberculosis	low
**Garrett et al[134]**	1998	Australia	case-control	148	NA	10.2	gas stove and NO2 concentration	no gas stove	respiratory symptoms (cough, shortness of breath, wheeze, chest tightness, asthma attacks)	high
**Gaviola et al[135]**	2016	Peru	cross-sectional	2953	49	55.0	biomass fuel	no regular exposure to biomass	asthma	high
**Ghimire et al[136]**	2019	Nepal	cross-sectional	23335	0	not reported	biomass energy	natural gas	perinatal mortality and extended perinatal mortality	high
**Ghosh et al[137]**	2006	India	cross-sectional	32470	52.2	not reported	biomass fuel	cleaner fuel	low birth weight	high
**Gillespie-Bennett et al[138]**	2011	New Zealand	cross-sectional	409	59	9.6	unflued gas heating and NO2 concentration	no NO2 exposure	asthma	high
**Gninafon et al[139]**	2011	Benin	case-control	600	65.8	34.8	solid fuel	non-solid fuel	tuberculosis	moderate
**Golshan et al[140]**	2002	Iran	cross-sectional	561	0	27.6	kerosene, wood, LPG	no kerosene, wood, LPG	asthma and chronic bronchitis	high
**Gonzalez-Garcia et al[141]**	2015	Colombia	cross-sectional	5539	33.2	over 40	wood smoke	no exposure to wood smoke	asthma, wheeze	high
**Guggisberg et al[142]**	2003	Canada	cross-sectional	402	47	25.5	woodsmoke	no exposure to woodsmoke	respiratory symptoms (wheeze, chest tightness,	high

									cough, phlegm, dyspnoea on exertion)	
**Gupta et al[143]**	1997	India	cross-sectional	707	69.9	not reported	wood or cow dung	gas, kerosene or coal	tuberculosis, COPD, acute respiratory infection	moderate
**Gupta et al[144]**	2001	India	case-control	890	86	not reported	coal, wood	not exposed to coal or wood	lung cancer	low
**Gurley et al[145]**	2013	Bangladesh	prospective cohort study	257	53	1.9	biomass fuel	clean fuel	acute respiratory infection	high
**Hansel et al[146]**	2008	USA	prospective cohort study	150	58	4.4	NO2 exposure	no exposure to NO2	asthma symptoms	high
**Hansel et al[147]**	2013	USA	prospective cohort study	84	58	68.9	indoor particles	indoor particles	exacerbations	high
**Haque et al[148]**	2016	Bangladesh	case-control	552	40.95	38.8	fuel of plant origin and cow dung	clear fuel	tuberculosis	high
**Harerimana et al[149]**	2016	Rwanda	cross-sectional	8599	50.7	under 5	improved cooking fuel	unimproved cooking fuel	acute lower respiratory infection	high
**Hasan et al[150]**	2017	Bangladesh, Nepal, Pakistan	cross-sectional	23940	NA	under 5	not ideal cooking fuel	ideal cooking fuel	acute respiratory infection	high
**Hasan et al[151]**	2019	India	cross-sectional	10575	0	15-49	biomass fuel	no biomass fuel	symptoms	high
**Hazra et al[152]**	2007	India	cross-sectional	NA	0	not reported	traditional fuel	modern fuel	asthma and tuberculosis	high
**Helsing et al[153]**	1982	USA	retrospective cohort study	708	80.8	not reported	gas	electricity	respiratory symptoms	high
**Hernandez-Garduno et al[154]**	2004	Mexico	case-control	386	0	44-87	woodsmoke	not exposed to woodsmoke	lung cancer	moderate
**Hersoug et al[155]**	2010	Denmark	cross-sectional	3471	44.7	18 to 69	wood	no exposure to wood	wheeze, cough	high
**Hessel et al[156]**	2001	Canada	case-control	284	60.6	5-19	gas	no gas	asthma	high
**Hoek et al[157]**	1984	Netherlands	case-control	231	NA	6.0	NO2 concentration	NA	respiratory symptoms	high
**Holscher et al[158]**	2000	Germany	cross-sectional	2198	50	5 to 14	gas	no exposure to gas	asthma, bronchitis, rhinitis, wheezing, shortness of breath, cough, cough without cold, cough during day/night, ‰¥3 febrile infections, acute infection	high
**Honicky et al[159]**	1985	USA	case-control	62	60	3.5	wood	no wood burning stove	wheeze and cough	high
**Hosein et al[160]**	1989	USA	cross-sectional	1357	53.7	7-70	gas	electricity	symptoms	high
**Hosgood et al[161]**	2008	China	retrospective cohort study	8418	52.1	56.3	portable stove	unvented stove	lung cancer death	moderate
**Hosgood et al[162]**	2010	USA, Canada, Singapore, China, Hungary, Poland, Czech Republic,Slovakia, Romania and Russia	case-control	11641	58.2	not reported	solid fuel	non-solid fuel	lung cancer	low
**Howden-Chapman et al[163]**	2008	New Zealand	randomised-controlled trial	349	57.3	10.0	non-polluting, more effective replacement heater	less effective form of heating	respiratory symptoms (wheeze, dry cough at night)	high
**Huang et al[164]**	1992	China	case-control	270	NA	not reported	coal	no coal	lung cancer	moderate

**Hystad et al[165]**	2019	Bangladesh, Brazil, Chile, China, Colombia, India, Pakistan, Philippines, South Africa, Tanzania, Zimbabwe	prospective cohort study	91350	41.2	50.2	solid fuel	clean fuel	all-cause mortality, cardiovascular mortality, respiratory mortality, myocardial infarction, stroke, heart failure, tb, copd, pneumonia, lung cancer, asthma	high
**Indahsari et al[166]**	2018	Indonesia	cross-sectional	156	NA	under 5	charcoal, wood, kerosene	no biomass	acute respiratory infection	high
**Infante-Rivard et al[167]**	1993	Canada	case-control	914	54.9	3-4	gas, kerosene, fire place, wood stove	no gas cooking appliance or no kerosene space heater, no fire place or no wood stove	asthma	moderate
**Ingale et al[168]**	2013	India	cross-sectional	750	50	46.0	LPG, agrowaste, wood	no LPG, agrowaste, wood	respiratory symptoms (difficulty in respiration, frequent coughing, throat irritation, chest pain)	high
**Islam et al[169]**	2013	India	cross-sectional	370	68	under 5	wood, kerosene, cow dung	gas	acute respiratory infection	high
**Jafta et al[170]**	2019	South Africa	case-control	227	46.3	under 15	dirty	clean	tuberculosis	low
**Jaganath et al[171]**	2015	Peru	cross-sectional	2957	49.3	over 35	Biomass fuel	no exposure to biomass fuel	COPD	moderate
**Jamali et al[172]**	2017	Pakistan	cohort study	605	0	32.4	improved stoves	traditional stoves	asthma and respiratory symptoms	high
**Janjua et al[173]**	2012	Pakistan	cross-sectional	566	50.2	under 5	biomass fuel	fossil fuel	acute respiratory infection	high
**Jarvis et al[174]**	1996	United Kingdom	cross-sectional	1159	43.1	22 to 44	gas	no exposure to gas	respiratory symptoms	high
**Jarvis et al[175]**	1998	14 European countries	prospective cohort study	11590	48	not reported	gas cooker	no gas cooker	wheeze	high
**Jedrychowski et al[176]**	1990	Poland	case-control	560	0	over 65	gas cooker	no gas cooker	respiratory symptoms	high
**Jedrychowski et al[177]**	1995	Poland	cross-sectional	1441	0	72.1	gas	no gas	asthma	high
**Jiang et al[178]**	2015	China	prospective cohort study	9895	0	not reported	coal, biomass, electromagnet	gas	low birth weight	low
**Jie et al[179]**	2016	China	prospective cohort study	610	45.6	44.9	coal	clean fuel	asthma	high
**Jindal et al[180]**	2006	India	cross-sectional	35295	51.6	over 35	liquid petroleum gas, kerosene, or solid fuel	no self cooking	COPD	high
**Jindal et al[181]**	2012	India	cross-sectional	169577	50.188	over 15	electricity/LPG, coal/wood, other fuels	not regular exposure to cooking fuel	asthma, chronic bronchitis	high

**Johnson et al[182]**	2008	Nigeria	prospective cohort study	323	54.8	15.7	wood smoke	no exposure to wood smoke	ALRI	high
**Johnson et al[183]**	2011	India	cross-sectional	900	0	30 to 70	biomass fuel	clean fuel	COPD	high
**Jubulis et al[184]**	2014	India	case-control	178	57	3.0	biomass fuel	not exposed to biomass fuel	tuberculosis	low
**Juntarawijit et al[185]**	2019	Thailand	cross-sectional	1134	16.3	53.4	wood, charcoal	lpg	symptoms	high
**Kadam et al[186]**	2013	India	retrospective cohort study	328	0	19-35	wood, kerosene	LPG	low birth weight	high
**Kafando et al[187]**	2018	Burkina Faso	cross-sectional	922	NA	under 5	wood, charcoal	gas	acute respiratory infection	high
**Kaji et al[188]**	2014	USA	case-control	77	61	69.4	PM2.5 concentration	na	respiratory symptoms	high
**Kan et al[189]**	2011	China	case-control	606	72.1	55.5	Solid fuel (coal and biomass) included coal/lignite, charcoal, wood, straw/ shrubs/grass, animal dung, and agricultural crop residue; non-solid fuel included electricity, liquefied petroleum gas, natural gas, biogas, and kerosene	No solid fuel	tuberculosis	high
**Kashima et al[190]**	2010	Indonesia	cross-sectional	15242	51.6	1.9	coal/lignite, charcoal, firewood/straw, and dung	electricity, liquid petroleum gas electricity, liquid petroleum gas/natural gas, biogas, and kerosene	acute upper respiratory infection, acute lower respiratory infection, neonatal mortality, infant mortality, stillbirth	high
**Kasznia-Kocot et al[191]**	2010	Poland	cross-sectional	1130	44.8	13 to 15	coal	no exposure to coal	asthma	high
**Khalequzzaman et al[192]**	2007	Bangladesh	cross-sectional	95	NA	under 5	biomass	fossil fuel	nasal discharge, cough, shortness of breath, chest tightness, wheeze, respiratory diseases (bronchiolitis, asthma, pneumonia)	high
**Khan et al[193]**	2017	Bangladesh	cross-sectional	22789	0	25.6	solid fuel	Clean fuel	Acute respiratory infection, low birth weight, stillbirth, all-cause mortality under 5 years, neonatal mortality	high
**Khan et al[194]**	2018	Pakistan	cross-sectional	11040	50.95	0-59 months	solid fuels and kerosene, where solid fuels include wood, animal dung, charcoal, coal, and shrubs/ grass/straw	natural gas, LPG, biogas, and electricity	acute respiratory infection	high
**Kilabuko et al[195]**	2007	Tanzania	case-control	5524	49.7	under 5	Biomass fuel	kerosene/charcoal	acute respiratory infection	high
**Kilpelainen et al[196]**	2001	Finland	cross-sectional	10667	39	18-25	Wood stove heating at age 0-6 years	Other heating system at age 0-6 years	asthma and wheeze	high
**Kim et al[197]**	2014	China	case-control	520	100	18-85	Ever smoky coal use	Never smoky coal use	lung cancer	high
**Kim et al[198]**	2015	China	prospective cohort study	71320	0	51.4	coal, coke balls	gas	lung cancer	high
**Kim et al[199]**	2016	China	prospective cohort study	74941	0	52.0	coal	no coal	all cause mortality, lung cancer mortality, cardiovascular mortality	low

**Kiraz et al[200]**	2003	Turkey	cross-sectional	344	0	41.4	biomass fuel	no biomass fuel	Chronic bronchitis	moderate
**Kleimola et al[201]**	2015	47 countries	cross-sectional	1526209	NA	under 5	solid fuel	clean fuel	neonatal and child mortality	high
**Kleinerman et al[202]**	2002	China	case-control	2651	74	35-70	coal	biomass	lung cancer	low
**Ko et al[203]**	1997	Taiwan	case-control	210	0	60.7	coal or anthracite, wood or charcoal	no cooking or gas	lung cancer	moderate
**Kolappan et al[204]**	2009	India	case-control	1530	86.67	over 15	biomass	non-biomass	tuberculosis	moderate
**Koo et al[205]**	1983	China	case-control	400	0	61.2	kerosene, LPG	no kerosene, LPG	lung cancer	moderate
**Kraai et al[206]**	2013	Venezuela	cross-sectional	630	50	6.0	wood	gas	wheeze	high
**Kristensen et al[207]**	2006	South Africa	prospective cohort study	571	50	not reported	paraffin or coal	electricity or gas	acute respiratory infection	moderate
**Kuai et al[208]**	2018	China	prospective cohort study	271217	41	51.0	solid fuel	clean fuel	cardiovascular mortality and all-cause mortality	low
**Kuhr et al[209]**	1991	Germany	cross-sectional	704	NA	7-16	LPG, wood, oil or coal	central, remote or floor heating	asthma	high
**Kumar et al[210]**	2004	India	case-control	100	70	2 months - 5 years	fuel other than LPG	LPG	ALRI	low
**Kumar et al[211]**	2008	India	cross-sectional	912	59.2	7-15	biomass fuel	LPG	asthma	high
**Kurmi et al[212]**	2014	Nepal	cross-sectional	1648	46.2	35.3	biomass	non-biomass	respiratory symptoms	high
**Kurt et al[213]**	2007	Turkey	cross-sectional	25843	50.1	9.5	natural gas	wood, coal, biomass	asthma, wheeze	high
**Kurt et al[214]**	2009	Turkey	cross-sectional	25843	NA	not reported	wood, biomass, coal	LPG, natural gas	asthma, wheeze	high
**Kurti et al[215]**	2016	Belize	cross-sectional	67	28.4	44.2	household air pollution	household air pollution	wheeze	high
**Kwas et al[216]**	2017	Tunisia	cross-sectional	140	0	48.0	biomass	no biomass	COPD	high
**Lakshmi et al[217]**	2012	India	case-control	378	0	over 20	biomass, kerosene	LPG	tuberculosis	moderate
**Lakshmi et al[218]**	2013	India	cross-sectional	188917	NA	15-49	wood, kerosene,animal dung,crop residues, coke/coal/charcoal	LPG/ Electricity	stillbirth	moderate
**Lamichhane et al[219]**	2017	India	cross-sectional	16157	NA	not reported	polluting fuel	Cleaner fuel	acute respiratory infection	high
**Lamichhane et al[219]**	2017	India	cross-sectional	16157	52	2.1	polluting fuel	cleaner fuel	Acute respiratory infection	high
**Lan et al[220]**	1993	China	case-control	278	0	not reported	smoky coal	no smoky coal	lung cancer	moderate
**Lan et al[221]**	2002	China	retrospective cohort study	21232	52.5	39.0	Improved stoves	Firepits and stoves without chimneys	Lung cancer	low
**Lan et al[222]**	2008	China	case-control	996	52.2	53.0	smoky coal	smokeless coal or wood	lung cancer	low
**Laniado-Laborin et al[223]**	2011	Mexico	cross-sectional	2293	39.8	57.6	biomass fuel	not exposed to biomass	COPD	low
**Lanphear et al[224]**	2001	USA	cross-sectional	8257	49	under 6	gas stove	no gas stove	asthma	high
**Lee et al[225]**	2001	Taiwan	case-control	527	44.78	61.4	coal/anthracite, wood/charcoal	no cooking or gas	lung cancer	low
**Lee et al[226]**	2012	China	cross-sectional	14068	45.9	48.6	solid fuels	no exposure to solid fuels	Hypertension, CHD, stroke, diabetes	high

**Levesqu et al**[227]	2001	Canada	cross-sectional	178	30.9	37.0	wood	electricity	upper and lower respiratory illness	high
**Lewis et al**[228]	2017	India	cross-sectional	105	NA	not reported	improved cookstove	traditional cookstove only	cold and cough in past month	high
**Li et al**[229]	2019	China	cross-sectional	1207	48.7	not reported	use of a coal stove for cooking or warming	no use of a coal stove for cooking or warming	asthma	high
**Li et al**[230]	2015	China	case-control	531	NA	51.9	smoky coal	wood or smokeless coal	lung cancer	moderate
**Li et al**[231]	2019	China	prospective cohort study	475827	40.6	45.5	solid fuel	cleaner fuel, no solid fuel	COPD	low
**Lin et al**[232]	2013	Netherlands	prospective cohort study	3590	52	not reported	LPG	no LPG	asthma and wheeze	high
**Lin et al**[233]	2012	China	cross-sectional	7259	43.2	not reported	smoky coal, coke, smokeless coal	wood	lung cancer	high
**Lissowska et al**[234]	2005	Seven European countries	case-control	5979	75.4	not reported	solid fuel	nonsolid fuel	lung cancer	low
**Liu et al**[235]	1993	China	case-control	316	70.9	61.0	LPG, wood	coal	lung cancer	low
**Liu et al**[236]	2007	China	cross-sectional	3286	41.7	over 40	coal, biomass	LPG	COPD	high
**Liu et al**[237]	2014	China	cross-sectional	23326	50.3	43986.0	coal	no coal	asthma and asthma related symptoms	high
**Liu et al**[238]	2014	China	cross-sectional	13335	50.8	9.5	coal, wood or natural gas	electric cooking	respiratory symptoms	high
**Liu et al**[239]	2018	China	prospective cohort study	5384	NA	above 45	solid fuel	no solid fuel	chronic lung disease, heart disease and stroke	high
**Liu et al**[240]	2018	China	cross-sectional	13335	50.8	above 45	natural gas	electicity	preterm birth (PTB), low birth weight (LBW), term low birth weight (T-LBW), small for gestational age (SGA)	high
**Liu et al**[239]	2018	China	prospective cohort study	5384	NA	4.8	solid fuel	no solid fuel	chronic lung disease, heart disease and stroke	high
**Loeb et al**[241]	2009	Canada	case-control	1584	44.6	76.5	gas stove	no gas stove	pneumonia	high
**Luo et al**[242]	1996	China	case-control	408	76.5	over 60	Coal	No coal use	Lung cancer	low
**Mahalanabis et al**[243]	2002	India	case-control	262	56.5	under 1	Solid fuel	No solid fuel	pneumonia	moderate
**Maier et al**[244]	1997	USA	cross-sectional	925	49.9	not reported	solid fuel	No solid fuel use	asthma and wheezing	moderate
**Malinauskiene et al**[245]	2011	Lithuania	case-control	1093	0	35-61	gas stove	electric stove	first myocardial infarction	moderate
**Manyeh et al**[246]	2016	Ghana	cross-sectional	6777	52.2	not reported	wood, charcoal, other	gas	low birth weight	high
**Mavalankar et al**[247]	1991	India	case-control	2076	0	not reported	cooking smoke	no exposure to cooking smoke	stillbirth	low
**Mavalankar et al**[248]	1992	India	case-control	2782	0	not reported	cooking smoke	no exposure to cooking smoke	low birth weight	high
**McConnell et al**[249]	2002	USA	cohort study	3535	47.4	42614	Gas, wood	no gas or wood	asthma	high
**McCormack et al**[250]	2009	USA	cohort study	150	58	4.4	PM2.5 concentration	NA	asthma symptoms	high
**Mejias et al**[251]	2018	Dominican Republic	cross-sectional	624	50	3-11	gas	electicity	asthma	high

**Melia et al[252]**	1977	UK	prospective cohort study	5758	50.7	6-11	gas	electricity	respiratory symptoms	high
**Melia et al[253]**	1979	United Kingdom	cohort study	2408	49.3	44141.0	gas cooking	electric cooking	respiratory symptoms	high
**Melsom et al[254]**	2001	Nepal	case-control	247	63	14.1	open fire or burning stove without a flue, wood and grass	gas, kerosene stove, fuel such as wood and grass	asthma	high
**Menezes et al[255]**	1994	Brazil	cross-sectional	1053	40.8	not reported	Indoor pollution	No source of smoke	chronic bronchitis	high
**Milanzi et al[256]**	2017	Malawi	cross-sectional	9124	0	not reported	High pollution fuel	Low pollution fuel	birth weight and size at birth	high
**Mir et al[257]**	2012	India	cross-sectional	1644	53.9	not reported	Cooking fuel other than LPG	LPG	acute respiratory infection	high
**Mishra et al[258]**	1999	India	cross-sectional	260162	51.4	over 20	biomass fuel (wood or dung)	cleaner fuel (charcoal, coal/coke/lignite, kerosene, electricity, petroleum gas, or biogas).	tuberculosis	high
**Mishra et al[259]**	2003	India	cross-sectional	38595	52.9	over 60	wood, crop residues, dung cakes or a mix of biomass fuels and cleaner fuels or coal/coke/lignite/charcoal group	kerosene, petroleum gas, biogas, or electricity	asthma	high
**Mishra et al[260]**	2003	Zimbabwe	cross-sectional	3559	48.7	under 5	high and medium pollution fuel	LPG/natural gas or electricity	acute respiratory infection	high
**Mishra et al[261]**	2004	Zimbabwe	cross-sectional	3559	NA	not reported	wood, dung, or straw	Liquid petroleum gas/natural gas or electricity	birth weight	high
**Mishra et al[262]**	2005	India	cross-sectional	19189	NA	not reported	wood, dung, crop residues, mix of biomass (the former) and any of (electricity, liquid petroleum gas, biogas, or kerosene), or coal,coke,lignite, charcoal	electricity, liquid petroleum gas, biogas, or kerosene	stillbirth	high
**Mishra et al[263]**	2005	India	cross-sectional	29768	51.8	40-49	Cooking fuel (high/medium pollution)	Cooking fuel (low pollution)	acute respiratory infection	high
**Misra et al[264]**	2018	South Africa	cross-sectional	415	0	24.0	wood	electricity	respiratory symptoms and illnesses	high
**Mitter et al[265]**	2016	Iran	prospective cohort study	50045	42	52.1	LPG, kerosene/diesel, wood, dung	no LPG, kerosene/diesel, wood, dung	all-cause death, cardiovascular death, ischaemic heart disease mortality , respiratory disease death and cerebrovascular accident mortality	low
**Mommers et al[266]**	2005	Germany, The Netherlands	case-control	1191	NA	7-8	gas cooking daily, heating with coal, wood, gas or oil	gas cooking not daily, central heating or heating with electricity	asthmatic symptoms, coughing	high
**Moran et al[267]**	1999	UK	cohort study	1449	NA	34-35	gas	electricity	asthma/wheeze, respiratory symptoms	high
**Morris et al[268]**	1990	USA	case-control	116	NA	under 2	Wood burning stove	No wood burning stove	lower respiratory tract infection (bronchiolitis or pneumonia)	high
**Mortimer et al[269]**	2017	Malawi	randomised-controlled trial	10750	48.4	under 5	cleaner burning biomass-fuelled cookstoves	traditional cooking methods	pneumonia, under-five mortality	low
**Mu et al[270]**	2013	China	case-control	865	50.4	not reported	solid fuel, coal, coke	clean fuel	lung cancer	moderate
**Mukherjee et al[271]**	2014	India	cross-sectional	1119	0	30.1	biomass	LPG	respiratory symptoms	high

**Mukherjee et al[272]**	2015	India	cross-sectional	404	0	34.6	wood, cow dung and agricultural residues like hay, paddy husk, dried leaf, bamboo, jute stick	liquefied petroleum gas	adverse reproductive outcomes	high
**Murray et al[273]**	2012	Bangladesh	prospective cohort study	6079	50	under 5	dung, wood, and crop residues	natural gas and other non biomass	acute lower respiratory infection	low
**Musafiri et al[274]**	2018	Rwanda	prospective cohort study	436	0	32.2	firewood, charcoal and grass/leaves	liquefied gas petroleum or had not been cooking the last three years	Chronic bronchitis	high
**Mustapha et al[275]**	2011	Nigeria	cross-sectional	1397	NA	7-14	wood or coal, kerosene	gas	asthma and respiratory symptoms	high
**Mzileni et al[276]**	1999	South Africa	case-control	728	64.7	57.2	wood, coal	no exposure to wood, coal	lung cancer	moderate
**Naz et al[277]**	2015	Bangladesh	cross-sectional	18308	NA	under 5	polluting fuel	clean fuel	neonatal mortality, infant mortality and under-five mortality	high
**Naz et al[278]**	2016	India	cross-sectional	166382	NA	under 5	polluting fuel	clean fuel	neonatal mortality, post-neonatal mortality, child mortality	high
**Naz et al[279]**	2017	Pakistan	cross-sectional	11507	50.9	under 5	kerosene, coal/lignite, charcoal, wood, straw/shrubs/grass and animal dung	electricity, liquid petroleum gas (LPG), natural gas and biogas	child mortality	high
**Naz et al[280]**	2018	Nepal	cross-sectional	17780	NA	under 5	polluting fuels	clean fuels	neonatal mortality, post-neonatal mortality, child mortality, under-five mortality	high
**Neas et al[281]**	1991	USA	cross-sectional	1567	NA	7-10	NO2 concentration	NA	asthma and respiratory symptoms	high
**Neas et al[282]**	1994	USA	cross-sectional	1237	NA	7-11	PM2.5	NA	asthma and respiratory symptoms	high
**Ngahane et al[283]**	2015	Cameroon	cross-sectional	300	0	51.0	wood	alternative source of energy for cooking	chronic bronchitis, dyspnea on exertion	high
**Ngocho et al[284]**	2019	Tanzania	case-control	463	59	13 months	biomass, firewood, charcoal, kerosene	gas or electricity	community acquired pneumonia	low
**Nisha et al[285]**	2018	Bangladesh	cross-sectional	27237	0	not reported	kerosene, coal/lignite, charcoal, wood, straw/shrubs/grass, agricultural crop, animal dung	electricity, liquefied petroleum gas, natural gas, biogas	stillbirth and early neonatal mortality	high
**Nitschke et al[286]**	2006	Australia	cross-sectional	174	54	8.0	NO2 concentration	NA	asthma symptoms	high
**Noonan et al[287]**	2007	USA	cross-sectional	397	49	6 - 15	woodstove, fireplace, propane or oil	electricity	wheezing in the past 4 weeks	high
**Noonan et al[288]**	2011	USA	prospective cohort study	920	50.6	not reported	improved woodstove	older woodstove	respiratory symptoms	high
**Noorhassim et al[289]**	1995	Malaysia	cross-sectional	1007	51.4	1-12	wood	no wood stove	asthma	high
**Norback et al[290]**	2019	China	cross-sectional	39782	52	4.4	biomass (coal, wood), natural gas	only electricity	doctor diagnosed asthma, current wheeze	high
**North et al[291]**	2017	Uganda	prospective cohort study	734	30	34.0	wood	charcoal	chronic cough	high
**Obaseki et al[292]**	2017	Nigeria	cross-sectional	1147	37	over 40	solid fuel	no solid fuel	respiratory symptoms	high
**Oluwole et al[293]**	2017	Nigeria	cross-sectional	1690	51.7	13.6	biomass fuel	no biomass fuel	asthma and respiratory symptoms	high

**Orozco-Levi et al[294]**	2006	Spain	case-control	120	0	71.0	wood, charcoal smoke	not exposed to wood, charcoal smoke	COPD	moderate
**Ostro et al[295]**	1993	USA	cohort study	321	48	36.6	gas stove	no gas stove	any lower respiratory tract symptom or illness	high
**Ostro et al[296]**	1994	USA	cross-sectional	256	33.8	44.3	gas, wood or fireplace	no gas, wood or fireplace	respiratory symptoms	high
**Owili et al[297]**	2017	23 subsaharan african countries (Benin, Bukina Faso, Burundi, Comoros, Congo, CoÌ‚te dâ€™Ivoire, DRC, Ethiopia, Gabon, Gambia, Guinea, Kenya, Liberia, Mali, Mozambique, Nigeria, Namibia, Rwanda, Sierra Leone, Togo, Uganda, Zambia, and Zimbabwe)	cross-sectional	783691	NA	under 5	biomass fuel, charcoal, coal, lignite or paraffin/kerosene	clean fuel	under-five mortality	high
**Oxlade et al[298]**	2012	India	cross-sectional	198754	0	not reported	biomass fuel	no biomass fuel	tuberculosis	high
**Ozturk et al[299]**	2014	Turkey	case-control	771	100	36.8	coal or wood	not coal or wood	pulmonary tuberculosis	moderate
**Padhi et al[300]**	2008	India	cross-sectional	1505	NA	5-10	biomass fuel, LPG	no biomass fuel, LPG	asthma and respiratory symptoms	high
**Pan et al[301]**	2002	China	retrospective cohort study	467	52.5	36.1	charcoal stove	no charcoal stove	asthma	high
**Pandey et al[302]**	2012	India	cross-sectional	34472	0	32.1	coal, wood, straw, shrubs, grass, agricultural crop waste, dung cakes, lignite, charcoal	electricity, liquid petroleum gas or Natural gas, biogas or Kerosene	child mortality	high
**Panigrahi et al[303]**	2018	India	cross-sectional	1120	0	30.0	solid biomass fuel	LPG	chronic bronchitis	high
**Pant et al[304]**	2012	Nepal	cross-sectional	2142	49	27.0	dung	biogas	asthma	high
**Patel et al[305]**	2013	India	cross-sectional	35954	51	0-36 months	wood, agricultural waste, dung, or straw, coal/lignite, charcoal, or kerosene	liquid petroleum gas/natural gas, electricity	acute respiratory infection	high
**Patel et al[306]**	2015	Guatemala, India, Kenya, Pakistan, Zambia	prospective cohort study	65912	0	not reported	Polluting fuels	clean fuels	perinatal mortality	low
**Patel et al[307]**	2019	India	cross-sectional	932341	52.4	under 5	biomass fuel	no biomass fuel	acute respiratory infection	high
**Pathirathna et al[308]**	2017	Sri Lanka	prospective cohort study	76	0	29.0	wood	no wood	low birthweight	high
**Patra et al[309]**	2012	India	case-control	400	NA	under 14	biomass fuel	LPG	tuberculosis	high
**Patra et al[310]**	2016	51 countries	cross-sectional	112711	46.3	not reported	solid fuel	no solid fuel	wheeze, asthma	high
**Paulin et al[311]**	2017	USA	cohort study	30	67	9.8	NO2 concentration	NA	asthma symptoms	high
**Peabody et al[312]**	2005	China	cross-sectional	6923	35.2	29.2	wood, crop residues, cleaner fuels	coal	COPD, Asthma	high
**Perez-Padilla et al[313]**	1996	Mexico	case-control	502	0	56.3	wood	no wood	Chronic airway obstruction and chronic bronchitis	low
**Perez-Padilla et al[314]**	2001	Mexico	case-control	925	45.7	39.3	wood stove	never used wood stoves	tuberculosis	low
**Pershagen et al[315]**	1995	Sweden	case-control	547	56.8	under 4	gas stove	no gas stove	wheezing	moderate

**Phillips et al[316]**	2018	England, Wales	geographical study	3535136	NA	35-74	solid fuel	no solid fuel	all cause mortality, cardiovascular mortality, respiratory mortality mortality	high
**Phoa et al[317]**	2004	Australia	cross-sectional	627	46.7	8-11	fume emitting heating	non-fume emitting heating	asthma	high
**Phukan et al[318]**	2014	India	case-control	690	0	58.7	coal, wood	LPG	lung cancer	low
**Piekarska et al[319]**	2018	Poland	cross-sectional	18617	46.2	6-44	solid fuel, electric heaters	other heating sources	asthma	high
**Pokharel et al[320]**	2001	India	case-control	120	67.5	12.4	biomass fuel	no exposure to biomass fuel	bronchial asthma	high
**Pokhrel et al[321]**	2010	Nepal	case-control	375	0	35.0	kerosene, biomass, coal	gas or electricity	tuberculosis	low
**Ponsonby et al[322]**	2000	Australia	prospective cohort study	863	69	7.0	gas	no gas	asthma	high
**Ponsonby et al[323]**	2001	Australia	cross-sectional	440	NA	9.1	gas	no gas	asthma	high
**Pope et al[324]**	1993	China	cross-sectional	973	0	20 to 40	coal	no exposure to coal	chest illness, cough, phlegm, shortness of breath, wheeze	high
**Praygod et al[325]**	2016	Tanzania	case-control	117	53	under 5	wood, charcoal	gas or electricity	severe pneumonia	high
**Prietsch et al[326]**	2006	Brazil	cross-sectional	685	NA	not reported	Inadequate heating (combustion)/wood burning stove for cooking	NA	wheezing	high
**Qian et al[327]**	2000	China	cross-sectional	2789	35.3	not reported	coal	no coal	respiratory symptoms	high
**Qian et al[328]**	2004	China	cross-sectional	7058	NA	38.4	Coal smoke for cooking/heating	NA	wheezing, bronchitis, asthma	high
**Qian et al[329]**	2007	China	cross-sectional	2517	100	5-14	Coal smoke for cooking/heating	NA	wheezing, bronchitis, asthma	high
**Qin et al[330]**	2002	China	case-control	252	0	57.7	LPG, coal	no LPG or coal	lung cancer	moderate
**Qu et al[331]**	2015	China	cross-sectional	13424	46.7	48.5	solid fuel	no solid fuel	stroke, coronary heart disease	high
**Rabbani et al[332]**	2017	India	case-control	356	0	34.0	biomass	cleaner fuel	tuberculosis	high
**Rahut et al[333]**	2017	Bhutan	cross-sectional	6551	NA	not reported	wood, kerosene, dung, charcoal, leaves	electricity, LPG	tuberculosis	high
**Ram et al[334]**	2014	Bangladesh	case-control	994	50.3	under 5	LPG, petroleum gas or kerosene	wood or bamboo	pneumonia	moderate
**Ramanakumar et al[335]**	2007	Canada	case-control	2746	60.6	35-75	wood or coal	no wood or coal	lung cancer	moderate
**Ramani et al[336]**	2016	India	cohort study	400	44.8	under 5	LPG and wood	no wood and LPG	acute respiratory infection	high
**Rana et al[337]**	2019	Afghanistan	cross-sectional	27565	0	51.7	solid fuel	no solid fuel	Acute respiratory infection	high
**Raspanti et al[338]**	2016	Nepal	case-control	1212	59.2	over 18	wood, charcoal, agricultural waste	electricity and natural gas	lung cancer	low
**Regalado et al[339]**	2006	Mexico	cross-sectional	841	0	38 and over	Biomass	Gas	COPD	low
**Rehfuess et al[340]**	2009	Sub Saharan Africa	cross-sectional	32620	NA	under 5	cooking with solid fuel	cooking with cleaner fuel	ALRI mortality in children <5 years age	high

**Rey-Ares et al**[341]	2016	Argentina, Chile	retrospective cohort study	1074	NA	under 5	Exposure to Household air pollution	Households not exposed to HAP	Lower respiratory tract infection (LRTI)	moderate
**Rinne et al**[342]	2007	Ecuador	cross-sectional	212	54.2	under 16	biomass fuel	LPG only	infant mortality and respiratory symptoms	high
**Riojas-Rodriguez et al**[343]	2001	Mexico	prospective cohort study	56	23.2	not reported	Ceta stove	open fire	respiratory symptoms	high
**Robin et al**[344]	1996	Arizona, United States	case-control	90	60	1 - 24 months of age	wood	gas, electricity	acute respiratory infection	low
**Romieu et al**[345]	2009	Mexico	randomised-controlled trial	552	NA	25.9	improved stove	open fire	respiratory symptoms	high
**Rudnai et al**[346]	2008	Hungary	cross-sectional	6670	50.3	9-10	wood, coal	no wood, coal	bronchitic and asthmatic symptoms	high
**Rumchev et al**[347]	2016	Myanmar	cross-sectional	80	NA	under 6	biomass fuel	no biomass fuel	respiratory symptoms	high
**Rumchev et al**[348]	2017	India	cross-sectional	478	NA	30.13, children under 15	biomass	liquid petroleum gas or kerosene	respiratory symptoms	high
**Rylance et al**[349]	2019	Malawi	cross-sectional	804	48.1	7.1	wood, crop waste, charcoal	wood, crop waste, charcoal	chronic cough, current wheeze, symptoms of severe asthma, shortness of breath	high
**Salo et al**[350]	2004	China	cross-sectional	5231	52.5	13.6	coal	no coal	respiratory symptoms	high
**Sama et al**[351]	2017	USA	case-control	171	40	70.0	wood and gas stove	no wood or gas stove	COPD exacerbation	high
**Samet et al**[352]	1993	USA	prospective cohort study	1205	52.1	not reported	gas stove	electric stove	respiratory symptoms	high
**Samuel et al**[353]	2018	Nigeria	cross-sectional	10983	NA	not reported	coal, cow dung, firewood and charcoal	electricity, liquid petroleum gas, kerosene	under-five mortality	high
**Sana et al**[354]	2019	Burkina Faso	cross-sectional	1705	0	36.0	firewood, charcoal	liquid petroleum gas	acute and chronic respiratory symptoms	high
**Sanbata et al**[355]	2014	Ethiopia	cross-sectional	422	67.5	under 5	biomass or kerosene; traditional stove	LPG and electricity; clean stove	acute respiratory infection	high
**Sanya et al**[356]	2014	Uganda	case-control	86	39.5	36.0	wood or charcoal	no wood or charcoal	asthma exacerbation	high
**Sapkota et al**[357]	2008	India	case-control	1517	85.8	not reported	solid fuel, wood, coal, mixed/other	modern fuel	lung cancer	low
**Sasco et al**[358]	2002	Morocco	case-control	353	96.6	59.0	coal	no coal	lung cancer	high
**Savitha et al**[359]	2007	India	case-control	208	58.2	under 5	wood, dung, kerosene	LPG	acute lower respiratory tract infection	high
**Schei et al**[360]	2004	Guatemala	cross-sectional	1058	48	4-6	open fire	plancha stove	asthma and asthma related symptoms	high
**Sezer et al**[361]	2006	Turkey	case-control	148	0	56.7	wood and biomass	no exposure to wood or biomass	COPD	high
**Shah et al**[362]	1994	India	case-control	400	57	2 months - 5 years	smoke producing stove	no smoke producing stove	severe pneumonia	low
**Sharma et al**[363]	1998	India	prospective cohort study	642	52.4	under 5	wood	kerosene	Lower respiratory tract infection	high
**Sharma et al**[364]	2013	India	cross-sectional	500	51.4	under 1	smoky fuel	smokeless fuel	acute respiratory infection	high

**Shen et al**[365]	2009	China	retrospective cohort study	42422	NA	not reported	stove improvement, smokeless coal	fire-pit or stove without a chimney and no smokeless coal	pneumonia	high
**Shetty et al**[366]	2006	India	case-control	370	58	15 to 83	biomass fuel	gas/electric	tuberculosis	high
**Shima et al**[367]	2000	Japan	prospective cohort study	842	51.5	9-10	NO2 concentration	NA	asthma, wheeze	high
**Shirinde et al**[368]	2014	South Africa	cross-sectional	3468	47.7	13-14	LPG, paraffin, wood, coal	electricity	wheeze	high
**Shrestha et al**[369]	2005	Nepal	cross-sectional	168	6	36.1	unprocessed solid fuels	processed fuels	acute respiratory infection, COPD/Asthma and respiratory symptoms	high
**Siddharthan et al**[370]	2018	Argentina, Chile, Uruguay, Peru, Bangladesh, Uganda	cross-sectional	12396	51.5	54.9	biomass	no exposure to biomass	COPD	low
**Siddiqui et al**[371]	2005	Pakistan	cross-sectional	122	0	28.6	wood	natural gas	respiratory symptoms	high
**Siddiqui et al**[372]	2008	Pakistan	retrospective cohort study	634	NA	24.7	wood	natural gas	low birth weight	high
**Sikolia et al**[373]	2002	Kenya	cross-sectional	300	NA	under 5	Firewood, kerosene	Charcoal	acute respiratory infection	high
**Singh et al**[374]	2002	India	cross-sectional	2275	55.1	7.8	smoke producing fuel	no smoke producing fuel	cough	high
**Sloan et al**[375]	2012	USA	case-control	528	43	not reported	wood or coal	no wood or coal	lung cancer	moderate
**Smith et al**[376]	2000	Australia	cross-sectional	129	55	not reported	NO2 concentration	NA	asthma symptoms	high
**Smith et al**[377]	2011	Guatemala	Randomised-controlled trial	518	NA	under 18 months	locally developed chimney stove	open wood fires	pneumonia	low
**Smith-Sivertsen et al**[378]	2009	Guatemala	randomised-controlled trial	504	0	27.7	improved stove	open fire	respiratory symptoms	high
**Sobue et al**[379]	1990	Japan	case-control	875	0	56.7	wood or straw; for heating kerosene, gas, coal, charcoal and wood stoves without chimneys	no wood, straw or coal	lung cancer	moderate
**Solis-Soto et al**[380]	2013	Bolivia	cross-sectional	2340	47.9	11.0	wood or coal	gas or electricity	asthma symptoms	high
**Sood et al**[381]	2010	USA	prospective cohort study	1861	19.2	not reported	wood	no wood	airflow obstruction and chronic bronchitis	high
**Soto-Moreno et al**[382]	2013	Columbia	cross-sectional	2085832	51.9	not reported	solid fuel	no solid fuel	cardiovascular and respiratory limitation	high
**Speizer et al**[383]	1980	USA	prospective cohort study	9280	NA	6-10	gas	electicity	bronchitis, serious respiratory illness before age 2, respiratory illness in the last year	high
**Spengler et al**[384]	2004	Russia	cross-sectional	5951	NA	8-12	gas	no gas	asthma, wheeze, cough	high
**Sreeramareddy et al**[385]	2011	India	cross-sectional	47139	NA	not reported	wood, straw, animal dung, and crop residues, kerosene, coal	electricity, liquid petroleum gas, natural gas and biogas	low birth weight	high
**Stankovic et al**[386]	2011	Serbia	cross-sectional	1082	0	not reported	biomass	no biomass	respiratory symptoms and disease	low
**Stankovic et al**[387]	2011	Serbia	cross-sectional	367	0	20-40	fossil fuel smoke	central heating or electricity	respiratory symptoms, stillbirth	high
**Stein et al**[388]	2008	South Africa	case-control	9690	35	52.0	wood, charcoal, coal, anthracite, paraffin, or gas	electricity	lung cancer and cardiovascular disease	low

**Strachan et al[389]**	1995	UK	case-control	961	NA	11 to 16	gas	no gas	wheeze	high
**Sukhsohale et al[390]**	2013	India	cross-sectional	760	0	32.5	biomass, kerosene, mixed	LPG	asthma	high
**Sun et al[391]**	1992	China	case-control	816	0	30 to 69	bituminous coal, brazier	no exposure to bituminous coal, brazier	lung cancer	moderate
**Sun et al[392]**	2002	China	case-control	618	NA	not reported	coal stove, kang, gas	no coal stove, no kang, no gas	lung cancer	high
**Sunyer et al[393]**	2004	United Kingdom and Spain	prospective cohort study	1611	NA	under 1	gas stove and gas heating	no gas stove or heating	lower respiratory tract infection	high
**Suryadhi et al[394]**	2019	Indonesia	cross-sectional	36842	52	4.8	solid fuel	clean fuel	low birth weight, neonatal death, infant death, acute upper respiratory infection and acute lower respiratory infection	high
**Svendsen et al[395]**	2018	USA	cross-sectional	5210	50.1	not reported	wood, fireplace, portable gas heater	portable electric heater	asthma	high
**Tamire et al[396]**	2020	Ethiopia	cross-sectional	545	0	30.3	solid fuel	cleaner fuel	symptoms	high
**Tan et al[397]**	2015	Canada	cross-sectional	5176	42.8	57.0	coal or coke, wood, crop residues or dung	< 10 years coal or coke, wood, crop residues or dung	COPD	low
**Tavernier et al[398]**	2006	United Kingdom	case-control	200	42.5	4-17	LPG	no LPG	asthma	high
**Taylor et al[399]**	2012	Sierra Leone	cross-sectional	1040	0	Women age 15 - 45; Children under 5	wood	charcoal	acute respiratory infections	high
**Tazinya et al[400]**	2018	Cameroon	cross-sectional	512	56.8	under 5	wood smoke	no wood smoke	Acute respiratory infection	high
**Thacher et al[401]**	2013	Nigeria	cross-sectional	299	45.2	5-11	biomass fuel	no biomass fuel	asthma symptoms	high
**Thompson et al[402]**	2011	Guatemala	randomised-controlled trial	266	0	26.4	improved stoves	open fire	birth weight	low
**Tielsch et al[403]**	2009	India	prospective cohort study	11728	0	not reported	wood or dung	no wood or dung	birth weight, low birth weight, preterm birth, stillbirth, neonatal mortality, acute respiratory infection	low
**Tin et al[404]**	2016	New Zealand	prospective cohort study	6112	0	not reported	gas, electricity, wood	no gas, electricity or wood	acute respiratory infection	low
**Trang et al[405]**	2010	USA	case-control	1412	53	not reported	LPG, wood	no LPG, wood	asthma	high
**Tripathy et al[406]**	2017	India	cross-sectional	3571	55.3	not reported	unclean fuel	clean fuel	Neonatal, Postneonatal and Maternal Deaths	high
**Tse et al[407]**	2011	China	case-control	2277	100	66.0	gas, liquid fuel and solid fuel	never cooked or electricity	lung cancer	high
**Tumwesigire et al[408]**	1995	Uganda	cross-sectional	152	53.3	2.1	gas or electricity	wood or charcoal	acute respiratory infection	high
**Tuthill et al[409]**	1984	USA	cross-sectional	399	NA	not reported	woodstove	no woodstove	acute respiratory illness	high
**Upadhyay et al[410]**	2015	India	prospective cohort study	3961	53.5	3.1	solid fuel	cleaner fuel	life threatening respiratory illness	high
**Uzun et al[411]**	2003	Turkey	cross-sectional	177	0	41.0	biomass	no biomass	COPD and asthma	high

**van Gemert et al[412]**	2016	Uganda	cross-sectional	588	49.5	45.8	biomass fuel	no biomass fuel	COPD	moderate
**van Miert et al[413]**	2012	Belgium	cross-sectional	744	45	15.2	wood	no wood	asthma, pneumonia	high
**van Strien et al[414]**	2004	USA	cross-sectional	768	49	under 2	NO2	NA	respiratory symptoms	high
**Van Vliet et al[415]**	2019	Ghana	cross-sectional	1183	0	27.3	CO concentration	NA	Respiratory symptoms	high
**Vasudeva et al[416]**	2010	India	cross-sectional	124395	0	15-49	kerosene, wood, and agricultural products	Liquid petroleum gas; electricity and biogas	asthma	high
**Veigi et al[417]**	1991	Italy	cross-sectional	3289	46.1	8-64	LPG	no LPG	respiratory symptoms	high
**Venn et al[418]**	2001	Ethiopia	cross-sectional	9844	47	18.0	non-biomass fuel	biomass fuel	respiratory symptoms	high
**Victora et al[419]**	1994	Brazil	case-control	1032	54.1	under 2	woodstove, fireplace, open-air fire	no woodstove, fireplace, open-air fire	pneumonia	high
**Viegi et al[420]**	1992	Italy	cross-sectional	3185	38.8	not reported	bottled gas, wood, kerosene, oil, coal	natural gas	respiratory symptoms and physician diagnosed respiratory conditions	high
**Volkmer et al[421]**	1995	Australia	cross-sectional	14124	NA	4-5	natural gas, wood	no natural gas or wood	asthma, wheeze, bronchitis, dry cough	high
**von Maffei et al[422]**	2001	USA	cross-sectional	2409	NA	under 18	gas, kerosene, wood, coal, fireplace	no gas, kerosene, wood, coal, fireplace	asthma	high
**von Mutius et al[423]**	1996	Germany	cross-sectional	1958	NA	11	coal or wood	no coal or wood	asthma	high
**Wafula et al[424]**	2000	Kenya	case-control	648	NA	women between 15 and 60 years and children under 5 years	traditional 3 stone stove	improved stove	acute respiratory infection	high
**Walker et al[425]**	2020	Honduras	cross-sectional	150	0	37.0	Traditional stove, older Justa stove, newer Justa stove, PM concentration	Justa stove, newer Justa stove	Respiratory symptoms	high
**Wang et al[426]**	1992	China	case-control	308	NA	not reported	coal	no coal	lung cancer	high
**Wang et al[427]**	1996	China	case-control	270	0	not reported	coal, kang	no coal, kang	lung cancer	high
**Wang et al[428]**	2013	China	cross-sectional	4618	53.7	4.7	gas or electricity	coal or wood	asthma, pneumonia and wheeze	high
**Ware et al[429]**	1984	USA	prospective cohort study	8384	NA	6-7	gas	no gas	bronchitis, respiratory illness before age of 2	high
**Ware et al[430]**	2014	USA	cross-sectional	561	53.6	under 18	woodstove	fuel oil	pneumonia, asthma, bronchitis, wheeze, cough	high
**Wayse et al[431]**	2004	India	case-control	150	59.3	2.0	liquid petroleum gas	biomass	severe acute lower respiratory infection	moderate
**Weber et al[432]**	2020	Ghana	prospective cohort study	1010	0	28.3	polluting fuel	Clean fuel	perinatal mortality, low birth weight, stillbirth	low
**Wichmann et al[433]**	2006	South Africa	cross-sectional	4679	50.4	under 5	smoke pollution fuels	clean fuels	acute lower respiratory infection	high
**Wichmann et al[434]**	2006	South Africa	cross-sectional	3556	0	not reported	polluting fuel	clean fuel	under-five mortality	high
**Wichmann et al[435]**	2009	South Africa	cross-sectional	2437	50.4	5.0	combination of dirty and clean fuel	clean fuel	wheeze	high

**Willers et al[436]**	2006	the Netherlands	cohort study	3148	NA	6-7	gas	electricity	respiratory symptoms	high
**Withers et al[437]**	1998	United Kingdom	prospective cohort study	2289	NA	14-16	gas	no gas	wheeze	high
**Woldeamanuel et al[438]**	2019	Ethiopia	cross-sectional	779	57.4	over 30	biomass fuel	no biomass fuel	COPD	moderate
**Woldesemayat et al[439]**	2014	Ethiopia	case-control	1159	58.9	33.6	biomass	no biomass	tuberculosis	high
**Wolff et al[440]**	2012	Madagascar	cross-sectional	1236	48.4	10.3	electricity	charcoal or gas	asthma	high
**Wong et al[441]**	2004	China	cross-sectional	10902	51.3	10.0	gas	no gas	current wheeze	high
**Wong et al[442]**	2013	47 countries	cross-sectional	512707	NA	6-7 and 13-14	open fire	not open fire	asthma and respiratory symptoms	high
**Wong et al[443]**	2019	China	case-control	1524	0	54.7	ever smoky coal users	Wood/dung/Smokeless coal users from Smokeless coal regions	lung cancer	moderate
**Wu et al[444]**	1985	USA	case-control	440	NA	60.2	coal	no coal	lung cancer	high
**Wu et al[445]**	2010	China	cross-sectional	197	NA	not reported	gas stove and CO, NO and NO2 concentrations	no gas stove	asthma and bronchitis	high
**Wu et al[446]**	2020	China	cross-sectional	2673	49	not reported	Coal, Wood, Natural gas, Electricity, Coal Gas, Compressed petroleum gas	NA	pneumonia	high
**Wu-Williams et al[447]**	1990	China	case-control	850	0	not reported	kang stove, coal stove, coal burners, central heating	no kang stove, coal stove, coal burners, central heating	lung cancer	low
**Wylie et al[448]**	2015	India	cross-sectional	1744	0	not reported	wood	LPG	stillbirth, birth weight and low birth weight	low
**Xu et al[449]**	1989	China	case-control	2594	58.5	57.8	kang stove, coal stove, gas central heating, gas fuel for cooking	no kang stove, coal stove, gas central heating, gas fuel for cooking	lung cancer	high
**Xu et al[450]**	1993	China	cross-sectional	1576	75.1	not reported	coal	no coal	asthma, bronchitis, respiratory symptoms	high
**Xu et al[451]**	1996	China	cross-sectional	28946	43	not reported	coal	no coal	wheeze and asthma	high
**Yang et al[452]**	1997	Taiwan	cross-sectional	4164	48.9	10.4	gas	no exposure to gas	asthma, cough, wheeze, bronchitis, allergic rhinitis	high
**Yang et al[453]**	2014	China	case-control	6246	66.3	not reported	biomass	no biomass	COPD and lung cancer	high
**Yu et al[454]**	2017	Hong Kong	prospective cohort study	543	51.9	under 1.5	gas	electricity	wheeze	high
**Yu et al[455]**	2020	China	prospective cohort study	171677	29.3	30-79	solid fuel	clean fuel	All-cause mortality, cardiovascular mortality, respiratory mortality	high
**Yucra et al[456]**	2011	Peru	case-control	190	NA	27.1	wood, peat/champa, charcoal or dung	gas propane, electricity, kerosene	low birth weight	moderate
**Yusuf et al[457]**	2020	Canada, Saudi Arabia, Sweden, and United Arab Emirates, Argentina, Brazil, Chile, China, Colombia, Iran, Malaysia, Palestine, Philippines, Poland, Turkey, and South Africa, Bangladesh, India, Pakistan, Tanzania, and Zimbabwe	prospective cohort study	155722	41.7	50.2	kerosene/solid fuel	clean fuel	cardiovascular disease, all-cause death	low

**Zacharasiewicz et al[458]**	1999	Austria	retrospective cohort study	28747	51.13	7.2	not electric	electric	wheeze	high
**Zejda et al[459]**	2003	Poland	prospective cohort study	663	49.3	7-9	coal	no coal	asthma	high
**Zha et al[460]**	2019	China	cross-sectional study	2770	49.2	53.8	coal/biomass	no coal/biomass	COPD	low
**Zhang et al[461]**	1988	China	prospective cohort study	957	100	not reported	coal	no coal	stroke mortality	low
**Zhang et al[462]**	2002	China	cross-sectional	22528	48.7	not reported	coal, wood, cornstalk	no coal, wood, cornstalk	respiratory symptoms	high
**Zhao et al[463]**	2009	China	case-control	206	0	62.1	coal or wood	no coal or wood	chronic bronchitis	high
**Zheng et al[464]**	2002	China	cross-sectional	1209	58.7	6-10	LPG, coal	electricity	asthma	high
**Zheng et al[465]**	2013	China	cross-sectional	4014	51.2	5.0	coal or natural gas	electricty	pneumonia	high
**Zhong et al[466]**	1999	China	case-control	1105	0	35 to 69	coal, gas	coal	lung cancer	low
**Zhong et al[467]**	2007	China	cross-sectional	20245	43	over 40	biomass	no exposure to biomass	COPD	low
**Zhou et al[468]**	1994	China	cross-sectional	624	NA	under 3	gas	coal	pneumonia	high
**Zhou et al[469]**	1995	China	cross-sectional	10892	48.3	not reported	smoky coal and smokeless coal	wood	COPD	high
**Zhou et al[470]**	2009	China	cross-sectional	12471	17.9	over 40	wood, charcoal, grass and crop residues or dung, or coal	no biomass	COPD	low
**Zhou et al[471]**	2013	France	prospective cohort study	1765	52	under 1	wood	no wood	bronchiolitis	high
**Zhou et al[472]**	2014	China	prospective cohort study	682	45.6	54.4	clean fuel	biomass	COPD	low

Table S4: Trends in cause-specific burden of disease attributable to household air pollution from 2000-2017
(DALYs in thousands [95% CI])

Year	Atherosclerotic cardiovascular disease	Lung cancer	Chronic respiratory disease	Communicable respiratory disease
**2000**	9312 [5394 - 14090]	4361 [2516 - 6441]	20614 [12581 - 30158]	52456 [33818 - 73140]
**2001**	9259 [5381 - 13945]	4388 [2576 - 6457]	20127 [12338 - 29429]	50490 [32451 - 70419]
**2002**	9308 [5469 - 13969]	4438 [2603 - 6536]	19813 [12254 - 28723]	48562 [31380 - 67779]
**2003**	9193 [5345 - 13862]	4429 [2587 - 6507]	19235 [11749 - 27913]	46463 [29818 - 64933]
**2004**	9205 [5398 - 13824]	4493 [2663 - 6610]	18783 [11670 - 27307]	44867 [28947 - 62690]
**2005**	9175 [5312 - 13877]	4491 [2622 - 6672]	18317 [11259 - 26612]	43158 [27627 - 60481]
**2006**	9021 [5262 - 13647]	4452 [2577 - 6600]	17914 [10955 - 26149]	41559 [26657 - 58376]
**2007**	9202 [5322 - 13893]	4589 [2675 - 6822]	18183 [11149 - 26357]	40328 [25879 - 56639]
**2008**	9195 [5368 - 13926]	4596 [2678 - 6906]	18138 [11179 - 26436]	38724 [24679 - 54668]
**2009**	9264 [5307 - 14195]	4683 [2626 - 7126]	18204 [11001 - 26715]	37184 [23545 - 52883]
**2010**	9307 [5371 - 14280]	4765 [2664 - 7247]	18248 [11080 - 26732]	35791 [22619 - 50917]
**2011**	9439 [5374 - 14589]	4850 [2683 - 7398]	18338 [11034 - 27014]	34801 [21833 - 49709]
**2012**	9402 [5219 - 14755]	4915 [2638 - 7666]	18102 [10750 - 26791]	33488 [20815 - 48195]
**2013**	9487 [5236 - 14868]	5091 [2693 - 7930]	18170 [10678 - 27045]	32266 [19950 - 46651]
**2014**	9480 [5057 - 15149]	5192 [2663 - 8182]	18248 [10537 - 27260]	31072 [19006 - 45322]
**2015**	9444 [4955 - 15280]	5329 [2589 - 8577]	18126 [10303 - 27428]	29717 [18037 - 43755]
**2016**	9549 [4933 - 15604]	5567 [2767 - 8936]	18260 [10247 - 27684]	28497 [17158 - 42328]
**2017**	9539 [4979 - 15570]	5535 [2762 - 8965]	18390 [10430 - 27815]	27420 [16382 - 40988]

Table S5: Trends in burden of disease attributable to household air pollution stratified by 2018 World Bank income groups from 2000-2017
(DALYs in thousands [95% CI])

Year	Low income	Lower middle income	Upper middle income	High income
**2000**	16260 [10820 - 22524]	51536 [33940 - 71133]	18806 [9513 - 29769]	142 [35 - 403]
**2001**	16052 [10657 - 22206]	50096 [32847 - 69290]	17977 [9206 - 28363]	139 [36 - 392]
**2002**	15745 [10449 - 21819]	48891 [32248 - 67518]	17350 [8975 - 27291]	136 [36 - 379]
**2003**	15625 [10400 - 21627]	47001 [30601 - 65041]	16565 [8464 - 26183]	130 [33 - 365]
**2004**	15355 [10226 - 21255]	45723 [29936 - 63353]	16145 [8486 - 25462]	124 [30 - 363]
**2005**	15112 [10046 - 20912]	44458 [28824 - 61731]	15467 [7928 - 24653]	104 [22 - 345]
**2006**	14950 [9942 - 20716]	43531 [28254 - 60562]	14361 [7232 - 23155]	103 [22 - 338]
**2007**	14778 [9842 - 20464]	43109 [27923 - 59971]	14316 [7240 - 22934]	101 [19 - 342]
**2008**	14552 [9633 - 20204]	42131 [27148 - 58868]	13878 [7106 - 22532]	93 [16 - 332]
**2009**	14375 [9502 - 20013]	40963 [26274 - 57551]	13905 [6687 - 23016]	93 [15 - 337]
**2010**	14176 [9324 - 19784]	40124 [25692 - 56392]	13720 [6704 - 22662]	90 [14 - 337]
**2011**	13999 [9198 - 19546]	39765 [25249 - 56201]	13578 [6466 - 22625]	87 [12 - 339]
**2012**	13835 [9037 - 19374]	38731 [24308 - 55067]	13253 [6066 - 22614]	88 [11 - 352]
**2013**	13669 [8927 - 19166]	37824 [23642 - 54119]	13432 [5976 - 22849]	90 [11 - 359]
**2014**	13506 [8787 - 19029]	37193 [22867 - 53580]	13206 [5600 - 22942]	88 [10 - 363]
**2015**	13353 [8635 - 18867]	35943 [22061 - 52243]	13234 [5180 - 23559]	87 [8 - 371]
**2016**	13114 [8458 - 18628]	34976 [21106 - 51460]	13694 [5533 - 24086]	89 [8 - 378]
**2017**	12966 [8337 - 18438]	34397 [20671 - 50815]	13430 [5537 - 23688]	91 [7 - 397]

Table S6: Trends in burden of disease attributable to household air pollution stratified by WHO regions from 2000-2017
(DALYs in thousands [95% CI])

Year	African region	Region of the Americas	South-East Asia region	European region	Eastern Mediterranean region	Western Pacific region
**2000**	23506 [15657 - 32475]	1636 [951 - 2612]	36011 [23560 - 49616]	1946 [731 - 3959]	5704 [3628 - 8335]	17942 [9781 - 26834]
**2001**	23222 [15438 - 32036]	1588 [906 - 2519]	34875 [22688 - 48182]	1729 [651 - 3654]	5494 [3499 - 8023]	17356 [9565 - 25836]
**2002**	22867 [15201 - 31643]	1489 [845 - 2425]	33925 [22208 - 46740]	1627 [622 - 3431]	5332 [3426 - 7751]	16881 [9405 - 25017]
**2003**	22552 [14965 - 31247]	1445 [823 - 2352]	32284 [20773 - 44612]	1532 [581 - 3193]	5291 [3420 - 7643]	16216 [8936 - 24169]
**2004**	22159 [14733 - 30638]	1358 [764 - 2251]	31278 [20272 - 43319]	1412 [521 - 3016]	5167 [3344 - 7491]	15976 [9045 - 23718]
**2005**	21760 [14420 - 30162]	1298 [732 - 2159]	30314 [19383 - 42070]	1329 [465 - 2923]	5043 [3268 - 7247]	15398 [8551 - 23081]
**2006**	21635 [14430 - 29938]	1254 [707 - 2094]	29512 [18813 - 41124]	1215 [430 - 2651]	4894 [3181 - 7060]	14435 [7891 - 21904]
**2007**	21369 [14290 - 29510]	1198 [656 - 2064]	29368 [18659 - 41000]	1156 [402 - 2546]	4703 [3057 - 6753]	14510 [7960 - 21839]
**2008**	20893 [13887 - 28944]	1167 [640 - 2004]	28710 [18131 - 40247]	979 [329 - 2372]	4650 [3016 - 6711]	14254 [7901 - 21657]
**2009**	20590 [13668 - 28585]	1153 [624 - 1999]	27820 [17456 - 39255]	918 [295 - 2298]	4439 [2875 - 6472]	14415 [7561 - 22311]
**2010**	20211 [13387 - 28094]	1090 [582 - 1926]	27295 [17117 - 38497]	843 [262 - 2132]	4333 [2776 - 6336]	14337 [7609 - 22191]
**2011**	19891 [13160 - 27680]	1064 [564 - 1889]	27244 [16896 - 38673]	774 [224 - 2017]	4190 [2683 - 6148]	14266 [7397 - 22302]
**2012**	19650 [12919 - 27416]	1012 [525 - 1866]	26392 [16144 - 37673]	738 [197 - 1979]	4082 [2582 - 6073]	14032 [7056 - 22400]
**2013**	19332 [12679 - 27007]	991 [512 - 1829]	25787 [15748 - 37086]	707 [185 - 1919]	3949 [2467 - 5919]	14248 [6966 - 22733]
**2014**	19091 [12479 - 26787]	967 [492 - 1810]	25292 [15100 - 36679]	662 [168 - 1860]	3900 [2401 - 5894]	14081 [6623 - 22884]
**2015**	18884 [12282 - 26661]	956 [483 - 1796]	24293 [14522 - 35449]	645 [155 - 1852]	3752 [2278 - 5755]	14087 [6165 - 23527]
**2016**	18677 [12088 - 26539]	938 [466 - 1789]	23519 [13757 - 34748]	584 [117 - 1847]	3598 [2162 - 5566]	14555 [6515 - 24063]
**2017**	18348 [11797 - 26152]	946 [466 - 1830]	23282 [13665 - 34413]	558 [103 - 1810]	3532 [2077 - 5529]	14218 [6445 - 23604]

Table S7: Country-level estimates of burden of disease and deaths attributable to household air pollution in 2017

Country	DALYs (in thousands, [95% CI])	Age-standardized DALYs (per 100,000, [95% CI])	Deaths (in thousands, [95% CI])	Age-standardized Deaths (per 100,000, [95% CI])
**Afghanistan**	561 [373 - 799]	2121 [1413 - 3003]	18.5 [12.3 - 26.3]	45 [27 - 66]
**Albania**	10 [2 - 23]	267 [61 - 611]	0.3 [0.1 - 0.7]	6 [1 - 16]
**Algeria**	167 [0 - 347]	482 [1 - 998]	5 [0 - 11.9]	12 [0 - 29]
**Andorra**	0 [0 - 0]	0 [0 - 0]	0 [0 - 0]	0 [0 - 0]
**Angola**	282 [165 - 428]	1362 [795 - 2040]	10.9 [7.1 - 15.8]	28 [19 - 40]
**Antigua and Barbuda**	0 [0 - 0]	12 [1 - 50]	0 [0 - 0]	0 [0 - 1]
**Argentina**	11 [1 - 48]	21 [2 - 93]	0.3 [0 - 1.2]	0 [0 - 2]
**Armenia**	2 [0 - 8]	41 [2 - 200]	0 [0 - 0.2]	1 [0 - 4]
**Australia**	0 [0 - 0]	0 [0 - 0]	0 [0 - 0]	0 [0 - 0]
**Austria**	0 [0 - 0]	0 [0 - 0]	0 [0 - 0]	0 [0 - 0]
**Azerbaijan**	6 [0 - 27]	63 [3 - 312]	0.1 [0 - 0.6]	1 [0 - 6]
**Bahamas**	0 [0 - 0]	0 [0 - 0]	0 [0 - 0]	0 [0 - 0]
**Bahrain**	0 [0 - 0]	0 [0 - 0]	0 [0 - 0]	0 [0 - 0]
**Bangladesh**	2048 [1423 - 2732]	1590 [1106 - 2126]	54.7 [37.9 - 73.6]	33 [23 - 45]
**Barbados**	0 [0 - 0]	8 [1 - 28]	0 [0 - 0]	0 [0 - 1]
**Belarus**	13 [0 - 69]	83 [2 - 451]	0.4 [0 - 2.1]	2 [0 - 13]
**Belgium**	0 [0 - 0]	0 [0 - 0]	0 [0 - 0]	0 [0 - 0]
**Belize**	1 [0 - 1]	177 [85 - 315]	0 [0 - 0]	3 [2 - 6]
**Benin**	190 [126 - 270]	2087 [1394 - 2891]	8.7 [6.1 - 11.9]	45 [33 - 60]
**Bhutan**	3 [1 - 6]	393 [114 - 862]	0.1 [0 - 0.1]	8 [2 - 18]
**Bolivia (Plurinational State of)**	28 [14 - 48]	279 [143 - 480]	0.8 [0.4 - 1.4]	6 [3 - 10]
**Bosnia and Herzegovina**	28 [15 - 45]	499 [257 - 802]	0.7 [0.3 - 1.2]	12 [6 - 21]
**Botswana**	14 [7 - 22]	877 [452 - 1392]	0.3 [0.2 - 0.5]	18 [9 - 29]
**Brazil**	137 [30 - 382]	63 [14 - 175]	3.1 [0.7 - 8.7]	1 [0 - 3]
**Brunei Darussalam**	0 [0 - 0]	0 [0 - 0]	0 [0 - 0]	0 [0 - 0]
**Bulgaria**	15 [0 - 80]	118 [2 - 616]	0.4 [0 - 2.3]	3 [0 - 15]

**Burkina Faso**	474 [314 - 669]	2390 [1600 - 3260]	21.1 [15 - 28.7]	48 [36 - 61]
**Burundi**	258 [158 - 372]	3177 [1913 - 4568]	9.5 [6.8 - 12.7]	73 [54 - 94]
**Cabo Verde**	2 [1 - 4]	454 [198 - 782]	0.1 [0 - 0.1]	10 [4 - 19]
**Cambodia**	230 [156 - 313]	1771 [1202 - 2389]	5.7 [4 - 7.7]	39 [28 - 51]
**Cameroon**	360 [229 - 522]	1889 [1198 - 2714]	13.9 [9.5 - 19.4]	42 [29 - 58]
**Canada**	0 [0 - 0]	0 [0 - 0]	0 [0 - 0]	0 [0 - 0]
**Central African Republic**	191 [110 - 287]	5152 [3038 - 7584]	6.9 [4.8 - 9.7]	103 [75 - 139]
**Chad**	524 [358 - 719]	3285 [2196 - 4457]	20.9 [15.4 - 27.5]	64 [48 - 81]
**Chile**	13 [2 - 39]	57 [9 - 179]	0.3 [0 - 1]	1 [0 - 4]
**China**	11699 [4974 - 19608]	649 [274 - 1089]	267.9 [103.6 - 477.8]	15 [6 - 27]
**Colombia**	28 [5 - 83]	55 [10 - 160]	0.7 [0.1 - 2]	1 [0 - 3]
**Comoros**	10 [6 - 14]	1779 [1136 - 2523]	0.3 [0.2 - 0.4]	42 [29 - 57]
**Congo**	62 [36 - 92]	1852 [1109 - 2721]	2 [1.3 - 2.9]	44 [29 - 62]
**Costa Rica**	2 [0 - 6]	45 [8 - 132]	0 [0 - 0.1]	1 [0 - 3]
**Cote d'Ivoire**	392 [244 - 569]	2023 [1267 - 2894]	15.5 [10.3 - 21.5]	44 [29 - 61]
**Croatia**	7 [1 - 21]	85 [13 - 261]	0.2 [0 - 0.5]	2 [0 - 6]
**Cuba**	47 [0 - 164]	276 [1 - 950]	1.1 [0 - 4.6]	6 [0 - 23]
**Cyprus**	0 [0 - 0]	0 [0 - 0]	0 [0 - 0]	0 [0 - 0]
**Czechia**	5 [0 - 22]	24 [1 - 114]	0.1 [0 - 0.5]	0 [0 - 2]
**Democratic People's Republic of Korea**	646 [424 - 891]	2268 [1491 - 3130]	14.7 [9.3 - 20.9]	49 [31 - 70]
**Democratic Republic of the Congo**	1923 [1206 - 2779]	2977 [1850 - 4324]	73.3 [52 - 99.8]	66 [45 - 91]
**Denmark**	0 [0 - 0]	0 [0 - 0]	0 [0 - 0]	0 [0 - 0]
**Djibouti**	13 [7 - 21]	1592 [909 - 2504]	0.4 [0.2 - 0.6]	37 [23 - 57]
**Dominica**	0 [0 - 0]	109 [25 - 280]	0 [0 - 0]	2 [0 - 5]
**Dominican Republic**	11 [5 - 23]	120 [49 - 244]	0.4 [0.1 - 0.7]	3 [1 - 5]
**Ecuador**	3 [0 - 15]	21 [1 - 99]	0.1 [0 - 0.4]	0 [0 - 2]
**Egypt**	18 [2 - 71]	25 [2 - 98]	0.4 [0 - 1.5]	1 [0 - 2]
**El Salvador**	8 [3 - 15]	132 [55 - 258]	0.2 [0.1 - 0.4]	3 [1 - 6]
**Equatorial Guinea**	9 [4 - 16]	1238 [464 - 2074]	0.4 [0.2 - 0.7]	29 [11 - 48]

**Eritrea**	125 [63 - 197]	2995 [1524 - 4574]	3.6 [2 - 5.3]	65 [38 - 93]
**Estonia**	1 [0 - 6]	51 [1 - 270]	0 [0 - 0.2]	1 [0 - 6]
**Ethiopia**	1411 [951 - 1886]	1798 [1173 - 2411]	56.7 [43 - 71.4]	40 [31 - 50]
**Fiji**	8 [3 - 14]	1083 [351 - 1770]	0.2 [0.1 - 0.4]	25 [7 - 44]
**Finland**	0 [0 - 0]	0 [0 - 0]	0 [0 - 0]	0 [0 - 0]
**France**	0 [0 - 0]	0 [0 - 0]	0 [0 - 0]	0 [0 - 0]
**Gabon**	8 [2 - 18]	626 [129 - 1413]	0.3 [0 - 0.6]	13 [3 - 31]
**Gambia**	31 [20 - 43]	2181 [1477 - 2977]	1.1 [0.8 - 1.6]	54 [38 - 72]
**Georgia**	19 [6 - 38]	342 [118 - 694]	0.5 [0.2 - 1.2]	9 [3 - 19]
**Germany**	0 [0 - 0]	0 [0 - 0]	0 [0 - 0]	0 [0 - 0]
**Ghana**	332 [217 - 465]	1624 [1061 - 2256]	12.4 [8.6 - 16.9]	39 [28 - 52]
**Greece**	3 [0 - 14]	16 [1 - 66]	0.1 [0 - 0.3]	0 [0 - 1]
**Grenada**	0 [0 - 0]	26 [1 - 123]	0 [0 - 0]	1 [0 - 3]
**Guatemala**	122 [85 - 168]	833 [584 - 1123]	3.2 [2.2 - 4.6]	16 [11 - 21]
**Guinea**	320 [216 - 441]	3139 [2092 - 4298]	12.3 [8.8 - 16.3]	68 [50 - 89]
**Guinea-Bissau**	35 [23 - 49]	2878 [1876 - 3954]	1.5 [1.1 - 2]	70 [51 - 91]
**Guyana**	2 [1 - 4]	341 [140 - 643]	0.1 [0 - 0.1]	8 [3 - 16]
**Haiti**	191 [128 - 269]	2016 [1380 - 2778]	6.6 [4.5 - 9.2]	45 [29 - 64]
**Honduras**	35 [20 - 54]	516 [302 - 784]	1 [0.6 - 1.6]	12 [6 - 19]
**Hungary**	0 [0 - 0]	0 [0 - 0]	0 [0 - 0]	0 [0 - 0]
**Iceland**	0 [0 - 0]	0 [0 - 0]	0 [0 - 0]	0 [0 - 0]
**India**	17264 [10041 - 25482]	1552 [901 - 2272]	422.5 [250.7 - 614]	32 [19 - 47]
**Indonesia**	1598 [716 - 2768]	741 [335 - 1272]	39.5 [18.1 - 67.4]	17 [8 - 30]
**Iran (Islamic Republic of)**	10 [1 - 41]	14 [1 - 57]	0.3 [0 - 1.1]	0 [0 - 1]
**Iraq**	5 [0 - 21]	15 [1 - 68]	0.2 [0 - 0.7]	0 [0 - 1]
**Ireland**	0 [0 - 0]	0 [0 - 0]	0 [0 - 0]	0 [0 - 0]
**Israel**	0 [0 - 0]	0 [0 - 0]	0 [0 - 0]	0 [0 - 0]
**Italy**	0 [0 - 0]	0 [0 - 0]	0 [0 - 0]	0 [0 - 0]
**Jamaica**	3 [1 - 6]	93 [33 - 204]	0.1 [0 - 0.1]	2 [1 - 4]

**Japan**	0 [0 - 0]	0 [0 - 0]	0 [0 - 0]	0 [0 - 0]
**Jordan**	0 [0 - 2]	7 [1 - 23]	0 [0 - 0]	0 [0 - 0]
**Kazakhstan**	8 [0 - 41]	49 [2 - 243]	0.2 [0 - 1]	1 [0 - 6]
**Kenya**	590 [394 - 796]	1725 [1135 - 2337]	18.6 [13.8 - 23.7]	38 [28 - 49]
**Kiribati**	2 [1 - 3]	2578 [1564 - 3599]	0.1 [0 - 0.1]	60 [40 - 81]
**Kuwait**	0 [0 - 0]	0 [0 - 0]	0 [0 - 0]	0 [0 - 0]
**Kyrgyzstan**	14 [3 - 37]	291 [54 - 750]	0.4 [0.1 - 1.1]	7 [1 - 19]
**Lao People's Democratic Republic**	145 [95 - 203]	2566 [1705 - 3541]	4 [2.7 - 5.6]	51 [34 - 71]
**Latvia**	4 [0 - 14]	116 [13 - 382]	0.1 [0 - 0.4]	3 [0 - 10]
**Lesotho**	44 [24 - 67]	2891 [1659 - 4357]	1.1 [0.7 - 1.5]	57 [36 - 81]
**Liberia**	67 [43 - 95]	1996 [1308 - 2752]	3 [2.1 - 4.1]	47 [34 - 62]
**Lithuania**	0 [0 - 0]	0 [0 - 0]	0 [0 - 0]	0 [0 - 0]
**Luxembourg**	0 [0 - 0]	0 [0 - 0]	0 [0 - 0]	0 [0 - 0]
**Madagascar**	576 [384 - 807]	2684 [1840 - 3627]	20.8 [14.4 - 29]	56 [40 - 75]
**Malawi**	272 [174 - 380]	2077 [1347 - 2860]	11.4 [8.2 - 15.2]	46 [34 - 60]
**Malaysia**	60 [1 - 288]	234 [2 - 1106]	1.3 [0 - 6.8]	5 [0 - 29]
**Maldives**	0 [0 - 1]	54 [1 - 285]	0 [0 - 0]	1 [0 - 6]
**Mali**	372 [248 - 532]	2030 [1362 - 2825]	24.5 [17.3 - 33.3]	41 [28 - 56]
**Malta**	0 [0 - 0]	0 [0 - 0]	0 [0 - 0]	0 [0 - 0]
**Marshall Islands**	0 [0 - 1]	1123 [412 - 2105]	0 [0 - 0]	24 [8 - 47]
**Mauritania**	29 [18 - 43]	1001 [631 - 1453]	1 [0.7 - 1.5]	22 [14 - 33]
**Mauritius**	0 [0 - 2]	28 [1 - 138]	0 [0 - 0]	1 [0 - 3]
**Mexico**	171 [97 - 276]	149 [85 - 237]	4.2 [2.4 - 6.8]	3 [2 - 5]
**Micronesia (Federated States of)**	2 [1 - 2]	1918 [1291 - 2657]	0 [0 - 0]	45 [28 - 63]
**Mongolia**	34 [18 - 50]	1276 [675 - 1879]	0.9 [0.5 - 1.3]	31 [15 - 48]
**Montenegro**	4 [2 - 8]	442 [178 - 801]	0.1 [0 - 0.2]	11 [4 - 22]
**Morocco**	9 [1 - 43]	29 [2 - 133]	0.2 [0 - 1.1]	1 [0 - 3]
**Mozambique**	513 [305 - 744]	2637 [1568 - 3743]	21.6 [15.7 - 29]	61 [45 - 80]
**Myanmar**	912 [584 - 1278]	2056 [1330 - 2869]	23 [15.5 - 31.8]	43 [29 - 59]

**Namibia**	28 [15 - 43]	1606 [841 - 2390]	0.8 [0.4 - 1.2]	33 [18 - 48]
**Nepal**	392 [257 - 560]	1691 [1113 - 2395]	9.5 [6.3 - 13.5]	35 [23 - 50]
**Netherlands**	0 [0 - 0]	0 [0 - 0]	0 [0 - 0]	0 [0 - 0]
**New Zealand**	0 [0 - 0]	0 [0 - 0]	0 [0 - 0]	0 [0 - 0]
**Nicaragua**	20 [13 - 28]	379 [253 - 526]	0.5 [0.4 - 0.8]	7 [5 - 11]
**Niger**	530 [350 - 765]	2501 [1632 - 3535]	24.1 [17.2 - 32.6]	50 [35 - 68]
**Nigeria**	5018 [3391 - 7053]	2201 [1458 - 3127]	194.8 [139.3 - 262.7]	37 [26 - 51]
**Norway**	0 [0 - 0]	0 [0 - 0]	0 [0 - 0]	0 [0 - 0]
**Oman**	2 [0 - 9]	67 [1 - 366]	0 [0 - 0.2]	2 [0 - 10]
**Pakistan**	2055 [1192 - 3135]	1350 [796 - 2023]	70.2 [42.3 - 106.2]	29 [17 - 45]
**Panama**	4 [1 - 7]	94 [37 - 192]	0.1 [0 - 0.2]	2 [1 - 3]
**Papua New Guinea**	273 [180 - 384]	4632 [3108 - 6440]	6.2 [4.1 - 8.8]	97 [65 - 134]
**Paraguay**	20 [12 - 31]	359 [221 - 538]	0.5 [0.3 - 0.8]	8 [5 - 12]
**Peru**	80 [46 - 125]	248 [144 - 389]	2.1 [1.3 - 3.3]	5 [3 - 7]
**Philippines**	1223 [737 - 1779]	1510 [924 - 2174]	28.4 [17.8 - 40.6]	35 [22 - 49]
**Poland**	0 [0 - 0]	0 [0 - 0]	0 [0 - 0]	0 [0 - 0]
**Portugal**	0 [0 - 0]	0 [0 - 0]	0 [0 - 0]	0 [0 - 0]
**Qatar**	0 [0 - 1]	10 [1 - 40]	0 [0 - 0]	0 [0 - 1]
**Republic of Korea**	25 [1 - 130]	31 [1 - 163]	0.5 [0 - 2.7]	1 [0 - 3]
**Republic of Moldova**	6 [1 - 17]	112 [18 - 345]	0.1 [0 - 0.5]	2 [0 - 8]
**Romania**	76 [11 - 214]	245 [35 - 677]	1.9 [0.3 - 5.7]	5 [1 - 15]
**Russian Federation**	72 [4 - 343]	33 [2 - 156]	1.8 [0.1 - 8.7]	1 [0 - 4]
**Rwanda**	188 [119 - 269]	1944 [1247 - 2715]	6.3 [4.6 - 8.6]	43 [32 - 55]
**Saint Lucia**	0 [0 - 0]	20 [1 - 93]	0 [0 - 0]	0 [0 - 2]
**Saint Vincent and the Grenadines**	0 [0 - 0]	24 [1 - 113]	0 [0 - 0]	0 [0 - 2]
**Samoa**	2 [1 - 2]	1098 [732 - 1534]	0 [0 - 0.1]	27 [16 - 39]
**Saudi Arabia**	10 [0 - 53]	49 [1 - 262]	0.2 [0 - 1.1]	1 [0 - 7]
**Senegal**	166 [103 - 241]	1511 [942 - 2159]	5.8 [3.8 - 8.2]	34 [23 - 47]
**Serbia**	65 [18 - 134]	429 [114 - 896]	1.7 [0.4 - 4]	11 [3 - 25]

**Seychelles**	0 [0 - 0]	54 [2 - 275]	0 [0 - 0]	1 [0 - 6]
**Sierra Leone**	195 [129 - 273]	2981 [1978 - 4085]	8.4 [6 - 11.4]	64 [47 - 82]
**Singapore**	0 [0 - 0]	0 [0 - 0]	0 [0 - 0]	0 [0 - 0]
**Slovakia**	2 [0 - 12]	29 [1 - 144]	0.1 [0 - 0.3]	1 [0 - 3]
**Slovenia**	1 [0 - 6]	29 [1 - 145]	0 [0 - 0.1]	1 [0 - 3]
**Solomon Islands**	11 [8 - 15]	2697 [1927 - 3552]	0.3 [0.2 - 0.4]	65 [45 - 87]
**Somalia**	435 [254 - 677]	3291 [1921 - 5056]	16.4 [11.2 - 23.4]	75 [50 - 106]
**South Africa**	181 [74 - 351]	378 [156 - 731]	3.9 [1.7 - 7.2]	6 [3 - 12]
**South Sudan**	353 [230 - 508]	3504 [2216 - 5085]	12.8 [9 - 17.3]	65 [45 - 90]
**Spain**	0 [0 - 0]	0 [0 - 0]	0 [0 - 0]	0 [0 - 0]
**Sri Lanka**	165 [108 - 230]	706 [464 - 978]	4.5 [2.9 - 6.5]	20 [13 - 28]
**Sudan**	271 [167 - 412]	950 [600 - 1407]	12.4 [7.6 - 18.9]	23 [13 - 36]
**Suriname**	1 [0 - 2]	135 [41 - 311]	0 [0 - 0]	3 [1 - 6]
**Swaziland**	13 [8 - 20]	1597 [940 - 2425]	0.3 [0.2 - 0.4]	30 [19 - 44]
**Sweden**	0 [0 - 0]	0 [0 - 0]	0 [0 - 0]	0 [0 - 0]
**Switzerland**	0 [0 - 0]	0 [0 - 0]	0 [0 - 0]	0 [0 - 0]
**Syrian Arab Republic**	2 [0 - 9]	16 [1 - 64]	0.1 [0 - 0.2]	0 [0 - 2]
**Tajikistan**	38 [10 - 85]	444 [124 - 994]	0.9 [0.3 - 2.1]	8 [2 - 19]
**Thailand**	235 [100 - 440]	260 [112 - 486]	5 [2.1 - 9.3]	5 [2 - 9]
**The former Yugoslav Republic of Macedonia**	14 [7 - 23]	435 [212 - 727]	0.3 [0.1 - 0.6]	10 [4 - 17]
**Timor-Leste**	18 [12 - 26]	1782 [1157 - 2532]	0.5 [0.4 - 0.7]	42 [27 - 60]
**Togo**	103 [67 - 145]	1989 [1313 - 2746]	4.1 [2.9 - 5.7]	47 [34 - 63]
**Tonga**	1 [0 - 1]	822 [449 - 1271]	0 [0 - 0]	17 [9 - 27]
**Trinidad and Tobago**	0 [0 - 1]	11 [1 - 46]	0 [0 - 0]	0 [0 - 1]
**Tunisia**	1 [0 - 3]	8 [1 - 27]	0 [0 - 0.1]	0 [0 - 1]
**Turkey**	51 [14 - 134]	60 [16 - 154]	1 [0.3 - 2.5]	1 [0 - 2]
**Turkmenistan**	1 [0 - 3]	18 [2 - 70]	0 [0 - 0.1]	0 [0 - 2]
**Uganda**	490 [308 - 695]	1864 [1169 - 2616]	23.7 [17.2 - 31.2]	44 [32 - 57]
**Ukraine**	45 [1 - 235]	66 [2 - 338]	1.3 [0 - 7.2]	2 [0 - 9]

**United Arab Emirates**	2 [0 - 8]	32 [2 - 154]	0 [0 - 0.1]	1 [0 - 3]
**United Kingdom**	0 [0 - 0]	0 [0 - 0]	0 [0 - 0]	0 [0 - 0]
**United Republic of Tanzania**	934 [605 - 1304]	2022 [1331 - 2774]	34.9 [24.9 - 47.2]	43 [32 - 54]
**United States of America**	0 [0 - 0]	0 [0 - 0]	0 [0 - 0]	0 [0 - 0]
**Uruguay**	1 [0 - 5]	23 [1 - 105]	0 [0 - 0.1]	0 [0 - 2]
**Uzbekistan**	47 [6 - 149]	186 [25 - 586]	1.1 [0.1 - 3.6]	5 [1 - 16]
**Vanuatu**	5 [3 - 8]	2493 [1631 - 3624]	0.1 [0.1 - 0.2]	57 [35 - 86]
**Venezuela (Bolivarian Republic of)**	6 [0 - 30]	23 [1 - 107]	0.1 [0 - 0.7]	0 [0 - 2]
**Viet Nam**	498 [266 - 802]	552 [296 - 892]	11.1 [6 - 18]	12 [6 - 19]
**Yemen**	138 [80 - 223]	706 [412 - 1115]	5.7 [3.3 - 9.1]	17 [9 - 29]
**Zambia**	255 [157 - 369]	2012 [1265 - 2861]	9.6 [6.6 - 13.3]	44 [32 - 58]
**Zimbabwe**	311 [195 - 441]	2757 [1709 - 3912]	7.8 [5.6 - 10.4]	54 [40 - 72]

References

1. Institute for Health Metrics and Evaluation. (2015) GBD results tool. http://ghdx.healthdata.org/gbd-results-tool [accessed on 14 May 2018].
2. Abusalah A, Gavana M, Haidich AB, et al. Low birth weight and prenatal exposure to indoor pollution from tobacco smoke and wood fuel smoke: a matched case-control study in Gaza Strip. *Matern Child Health J* 2012; **16**(8): 1718–27.
3. Acharya P, Mishra SR, Berg-Beckhoff G. Solid fuel in kitchen and acute respiratory tract infection among under five children: evidence from Nepal demographic and health survey 2011. *J Community Health* 2015; **40**(3): 515–21.
4. Adane MM, Alene GD, Mereta ST, Wanyonyi KL. Prevalence and risk factors of acute lower respiratory infection among children living in biomass fuel using households: a community-based cross-sectional study in Northwest Ethiopia. *BMC public health* 2020; **20**(1): 363.
5. Addo-Yobo EO, Custovic A, Taggart SC, Craven M, Bonnie B, Woodcock A. Risk factors for asthma in urban Ghana. *J Allergy Clin Immunol* 2001; **108**(3): 363–8.
6. Adesanya OA, Chiao C. A multilevel analysis of lifestyle variations in symptoms of acute respiratory infection among young children under five in Nigeria. *Bmc Public Health* 2016; **16**(880).
7. Adeyemi RA, Zewotir T, Ramroop S. Semiparametric Multinomial Ordinal Model to Analyze Spatial Patterns of Child Birth Weight in Nigeria. *International journal of environmental research and public health* 2016; **13**(11).
8. Admasie A, Kumie A, Worku A. Children under Five from Houses of Unclean Fuel Sources and Poorly Ventilated Houses Have Higher Odds of Suffering from Acute Respiratory Infection in Wolaita-Sodo, Southern Ethiopia: A Case-Control Study. *J Environ Public Health* 2018; **2018**(Journal Article): 9320603.
9. Aggarwal AN, Chaudhry K, Chhabra SK, et al. Prevalence and risk factors for bronchial asthma in Indian adults: a multicentre study. *Indian J Chest Dis Allied Sci* 2006; **48**(1): 13–22.
10. Agrawal S. Effect of indoor air pollution from biomass and solid fuel combustion on prevalence of self-reported asthma among adult men and women in India: findings from a nationwide large-scale cross-sectional survey. *J Asthma* 2012; **49**(4): 355–65.
11. Akhtar T, Ullah Z, Khan MH, Nazli R. Chronic bronchitis in women using solid biomass fuel in rural Peshawar, Pakistan. *Chest* 2007; **132**(5): 1472–5.
12. Akinyemi JO, Adedini SA, Wandera SO, Odimegwu CO. Independent and combined effects of maternal smoking and solid fuel on infant and child mortality in sub-Saharan Africa. *Trop Med Int Health* 2016; **21**(12): 1572–82.
13. Akinyemi JO, Morakinyo OM. Household environment and symptoms of childhood acute respiratory tract infections in Nigeria, 2003-2013: a decade of progress and stagnation. *Bmc Infectious Diseases* 2018; **18**.
14. Alam DS, Chowdhury MA, Siddiquee AT, et al. Adult cardiopulmonary mortality and indoor air pollution: a 10-year retrospective cohort study in a low-income rural setting. *Glob Heart* 2012; **7**(3): 215–21.
15. Alam DS, Chowdhury MA, Siddiquee AT, Ahmed S, Clemens JD. Prevalence and Determinants of Chronic Obstructive Pulmonary Disease (COPD) in Bangladesh. *COPD* 2015; **12**(6): 658–67.
16. Aldous MB, Holberg CJ, Wright AL, Martinez FD, Taussig LM. Evaporative cooling and other home factors and lower respiratory tract illness during the first year of life. Group Health Medical Associates. *Am J Epidemiol* 1996; **143**(5): 423–30.
17. Alemayehu M, Alemu K, Sharma HR, Zemicheal G, Abera S. Household fuel use and acute respiratory infections in children under five years of age in Gondar city of Ethiopia. *Journal of Environment and Earth Science* 2014; **4**(7): 77–85.
18. Alemayehu S, Kidanu K, Kahsay T, Kassa M. Risk factors of acute respiratory infections among under five children attending public hospitals in southern Tigray, Ethiopia, 2016/2017. *Bmc Pediatrics* 2019; **19**(1).
19. Alexander DA, Northcross A, Karrison T, et al. Pregnancy outcomes and ethanol cook stove intervention: A randomized-controlled trial in Ibadan, Nigeria. *Environ Int* 2018; **111**((Adu, Ibigbami, Olamijulo, Adepoju) Healthy Life for All Foundation, Ibadan, Nigeria): 152–63.
20. Alim MA, Sarker MAB, Selim S, Karim MR, Yoshida Y, Hamajima N. Respiratory involvements among women exposed to the smoke of traditional biomass fuel and gas fuel in a district of Bangladesh. *Environmental Health and Preventive Medicine* 2014; **19**(2): 126–34.
21. Alvis-Guzman N, De la Hoz-Restrepo F, Montes-Farah J, Paternina-Caicedo A. Effect of biomass smoke on chronic obstructive pulmonary disease in rural localities of Colombia. *Rev Salud Publica (Bogota)* 2013; **15**(4): 589–600.
22. Amegah AK, Jaakkola JJK, Quansah R, Norgbe GK, Dzodzomenyo M. Cooking fuel choices and garbage burning practices as determinants of birth weight: a cross-sectional study in Accra, Ghana. *Environmental Health* 2012; **11**(78).
23. Anteneh ZA, Hassen HY. Determinants of Acute Respiratory Infection Among Children in Ethiopia: A Multilevel Analysis from Ethiopian Demographic and Health Survey. *International Journal of General Medicine* 2020; **13**: 17-26.

24. Arinola GO, Dutta A, Oluwole O, Olopade CO. Household Air Pollution, Levels of Micronutrients and Heavy Metals in Cord and Maternal Blood, and Pregnancy Outcomes. *International journal of environmental research and public health* 2018; **15**(12).
25. Arlington L, Patel AB, Simmons E, et al. Duration of solid fuel cookstove use is associated with increased risk of acute lower respiratory infection among children under six months in rural central India. *PloS one* 2019; **14**(10): e0224374.
26. Aung WW, Wai KT, Thu HM, et al. Aetiological agents, modifiable risk factors and gamma interferon status of children with acute respiratory infections attending general practitioner's clinics. *Myanmar Health Sciences Research Journal* 2013; **25**(3): 189–95.
27. Awasthi S, Glick HA, Fletcher RH, Ahmed N. Ambient air pollution & respiratory symptoms complex in preschool children. *Indian J Med Res* 1996; **104**(gjf, 0374701): 257–62.
28. Azad SMY, Bahauddin KM, Uddin MH, Shamima P. Indoor air pollution and prevalence of acute respiratory infection among children in rural area of Bangladesh. *Journal of Biology, Agriculture and Healthcare* 2014; **4**(2): 60–71.
29. Azizi BH, Henry RL. The effects of indoor environmental factors on respiratory illness in primary school children in Kuala Lumpur. *Int J Epidemiol* 1991; **20**(1): 144–50.
30. Azizi BH, Zulkifli HI, Kasim S. Indoor air pollution and asthma in hospitalized children in a tropical environment. *J Asthma* 1995; **32**(6): 413–8.
31. Baker RJ, Hertz-Picciotto I, Dostal M, et al. Coal home heating and environmental tobacco smoke in relation to lower respiratory illness in Czech children, from birth to 3 years of age. *Environ Health Perspect* 2006; **114**(7): 1126–32.
32. Balakrishnan K, Ghosh S, Thangavel G, et al. Exposures to fine particulate matter (PM2.5) and birthweight in a rural-urban, mother-child cohort in Tamil Nadu, India. *Environ Res* 2018; **161**((Balakrishnan, Ghosh, Thangavel, Sambandam, Mukhopadhyay, Puttaswamy, Sadasivam, Ramaswamy, Johnson, Kuppuswamy, Natesan, Maheshwari, Natarajan, Rajendran, Ramasami, Madhav, Manivannan, Nargunanadan, Natarajan, Saidam, Chakraborty, Balakrishnan, Thanaseka): 524–31.
33. Barone-Adesi F, Chapman RS, Silverman DT, et al. Risk of lung cancer associated with domestic use of coal in Xuanwei, China: retrospective cohort study. *British Medical Journal* 2012; **345**(e5414).
34. Barria PRM, Calvo GM. Factors associated to morbidity consultations due respiratory infection within first three months of life. *Revista Chilena de Pediatria* 2008; **79**(3): 281–9.
35. Barry AC, Mannino DM, Hopenhayn C, Bush H. Exposure to indoor biomass fuel pollutants and asthma prevalence in Southeastern Kentucky: results from the Burden of Lung Disease (BOLD) study. *J Asthma* 2010; **47**(7): 735–41.
36. Bassani DG, Jha P, Dhingra N, Kumar R. Child mortality from solid-fuel use in India: a nationally-representative case-control study. *BMC Public Health* 2010; **10**(100968562): 491.
37. Bassig BA, Dean Hosgood H, Shu XO, et al. Ischaemic heart disease and stroke mortality by specific coal type among non-smoking women with substantial indoor air pollution exposure in China. *International journal of epidemiology* 2019; ((Xiang) State Key Laboratory of Oncogene and Related Genes & Department of Epidemiology, Shanghai Cancer Institute, Renji Hospital, Shanghai Jiao Tong University School of Medicine, Shanghai, China).
38. Bates MN, Chandyo RK, Valentiner-Branth P, et al. Acute lower respiratory infection in childhood and household fuel use in Bhaktapur, Nepal. *Environ Health Perspect* 2013; **121**(5): 637–42.
39. Bates MN, Pokhrel AK, Chandyo RK, et al. Kitchen PM2.5 concentrations and child acute lower respiratory infection in Bhaktapur, Nepal: The importance of fuel type. *Environmental Research* 2018; **161**: 546-53.
40. Bates MN, Pope K, Sijali TR, et al. Household fuel use and pulmonary tuberculosis in western Nepal: A case-control study. *Environmental Research* 2019; **168**: 193-205.
41. Bautista LE, Correa A, Baumgartner J, Breysse P, Matanoski GM. Indoor charcoal smoke and acute respiratory infections in young children in the Dominican Republic. *Am J Epidemiol* 2009; **169**(5): 572–80.
42. Behera D, Balamugesh T. Indoor air pollution as a risk factor for lung cancer in women. *The Journal of the Association of Physicians of India* 2005; **53**(hg7, 7505585): 190–2.
43. Behera D, Aggarwal G. Domestic cooking fuel exposure and tuberculosis in Indian women. *Indian Journal of Chest Diseases and Allied Sciences* 2010; **52**(3): 139–43.
44. Behrens T, Maziak W, Weiland SK, Rzehak P, Siebert E, Keil U. Symptoms of asthma and the home environment. The ISAAC I and III cross-sectional surveys in Munster, Germany. *Int Arch Allergy Immunol* 2005; **137**(1): 53–61.
45. Belanger K, Beckett W, Triche E, et al. Symptoms of wheeze and persistent cough in the first year of life: Associations with indoor allergens, air contaminants, and maternal history of asthma. *American Journal of Epidemiology* 2003; **158**(3): 195–202.
46. Belanger K, Gent JF, Triche EW, Bracken MB, Leaderer BP. Association of indoor nitrogen dioxide exposure with respiratory symptoms in children with asthma. *Am J Respir Crit Care Med* 2006; **173**(3): 297–303.
47. Belanger K, Holford TR, Gent JF, Hill ME, Kezik JM, Leaderer BP. Household levels of nitrogen dioxide and pediatric asthma severity. *Epidemiology (Cambridge, Mass)* 2013; **24**(2): 320–30.

48. Bhat YR, Manjunath N, Sanjay D, Dhanya Y. Association of indoor air pollution with acute lower respiratory tract infections in children under 5 years of age. *Paediatrics and International Child Health* 2012; **32**(3): 132–5.
49. Bhat RY, Manjunath N. Correlates of acute lower respiratory tract infections in children under 5 years of age in India. *Int J Tuberc Lung Dis* 2013; **17**(3): 418–22.
50. Owusu Boadi K, Kuitunen M. Factors affecting the choice of cooking fuel, cooking place and respiratory health in the Accra metropolitan area, Ghana. *Journal of biosocial science* 2006; **38**(3): 403–12.
51. Bothwell JE, Stewart MC, Shields MD, McManus L, Crawford VLS, Burns G. Home heating and respiratory symptoms among children in Belfast, northern Ireland. *Archives of Environmental Health* 2003; **58**(9): 549–53.
52. Boy E, Bruce N, Delgado H. Birth weight and exposure to kitchen wood smoke during pregnancy in rural Guatemala. *Environ Health Perspect* 2002; **110**(1): 109–14.
53. Broor S, Pandey RM, Ghosh M, et al. Risk factors for severe acute lower respiratory tract infection in under-five children. *Indian Pediatr* 2001; **38**(12): 1361–9.
54. Bruce N, Neufeld L, Boy E, West C. Indoor biofuel air pollution and respiratory health: the role of confounding factors among women in highland Guatemala. *International Journal of Epidemiology* 1998; **27**(3): 454–8.
55. Buchner H, Rehfuess EA. Cooking and season as risk factors for acute lower respiratory infections in African children: a cross-sectional multi-country analysis. *PLoS One* 2015; **10**(6): e0128933.
56. Budge PJ, Griffin MR, Edwards KM, et al. Impact of home environment interventions on the risk of influenza-associated ARI in Andean children: observations from a prospective household-based cohort study. *PLoS One* 2014; **9**(3): e91247.
57. Budhathoki SS, Tinkari BS, Bhandari A, et al. The Association of Childhood Pneumonia with Household Air Pollution in Nepal: Evidence from Nepal Demographic Health Surveys. *Maternal and Child Health Journal* 2020; **24**(SUPPL 1).
58. Bueso A, Figueroa M, Cousin L, et al. Poverty-associated risk factors for wheezing in the first year of life in Honduras and El Salvador. *Allergol Immunopathol (Madr)* 2010; **38**(4): 203–12.
59. Bulkow LR, Singleton RJ, DeByle C, et al. Risk factors for hospitalization with lower respiratory tract infections in children in rural Alaska. *Pediatrics* 2012; **129**(5): e1220–7.
60. Burr ML, Anderson HR, Austin JB, et al. Respiratory symptoms and home environment in children: a national survey. *Thorax* 1999; **54**(1): 27–32.
61. Buthelezi SA, Kapwata T, Wernecke B, Webster C, Mathee A, Wright CY. Household Fuel Use for Heating and Cooking and Respiratory Health in a Low-Income, South African Coastal Community. *International journal of environmental research and public health* 2019; **16**(4).
62. Butland BK, Strachan DP, Anderson HR. The home environment and asthma symptoms in childhood: two population based case-control studies 13 years apart. *Thorax* 1997; **52**(7): 618–24.
63. Cardoso AM, Coimbra CE, Jr., Werneck GL. Risk factors for hospital admission due to acute lower respiratory tract infection in Guarani indigenous children in southern Brazil: a population-based case-control study. *Trop Med Int Health* 2013; **18**(5): 596–607.
64. Cardoso AM, Horta BL, Santos RV, Escobar AL, Welch JR, Coimbra CE, Jr. Prevalence of pneumonia and associated factors among indigenous children in Brazil: results from the First National Survey of Indigenous People's Health and Nutrition. *Int Health* 2015; **7**(6): 412–9.
65. Carlsten C, Brauer M, Dimich-Ward H, Dybuncio A, Becker AB, Chan-Yeung M. Combined exposure to dog and indoor pollution: incident asthma in a high-risk birth cohort. *Eur Respir J* 2011; **37**(2): 324–30.
66. Casas L, Tischer C, Tiesler C, et al. Association of gas cooking with children's respiratory health: results from GINIplus and LISAplus birth cohort studies. *Indoor Air* 2012; **22**(6): 476–82.
67. Cerqueiro MC, Murtagh P, Halac A, Avila M, Weissenbacher M. Epidemiologic risk factors for children with acute lower respiratory tract infection in Buenos Aires, Argentina: a matched case-control study. *Rev Infect Dis* 1990; **12 Suppl 8**: S1021–8.
68. Cetinkaya F, Gulmez I, Aydin T, Ozturk Y, Ozesmi M, Demir R. Prevalence of chronic bronchitis and associated risk factors in a rural area of Kayseri, Central Anatolia, Turkey. *Monaldi Arch Chest Dis* 2000; **55**(3): 189–93.
69. Chan KH, Kurmi OP, Bennett DA, et al. Solid Fuel Use and Risks of Respiratory Diseases A Cohort Study of 280,000 Chinese Never-Smokers. *American Journal of Respiratory and Critical Care Medicine* 2019; **199**(3): 352-61.
70. Chen CJ, Wu HY, Chuang YC, et al. Epidemiologic characteristics and multiple risk factors of lung cancer in Taiwan. *Anticancer Res* 1990; **10**(4): 971–6.
71. Chen HC, Wu CF, Chong IW, Wu MT. Exposure to cooking oil fumes and chronic bronchitis in nonsmoking women aged 40 years and over: a health-care based study. *BMC Public Health* 2018; **18**(1): 246.
72. Choi JY, Baumgartner J, Harnden S, et al. Increased risk of respiratory illness associated with kerosene fuel use among women and children in urban Bangalore, India. *Occupational and environmental medicine* 2015; **72**(2): 114–22.

73. Clark ML, Peel JL, Burch JB, et al. Impact of improved cookstoves on indoor air pollution and adverse health effects among Honduran women. *Int J Environ Health Res* 2009; **19**(5): 357–68.
74. Coggon D, Barker DJ, Inskip H, Wield G. Housing in early life and later mortality. *J Epidemiol Community Health* 1993; **47**(5): 345–8.
75. Coker ES, Smit E, Harding AK, Molitor J, Kile ML. A cross sectional analysis of behaviors related to operating gas stoves and pneumonia in U.S. children under the age of 5. *BMC Public Health* 2015; **15**(77): 77.
76. Collings DA, Sithole SD, Martin KS. Indoor woodsmoke pollution causing lower respiratory disease in children. *Trop Doct* 1990; **20**(4): 151–5.
77. Comstock GW, Meyer MB, Helsing KJ, Tockman MS. Respiratory effects on household exposures to tobacco smoke and gas cooking. *Am Rev Respir Dis* 1981; **124**(2): 143–8.
78. Crampin AC, Glynn JR, Floyd S, et al. Tuberculosis and gender: exploring the patterns in a case control study in Malawi. *International Journal of Tuberculosis and Lung Disease* 2004; **8**(2): 194–203.
79. da Silva LFF, Saldiva SRDM, Saldiva PHN, Dolhnikoff M. Impaired lung function in individuals chronically exposed to biomass combustion. *Environmental Research* 2012; **112**(Department of Pathology, University of Sao Paulo School of Medicine, Sao Paulo, Brazil.): 111–7.
80. Dagoye D, Bekele Z, Woldemichael K, et al. Domestic risk factors for wheeze in urban and rural Ethiopian children. *QJM* 2004; **97**(8): 489–98.
81. Dai XD, Lin CY, Sun XW, Shi YB, Lin YJ. The etiology of lung cancer in nonsmoking females in Harbin, China. *Lung Cancer* 1996; **14 Suppl 1**(SUPPL. 1): S85–91.
82. Daigler GE, Markello SJ, Cummings KM. The effect of indoor air pollutants on otitis media and asthma in children. *Laryngoscope* 1991; **101**(3): 293–6.
83. Das I, Jagger P, Yeatts K. Biomass Cooking Fuels and Health Outcomes for Women in Malawi. *Ecohealth* 2017; **14**(1): 7–19.
84. Montes de Oca M, Zabert G, Moreno D, Laucho-Contreras ME, Lopez Varela MV, Surmont F. Smoke, Biomass Exposure, and COPD Risk in the Primary Care Setting: The PUMA Study. *Respir Care* 2017; **62**(8): 1058–66.
85. Dekker C, Dales R, Bartlett S, Brunekreef B, Zwanenburg H. Childhood asthma and the indoor environment. *Chest* 1991; **100**(4): 922–6.
86. Dennis RJ, Maldonado D, Norman S, Baena E, Martinez G. Woodsmoke exposure and risk for obstructive airways disease among women. *Chest* 1996; **109**(1): 115–9.
87. Desalu OO, Adekoya AO, Ampitan BA. Increased risk of respiratory symptoms and chronic bronchitis in women using biomass fuels in Nigeria. *Jornal Brasileiro De Pneumologia* 2010; **36**(4): 441–6.
88. Devien L, Giovannelli J, Cuny D, et al. Sources of household air pollution: The association with lung function and respiratory symptoms in middle-aged adult. *Environ Res* 2018; **164**((Matran, Hulo, Edme) Pulmonary Function Testing Department, CHU Lille, Lille F-59000, France): 140–8.
89. Dhanaraj B, Papanna MK, Adinarayanan S, et al. Prevalence and risk factors for adult pulmonary tuberculosis in a metropolitan city of South India. *PLoS One* 2015; **10**(4): e0124260.
90. Dharmage SC, Rajapaksa LC, Fernando DN. Risk factors of acute lower respiratory tract infections in children under five years of age. *Southeast Asian J Trop Med Public Health* 1996; **27**(1): 107–10.
91. Diette GB, Hansel NN, Buckley TJ, et al. Home indoor pollutant exposures among inner-city children with and without asthma. *Environ Health Perspect* 2007; **115**(11): 1665–9.
92. Ding W, Wang X, Zhu F. Indoor environment and childhood asthma in Ningxia: a case-control study. *Journal of Environment and Health* 2009; **26**(9): 804–5.
93. Ding YP, Yao HX, Tang XL, et al. An epidemiology study of bronchial asthma in the Li ethnic group in. China. *Asian Pacific Journal of Tropical Medicine* 2012; **5**(2): 157–61.
94. Ding Y, Xu J, Yao J, et al. The analyses of risk factors for COPD in the Li ethnic group in Hainan, People's Republic of China. *Int J Chron Obstruct Pulmon Dis* 2015; **10**(101273481): 2593–600.
95. Dockery D, Spengler J, Neas L, et al. An epidemiologic study of respiratory health status and indicators of indoor air pollution from combustion sources; 1989.
96. Dodge R. The effects of indoor pollution on Arizona children. *Arch Environ Health* 1982; **37**(3): 151–5.
97. Dohoo C, Guernsey JR, Critchley K, VanLeeuwen J. Pilot study on the impact of biogas as a fuel source on respiratory health of women on rural Kenyan smallholder dairy farms. *J Environ Public Health* 2012; **2012**(101516361): 636298.
98. Dong GH, Ma YN, Ding HL, et al. Housing characteristics, home environmental factors and respiratory health in 3945 pre-school children in China. *Int J Environ Health Res* 2008; **18**(4): 267–82.
99. Dong GH, Ma YN, Ding HL, et al. Effects of housing characteristics and home environmental factors on respiratory symptoms of 10,784 elementary school children from northeast china. *Respiration* 2008; **76**(1): 82–91.
100. Dong GH, Qian ZM, Wang J, et al. Residential characteristics and household risk factors and respiratory diseases in Chinese women: the Seven Northeast Cities (SNEC) study. *The Science of the total environment* 2013; **463-464**(uj0, 0330500): 389–94.
101. Dossing M, Khan J, al-Rabiah F. Risk factors for chronic obstructive lung disease in Saudi Arabia. *Respir Med* 1994; **88**(7): 519–22.

102. Dow L, Phelps L, Fowler L, Waters K, Coggon D, Holgate ST. Respiratory symptoms in older people and use of domestic gas appliances. *Thorax* 1999; **54**(12): 1104–6.
103. Du YX, Cha Q, Chen XW, et al. An epidemiological study of risk factors for lung cancer in Guangzhou, China. *Lung Cancer* 1996; **14**(1}): S9–S37.
104. Dutt D, Srinivasa DK, Rotti SB, Sahai A, Konar D. Effect of indoor air pollution on the respiratory system of women using different fuels for cooking in an urban slum of Pondicherry. *National Medical Journal of India* 1996; **9**(3): 113–7.
105. Dutta A, Ray MR. Hypertension and respiratory health in biomass smoke-exposed premenopausal Indian women. *Air Quality, Atmosphere & Health* 2013; **7**(2): 229–38.
106. Ehrlich RI, White N, Norman R, et al. Predictors of chronic bronchitis in South African adults. *Int J Tuberc Lung Dis* 2004; **8**(3): 369–76.
107. Eisner MD, Yelin EH, Katz PP, Earnest G, Blanc PD. Exposure to indoor combustion and adult asthma outcomes: environmental tobacco smoke, gas stoves, and woodsmoke. *Thorax* 2002; **57**(11): 973–8.
108. Eisner MD, Blanc PD. Gas stove use and respiratory health among adults with asthma in NHANES III. *Occupational and environmental medicine* 2003; **60**(10): 759–64.
109. Ekici A, Ekici M, Kurtipek E, et al. Obstructive airway diseases in women exposed to biomass smoke. *Environ Res* 2005; **99**(1): 93–8.
110. Elf JL, Eke O, Rakgokong M, et al. Indoor air pollution from secondhand tobacco smoke, solid fuels, and kerosene in homes with active tuberculosis disease in South Africa. *BMC Res Notes* 2017; **10**(1): 591.
111. Elf JL, Kinikar A, Khadse S, et al. The association of household fine particulate matter and kerosene with tuberculosis in women and children in Pune, India. *Occupational and environmental medicine* 2019; **76**(1): 40-7.
112. Emenius G, Pershagen G, Berglind N, et al. NO2, as a marker of air pollution, and recurrent wheezing in children: a nested case-control study within the BAMSE birth cohort. *Occupational and environmental medicine* 2003; **60**(11): 876–81.
113. Emuron D, Siddharthan T, Morgan B, et al. Lack of an Association Between Household Air Pollution Exposure and Previous Pulmonary Tuberculosis. *Lung* 2019; **197**(6): 793-801.
114. Epstein MB, Bates MN, Arora NK, Balakrishnan K, Jack DW, Smith KR. Household fuels, low birth weight, and neonatal death in India: the separate impacts of biomass, kerosene, and coal. *Int J Hyg Environ Health* 2013; **216**(5): 523–32.
115. Esplugues A, Ballester F, Estarlich M, et al. Outdoor, but not indoor, nitrogen dioxide exposure is associated with persistent cough during the first year of life. *The Science of the total environment* 2011; **409**(22): 4667–73.
116. Esplugues A, Estarlich M, Sunyer J, et al. Prenatal exposure to cooking gas and respiratory health in infants is modified by tobacco smoke exposure and diet in the INMA birth cohort study. *Environmental Health* 2013; **12**(101147645): 100.
117. Etiler N, Velipasaoglu S, Aktekin M. Incidence of acute respiratory infections and the relationship with some factors in infancy in Antalya, Turkey. *Pediatrics International* 2002; **44**(1): 64–9.
118. Ezeh OK, Agho KE, Dibley MJ, Hall JJ, Page AN. The effect of solid fuel use on childhood mortality in Nigeria: evidence from the 2013 cross-sectional household survey. *Environ Health* 2014; **13**(Journal Article): 113.
119. Fagbule D, Ekanem EE. Some Environmental Risk-Factors for Childhood Asthma - a Case-Control Study. *Annals of Tropical Paediatrics* 1994; **14**(1): 15–9.
120. Faizan MA, Thakur R. Association Between Solid Cooking Fuels and Respiratory Disease Across Socio-Demographic Groups in India. *Journal of Health and Pollution* 2019; **9**(23).
121. Fakunle GA, Ana GR, Ayede AI. Environmental risk factors for acute respiratory infections in hospitalized children under 5 years of age in Ibadan, Nigeria. *Paediatr Int Child Health* 2014; **34**(2): 120–4.
122. Farrow A, Greenwood R, Preece S, Golding J. Nitrogen dioxide, the oxides of nitrogen, and infants' health symptoms. ALSPAC Study Team. Avon Longitudinal Study of Pregnancy and Childhood. *Arch Environ Health* 1997; **52**(3): 189–94.
123. Fatmi Z, Coggon D, Kazi A, Naeem I, Kadir MM, Sathiakumar N. Solid fuel use is a major risk factor for acute coronary syndromes among rural women: a matched case control study. *Public Health* 2014; **128**(1): 77–82.
124. Fatmi Z, Ntani G, Coggon D. Coronary heart disease, hypertension and use of biomass fuel among women: comparative cross-sectional study. *BMJ Open* 2019; **9**(8).
125. Fedortsiv O, Brozek GM, Luchyshyn N, et al. Prevalence of childhood asthma, rhinitis, and eczema in the Ternopil region of Ukraine--results of BUPAS study. *Adv Med Sci* 2012; **57**(2): 282–9.
126. Xu F, Yin X, Shen H, Xu Y, Ware RS, Owen N. Better understanding the influence of cigarette smoking and indoor air pollution on chronic obstructive pulmonary disease: a case-control study in Mainland China. *Respirology* 2007; **12**(6): 891–7.
127. Firdaus G, Ahmad A. Indoor air pollution and self-reported diseases - a case study of NCT of Delhi. *Indoor Air* 2011; **21**(5): 410–6.

128. Fischer PH, Kriz B, Martuzzi M, et al. Risk factors indoors and prevalences of childhood respiratory health in four countries in Western and Central Europe. *Indoor Air-International Journal of Indoor Air Quality and Climate* 1998; **8**(4): 244–54.
129. Foote EM, Gieraltowski L, Ayers T, et al. Impact of locally-produced, ceramic cookstoves on respiratory disease in children in rural western Kenya. *Am J Trop Med Hyg* 2013; **88**(1): 132–7.
130. Franklin PJ, Loveday J, Cook A. Unflued gas heaters and respiratory symptoms in older people with asthma. *Thorax* 2012; **67**(4): 315–20.
131. Galeone C, Pelucchi C, La Vecchia C, Negri E, Bosetti C, Hu J. Indoor air pollution from solid fuel use, chronic lung diseases and lung cancer in Harbin, Northeast China. *Eur J Cancer Prev* 2008; **17**(5): 473–8.
132. Gao YT, Blot WJ, Zheng W, et al. Lung cancer among Chinese women. *International journal of cancer* 1987; **40**(5): 604–9.
133. Cecilia Garcia-Sancho M, Garcia-Garcia L, Baez-Saldana R, et al. Indoor pollution as an occupational risk factor for tuberculosis among women: a population-based, gender oriented, case-control study in Southern Mexico. *REVISTA DE INVESTIGACION CLINICA-CLINICAL AND TRANSLATIONAL INVESTIGATION}* 2009; **61}**(5}): 392–8}.
134. Garrett MH, Hooper MA, Hooper BM, Abramson MJ. Respiratory symptoms in children and indoor exposure to nitrogen dioxide and gas stoves. *Am J Respir Crit Care Med* 1998; **158**(3): 891–5.
135. Gaviola C, Miele CH, Wise RA, et al. Urbanisation but not biomass fuel smoke exposure is associated with asthma prevalence in four resource-limited settings. *Thorax* 2016; **71**(2): 154–60.
136. Ghimire PR, Agho KE, Renzaho AMN, Nisha MK, Dibley M, Raynes-Greenow C. Factors associated with perinatal mortality in Nepal: evidence from Nepal demographic and health survey 2001-2016. *BMC Pregnancy and Childbirth* 2019; **19**.
137. Ghosh S. Indoor air pollution in India and a baby's size at birth: is there a link? *World Health & Population* 2006; **8**(4): 34–50.
138. Gillespie-Bennett J, Pierse N, Wickens K, et al. The respiratory health effects of nitrogen dioxide in children with asthma. *Eur Respir J* 2011; **38**(2): 303–9.
139. Gninafon M, Ade G, Ait-Khaled N, Enarson DA, Chiang CY. Exposure to combustion of solid fuel and tuberculosis: a matched case-control study. *Eur Respir J* 2011; **38**(1): 132–8.
140. Golshan M, Faghihi M, Marandi MM. Indoor women jobs and pulmonary risks in rural areas of Isfahan, Iran, 2000. *Respir Med* 2002; **96**(6): 382–8.
141. Gonzalez-Garcia M, Caballero A, Jaramillo C, Maldonado D, Torres-Duque CA. Prevalence, risk factors and underdiagnosis of asthma and wheezing in adults 40 years and older: A population-based study. *J Asthma* 2015; **52**(8): 823–30.
142. Guggisberg M, Hessel PA, Michaelchuk D, Ahmed I. Respiratory symptoms and exposure to wood smoke in an isolated northern community. *Can J Public Health* 2003; **94**(5): 372–6.
143. Gupta BN, Mathur N. A study of household environmental risk factors pertaining to respiratory diseases; 1997.
144. Gupta D, Boffetta P, Gaborieau V, Jindal SK. Risk factors of lung cancer in Chandigarh, India. *Indian Journal of Medical Research* 2001; **113**(gjf, 0374701): 142–50.
145. Gurley ES, Homaira N, Salje H, et al. Indoor exposure to particulate matter and the incidence of acute lower respiratory infections among children: a birth cohort study in urban Bangladesh. *Indoor Air* 2013; **23**(5): 379–86.
146. Hansel NN, Breysse PN, McCormack MC, et al. A longitudinal study of indoor nitrogen dioxide levels and respiratory symptoms in inner-city children with asthma. *Environ Health Perspect* 2008; **116**(10): 1428–32.
147. Hansel NN, McCormack MC, Belli AJ, et al. In-home air pollution is linked to respiratory morbidity in former smokers with chronic obstructive pulmonary disease. *Am J Respir Crit Care Med* 2013; **187**(10): 1085–90.
148. Haque MA, Barman N, Islam MT, et al. Biomass Fuel Smoke and Tuberculosis: A Case-Control Study. *Mymensingh medical journal : MMJ* 2016; **25**(1): 31–8.
149. Harerimana JM, Nyirazinyoye L, Thomson DR, Ntaganira J. Social, economic and environmental risk factors for acute lower respiratory infections among children under five years of age in Rwanda. *Archives of Public Health* 2016; **74**(19).
150. Hasan MM, Richardson A. How sustainable household environment and knowledge of healthy practices relate to childhood morbidity in South Asia: analysis of survey data from Bangladesh, Nepal and Pakistan. *BMJ Open* 2017; **7**(6): e015019.
151. Hasan M, Tasfina S, Haque SMR, et al. Association of biomass fuel smoke with respiratory symptoms among children under 5 years of age in urban areas: results from Bangladesh Urban Health Survey, 2013. *Environmental health and preventive medicine* 2019; **24**(1): 65.
152. Hazra A, Datta S, Guha M. Effect of Household Environment on Women't Health in North-East India. *Population and Environment Linkages*; (15): 264–74.
153. Helsing KJ, Comstock GW, Meyer MB, Tockman ML. Respiratory effects of household exposures to tobacco smoke and gas cooking on nonsmokers. *Environment International* 1982; **8**(1): 365–70.

154. Hernandez-Garduno E, Brauer M, Perez-Neria J, Vedal S. Wood smoke exposure and lung adenocarcinoma in non-smoking Mexican women. *Int J Tuberc Lung Dis* 2004; **8**(3): 377–83.
155. Hersoug LG, Husemoen LL, Sigsgaard T, Madsen F, Linneberg A. Indoor exposure to environmental cigarette smoke, but not other inhaled particulates associates with respiratory symptoms and diminished lung function in adults. *Respirology* 2010; **15**(6): 993–1000.
156. Hessel PA, Klaver J, Michaelchuk D, McGhan S, Carson MM, Melvin D. The epidemiology of childhood asthma in Red Deer and Medicine Hat, Alberta. *Can Respir J* 2001; **8**(3): 139–46.
157. Hoek G, Brunekreef B, Meijer R, Scholten A, Boleij J. Indoor nitrogen dioxide pollution and respiratory symptoms of schoolchildren. *Int Arch Occup Environ Health* 1984; **55**(1): 79–86.
158. Holscher B, Heinrich J, Jacob B, Ritz B, Wichmann HE. Gas cooking, respiratory health and white blood cell counts in children. *Int J Hyg Environ Health* 2000; **203**(1): 29–37.
159. Honicky RE, Osborne JS, Akpom CA. Symptoms of Respiratory Illness in Young-Children and the Use of Wood-Burning Stoves for Indoor Heating. *Pediatrics* 1985; **75**(3): 587–93.
160. Hosein HR, Corey P, Robertson JM. The effect of domestic factors on respiratory symptoms and FEV1. *Int J Epidemiol* 1989; **18**(2): 390–6.
161. Hosgood HD, 3rd, Chapman R, Shen M, et al. Portable stove use is associated with lower lung cancer mortality risk in lifetime smoky coal users. *Br J Cancer* 2008; **99**(11): 1934–9.
162. Hosgood HD, 3rd, Boffetta P, Greenland S, et al. In-home coal and wood use and lung cancer risk: a pooled analysis of the International Lung Cancer Consortium. *Environ Health Perspect* 2010; **118**(12): 1743–7.
163. Howden-Chapman P, Pierse N, Nicholls S, et al. Effects of improved home heating on asthma in community dwelling children: randomised controlled trial. *BMJ* 2008; **337**(8900488, bmj, 101090866): a1411.
164. Huang C, Zhang X, Qiao Z, et al. A case-control study of dietary factors in patients with lung cancer. *Biomed Environ Sci* 1992; **5**(3): 257–65.
165. Hystad P, Duong M, Brauer M, et al. Health Effects of Household Solid Fuel Use: Findings from 11 Countries within the Prospective Urban and Rural Epidemiology Study. *Environmental Health Perspectives* 2019; **127**(5).
166. Indahsari N, Noor NN, Arsyad DS, Acm. Relationship Of Exposure Of Air Pollution In House With Occurrence Of Acute Respiratory Infections In Children Under Five At Puskesmas Malimongan Baru in 2016; 2018.
167. Infante-Rivard C. Childhood asthma and indoor environmental risk factors. *American journal of epidemiology* 1993; **137**(8): 834–44.
168. Ingale LT, Dube KJ, Sarode DB, Attarde SB, Ingle ST. Monitoring and respiratory health assessment of the population exposed to cooking fuel emissions in a rural area of Jalgaon district, India. *Asia Pac J Public Health* 2013; **25**(6): 463–75.
169. Islam F, Sarma R, Debroy A, Kar S, Pal R. Profiling acute respiratory tract infections in children from assam, India. *J Glob Infect Dis* 2013; **5**(1): 8–14.
170. Jafta N, Jeena PM, Barregard L, Naidoo RN. Association of childhood pulmonary tuberculosis with exposure to indoor air pollution: a case control study. *BMC Public Health* 2019; **19**.
171. Jaganath D, Miranda JJ, Gilman RH, et al. Prevalence of chronic obstructive pulmonary disease and variation in risk factors across four geographically diverse resource-limited settings in Peru. *Respir Res* 2015; **16**: 40.
172. Jamali T, Fatmi Z, Shahid A, Khoso A, Kadir MM, Sathiakumar N. Evaluation of short-term health effects among rural women and reduction in household air pollution due to improved cooking stoves: quasi experimental study. *Air Quality Atmosphere and Health* 2017; **10**(7): 809–19.
173. Janjua NZ, Mahmood B, Dharma VK, Sathiakumar N, Khan MI. Use of biomass fuel and acute respiratory infections in rural Pakistan. *Public Health* 2012; **126**(10): 855–62.
174. Jarvis D, Chinn S, Luczynska C, Burney P. Association of respiratory symptoms and lung function in young adults with use of domestic gas appliances. *Lancet* 1996; **347**(8999): 426–31.
175. Jarvis D, Chinn S, Sterne J, Luczynska C, Burney P. The association of respiratory symptoms and lung function with the use of gas for cooking. European Community Respiratory Health Survey. *The European respiratory journal* 1998; **11**(3): 651–8.
176. Jedrychowski W, Tobiasz-Adamczyk B, Flak E, Mroz E, Gomola K. Effect of indoor air pollution caused by domestic cooking on respiratory problems of elderly women. *Environment International* 1990; **16**(1): 57–60.
177. Jedrychowski W, Maugeri U, Gomola K, Tobiasz-Adamczyk B, Bianchi II. Effects of Deomestic Gas Cooking and Passive Smoking on Chronic Respiratory Symptoms and Asthma in Elderly Women. *Int J Occup Environ Health* 1995; **1**(1): 16–20.
178. Jiang M, Qiu J, Zhou M, et al. Exposure to cooking fuels and birth weight in Lanzhou, China: a birth cohort study. *BMC Public Health* 2015; **15**(712): 712.
179. Jie Y, Li KB, Yin T, Jie X. Prevalence of Asthma and Asthma-Related Symptoms Among Adults Exposed to Indoor Environmental Risk Factors: a Comparison between Winter and Summer in Zunyi, China. *Polish Journal of Environmental Studies* 2016; **25**(2): 621–33.

180. Jindal SK, Aggarwal AN, Chaudhry K, et al. A multicentric study on epidemiology of chronic obstructive pulmonary disease and its relationship with tobacco smoking and environmental tobacco smoke exposure. *Indian J Chest Dis Allied Sci* 2006; **48**(1): 23–9.
181. Jindal SK, Aggarwal AN, Gupta D, et al. Indian study on epidemiology of asthma, respiratory symptoms and chronic bronchitis in adults (INSEARCH). *Int J Tuberc Lung Dis* 2012; **16**(9): 1270–7.
182. Johnson AW, Osinusi K, Aderele WI, Gbadero DA, Olaleye OD, Adeyemi-Doro FA. Etiologic agents and outcome determinants of community-acquired pneumonia in urban children: a hospital-based study. *J Natl Med Assoc* 2008; **100**(4): 370–85.
183. Johnson P, Balakrishnan K, Ramaswamy P, et al. Prevalence of chronic obstructive pulmonary disease in rural women of Tamilnadu: implications for refining disease burden assessments attributable to household biomass combustion. *Glob Health Action* 2011; **4**(Department of Physiology, Sri Ramachandra University, No. 1, Ramachandra Nagar, Porur Chennai, Tamilnadu 600 116, India.): 7226.
184. Jubulis J, Kinikar A, Ithape M, et al. Modifiable risk factors associated with tuberculosis disease in children in Pune, India. *International Journal of Tuberculosis and Lung Disease* 2014; **18**(2): 198–204.
185. Juntarawijit Y, Juntarawijit C. Cooking smoke exposure and respiratory symptoms among those responsible for household cooking: A study in Phitsanulok, Thailand. *Heliyon* 2019; **5**(5).
186. Kadam YR, Mimansa A, Chavan PV, Gore AD. Effect of prenatal exposure to kitchen fuel on birth weight. *Indian J Community Med* 2013; **38**(4): 212–6.
187. Kafando B, Savadogo PW, Millogo T, Sana A, Kouanda S, Sondo B. Indoor air pollution and acute respiratory infection prevalence in infants in Ouagadougou. *Sante Publique* 2018; **30**(4): 575-86.
188. Kaji DA, Belli AJ, McCormack MC, et al. Indoor pollutant exposure is associated with heightened respiratory symptoms in atopic compared to non-atopic individuals with COPD. *BMC Pulm Med* 2014; **14**(147).
189. Kan X, Chiang CY, Enarson DA, Chen W, Yang J, Chen G. Indoor solid fuel use and tuberculosis in China: a matched case-control study. *BMC Public Health* 2011; **11**(Journal Article): 498.
190. Kashima S, Yorifuji T, Tsuda T, Ibrahim J, Doi H. Effects of traffic-related outdoor air pollution on respiratory illness and mortality in children, taking into account indoor air pollution, in Indonesia. *J Occup Environ Med* 2010; **52**(3): 340–5.
191. Kasznia-Kocot J, Kowalska M, Gorny RL, Niesler A, Wypych-Slusarska A. Environmental risk factors for respiratory symptoms and childhood asthma. *Ann Agric Environ Med* 2010; **17**(2): 221–9.
192. Khalequzzaman M, Kamijima M, Sakai K, Chowdhury NA, Hamajima N, Nakajima T. Indoor air pollution and its impact on children under five years old in Bangladesh. *Indoor Air* 2007; **17**(4): 297–304.
193. Khan MN, CZ BN, Mofizul Islam M, Islam MR, Rahman MM. Household air pollution from cooking and risk of adverse health and birth outcomes in Bangladesh: a nationwide population-based study. *Environ Health* 2017; **16**(1): 57.
194. Khan MSB, Lohano HD. Household air pollution from cooking fuel and respiratory health risks for children in Pakistan. *Environmental Science and Pollution Research* 2018; **25**(25): 24778-86.
195. Kilabuko JH, Nakai S. Effects of cooking fuels on acute respiratory infections in children in Tanzania. *International journal of environmental research and public health* 2007; **4**(4): 283–8.
196. Kilpelainen M, Koskenvuo M, Helenius H, Terho E. Wood stove heating, asthma and allergies. *Respir Med* 2001; **95**(11): 911–6.
197. Kim C, Chapman RS, Hu W, et al. Smoky coal, tobacco smoking, and lung cancer risk in Xuanwei, China. *Lung Cancer* 2014; **84**(1): 31–5.
198. Kim AS, Cahill E, Cheng NT. Global Stroke Belt: Geographic Variation in Stroke Burden Worldwide. *Stroke* 2015; **46**(12): 3564–70.
199. Kim C, Seow WJ, Shu XO, et al. Cooking Coal Use and All-Cause and Cause-Specific Mortality in a Prospective Cohort Study of Women in Shanghai, China. *Environmental Health Perspectives* 2016; **124**(9): 1384–9.
200. Kiraz K, Kart L, Demir R, et al. Chronic pulmonary disease in rural women exposed to biomass fumes. *Clin Invest Med* 2003; **26**(5): 243–8.
201. Kleimola LB, Patel AB, Borkar JA, Hibberd PL. Consequences of household air pollution on child survival: evidence from demographic and health surveys in 47 countries. *Int J Occup Environ Health* 2015; **21**(4): 294–302.
202. Kleinerman RA, Wang Z, Wang L, et al. Lung cancer and indoor exposure to coal and biomass in rural China. *J Occup Environ Med* 2002; **44**(4): 338–44.
203. Ko YC, Lee CH, Chen MJ, et al. Risk factors for primary lung cancer among non-smoking women in Taiwan. *International Journal of Epidemiology* 1997; **26**(1): 24–31.
204. Kolappan C, Subramani R. Association between biomass fuel and pulmonary tuberculosis: a nested case-control study. *Thorax* 2009; **64**(8): 705–8.
205. Koo LC, Lee N, Ho JH. Do cooking fuels pose a risk for lung cancer? A case-control study of women in Hong Kong. *Ecol Dis* 1983; **2**(4): 255–65.
206. Kraai S, Verhagen LM, Valladares E, et al. High prevalence of asthma symptoms in Warao Amerindian children in Venezuela is significantly associated with open-fire cooking: a cross-sectional observational study. *Respir Res* 2013; **14**(Journal Article): 76.

207. Kristensen IA, Olsen J. Determinants of acute respiratory infections in Soweto - a population-based birth cohort. *Samj South African Medical Journal* 2006; **96**(7): 633–40.
208. Yu K, Qiu GK, Chan KH, et al. Association of Solid Fuel Use With Risk of Cardiovascular and All-Cause Mortality in Rural China. *Jama-Journal of the American Medical Association* 2018; **319**(13): 1351–61.
209. Kuhr J, Hendelkramer A, Karmaus W, et al. Air-Pollution and Asthma among School-Children. *Sozial-Und Praventivmedizin* 1991; **36**(2): 67–73.
210. Kumar S, Awasthi S, Jain A, Srivastava RC. Blood zinc levels in children hospitalized with severe pneumonia: a case control study. *Indian Pediatr* 2004; **41**(5): 486–91.
211. Kumar R, Nagar JK, Raj N, et al. Impact of Domestic Air Pollution from Cooking Fuel on Respiratory Allergies in Children in India. *Asian Pacific Journal of Allergy and Immunology* 2008; **26**(4): 213–22.
212. Kurmi OP, Semple S, Devereux GS, et al. The effect of exposure to biomass smoke on respiratory symptoms in adult rural and urban Nepalese populations. *Environ Health* 2014; **13**(Journal Article): 92.
213. Kurt E, Metintas S, Basyigit I, et al. Prevalence and risk factors of allergies in Turkey: Results of a multicentric cross-sectional study in children. *Pediatr Allergy Immunol* 2007; **18**(7): 566–74.
214. Kurt E, Metintas S, Basyigit I, et al. Prevalence and Risk Factors of Allergies in Turkey (PARFAIT): results of a multicentre cross-sectional study in adults. *Eur Respir J* 2009; **33**(4): 724–33.
215. Kurti SP, Kurti AN, Emerson SR, et al. Household Air Pollution Exposure and Influence of Lifestyle on Respiratory Health and Lung Function in Belizean Adults and Children: A Field Study. *International journal of environmental research and public health* 2016; **13**(7): 643.
216. Kwas H, Rahmouni N, Zendah I, Ghedira H. [Respiratory symptoms and obstructive ventilatory disorder in Tunisian woman exposed to biomass]. *Rev Pneumol Clin* 2017; **73**(3): 120–6.
217. Lakshmi PV, Virdi NK, Thakur JS, Smith KR, Bates MN, Kumar R. Biomass fuel and risk of tuberculosis: a case-control study from Northern India. *J Epidemiol Community Health* 2012; **66**(5): 457–61.
218. Lakshmi PV, Virdi NK, Sharma A, et al. Household air pollution and stillbirths in India: analysis of the DLHS-II National Survey. *Environ Res* 2013; **121**(School of Public Health, Post Graduate Institute of Medical Education and Research (PGIMER), Chandigarh - 160 012, India.): 17–22.
219. Lamichhane P, Sharma A, Mahal A. Impact of cleaner fuel use and improved stoves on acute respiratory infections: evidence from India. *Int Health* 2017; **9**(6): 349–66.
220. Lan Q, Chen W, Chen H, He XZ. Risk factors for lung cancer in non-smokers in Xuanwei County of China. *Biomed Environ Sci* 1993; **6**(2): 112–8.
221. Lan Q, Chapman RS, Schreinemachers DM, Tian L, He X. Household stove improvement and risk of lung cancer in Xuanwei, China. *J Natl Cancer Inst* 2002; **94**(11): 826–35.
222. Lan Q, He X, Shen M, et al. Variation in lung cancer risk by smoky coal subtype in Xuanwei, China. *International journal of cancer* 2008; **123**(9): 2164–9.
223. Laniado-Laborin R, Rendon A, Bauerle O. Chronic obstructive pulmonary disease case finding in Mexico in an at-risk population. *Int J Tuberc Lung Dis* 2011; **15**(6): 818–23.
224. Lanphear BP, Aligne CA, Auinger P, Weitzman M, Byrd RS. Residential exposures associated with asthma in US children. *Pediatrics* 2001; **107**(3): 505–11.
225. Lee CH, Ko YC, Cheng LS, et al. The heterogeneity in risk factors of lung cancer and the difference of histologic distribution between genders in Taiwan. *Cancer Causes Control* 2001; **12**(4): 289–300.
226. Lee MS, Hang JQ, Zhang FY, Dai HL, Su L, Christiani DC. In-home solid fuel use and cardiovascular disease: a cross-sectional analysis of the Shanghai Putuo study. *Environ Health* 2012; **11**(Journal Article): 18.
227. Levesqu B, Allaire S, Gauvin D, et al. Wood-burning appliances and indoor air quality. *The Science of the total environment* 2001; **281**(1-3): 47–62.
228. Lewis JJ, Hollingsworth JW, Chartier RT, et al. Biogas Stoves Reduce Firewood Use, Household Air Pollution, and Hospital Visits in Odisha, India. *Environ Sci Technol* 2017; **51**(1): 560–9.
229. Li SX, Xu J, Jiang ZG, Luo Y, Yang Y, Yu J. Correlation between indoor air pollution and adult respiratory health in Zunyi City in Southwest China: situation in two different seasons. *BMC Public Health* 2019; **19**.
230. Li J, He J, Tang R, et al. Molecular epidemiology study in Xuanwei: the relationship among coal type, genotype and lung cancer risk. *Chinese Journal of Lung Cancer* 2015; **18**(1): 16–22.
231. Li JC, Qin CX, Lv J, et al. Solid Fuel Use and Incident COPD in Chinese Adults: Findings from the China Kadoorie Biobank. *Environmental Health Perspectives* 2019; **127**(5).
232. Lin W, Gehring U, Oldenwening M, et al. Gas cooking, respiratory and allergic outcomes in the PIAMA birth cohort study. *Occupational and environmental medicine* 2013; **70**(3): 187–94.
233. Zhang L, Li J, Wang Y, Wu G, Wei F. Descriptive study of the environmental epidemiology of high lung cancer incidence rate in Qujing, Yunnan, China. *Chinese Journal of Lung Cancer* 2012; **15**(3): 159–63.
234. Lissowska J, Bardin-Mikolajczak A, Fletcher T, et al. Lung cancer and indoor pollution from heating and cooking with solid fuels: the IARC international multicentre case-control study in Eastern/Central Europe and the United Kingdom. *Am J Epidemiol* 2005; **162**(4): 326–33.
235. Liu Q, Sasco AJ, Riboli E, Hu MX. Indoor air pollution and lung cancer in Guangzhou, People's Republic of China. *Am J Epidemiol* 1993; **137**(2): 145–54.

236. Liu S, Zhou Y, Wang X, et al. Biomass fuels are the probable risk factor for chronic obstructive pulmonary disease in rural South China. *Thorax* 2007; **62**(10): 889–97.
237. Liu F, Zhao Y, Liu YQ, et al. Asthma and asthma related symptoms in 23,326 Chinese children in relation to indoor and outdoor environmental factors: the Seven Northeastern Cities (SNEC) Study. *The Science of the total environment* 2014; **497-498**(uj0, 0330500): 10–7.
238. Liu W, Huang C, Hu Y, Zou ZJ, Zhao ZH, Sundell J. Association of building characteristics, residential heating and ventilation with asthmatic symptoms of preschool children in Shanghai: A cross-sectional study. *Indoor and Built Environment* 2014; **23**(2): 270–83.
239. Liu J, Hou BD, Ma XW, Liao H. Solid fuel use for cooking and its health effects on the elderly in rural China. *Environmental Science and Pollution Research* 2018; **25**(4): 3669-80.
240. Liu W, Huang C, Cai J, Wang XY, Zou ZJ, Sun CJ. Household environmental exposures during gestation and birth outcomes: A cross-sectional study in Shanghai, China. *Science of the Total Environment* 2018; **615**: 1110-8.
241. Loeb M, Neupane B, Walter SD, et al. Environmental risk factors for community-acquired pneumonia hospitalization in older adults. *Journal of the American Geriatrics Society* 2009; **57**(6): 1036–40.
242. Luo RX, Wu B, Yi YN, Huang ZW, Lin RT. Indoor burning coal air pollution and lung cancer--a case-control study in Fuzhou, China. *Lung Cancer* 1996; **14 Suppl 1**(SUPPL. 1): S113–9.
243. Mahalanabis D, Gupta S, Paul D, Gupta A, Lahiri M, Khaled MA. Risk factors for pneumonia in infants and young children and the role of solid fuel for cooking: a case-control study. *Epidemiology and infection* 2002; **129**(1): 65–71.
244. Maier WC, Arrighi HM, Morray B, Llewellyn C, Redding GJ. Indoor risk factors for asthma and wheezing among Seattle school children. *Environmental Health Perspectives* 1997; **105**(2): 208–14.
245. Malinauskiene V, Leisyte P, Malinauskas R, Bagdonas G, Jankauskiene L, Malinauskaite I. Outdoor and Indoor Air Pollution and Myocardial Infarction among Women in Kaunas, Lithuania: a Case-Control Study. *Polish Journal of Environmental Studies* 2011; **20**(4): 969–75.
246. Manyeh AK, Kukula V, Odonkor G, et al. Socioeconomic and demographic determinants of birth weight in southern rural Ghana: evidence from Dodowa Health and Demographic Surveillance System. *BMC Pregnancy Childbirth* 2016; **16**(1): 160.
247. Mavalankar DV, Trivedi CR, Gray RH. Levels and risk factors for perinatal mortality in Ahmedabad, India. *Bull World Health Organ* 1991; **69**(4): 435–42.
248. Mavalankar DV, Gray RH, Trivedi CR. Risk factors for preterm and term low birthweight in Ahmedabad, India. *Int J Epidemiol* 1992; **21**(2): 263–72.
249. McConnell R, Berhane K, Gilliland F, et al. Indoor risk factors for asthma in a prospective study of adolescents. *Epidemiology (Cambridge, Mass)* 2002; **13**(3): 288–95.
250. McCormack MC, Breysse PN, Matsui EC, et al. In-home particle concentrations and childhood asthma morbidity. *Environ Health Perspect* 2009; **117**(2): 294–8.
251. Mejias SG, Ramphul K. Prevalence and Associated Risk Factors Bronchial Asthma in Children in Santo Domingo, Dominican Republic. *Cureus* 2018; **10**(2).
252. Melia RJW, Florey CDV, Altman DG, Swan AV. Association between Gas Cooking and Respiratory-Disease in Children. *British Medical Journal* 1977; **2**(6080): 149–52.
253. Melia RJ, Florey CV, Chinn S. The relation between respiratory illness in primary schoolchildren and the use of gas for cooking--I. Results from a national survey. *Int J Epidemiol* 1979; **8**(4): 333–8.
254. Melsom T, Brinch L, Hessen JO, et al. Asthma and indoor environment in Nepal. *Thorax* 2001; **56**(6): 477–81.
255. Menezes AM, Victora CG, Rigatto M. Prevalence and risk factors for chronic bronchitis in Pelotas, RS, Brazil: a population-based study. *Thorax* 1994; **49**(12): 1217–21.
256. Milanzi EB, Namacha NM. Maternal biomass smoke exposure and birth weight in Malawi: Analysis of data from the 2010 Malawi Demographic and Health Survey. *Malawi Med J* 2017; **29**(2): 160–5.
257. Mir AA, Imtiyaz A, Anjum F, Javeed I, Rohul J, Salathia A. Prevalence and risk factor analysis of acute respiratory tract infections in rural areas of Kashmir valley under 5 years of age. *International Journal of Medicine and Public Health* 2012; **2**(3): 47–52.
258. Mishra VK, Retherford RD, Smith KR. Biomass cooking fuels and prevalence of tuberculosis in India. *International journal of infectious diseases : IJID : official publication of the International Society for Infectious Diseases* 1999; **3**(3): 119–29.
259. Mishra V. Effect of indoor air pollution from biomass combustion on prevalence of asthma in the elderly. *Environ Health Perspect* 2003; **111**(1): 71–8.
260. Mishra V. Indoor air pollution from biomass combustion and acute respiratory illness in preschool age children in Zimbabwe. *International Journal of Epidemiology* 2003; **32**(5): 847–53.
261. Mishra V, Dai X, Smith KR, Mika L. Maternal exposure to biomass smoke and reduced birth weight in Zimbabwe. *Ann Epidemiol* 2004; **14**(10): 740–7.
262. Mishra V, Retherford RD, Smith KR. Cooking smoke and tobacco smoke as risk factors for stillbirth. *Int J Environ Health Res* 2005; **15**(6): 397–410.

263. Mishra V, Smith KR, Retherford RD. Effects of cooking smoke and environmental tobacco smoke on acute respiratory infections in young Indian children. *Population and Environment* 2005; **26**(5): 375–96.
264. Misra A, Longnecker MP, Dionisio KL, et al. Household fuel use and biomarkers of inflammation and respiratory illness among rural South African Women. *Environmental Research* 2018; **166**: 112-6.
265. Mitter SS, Vedanthan R, Islami F, et al. Household Fuel Use and Cardiovascular Disease Mortality: Golestan Cohort Study. *Circulation* 2016; **133**(24): 2360–9.
266. Mommers M, Jongmans-Liedekerken AW, Derkx R, et al. Indoor environment and respiratory symptoms in children living in the Dutch-German borderland. *Int J Hyg Environ Health* 2005; **208**(5): 373–81.
267. Moran SE, Strachan DP, Johnston IDA, Anderson HR. Effects of exposure to gas cooking in childhood and adulthood on respiratory symptoms, allergic sensitization and lung function in young British adults. *Clinical and Experimental Allergy* 1999; **29**(8): 1033–41.
268. Morris K, Morgenlander M, Coulehan JL, Gahagen S, Arena VC. Wood-burning stoves and lower respiratory tract infection in American Indian children. *Am J Dis Child* 1990; **144**(1): 105–8.
269. Mortimer K, Ndamala CB, Naunje AW, et al. A cleaner burning biomass-fuelled cookstove intervention to prevent pneumonia in children under 5 years old in rural Malawi (the Cooking and Pneumonia Study): a cluster randomised controlled trial. *Lancet* 2017; **389**(10065): 167–75.
270. Mu L, Liu L, Niu R, et al. Indoor air pollution and risk of lung cancer among Chinese female non-smokers. *Cancer Causes Control* 2013; **24**(3): 439–50.
271. Mukherjee S, Roychoudhury S, Siddique S, et al. Respiratory symptoms, lung function decrement and chronic obstructive pulmonary disease in pre-menopausal Indian women exposed to biomass smoke. *Inhal Toxicol* 2014; **26**(14): 866–72.
272. Mukherjee S, Siddique S, Chakraborty S, et al. Adverse reproductive health outcomes in pre-menopausal Indian women chronically exposed to biomass smoke. *Journal of Public Health-Heidelberg* 2015; **23**(6): 363–72.
273. Murray EL, Brondi L, Kleinbaum D, et al. Cooking fuel type, household ventilation, and the risk of acute lower respiratory illness in urban Bangladeshi children: a longitudinal study. *Indoor Air* 2012; **22**(2): 132–9.
274. Musafiri S, Masaisa F, Bavuma MC, Manzi OO, Kalisa UL, Rutayisire PC. Indoor air pollution from cooking with biomass fuels is a major cause of chronic bronchitis among women in a rural district of Rwanda. *African Journal of Respiratory Medicine* 2018; **14**(1): 16-9.
275. Mustapha BA, Blangiardo M, Briggs DJ, Hansell AL. Traffic air pollution and other risk factors for respiratory illness in schoolchildren in the niger-delta region of Nigeria. *Environ Health Perspect* 2011; **119**(10): 1478–82.
276. Mzileni O, Sitas F, Steyn K, Carrara H, Bekker P. Lung cancer, tobacco, and environmental factors in the African population of the Northern Province, South Africa. *Tob Control* 1999; **8**(4): 398–401.
277. Naz S, Page A, Agho KE. Household Air Pollution and Under-Five Mortality in Bangladesh (2004-2011). *International journal of environmental research and public health* 2015; **12**(10): 12847–62.
278. Naz S, Page A, Agho KE. Household air pollution and under-five mortality in India (1992-2006). *Environ Health* 2016; **15**(Journal Article): 54.
279. Naz S, Page A, Agho KE. Household air pollution from use of cooking fuel and under-five mortality: The role of breastfeeding status and kitchen location in Pakistan. *Plos One* 2017; **12**(3): e0173256.
280. Naz S, Page A, Agho KE. Potential Impacts of Modifiable Behavioral and Environmental Exposures on Reducing Burden of Under-five Mortality Associated with Household Air Pollution in Nepal. *Matern Child Health J* 2018; **22**(1): 59–70.
281. Neas LM, Dockery DW, Ware JH, Spengler JD, Speizer FE, Ferris BG, Jr. Association of indoor nitrogen dioxide with respiratory symptoms and pulmonary function in children. *Am J Epidemiol* 1991; **134**(2): 204–19.
282. Neas LM, Dockery DW, Ware JH, Spengler JD, Ferris BG, Speizer FE. Concentration of Indoor Particulate Matter as a Determinant of Respiratory Health in Children. *American Journal of Epidemiology* 1994; **139**(11): 1088–99.
283. Mbatchou Ngahane BH, Afane Ze E, Chebu C, et al. Effects of cooking fuel smoke on respiratory symptoms and lung function in semi-rural women in Cameroon. *Int J Occup Environ Health* 2015; **21**(1): 61–5.
284. Ngocho JS, de Jonge MI, Minja L, et al. Modifiable risk factors for community-acquired pneumonia in children under 5 years of age in resource-poor settings: a case-control study. *Tropical medicine & international health : TM & IH* 2019; **24**(4): 484-92.
285. Nisha MK, Alam A, Raynes-Greenow C. Variations in perinatal mortality associated with different polluting fuel types and kitchen location in Bangladesh. *International Journal of Occupational and Environmental Health* 2018; **24**(1-2): 47-54.
286. Nitschke M, Pilotto LS, Attewell RG, et al. A cohort study of indoor nitrogen dioxide and house dust mite exposure in asthmatic children. *J Occup Environ Med* 2006; **48**(5): 462–9.
287. Noonan CW, Ward TJ. Environmental tobacco smoke, woodstove heating and risk of asthma symptoms. *J Asthma* 2007; **44**(9): 735–8.
288. Noonan CW, Ward TJ, Navidi W, Sheppard L. A rural community intervention targeting biomass combustion sources: effects on air quality and reporting of children's respiratory outcomes. *Occupational and environmental medicine* 2012; **69**(5): 354–60.

289. Noorhassim I, Rampal KG, Hashim JH. The relationship between prevalence of asthma and environmental factors in rural households. *The Medical journal of Malaysia* 1995; **50**(3): 263–7.
290. Norback D, Lu C, Zhang YP, et al. Sources of indoor particulate matter (PM) and outdoor air pollution in China in relation to asthma, wheeze, rhinitis and eczema among pre-school children: Synergistic effects between antibiotics use and PM10 and second hand smoke. *Environment International* 2019; **125**: 252-60.
291. North CM, Valeri L, Hunt PW, et al. Cooking fuel and respiratory symptoms among people living with HIV in rural Uganda. *ERJ Open Res* 2017; **3**(2): 00094–2016.
292. Obaseki DO, Awopeju OF, Awokola BI, et al. Domestic solid fuel combustion in an adult population in Nigeria: A cross sectional analysis of association with respiratory symptoms, quality of life and lung function. *Respir Med* 2017; **130**(Department of Medicine, Obafemi Awolowo University, Ile-Ife, Nigeria.): 61–8.
293. Oluwole O, Arinola GO, Huo D, Olopade CO. Biomass fuel exposure and asthma symptoms among rural school children in Nigeria. *J Asthma* 2017; **54**(4): 347–56.
294. Orozco-Levi M, Garcia-Aymerich J, Villar J, Ramirez-Sarmiento A, Anto JM, Gea J. Wood smoke exposure and risk of chronic obstructive pulmonary disease. *Eur Respir J* 2006; **27**(3): 542–6.
295. Ostro BD, Lipsett MJ, Mann JK, Krupnick A, Harrington W. Air-Pollution and Respiratory Morbidity among Adults in Southern California. *American Journal of Epidemiology* 1993; **137**(7): 691–700.
296. Ostro BD, Lipsett MJ, Mann JK, Wiener MB, Selner J. Indoor Air-Pollution and Asthma - Results from a Panel Study. *American Journal of Respiratory and Critical Care Medicine* 1994; **149**(6): 1400–6.
297. Owili PO, Muga MA, Pan WC, Kuo HW. Cooking fuel and risk of under-five mortality in 23 Sub-Saharan African countries: a population-based study. *Int J Environ Health Res* 2017; **27**(3): 191–204.
298. Oxlade O, Murray M. Tuberculosis and poverty: why are the poor at greater risk in India? *PLoS One* 2012; **7**(11): e47533.
299. Ozturk AB, Klcaslan Z, Issever H. Effect of smoking and indoor air pollution on the risk of tuberculosis: smoking, indoor air pollution and tuberculosis. *Tuberkuloz ve Toraks* 2014; **62**(1): 1–6.
300. Padhi BK, Padhy PK. Domestic fuels, indoor air pollution, and children's health. *Ann N Y Acad Sci* 2008; **1140**(5nm, 7506858): 209–17.
301. Pan X, Dong Z, Wang L, Yue W. An Evaluation Of The Indoor/Outdoor Air Pollution And Respiratory Health Of Farmers Living In Rural Areas Of Anhui Province, China. *Indoor Air* 2002: 982–7.
302. Pandey S, Lin Y. Adjusted effects of domestic violence, tobacco use, and indoor air pollution from use of solid fuel on child mortality. *Matern Child Health J* 2013; **17**(8): 1499–507.
303. Panigrahi A, Padhi BK. Chronic bronchitis and airflow obstruction is associated with household cooking fuel use among never-smoking women: a community-based cross-sectional study in Odisha, India. *BMC Public Health* 2018; **18**.
304. Pant KP. Cheaper fuel and higher health costs among the poor in rural Nepal. *Ambio* 2012; **41**(3): 271–83.
305. Patel AB, Dhande LA, Pusdekar YV, Borkar JA, Badhoniya NB, Hibberd PL. Childhood illness in households using biomass fuels in India: secondary data analysis of nationally representative national family health surveys. *International Journal of Occupational and Environmental Health* 2013; **19**(1): 35–42.
306. Patel AB, Meleth S, Pasha O, et al. Impact of exposure to cooking fuels on stillbirths, perinatal, very early and late neonatal mortality - a multicenter prospective cohort study in rural communities in India, Pakistan, Kenya, Zambia and Guatemala. *Matern Health Neonatol Perinatol* 2015; **1**: 18.
307. Patel SK, Patel S, Kumar A. Effects of cooking fuel sources on the respiratory health of children: evidence from the Annual Health Survey, Uttar Pradesh, India. *Public health* 2019; **169**(qi7, 0376507): 59-68.
308. Pathirathna ML, Abeywickrama HM, Sekijima K, et al. Effects of Prenatal Tobacco and Wood-Fuel Smoke Exposure on Birth Weight in Sri Lanka. *Healthcare (Basel)* 2017; **5**(4).
309. Patra S, Sharma S, Behera D. Passive smoking, indoor air pollution and childhood tuberculosis: a case control study. *Indian Journal of Tuberculosis* 2012; **59**(3): 151–5.
310. Patra J, Maher YI, Mishra S, et al. Effects of body mass index, tobacco smoking, alcohol drinking and solid fuel use on the risk of asthma: Individual Participant Data (IPD) meta-analysis of 175 000 individuals from 51 nationally representative surveys. *BMJ Open Respir Res* 2016; **3**(1): e000121.
311. Paulin LM, Williams DL, Peng R, et al. 24-h Nitrogen dioxide concentration is associated with cooking behaviors and an increase in rescue medication use in children with asthma. *Environ Res* 2017; **159**(Johns Hopkins Pulmonary/Critical Care, Baltimore, Maryland, USA.): 118–23.
312. Peabody JW, Riddell TJ, Smith KR, et al. Indoor air pollution in rural China: cooking fuels, stoves, and health status. *Arch Environ Occup Health* 2005; **60**(2): 86–95.
313. Perez-Padilla R, Regalado J, Vedal S, et al. Exposure to biomass smoke and chronic airway disease in Mexican women. A case-control study. *Am J Respir Crit Care Med* 1996; **154**(3 Pt 1): 701–6.
314. Perez-Padilla R, Perez-Guzman C, Baez-Saldana R, Torres-Cruz A. Cooking with biomass stoves and tuberculosis: a case control study. *Int J Tuberc Lung Dis* 2001; **5**(5): 441–7.
315. Pershagen G, Rylander E, Norberg S, Eriksson M, Nordvall SL. Air pollution involving nitrogen dioxide exposure and wheezing bronchitis in children. *Int J Epidemiol* 1995; **24**(6): 1147–53.

316. Phillips DIW, Osmond C, Southall H, Aucott P, Jones A, Holgate ST. Evaluating the long-term consequences of air pollution in early life: geographical correlations between coal consumption in 1951/1952 and current mortality in England and Wales. *BMJ Open* 2018; **8**(4).
317. Phoa LL, Toelle BG, Ng K, Marks GB. Effects of gas and other fume emitting heaters on the development of asthma during childhood. *Thorax* 2004; **59**(9): 741–5.
318. Phukan RK, Saikia BJ, Borah PK, Zomawia E, Sekhon GS, Mahanta J. Role of Household Exposure, Dietary Habits and Glutathione S-Transferases M1, T1 Polymorphisms in Susceptibility to Lung Cancer among Women in Mizoram India. *Asian Pacific Journal of Cancer Prevention* 2014; **15**(7): 3253–60.
319. Piekarska B, Stankiewicz-Choroszucha BL, Sybilski AJ, et al. Effect of indoor air quality on the natural history of asthma in an urban population in Poland. *Allergy and Asthma Proceedings* 2018; **39**(6): E64-E70.
320. Pokharel PK, Kabra SK, Kapoor SK, Pandey RM. Risk factors associated with bronchial asthma in school going children of rural Haryana. *Indian J Pediatr* 2001; **68**(2): 103–6.
321. Pokhrel AK, Bates MN, Verma SC, Joshi HS, Sreeramareddy CT, Smith KR. Tuberculosis and indoor biomass and kerosene use in Nepal: a case-control study. *Environ Health Perspect* 2010; **118**(4): 558–64.
322. Ponsonby AL, Couper D, Dwyer T, Carmichael A, Kemp A, Cochrane J. The relation between infant indoor environment and subsequent asthma. *Epidemiology (Cambridge, Mass)* 2000; **11**(2): 128–35.
323. Ponsonby AL, Glasgow N, Gatenby P, et al. The relationship between low level nitrogen dioxide exposure and child lung function after cold air challenge. *Clinical and Experimental Allergy* 2001; **31**(8): 1205–12.
324. Pope CA, 3rd, Xu X. Passive cigarette smoke, coal heating, and respiratory symptoms of nonsmoking women in China. *Environ Health Perspect* 1993; **101**(4): 314–6.
325. PrayGod G, Mukerebe C, Magawa R, Jeremiah K, Torok ME. Indoor Air Pollution and Delayed Measles Vaccination Increase the Risk of Severe Pneumonia in Children: Results from a Case-Control Study in Mwanza, Tanzania. *PLoS One* 2016; **11**(8): e0160804.
326. Prietsch SO, Fischer GB, Cesar JA, et al. [Risk factors for recurrent wheezing in children under 13 years old in the South of Brazil]. *Rev Panam Salud Publica* 2006; **20**(5): 331–7.
327. Qian Z, Chapman RS, Tian Q, Chen Y, Lioy PJ, Zhang J. Effects of air pollution on children's respiratory health in three Chinese cities. *Arch Environ Health* 2000; **55**(2): 126–33.
328. Qian Z, Zhang J, Korn LR, Wei F, Chapman RS. Factor analysis of household factors: are they associated with respiratory conditions in Chinese children? *International journal of epidemiology* 2004; **33**(3): 582–8.
329. Qian Z, He Q, Kong L, et al. Respiratory responses to diverse indoor combustion air pollution sources. *Indoor Air* 2007; **17**(2): 135–42.
330. Qin Y, Zhou B, Xu Z. [A case-control study on risk factor of lung cancer in female nonsmokers]. *Zhongguo Fei Ai Za Zhi* 2002; **5**(2): 98–100.
331. Qu WH, Yan ZJ, Qu GH, Ikram M. Household Solid Fuel Use and Cardiovascular Disease in Rural Areas in Shanxi, China. *Iranian Journal of Public Health* 2015; **44**(5): 625–38.
332. Rabbani U, Sahito A, Nafees AA, Kazi A, Fatmi Z. Pulmonary Tuberculosis Is Associated With Biomass Fuel Use Among Rural Women in Pakistan: An Age- and Residence-Matched Case-Control Study. *Asia Pac J Public Health* 2017; **29**(3): 211–8.
333. Rahut DB, Ali A, Behera B. Domestic use of dirty energy and its effects on human health: empirical evidence from Bhutan. *International Journal of Sustainable Energy* 2017; **36**(10): 983–93.
334. Ram PK, Dutt D, Silk BJ, et al. Household air quality risk factors associated with childhood pneumonia in urban Dhaka, Bangladesh. *Am J Trop Med Hyg* 2014; **90**(5): 968–75.
335. Ramanakumar AV, Parent ME, Siemiatycki J. Risk of lung cancer from residential heating and cooking fuels in Montreal, Canada. *Am J Epidemiol* 2007; **165**(6): 634–42.
336. Ramani VK, Pattankar J, Puttahonnappa SK. Acute Respiratory Infections among Under-Five Age Group Children at Urban Slums of Gulbarga City: A Longitudinal Study. *J Clin Diagn Res* 2016; **10**(5): LC08–13.
337. Rana J, Uddin J, Peltier R, Oulhote Y. Associations between Indoor Air Pollution and Acute Respiratory Infections among Under-Five Children in Afghanistan: Do SES and Sex Matter? *International journal of environmental research and public health* 2019; **16**(16).
338. Raspanti GA, Hashibe M, Siwakoti B, et al. Household air pollution and lung cancer risk among never-smokers in Nepal. *Environ Res* 2016; **147**(Maryland Institute for Applied Environmental Health, University of Maryland School of Public Health, College Park, MD 20742, USA.): 141–5.
339. Regalado J, Perez-Padilla R, Sansores R, et al. The effect of biomass burning on respiratory symptoms and lung function in rural Mexican women. *Am J Respir Crit Care Med* 2006; **174**(8): 901–5.
340. Rehfuess EA, Tzala L, Best N, Briggs DJ, Joffe M. Solid fuel use and cooking practices as a major risk factor for ALRI mortality among African children. *Journal of Epidemiology and Community Health* 2009; **63**(11): 887–92.
341. Rey-Ares L, Irazola V, Althabe F, et al. Lower tract respiratory infection in children younger than 5years of age and adverse pregnancy outcomes related to household air pollution in Bariloche (Argentina) and Temuco (Chile). *Indoor Air* 2016; **26**(6): 964–75.

342. Rinne ST, Rodas EJ, Rinne ML, Simpson JM, Glickman LT. Use of biomass fuel is associated with infant mortality and child health in trend analysis. *Am J Trop Med Hyg* 2007; **76**(3): 585–91.
343. Riojas-Rodriguez H, Romano-Riquer P, Santos-Burgoa C, Smith KR. Household firewood use and the health of children and women of Indian communities in Chiapas, Mexico. *International journal of occupational and environmental health* 2001; **7**(1): 44–53.
344. Robin LF, Lees PSJ, Winget M, et al. Wood-burning stoves and lower respiratory illnesses in Navajo children. *Pediatric Infectious Disease Journal* 1996; **15**(10): 859–65.
345. Romieu I, Riojas-Rodriguez H, Marron-Mares AT, Schilmann A, Perez-Padilla R, Masera O. Improved biomass stove intervention in rural Mexico: impact on the respiratory health of women. *Am J Respir Crit Care Med* 2009; **180**(7): 649–56.
346. Rudnai P, Varro MJ, Macsik A, Szabo E, Benyi M. Respiratory symptoms of school children and their home environment in Northern Transdanubia, Hungary. *International Journal of Environment and Health* 2008; **2**(3/4): 386–96.
347. Rumchev K, Win T, Bertolatti D, Dhaliwal S. Prevalence of respiratory symptoms among children in rural Myanmar-disease burden assessment attributable to household biomass smoke. *Indoor and Built Environment* 2016; **25**(5): 728–36.
348. Rumchev K, Zhao Y, Spickett J. Health Risk Assessment of Indoor Air Quality, Socioeconomic and House Characteristics on Respiratory Health among Women and Children of Tirupur, South India. *International journal of environmental research and public health* 2017; **14**(4): 429.
349. Rylance S, Nightingale R, Naunje A, et al. Lung health and exposure to air pollution in Malawian children (CAPS): a cross-sectional study. *Thorax* 2019; **74**(11): 1070-7.
350. Salo PM, Xia J, Johnson CA, et al. Respiratory symptoms in relation to residential coal burning and environmental tobacco smoke among early adolescents in Wuhan, China: a cross-sectional study. *Environmental health : a global access science source* 2004; **3**(1): 14.
351. Sama SR, Kriebel D, Gore RJ, DeVries R, Rosiello R. Environmental triggers of COPD symptoms: a case cross-over study. *BMJ Open Respir Res* 2017; **4**(1): e000179.
352. Samet JM, Lambert WE, Skipper BJ, et al. Nitrogen dioxide and respiratory illnesses in infants. *Am Rev Respir Dis* 1993; **148**(5): 1258–65.
353. Samuel OW, Oni GA, Samir KC, Wurzer M, Akinyemi AI. Household use of solid fuel for cooking and under-five mortality in Nigeria. *African Population Studies* 2018; **32**(1 Suppl.): 4034-42.
354. Sana A, Meda N, Badoum G, Kafando B, Bouland C. Primary Cooking Fuel Choice and Respiratory Health Outcomes among Women in Charge of Household Cooking in Ouagadougou, Burkina Faso: Cross-Sectional Study. *International journal of environmental research and public health* 2019; **16**(6).
355. Sanbata H, Asfaw A, Kumie A. Association of biomass fuel use with acute respiratory infections among under- five children in a slum urban of Addis Ababa, Ethiopia. *Bmc Public Health* 2014; **14**(Journal Article).
356. Sanya RE, Kirenga BJ, Worodria W, Okot-Nwang M. Risk factors for asthma exacerbation in patients presenting to an emergency unit of a national referral hospital in Kampala, Uganda. *Afr Health Sci* 2014; **14**(3): 707–15.
357. Sapkota A, Gajalakshmi V, Jetly DH, et al. Indoor air pollution from solid fuels and risk of hypopharyngeal/laryngeal and lung cancers: a multicentric case-control study from India. *Int J Epidemiol* 2008; **37**(2): 321–8.
358. Sasco AJ, Merrill RM, Dari I, et al. A case-control study of lung cancer in Casablanca, Morocco. *Cancer Causes Control* 2002; **13**(7): 609–16.
359. Savitha MR, Nandeeshwara SB, Pradeep Kumar MJ, ul-Haque F, Raju CK. Modifiable risk factors for acute lower respiratory tract infections. *Indian J Pediatr* 2007; **74**(5): 477–82.
360. Schei MA, Hessen JO, Smith KR, Bruce N, McCracken J, Lopez V. Childhood asthma and indoor woodsmoke from cooking in Guatemala. *J Expo Anal Environ Epidemiol* 2004; **14 Suppl 1**(1}): S110–7.
361. Sezer H, Akkurt I, Guler N, Marakoglu K, Berk S. A case-control study on the effect of exposure to different substances on the development of COPD. *Ann Epidemiol* 2006; **16**(1): 59–62.
362. Shah N, Ramankutty V, Premila PG, Sathy N. Risk factors for severe pneumonia in children in south Kerala: a hospital-based case-control study. *J Trop Pediatr* 1994; **40**(4): 201–6.
363. Sharma S, Sethi GR, Rohtagi A, et al. Indoor air quality and acute lower respiratory infection in Indian urban slums. *Environ Health Perspect* 1998; **106**(5): 291–7.
364. Sharma D, Kuppusamy K, Bhoorasamy A. Prevalence of acute respiratory infections (ARI) and their determinants in under five children in urban and rural areas of Kancheepuram district, South India. *Annals of Tropical Medicine and Public Health* 2013; **6**(5): 513–8.
365. Shen M, Chapman RS, Vermeulen R, et al. Coal use, stove improvement, and adult pneumonia mortality in Xuanwei, China: a retrospective cohort study. *Environ Health Perspect* 2009; **117**(2): 261–6.
366. Shetty N, Shemko M, Vaz M, D'Souza G. An epidemiological evaluation of risk factors for tuberculosis in South India: a matched case control study. *Int J Tuberc Lung Dis* 2006; **10**(1): 80–6.
367. Shima M, Adachi M. Effect of outdoor and indoor nitrogen dioxide on respiratory symptoms in schoolchildren. *Int J Epidemiol* 2000; **29**(5): 862–70.

368. Shirinde J, Wichmann J, Voyi K. Association between wheeze and selected air pollution sources in an air pollution priority area in South Africa: a cross-sectional study. *Environ Health* 2014; **13**(1): 32.
369. Shrestha IL, Shrestha SL. Indoor air pollution from biomass fuels and respiratory health of the exposed population in Nepalese households. *International Journal of Occupational and Environmental Health* 2005; **11**(2): 150–60.
370. Siddharthan T, Grigsby MR, Goodman D, et al. Association between Household Air Pollution Exposure and Chronic Obstructive Pulmonary Disease Outcomes in 13 Low- and Middle-Income Country Settings. *Am J Respir Crit Care Med* 2018; **197**(5): 611–20.
371. Siddiqui AR, Lee K, Gold EB, Bhutta ZA. Eye and respiratory symptoms among women exposed to wood smoke emitted from indoor cooking: a study from southern Pakistan. *Energy for Sustainable Development* 2005; **9**(3): 58–66.
372. Siddiqui AR, Gold EB, Yang X, Lee K, Brown KH, Bhutta ZA. Prenatal exposure to wood fuel smoke and low birth weight. *Environ Health Perspect* 2008; **116**(4): 543–9.
373. Sikolia DN, Mwololo K, Cherop H, et al. The prevalence of acute respiratory infections and the associated risk factors: a study of children under five years of age in Kibera Lindi Village, Nairobi, Kenya. *Journal of National Institute of Public Health* 2002; **51**(1): 67–72.
374. Singh D, Arora V, Sobti PC. Chronic/recurrent cough in rural children in Ludhiana, Punjab. *Indian Pediatr* 2002; **39**(1): 23–9.
375. Sloan CD, Andrew AS, Gruber JF, et al. Indoor and Outdoor Air Pollution and Lung Cancer in New Hampshire and Vermont. *Toxicol Environ Chem* 2012; **94**(3): 605–15.
376. Smith BJ, Nitschke M, Pilotto LS, Ruffin RE, Pisaniello DL, Willson KJ. Health effects of daily indoor nitrogen dioxide exposure in people with asthma. *Eur Respir J* 2000; **16**(5): 879–85.
377. Smith KR, McCracken JP, Weber MW, et al. Effect of reduction in household air pollution on childhood pneumonia in Guatemala (RESPIRE): a randomised controlled trial. *Lancet* 2011; **378**(9804): 1717–26.
378. Smith-Sivertsen T, Diaz E, Pope D, et al. Effect of reducing indoor air pollution on women's respiratory symptoms and lung function: the RESPIRE Randomized Trial, Guatemala. *Am J Epidemiol* 2009; **170**(2): 211–20.
379. Sobue T. Association of indoor air pollution and lifestyle with lung cancer in Osaka, Japan. *Int J Epidemiol* 1990; **19 Suppl 1**(gr6, 7802871): S62–6.
380. Solis-Soto MT, Patino A, Nowak D, Radon K. Association between environmental factors and current asthma, rhinoconjunctivitis and eczema symptoms in school-aged children from Oropeza Province--Bolivia: a cross-sectional study. *Environ Health* 2013; **12**(Journal Article): 95.
381. Sood A, Petersen H, Blanchette CM, et al. Wood smoke exposure and gene promoter methylation are associated with increased risk for COPD in smokers. *Am J Respir Crit Care Med* 2010; **182**(9): 1098–104.
382. Soto-Moreno JA, Ballester-Diez F. [Indoor air pollution in extremely poor Colombian households]. *Rev Salud Publica (Bogota)* 2013; **15**(1): 80–9.
383. Speizer FE, Ferris B, Jr., Bishop YM, Spengler J. Respiratory disease rates and pulmonary function in children associated with NO2 exposure. *Am Rev Respir Dis* 1980; **121**(1): 3–10.
384. Spengler JD, Jaakkola JJ, Parise H, Katsnelson BA, Privalova LI, Kosheleva AA. Housing characteristics and children's respiratory health in the Russian Federation. *Am J Public Health* 2004; **94**(4): 657–62.
385. Sreeramareddy CT, Shidhaye RR, Sathiakumar N. Association between biomass fuel use and maternal report of child size at birth--an analysis of 2005-06 India Demographic Health Survey data. *BMC Public Health* 2011; **11**(Journal Article): 403.
386. Stankovic A, Nikolic M, Arandjelovic M. Effects of indoor air pollution on respiratory symptoms of non-smoking women in Nis, Serbia. *Multidiscip Respir Med* 2011; **6**(6): 351–5.
387. Stankovic A, Mitrovic V, Zivadinovic R. [Influence of air pollution on pregnant women's health and pregnancy outcomes]. *Medicinski pregled* 2011; **64**(5-6): 279–84.
388. Stein L, Urban MI, Weber M, et al. Effects of tobacco smoking on cancer and cardiovascular disease in urban black South Africans. *Br J Cancer* 2008; **98**(9): 1586–92.
389. Strachan DP, Carey IM. Home environment and severe asthma in adolescence: a population based case-control study. *BMJ* 1995; **311**(7012): 1053–6.
390. Sukhsohale ND, Narlawar UW, Phatak MS. Indoor air pollution from biomass combustion and its adverse health effects in central India: an exposure-response study. *Indian J Community Med* 2013; **38**(3): 162–7.
391. Sun XW. [Heating fuels and respiratory diseases in the risks of female lung cancer]. *Zhonghua Zhong Liu Za Zhi* 1992; **13**(6): 413–5.
392. Sun X, Dai X, Shi Y, Lin Y. A case-control study on the relationship among indoor air pollution, depression and oncogenesis of lung cancer. *Chinese Journal of Lung Cancer* 2002; **5**(2): 101–3.
393. Sunyer J. Nitrogen dioxide is not associated with respiratory infection during the first year of life. *International Journal of Epidemiology* 2004; **33**(1): 116–20.
394. Suryadhi MAH, Abudureyimu K, Kashima S, Yorifuji T. Effects of Household Air Pollution From Solid Fuel Use and Environmental Tobacco Smoke on Child Health Outcomes in Indonesia. *Journal of occupational and environmental medicine* 2019; **61**(4): 335-9.

395. Svendsen ER, Gonzales M, Commodore A. The role of the indoor environment: Residential determinants of allergy, asthma and pulmonary function in children from a US-Mexico border community. *The Science of the total environment* 2018; **616-617**(Journal Article): 1513–23.
396. Tamire M, Addissie A, Kumie A, et al. Respiratory Symptoms and Lung Function among Ethiopian Women in Relation to Household Fuel Use. *International journal of environmental research and public health* 2020; **17**(1).
397. Tan WC, Sin DD, Bourbeau J, et al. Characteristics of COPD in never-smokers and ever-smokers in the general population: results from the CanCOLD study. *Thorax* 2015; **70**(9): 822–9.
398. Tavernier G, Fletcher G, Gee I, et al. IPEADAM study: Indoor endotoxin exposure, family status, and some housing characteristics in English children. *Journal of Allergy and Clinical Immunology* 2006; **117**(3): 656–62.
399. Taylor ET, Nakai S. Prevalence of acute respiratory infections in women and children in Western Sierra Leone due to smoke from wood and charcoal stoves. *International journal of environmental research and public health* 2012; **9**(6): 2252–65.
400. Tazinya AA, Halle-Ekane GE, Mbuagbaw LT, Abanda M, Atashili J, Obama MT. Risk factors for acute respiratory infections in children under five years attending the Bamenda Regional Hospital in Cameroon. *BMC Pulm Med* 2018; **18**(1): 7.
401. Thacher JD, Emmelin A, Madaki AJ, Thacher TD. Biomass fuel use and the risk of asthma in Nigerian children. *Respir Med* 2013; **107**(12): 1845–51.
402. Thompson LM, Bruce N, Eskenazi B, Diaz A, Pope D, Smith KR. Impact of reduced maternal exposures to wood smoke from an introduced chimney stove on newborn birth weight in rural Guatemala. *Environ Health Perspect* 2011; **119**(10): 1489–94.
403. Tielsch JM, Katz J, Thulasiraj RD, et al. Exposure to indoor biomass fuel and tobacco smoke and risk of adverse reproductive outcomes, mortality, respiratory morbidity and growth among newborn infants in south India. *International Journal of Epidemiology* 2009; **38**(5): 1351–63.
404. Tin ST, Woodward A, Saraf R, et al. Internal living environment and respiratory disease in children: findings from the Growing Up in New Zealand longitudinal child cohort study. *Environmental Health* 2016; **15**(120).
405. Trang N, Lurie M, Gomez M, Reddy A, Pandya K, Medvesky M. The National Asthma Survey - New York State: association of the home environment with current asthma status. *Public Health Reports* 2010; **125**(6): 877–87.
406. Tripathy JP, Mishra S. Causes and Predictors of Neonatal, Post-neonatal and Maternal Deaths in India: Analysis of a Nationwide District-Level Household Survey-4 (DLHS-4), 2012-13. *J Trop Pediatr* 2017; **63}**(6}): 431–9}.
407. Tse LA, Yu IT, Qiu H, Au JS, Wang XR. A case-referent study of lung cancer and incense smoke, smoking, and residential radon in Chinese men. *Environ Health Perspect* 2011; **119**(11): 1641–6.
408. Tumwesigire SG, Barton T. Environmental Risk-Factors for Acute Respiratory-Infections among Children of Military Personnel in Uganda. *East African Medical Journal* 1995; **72**(5): 290–4.
409. Tuthill RW. Woodstoves, formaldehyde, and respiratory disease. *Am J Epidemiol* 1984; **120**(6): 952–5.
410. Upadhyay AK, Singh A, Kumar K, Singh A. Impact of indoor air pollution from the use of solid fuels on the incidence of life threatening respiratory illnesses in children in India. *Bmc Public Health* 2015; **15**(300).
411. Uzun K, Ozbay B, Ceylan E, Gencer M, Zehir I. Prevalence of chronic bronchitis-asthma symptoms in biomass fuel exposed females. *Environ Health Prev Med* 2003; **8**(1): 13–7.
412. van Gemert F, Chavannes N, Kirenga B, et al. Socio-economic factors, gender and smoking as determinants of COPD in a low-income country of sub-Saharan Africa: FRESH AIR Uganda. *NPJ Prim Care Respir Med* 2016; **26**(101631999): 16050.
413. Van Miert E, Sardella A, Nickmilder M, Bernard A. Respiratory effects associated with wood fuel use: a cross-sectional biomarker study among adolescents. *Pediatr Pulmonol* 2012; **47**(4): 358–66.
414. van Strien RT, Gent JF, Belanger K, Triche E, Bracken MB, Leaderer BP. Exposure to NO2 and nitrous acid and respiratory symptoms in the first year of life. *Epidemiology (Cambridge, Mass)* 2004; **15**(4): 471–8.
415. Van Vliet EDS, Kinney PL, Owusu-Agyei S, et al. Current respiratory symptoms and risk factors in pregnant women cooking with biomass fuels in rural Ghana. *Environment International* 2019; **124**: 533-40.
416. Guddattu V, Swathi A, Nair NS. Household and environment factors associated with asthma among Indian women: a multilevel approach. *J Asthma* 2010; **47**(4): 407–11.
417. Viegi G, Paoletti P, Carrozzi L, et al. Effects of home environment on respiratory symptoms and lung function in a general population sample in north Italy. *Eur Respir J* 1991; **4**(5): 580–6.
418. Venn AJ, Yemaneberhan H, Bekele Z, Lewis SA, Parry E, Britton J. Increased risk of allergy associated with the use of kerosene fuel in the home. *American Journal of Respiratory and Critical Care Medicine* 2001; **164**(9): 1660–4.
419. Victora CG, Fuchs SC, Flores JAC, Fonseca W, Kirkwood B. Risk-Factors for Pneumonia among Children in a Brazilian Metropolitan-Area. *Pediatrics* 1994; **93**(6): 977–85.
420. Viegi G, Carrozzi L, Paoletti P, et al. Effects of the home environment on respiratory symptoms of a general population sample in middle Italy. *Arch Environ Health* 1992; **47**(1): 64–70.

421. Volkmer RE, Ruffin RE, Wigg NR, Davies N. The prevalence of respiratory symptoms in South Australian preschool children. II. Factors associated with indoor air quality. *J Paediatr Child Health* 1995; **31**(2): 116–20.
422. von Maffei J, Beckett WS, Belanger K, et al. Risk Factors for Asthma Prevalence Among Urban and Nonurban African American Children. *Journal of Asthma* 2001; **38**(7): 555–64.
423. von Mutius E, Illi S, Nicolai T, Martinez FD. Relation of indoor heating with asthma, allergic sensitisation, and bronchial responsiveness: survey of children in south Bavaria. *BMJ* 1996; **312**(7044): 1448–50.
424. Wafula EM, Kinyanjui MM, Nyabola L, Tenambergen ED. Effect of improved stoves on prevalence of acute respiration infection and conjunctivitis among children and women in a rural community in Kenya. *East African Medical Journal* 2000; **77**(1): 37–41.
425. Walker ES, Clark ML, Young BN, et al. Exposure to household air pollution from biomass cookstoves and self-reported symptoms among women in rural Honduras. *International Journal of Environmental Health Research* 2020; **30**(2): 160-73.
426. Wang GX. [Multivariate analyses of causal factors included cooking oil fume and others in matched case-control study of lung cancer]. *Zhonghua Yu Fang Yi Xue Za Zhi* 1992; **26**(2): 89–91.
427. Wang TJ, Zhou BS, Shi JP. Lung cancer in nonsmoking Chinese women: a case-control study. *Lung Cancer* 1996; **14 Suppl 1**(b3u, 8800805): S93–8.
428. Wang TT, Zhao ZH, Yao H, et al. Housing characteristics and indoor environment in relation to children's asthma, allergic diseases and pneumonia in Urumqi, China. *Chinese Science Bulletin* 2013; **58**(34): 4237–44.
429. Ware JH, Dockery DW, Spiro A, 3rd, Speizer FE, Ferris BG, Jr. Passive smoking, gas cooking, and respiratory health of children living in six cities. *Am Rev Respir Dis* 1984; **129**(3): 366–74.
430. Ware DN, Lewis J, Hopkins S, et al. Household reporting of childhood respiratory health and air pollution in rural Alaska Native communities. *Int J Circumpolar Health* 2014; **73**(Journal Article): 1–10.
431. Wayse V, Yousafzai A, Mogale K, Filteau S. Association of subclinical vitamin D deficiency with severe acute lower respiratory infection in Indian children under 5 y. *Eur J Clin Nutr* 2004; **58**(4): 563–7.
432. Weber E, Adu-Bonsaffoh K, Vermeulen R, et al. Household fuel use and adverse pregnancy outcomes in a Ghanaian cohort study. *Reproductive Health* 2020; **17**(1).
433. Wichmann J, Voyi KVV. Impact of cooking and heating fuel use on acute respiratory health of preschool children in South Africa. *Southern African Journal of Epidemiology & Infection* 2006; **21**(2): 48–54.
434. Wichmann J, Voyi KV. Influence of cooking and heating fuel use on 1-59 month old mortality in South Africa. *Matern Child Health J* 2006; **10**(6): 553–61.
435. Wichmann J, Wolvaardt JE, Maritz C, Voyi KVV. Household Conditions, Eczema Symptoms and Rhinitis Symptoms: Relationship with Wheeze and Severe Wheeze in Children Living in the Polokwane Area, South Africa. *Maternal and Child Health Journal* 2009; **13**(1): 107–18.
436. Willers SM, Brunekreef B, Oldenwening M, et al. Gas cooking, kitchen ventilation, and asthma, allergic symptoms and sensitization in young children--the PIAMA study. *Allergy* 2006; **61**(5): 563–8.
437. Withers NJ, Low L, Holgate ST, Clough JB. The natural history of respiratory symptoms in a cohort of adolescents. *Am J Respir Crit Care Med* 1998; **158**(2): 352–7.
438. Woldeamanuel GG, Mingude AB, Geta TG. Prevalence of chronic obstructive pulmonary disease (COPD) and its associated factors among adults in Abeshge District, Ethiopia: a cross sectional study. *BMC Pulm Med* 2019; **19**(1): 181.
439. Woldesemayat EM, Datiko DG, Lindtjorn B. Use of biomass fuel in households is not a risk factor for pulmonary tuberculosis in South Ethiopia. *Int J Tuberc Lung Dis* 2014; **18**(1): 67–72.
440. Wolff PT, Arison L, Rahajamiakatra A, Raserijaona F, Niggemann B. High asthma prevalence and associated factors in urban malagasy schoolchildren. *J Asthma* 2012; **49**(6): 575–80.
441. Wong GW, Ko FW, Hui DS, et al. Factors associated with difference in prevalence of asthma in children from three cities in China: multicentre epidemiological survey. *BMJ* 2004; **329**(7464): 486.
442. Wong GWK, Brunekreef B, Ellwood P, et al. Cooking fuels and prevalence of asthma: a global analysis of phase three of the International Study of Asthma and Allergies in Childhood (ISAAC). *Lancet Respiratory Medicine* 2013; **1**(5): 386–94.
443. Wong JYY, Downward GS, Hu W, et al. Lung cancer risk by geologic coal deposits: A case-control study of female never-smokers from Xuanwei and Fuyuan, China. *International journal of cancer* 2019; **144**(12): 2918-27.
444. Wu AH, Henderson BE, Pike MC, Yu MC. Smoking and other risk factors for lung cancer in women. *J Natl Cancer Inst* 1985; **74**(4): 747–51.
445. Wu J, Tang C, Zhuang Z. Effect of indoor air pollution related to traffic and fuel gas using for cooking on respiratory diseases in children and teenagers in urban area of Shanghai. *Journal of Environment and Health* 2010; **27**(3): 244–7.
446. Wu ZY, Zhang SJ, Tang YT, et al. Indoor environment in relation to recurrent childhood pneumonia in Southern China. *Building and Environment* 2020; **172**.
447. Wu-Williams AH, Dai XD, Blot W, et al. Lung cancer among women in north-east China. *British journal of cancer* 1990; **62**(6): 982–7.

448. Wylie BJ, Coull BA, Hamer DH, et al. Impact of biomass fuels on pregnancy outcomes in central East India. *Environ Health* 2014; **13**(1): 1.
449. Xu ZY, Blot WJ, Xiao HP, et al. Smoking, air pollution, and the high rates of lung cancer in Shenyang, China. *J Natl Cancer Inst* 1989; **81**(23): 1800–6.
450. Xu X, Wang L. Association of indoor and outdoor particulate level with chronic respiratory illness. *Am Rev Respir Dis* 1993; **148**(6 Pt 1): 1516–22.
451. Xu X, Niu T, Christiani DC, et al. Occupational and Environmental Risk Factors for Asthma in Rural Communities in China. *Int J Occup Environ Health* 1996; **2**(3): 172–6.
452. Yang CY, Chiu JF, Cheng MF, Lin MC. Effects of indoor environmental factors on respiratory health of children in a subtropical climate. *Environmental Research* 1997; **75**(1): 49–55.
453. Yang L, Lu X, Deng J, et al. Risk factors shared by COPD and lung cancer and mediation effect of COPD: two center case-control studies. *Cancer Causes Control* 2015; **26**(1): 11–24.
454. Yu IT, Li AM, Goggins W, et al. Association of wheeze during the first 18 months of life with indoor nitrogen dioxide, formaldehyde, and family history of asthma: a prospective cohort study. *Hong Kong medical journal = Xianggang yi xue za zhi* 2017; **23**(3 Supplement 2): 19-23.
455. Yu K, Lv J, Qiu GK, et al. Cooking fuels and risk of all-cause and cardiopulmonary mortality in urban China: a prospective cohort study. *Lancet Global Health* 2020; **8**(3): E430-E9.
456. Yucra S, Tapia V, Steenland K, Naeher LP, Gonzales GF. Association Between Biofuel Exposure and Adverse Birth Outcomes at High Altitudes in Peru: A Matched Case-control Study. *International Journal of Occupational and Environmental Health* 2011; **17**(4): 307–13.
457. Yusuf S, Joseph P, Rangarajan S, et al. Modifiable risk factors, cardiovascular disease, and mortality in 155 722 individuals from 21 high-income, middle-income, and low-income countries (PURE): a prospective cohort study. *Lancet* 2020; **395**(10226): 795-808.
458. Zacharasiewicz A, Zidek T, Haidinger G, Waldhor T, Suess G, Vutuc C. Indoor factors and their association to respiratory symptoms suggestive of asthma in Austrian children aged 6-9 years. *WIENER KLINISCHE WOCHENSCHRIFT}* 1999; **111}**(21}): 882–6}.
459. Zejda JE, Kowalska M. Risk factors for asthma in school children--results of a seven-year follow-up. *Cent Eur J Public Health* 2003; **11**(3): 149–54.
460. Zha ZQ, Leng RX, Xu W, et al. Prevalence and risk factors of chronic obstructive pulmonary disease in Anhui Province, China: a population-based survey. *BMC Pulm Med* 2019; **19**.
461. Zhang ZF, Yu SZ, Zhou GD. Indoor air pollution of coal fumes as a risk factor of stroke, Shanghai. *Am J Public Health* 1988; **78**(8): 975–7.
462. Zhang LX, Enarson DA, He GX, Li B, Chan-Yeung M. Occupational and environmental risk factors for respiratory symptoms in rural Beijing, China. *European Respiratory Journal* 2002; **20**(6): 1525–31.
463. Zhao X, Huang X, Huang P, et al. [Interaction between exposure to environmental smoke and the family history of chronic bronchitis (CB) on the risk of CB in rural females in Liuyang city, Hunan province]. *Zhonghua Liu Xing Bing Xue Za Zhi* 2009; **30**(12): 1258–60.
464. Zheng T, Niu S, Lu B, et al. Childhood asthma in Beijing, China: a population-based case-control study. *Am J Epidemiol* 2002; **156**(10): 977–83.
465. Zheng XH, Qian H, Zhao YL, et al. Home risk factors for childhood pneumonia in Nanjing, China. *Chinese Science Bulletin* 2013; **58**(34): 4230–6.
466. Zhong LJ, Goldberg MS, Gao YT, Jin F. Lung cancer and indoor air pollution arising from Chinese style cooking among nonsmoking women living in Shanghai, China. *Epidemiology (Cambridge, Mass)* 1999; **10**(5): 488–94.
467. Zhong N, Wang C, Yao W, et al. Prevalence of chronic obstructive pulmonary disease in China: a large, population-based survey. *Am J Respir Crit Care Med* 2007; **176**(8): 753–60.
468. Zhou H. The Health Effects of Using Coal for Cooking to Infants. *Journal of Environment and Health* 1994; **3**.
469. Zhou X, Jin Y, He X. [A study on the relationship between in-door air pollution and chronic obstructive pulmonary disease in Xuanwei County]. *Zhonghua yu fang yi xue za zhi [Chinese journal of preventive medicine]* 1995; **29**(1): 38–40.
470. Zhou Y, Wang C, Yao W, et al. COPD in Chinese nonsmokers. *Eur Respir J* 2009; **33**(3): 509–18.
471. Zhou C, Baiz N, Zhang T, Banerjee S, Annesi-Maesano I, Group EM-CCS. Modifiable exposures to air pollutants related to asthma phenotypes in the first year of life in children of the EDEN mother-child cohort study. *BMC Public Health* 2013; **13**(Journal Article): 506.
472. Zhou Y, Zou Y, Li X, et al. Lung function and incidence of chronic obstructive pulmonary disease after improved cooking fuels and kitchen ventilation: a 9-year prospective cohort study. *PLoS Med* 2014; **11**(3): e1001621.
